# Supplementary material for: ELAVL1 and ELAVL4 Are Required for Musashi-Dependent Translational Activation
Source: Biomolecules. 2026 Jul 31;16(8):1119. doi: 10.3390/biom16081119 (PMC13510767; doi:10.3390/biom16081119)
Supplement: Supplementary file 1 [file biomolecules-16-01119-s001.zip › Bronson etal Supplementary Tables and Legends FINAL.pdf]

ELAVL1 and ELAVL4 are required for Musashi-dependent translational activation

Katherine Bronson[1,2,#], Milla M. Reddick[1,#], Kenzie B. MacNicol[1], Cole Bolen[1], Linda Hardy[1], Alex Lagasse[1], Angela K. Odle[1], Gwen V. Childs[1], Melanie C. MacNicol[1,3,$] and Angus M. MacNicol[1*,$]

Supplementary Data Table Legends:

Supplementary Table S1

An excel file listing all proteins identified in MSI1/2 immunoprecipitation vs control IgG immunoprecipitations from mouse whole pituitaries. The data includes 3 independent replicates for each immunoprecipitation condition.

Supplementary Table S2

An excel file showing only proteins from Supplemental Table 1 which showed enrichment in MSI1/2 over control IgG immunoprecipitations with a FDR p-value <0.055 and a positive log fold change > 2.

Protein Annotation										Log2 Cyclic Loess Normalized Intensities						Musashi_vs_IgG									
id	rotein Nam	ession Num	lecular Wei	ein Group S	ied Peptide	Exclusivity	UniProt ID	Gene name	Description	E1_MSI12	E1_IgG	E2_MSI12	E2_IgG	E3_MSI12	E3_IgG	logFC	CI.L	CI.R	AveExpr	t	B	P.Value	adj.P.Val	sig.PVal	sig.FDR
1223	sp|Q9JKC8	AP3M1_MC	47 kDa	0.997625	2	100%	Q9JKC8	Ap3m1	AP-3 comp	20.76077	27.76044	21.21283	27.62662	20.90359	27.16517	-6.55835	-7.27387	-5.84282	24.23824	-21.8157	7.658529	1.55E-07	0.000211	-1	-1
27	sp|Q9Z1G4	VPP1_MOU	96 kDa	0.999864	25	92%	Q9Z1G4	Atp6v0a1	V-type prot	24.21201	30.41103	24.84352	30.34598	24.00178	30.14157	-5.94709	-6.70844	-5.18574	27.32598	-18.5917	6.892845	4.53E-07	0.000237	-1	-1
146	sp|P51863	VA0D1_MC	40 kDa	0.999576	12	100%	P51863	Atp6v0d1	V-type prot	23.90194	29.86094	24.62866	29.91128	24.0949	29.59593	-5.58089	-6.31142	-4.85036	26.99894	-18.183	6.779771	5.25E-07	0.000237	-1	-1
815	sp|P97873	LOXL1_MO	67 kDa	0.999425	3	100%	P97873	Loxl1	Lysyl oxida	19.32217	24.25602	18.69365	23.90748	19.21276	23.77751	-4.90414	-5.63738	-4.1709	21.52826	-15.9191	6.072755	1.27E-06	0.000425	-1	-1
150	sp|Q6IRU5	CLCB_MOU	25 kDa	0.999464	12	92%	Q6IRU5	Cltb	Clathrin lig	28.38085	23.80329	28.405	24.14557	28.20823	23.45666	4.529515	3.830334	5.228696	26.0666	15.41923	5.895842	1.57E-06	0.000425	1	1
965	sp|Q9ET30	TM9S3_MC	68 kDa	0.997681	3	100%	Q9ET30	Tm9sf3	Transmemb	21.73077	25.95239	21.51526	25.49191	21.92613	25.78442	-4.01885	-4.69071	-3.34699	23.73348	-14.2371	5.442526	2.65E-06	0.000427	-1	-1
1269	sp|Q8CHP0	ZC3H3_MO	103 kDa	0.996205	2	100%	Q8CHP0	Zc3h3	Zinc finger	17.30309	21.83121	16.87701	21.70069	16.17289	21.75948	-4.97947	-5.82331	-4.13562	19.27406	-14.0552	5.341256	2.98E-06	0.000427	-1	-1
346	sp|P70662	LDB1_MOU	47 kDa	0.999359	7	86%	P70662	Ldb1	LIM domair	26.67237	21.65568	26.89319	21.90304	26.29582	22.43223	4.62348	3.83559	5.41137	24.30872	13.96704	5.331501	3.01E-06	0.000427	1	1
778	sp|P01811	HVM41_M	13 kDa	0.995278	4	25%	P01811	NA	Ig heavy ch	21.20257	14.84614	21.92793	15.22002	20.71727	15.79102	5.996865	4.988373	7.005358	18.28416	14.19065	5.325797	3.03E-06	0.000427	1	1
25	sp|O35245	PKD2_MOU	109 kDa	0.999878	26	100%	O35245	Pkd2	Polycystin-	26.92774	23.18959	26.6901	22.84655	26.98119	22.60046	3.98748	3.303276	4.671684	24.8726	13.87119	5.291385	3.15E-06	0.000427	1	1
1015.2	tr|E9Q035	E9Q035_M	108 kDa	0.99986	2	100%	E9Q035	Gm20425	Predicted g	24.19942	18.47307	24.71601	18.60751	23.98793	19.52478	5.432666	4.499976	6.365356	21.58479	13.90037	5.207377	3.46E-06	0.000427	1	1
821	sp|P80315	TCPD_MOU	58 kDa	0.999397	3	67%	P80315	Cct4	T-complex	22.14219	26.46881	22.63716	25.86335	22.41899	26.38492	-3.83958	-4.56915	-3.11001	24.31923	-12.5262	4.68612	6.15E-06	0.000695	-1	-1
89	sp|P60879	SNP25_MO	23 kDa	0.999799	16	94%	P60879	Snap25	Synaptosor	28.13807	22.63727	27.60588	23.42141	27.93553	23.73367	4.629044	3.744928	5.513159	25.57864	12.48178	4.611365	6.68E-06	0.000697	1	1
131	sp|Q61474	MSI1H_MO	39 kDa	0.999636	13	92%	Q61474	Msi1	RNA-bindir	28.00456	24.28889	27.83918	23.26068	27.77549	23.72374	4.115307	3.306103	4.924512	25.81542	12.10503	4.477346	7.7E-06	0.000746	1	1
222	sp|O09044	SNP23_MO	23 kDa	0.999882	9	89%	O09044	Snap23	Synaptosor	24.79802	21.10309	24.14536	20.64175	24.43624	21.11742	3.505791	2.767889	4.243692	22.70698	11.30807	4.061053	1.2E-05	0.000976	1	1
817	tr|A0A0B4.	A0A0B4J1N	11 kDa	0.999419	3	33%	A0A0B4J1N	Ighv1-77	Immunoglo	19.53749	16.75791	19.94923	16.80292	19.96815	16.76371	3.043447	2.402736	3.684159	18.29657	11.30587	4.059855	1.2E-05	0.000976	1	1
1184	sp|Q8K386	RAB15_MO	24 kDa	0.998107	2	50%	Q8K386	Rab15	Ras-related	18.95745	15.91514	19.35923	16.2916	19.33877	16.0425	3.135401	2.470756	3.800046	17.65078	11.22805	4.017113	1.25E-05	0.000976	1	1
1	sp|Q68FD5	CLH1_MOU	192 kDa	0.999927	86	100%	Q68FD5	Cltc	Clathrin he	33.45827	28.44516	32.44402	27.97304	33.37165	28.92852	4.642404	3.66433	5.620478	30.77011	11.32718	3.990771	1.3E-05	0.000976	1	1
35	sp|Q6NZC7	S23IP_MOU	111 kDa	0.999696	23	100%	Q6NZC7	Sec23ip	SEC23-inte	28.70469	24.29238	27.76828	24.26505	28.49033	24.75437	3.883829	3.049447	4.718211	26.37918	11.07943	3.933021	1.37E-05	0.000976	1	1
465	sp|O08585	CLCA_MOU	26 kDa	0.998487	6	83%	O08585	Clta	Clathrin lig	26.99857	21.91196	26.85232	23.2787	26.83168	22.39684	4.365024	3.419942	5.310106	24.71168	11.02218	3.823632	1.54E-05	0.001047	1	1
673	sp|P53395	ODB2_MOU	53 kDa	0.999094	4	100%	P53395	Dbt	Lipoamide	17.56244	21.5222	18.35403	21.60027	17.99298	21.1373	-3.45011	-4.21641	-2.6838	19.69487	-10.716	3.726752	1.69E-05	0.001058	-1	-1
305	sp|Q60749	KHDR1_MC	48 kDa	0.999885	7	86%	Q60749	Khdrbs1	KH domain	26.58346	21.36758	25.47485	20.85161	27.02601	20.11857	5.582188	4.353376	6.811	23.57035	10.84101	3.721701	1.72E-05	0.001058	1	1
877	sp|P46412	GPX3_MOU	25 kDa	0.998944	3	100%	P46412	Gpx3	Glutathion	18.0483	24.1013	19.86396	23.92534	18.39102	24.25832	-5.32723	-6.52138	-4.13308	21.43137	-10.6462	3.609801	1.93E-05	0.001124	-1	-1
273	sp|O35737	HNRH1_MC	49 kDa	0.999513	8	38%	O35737	Hnrnph1	Heterogene	23.74254	26.19736	23.47067	26.27192	23.46819	26.46083	-2.74957	-3.37576	-2.12338	24.93525	-10.451	3.570037	1.99E-05	0.001124	-1	-1
1061	sp|O09108	LSHB_MOU	15 kDa	0.999248	2	100%	O09108	Lhb	Lutropin su	19.02823	NA	18.45906	13.25611	19.67979	13.71208	5.571598	4.430488	6.712707	16.82705	12.10273	3.290413	2.8E-05	0.001518	1	1
329	sp|P06879	PRL_MOUS	25 kDa	0.999529	7	100%	P06879	Prl	Prolactin	30.24519	26.1948	30.41172	26.66332	29.35326	26.185	3.655683	2.766844	4.544523	28.17555	9.804813	3.125505	3.15E-05	0.001631	1	1
593	tr|A0A075	A0A075B5	13 kDa	0.999779	4	50%	A0A075B5	Ighv5-15	Immunoglo	20.77639	23.62668	20.93077	23.51604	20.3823	23.2847	-2.77932	-3.46261	-2.09603	22.08615	-9.68131	3.08767	3.25E-05	0.001631	-1	-1
18	sp|P63017	HSP7C_MO	71 kDa	0.999881	31	65%	P63017	Hspa8	Heat shock	27.85382	24.30508	27.7775	24.49842	27.83724	25.23323	3.143945	2.361162	3.926729	26.25088	9.559479	3.007402	3.52E-05	0.001704	1	1
266	sp|Q8BMF4	ODP2_MOU	68 kDa	0.999605	8	100%	Q8BMF4	Dlat	Dihydrolip	22.12227	25.28007	22.79759	25.53783	22.747	25.6216	-2.92421	-3.65684	-2.19158	24.01772	-9.50006	2.967848	3.66E-05	0.001704	-1	-1
611	sp|Q9CYZ8	SSBP2_MO	38 kDa	0.999605	4	75%	Q9CYZ8	Ssbp2	Single-strar	23.22381	17.48517	23.58941	19.2692	22.92989	18.70953	4.759738	3.573528	5.945949	20.86784	9.575731	2.949857	3.77E-05	0.001704	1	1
415	sp|P42669	PURA_MOU	35 kDa	0.999562	6	100%	P42669	Pura	Transcripti	27.2554	23.15779	26.17552	23.10798	27.42142	23.38845	3.732709	2.792645	4.672772	25.08443	9.475831	2.884084	4.03E-05	0.001752	1	1
45	sp|P28740	KIF2A_MOU	80 kDa	0.999855	21	100%	P28740	Kif2a	Kinesin-like	27.45602	23.07958	26.23326	23.07752	27.59822	23.29447	3.94531	2.947576	4.943043	25.12318	9.436634	2.85807	4.13E-05	0.001752	1	1
401	sp|Q9CX30	YIF1B_MOU	34 kDa	0.999669	6	100%	Q9CX30	Yif1b	Protein YIF	25.40531	22.23832	25.71674	23.02212	25.38173	22.52984	2.904495	2.157756	3.651234	24.04901	9.257689	2.803678	4.32E-05	0.001774	1	1
185	sp|Q7TSH6	SCAF4_MO	129 kDa	0.999718	10	70%	Q7TSH6	Scaf4	SR-related	26.36227	22.25709	25.69055	23.08979	26.06716	21.89685	3.625415	2.680733	4.570096	24.22728	9.158468	2.670024	4.99E-05	0.001989	1	1
336	sp|Q8VCM7	FIBG_MOU	49 kDa	0.999459	7	100%	Q8VCM7	Fgg	Fibrinogen	24.21079	21.07079	23.39936	21.09125	23.81612	20.64447	2.873253	2.09987	3.646637	22.37213	8.842596	2.511556	5.77E-05	0.002206	1	1
869	sp|Q61543	GSLG1_MO	134 kDa	0.998993	3	100%	Q61543	Glg1	Golgi appar	22.33651	19.98973	22.35442	19.45564	22.00334	19.49077	2.58604	1.888317	3.283763	20.9384	8.821714	2.496481	5.86E-05	0.002206	1	1
960	sp|Q8CC88	VWA8_MO	213 kDa	0.997749	3	100%	Q8CC88	Vwa8	von Willeb	19.3021	21.82637	18.8084	22.5329	19.51113	22.76163	-3.16642	-4.03557	-2.29728	20.79042	-8.68502	2.359742	6.75E-05	0.002473	-1	-1
725	tr|A0A075	A0A075B5I	12 kDa	0.998596	4	100%	A0A075B5I	Igkv12-38	Immunoglo	22.06952	25.55634	21.10743	25.18654	22.49077	25.58051	-3.55189	-4.53411	-2.56966	23.66519	-8.62976	2.295263	7.22E-05	0.002578	-1	-1
32	sp|Q6PDG5	SMRC2_MC	133 kDa	0.999852	24	67%	Q6PDG5	Smarcc2	SWI/SNF co	25.9951	22.71468	25.24442	22.74158	26.21342	23.12373	2.957648	2.127008	3.788287	24.33882	8.47531	2.239788	7.54E-05	0.002622	1	1
317	sp|Q8BP99	CA216_MO	25 kDa	0.999646	7	100%	Q8BP99	NA	UPF0500 p	22.22913	17.70089	22.15634	19.02669	22.08825	19.02444	3.573903	2.569788	4.578018	20.37096	8.493954	2.195138	7.97E-05	0.002702	1	1
10	sp|Q9EPU0	RENT1_MO	124 kDa	0.999794	40	100%	Q9EPU0	Upf1	Regulator c	26.88991	24.32191	26.69588	24.45162	26.57692	24.69033	2.232949	1.592188	2.873711	25.60443	8.294372	2.10313	8.62E-05	0.002851	1	1
760	sp|Q8R3N6	THOC1_MC	75 kDa	0.997356	4	100%	Q8R3N6	Thoc1	THO compl	21.25154	18.81515	20.96518	18.36662	21.37676	19.01436	2.465782	1.747415	3.184149	19.96494	8.169757	2.006487	9.47E-05	0.003058	1	1
171	sp|Q99KN9	EPN4_MOU	69 kDa	0.999326	11	100%	Q99KN9	Clint1	Clathrin int	24.55638	21.62096	24.17584	21.75137	23.7416	21.67437	2.47571	1.745107	3.206314	22.92009	8.065269	1.924321	0.000103	0.003176	1	1
614	sp|O70439	STX7_MOU	30 kDa	0.999563	4	100%	O70439	Stx7	Syntaxin-7	24.47173	21.79934	24.09237	22.1601	24.28612	21.40442	2.495454	1.75852	3.232387	23.03568	8.059757	1.919958	0.000103	0.003176	1	1
700	sp|P01672	KV3AK_MO	12 kDa	0.998891	4	25%	P01672	NA	Ig kappa ch	17.85217	25.33286	16.11599	23.82975	19.55036	25.45224	-7.03211	-9.17916	-4.88507	21.35556	-7.81619	1.670113	0.000133	0.003972	-1	-1
672	sp|P62814	VATB2_MO	57 kDa	0.999109	4	100%	P62814	Atp6v1b2	V-type prot	21.42942	23.58816	21.8876	23.90719	21.62955	23.90907	-2.15262	-2.81644	-1.4888	22.72516	-7.71826	1.643795	0.000135	0.003972	-1	-1
842	sp|P35278	RAB5C_MO	23 kDa	0.999231	3	33%	P35278	Rab5c	Ras-related	23.52915	25.74374	23.58007	25.43355	23.42921	25.40566	-2.01484	-2.63907	-1.39061	24.52023	-7.68242	1.614133	0.000139	0.004	-1	-1
1006	sp|Q8R1F6	HID1_MOU	89 kDa	0.995228	3	100%	Q8R1F6	Hid1	Protein HID	21.51472	18.28828	21.00575	17.14254	22.13313	16.37584	4.282311	2.959029	5.605593	19.41004	7.722831	1.594312	0.000143	0.004042	1	1
1020	sp|Q91VK4	ITM2C_MO	30 kDa	0.99973	2	100%	Q91VK4	Itm2c	Integral me	22.43251	24.79777	22.86348	25.01832	22.11566	24.62815	-2.3442	-3.08138	-1.60702	23.64265	-7.56874	1.519172	0.000152	0.004094	-1	-1
1021	tr|A0A075	A0A075B5	13 kDa	0.999721	2	50%	A0A075B5	Ighv8-11	Immunoglo	22.43251	24.79777	22.86348	25.01832	22.11566	24.62815	-2.3442	-3.08138	-1.60702	23.64265	-7.56874	1.519172	0.000152	0.004094	-1	-1
487	sp|P29699	FETUA_MO	37 kDa	0.99975	5	100%	P29699	Ahsg	Alpha-2-HS	25.11261	22.42983	25.37017	22.68068	24.46618	22.30354	2.511637	1.720119	3.303154	23.72717	7.552614	1.505597	0.000154	0.004094	1	1
16	sp|P14873	MAP1B_MC	270 kDa	0.999829	33	100%	P14873	Map1b	Microtubul	23.45623	25.67341	23.8141	25.9599	23.4985	25.48232	-2.1156	-2.78703	-1.44416	24.64741	-7.49944	1.460631	0.000161	0.004193	-1	-1
149	sp|P46460	NSF_MOUS	83 kDa	0.999474	12	100%	P46460	Nsf	Vesicle-fusi	23.79325	25.98677	24.10546	25.83201	24.08428	26.0159	-1.95057	-2.57399	-1.32714	24.96961	-7.44693	1.415927	0.000168	0.004295	-1	-1
1167	sp|P11438	LAMP1_MC	44 kDa	0.998266	2	100%	P11438	Lamp1	Lysosome-a	19.02603	22.54742	18.41984	21.2358	19.0289	21.83544	-3.04796	-4.03173	-2.0642	20.3489	-7.39381	1.320057	0.000186	0.004586	-1	-1
1034	tr|A0A075	A0A075B6	10 kDa	0.999481	2	100%	A0A075B6	Igkv4-53	Immunoglo	22.30582	18.99434	21.35155	19.49426	22.41964	18.67668	2.970574	2.010291	3.930856	20.54038	7.382304	1.310256	0.000188	0.004586	1	1
202	sp|Q61235	SNTB2_MO	56 kDa	0.99945	10	90%	Q61235	Sntb2	Beta-2-synt	23.90047	20.00991	23.21943	18.6166	23.04242	20.27871	3.752367	2.538014	4.966719	21.51126	7.374136	1.303293	0.000189	0.004586	1	1
240	sp|Q9WTY1	PDCD7_MC	54 kDa	0.999289	9	100%	Q9WTY1	Pdcd7	Programme	24.95165	20.8272	23.6166	21.2312	25.04715	21.50362	3.351132	2.257518	4.444745	22.8629	7.312712	1.250699	0.000199	0.004622	1	1
932	sp|Q6P5F9	XPO1_MOU	123 kDa	0.998194	3	100%	Q6P5F9	Xpo1	Exportin-1	24.83588	26.61359	24.54679	26.46385	24.7107	26.59012	-1.85806	-2.47004	-1.24609	25.62682	-7.22649	1.225039	0.000202	0.004622	-1	-1
113	tr|Q3UJB0	Q3UJB0_M	98 kDa	0.999862	14	100%	Q3UJB0	Sf3b2	Splicing fac	24.56331	21.89792	23.82033	22.09233	24.76927	21.53842	2.541412	1.698843	3.383981	23.1136	7.184315	1.174536	0.000212	0.004622	1	1
895	tr|A0A075	A0A075B5	13 kDa	0.998746	3	33%	A0A075B5	Ighv1-54	Immunoglo	19.32216	22.55592	20.29939	22.08272	19.97117	22.64287	-2.56293	-3.41249	-1.71337	21.1457	-7.19014	1.167838	0.000214	0.004622	-1	-1
867.1	tr|A0A075	A0A075B5I	13 kDa	0.999017	3	100%	A0A075B5I	Igkv6-29	Immunoglo	20.24114	23.03438	20.98652	23.70762	19.87035	22.95128	-2.86509	-3.81424	-1.91594	21.79855	-7.2037	1.156352	0.000218	0.004622	-1	-1
967	sp|Q99LX0	PARK7_MO	20 kDa	0.997612	3	100%	Q99LX0	Park7	Protein/nu	19.73811	17.72553	20.29779	17.60711	19.48512	17.55125	2.212375	1.473917	2.950833	18.73415	7.130727	1.140447	0.000219	0.004622	1	1
128	sp|Q8CJG0	AGO2_MOU	97 kDa	0.999715	13	85%	Q8CJG0	Ago2	Protein arg	23.84731	21.8418	23.43332	21.78657	23.93438	21.52956	2.019025	1.344763	2.693288	22.72882	7.127113	1.137234	0.000219	0.004622	1	1
224	sp|Q3UH66	WNK2_MO	228 kDa	0.999818	9	89%	Q3UH66	Wnk2	Serine/thre	21.59838	18.0727	21.81789	19.49968	21.87457	18.92093	2.932511	1.959063	3.90596	20.29736	7.189146	1.143654	0.000221	0.004622	1	1
1135	sp|Q61771	KIF3B_MOU	85 kDa	0.998572	2	100%	Q61771	Kif3b	Kinesin-like	20.20314	22.59185	19.91895	21.91601	19.55877	22.09827	-2.30842	-3.08057	-1.53628	21.04783	-7.11573	1.127105	0.000222	0.004622	-1	-1
51	sp|Q8BZ98	DYN3_MOU	97 kDa	0.999347	20	65%	Q8BZ98	Dnm3	Dynamin-3	25.00528	21.075	23.35635	19.66307	25.03548	20.22352	4.145173	2.745449	5.544897	22.39312	7.067259	1.03641	0.000245	0.004931	1	1
257	sp|Q8VDM4	PSMD2_MC	100 kDa	0.999764	8	100%	Q8VDM4	Psmd2	26S protea	22.18327	24.19029	22.36904	24.03655	22.13198	23.91414	-1.81889	-2.43739	-1.2004	23.13754	-6.99963	1.022977	0.000245	0.004931	-1	-1
47	sp|Q9QX47	SON_MOUS	266 kDa	0.999742	20	100%	Q9QX47	Son	Protein SON	23.98495	21.62267	23.9458	21.34901	24.5157	22.22077	2.417995	1.5945	3.24149	22.93982	6.989019	1.012516	0.000247	0.004931	1	1
1122	tr|B2RXQ2	B2RXQ2_M	143 kDa	0.998694	2	100%	B2RXQ2	Ppfia1	Ppfia1 prot	20.92404	19.22578	20.93676	18.99896	20.78955	18.725	1.900201	1.251107	2.549294	19.93335	6.967754	0.994115	0.000252	0.004945	1	1
1201	sp|Q9D883	U2AF1_MO	28 kDa	0.997941	2	100%	Q9D883	U2af1	Splicing fac	13.95881	20.42201	13.85657	18.76473	15.7423	18.70523	-4.7781	-6.41853	-3.13767	16.90828	-6.95101	0.932566	0.00027	0.005213	-1	-1
133	sp|Q8C3F2	F120C_MO	120 kDa	0.999553	13	85%	Q8C3F2	Fam120c	Constitutiv	24.45541	21.95124	24.25257	21.71945	23.83938	22.35075	2.175304	1.422157	2.928451	23.0948	6.874494	0.909007	0.000273	0.005213	1	1
1200	sp|O35226	PSMD4_M	41 kDa	0.997946	2	100%	O35226	Psmd4	26S protea	19.63834	18.04042	19.6948	17.72898	19.696	17.97699	1.760916	1.148333	2.373499	18.79592	6.841865	0.87899	0.000281	0.00529	1	1
1001	sp|Q9DC51	GNAI3_MO	41 kDa	0.995483	3	33%	Q9DC51	Gnai3	Guanine nu	20.16763	22.66421	21.0293	22.78926	20.4766	22.65797	-2.14597	-2.8958	-1.39614	21.63083	-6.81184	0.851257	0.000288	0.005357	-1	-1
660	sp|P56395	CYB5_MOU	15 kDa	0.999229	4	100%	P56395	Cyb5a	Cytochrom	21.33571	25.39062	22.28798	24.08911	21.39577	24.7135	-3.05792	-4.1267	-1.98914	23.20212	-6.82791	0.820907	0.000301	0.005507	-1	-1
488	tr|A0A0A6	A0A0A6YYE	13 kDa	0.999748	5	80%	A0A0A6YYE	Igkv4-57	Immunoglo	22.71461	25.18804	23.24824	25.04631	22.70693	24.64219	-2.06892	-2.80411	-1.33374	23.92439	-6.69807	0.745204	0.000319	0.00576	-1	-1
158	sp|P27659	RL3_MOUS	46 kDa	0.999783	11	100%	P27659	Rpl3	60S riboson	25.38364	23.54433	25.15199	23.32146	25.12776	23.56829	1.7431	1.121216	2.364985	24.34958	6.671347	0.720072	0.000327	0.00576	1	1
505	sp|Q9CQF3	CPSF5_MO	26 kDa	0.999523	5	100%	Q9CQF3	Nudt21	Cleavage ar	23.55598	21.18985	22.98271	21.12811	23.0107	21.40734	1.94136	1.245526	2.637194	22.21245	6.640507	0.690958	0.000336	0.00576	1	1
209	tr|Q6PHQ9	Q6PHQ9_M	72 kDa	0.999289	10	80%	Q6PHQ9	Pabpc4	Polyadenyl	22.35413	19.69856	21.93965	19.30669	21.99438	20.32371	2.319733	1.48701	3.152457	20.93619	6.630695	0.680866	0.000339	0.00576	1	1
162	sp|P47911	RL6_MOUS	34 kDa	0.999527	11	100%	P47911	Rpl6	60S riboson	25.64704	24.0894	25.82642	24.06906	25.67027	23.73458	1.750233	1.118812	2.381655	24.83946	6.597471	0.650136	0.000349	0.00576	1	1
72	sp|Q9D8E6	RL4_MOUS	47 kDa	0.999669	17	100%	Q9D8E6	Rpl4	60S riboson	25.47186	23.96585	25.47296	23.57612	25.53497	23.76307	1.724916	1.102302	2.347531	24.63081	6.594014	0.646847	0.00035	0.00576	1	1
100.1	sp|P17879	HS71B_MO	70 kDa	0.999699	15	73%	P17879	Hspa1b	Heat shock	24.99751	22.79349	24.59787	22.00965	24.82205	22.90713	2.23572	1.426672	3.044768	23.68795	6.577565	0.630384	0.000356	0.00576	1	1
100.2	sp|Q61696	HS71A_MO	70 kDa	0.999699	15	73%	Q61696	Hspa1a	Heat shock	24.99751	22.79349	24.59787	22.00965	24.82205	22.90713	2.23572	1.426672	3.044768	23.68795	6.577565	0.630384	0.000356	0.00576	1	1
474	sp|P06330	HVM51_M	13 kDa	0.997551	6	33%	P06330	NA	Ig heavy ch	22.48901	24.47297	22.66889	24.13055	22.60441	24.30527	-1.71549	-2.3366	-1.09438	23.44518	-6.57383	0.627613	0.000357	0.00576	-1	-1
534	sp|Q91YN9	BAG2_MOU	23 kDa	0.999217	5	100%	Q91YN9	Bag2	BAG family	22.41719	20.56972	22.06817	20.48345	22.55459	19.98094	2.001945	1.276328	2.727562	21.34568	6.566671	0.620783	0.000359	0.00576	1	1
390	sp|Q02105	C1QC_MOU	26 kDa	0.999859	6	100%	Q02105	C1qc	Compleme	25.20597	27.0774	25.37386	27.64586	25.31825	26.98727	-1.93749	-2.64036	-1.23461	26.2681	-6.56089	0.615261	0.000361	0.00576	-1	-1
1207	sp|Q9CR67	TMM33_M	28 kDa	0.997827	2	100%	Q9CR67	Tmem33	Transmemb	19.33726	20.98385	18.95207	21.01609	18.36109	21.07694	-2.14216	-2.92678	-1.35754	19.95455	-6.49818	0.555091	0.000382	0.006028	-1	-1
603	sp|Q91V92	ACLY_MOU	120 kDa	0.999696	4	100%	Q91V92	Acly	ATP-citrate	19.25311	21.09801	18.97263	21.2785	19.51354	20.91574	-1.85099	-2.53071	-1.17127	20.17192	-6.48145	0.538951	0.000388	0.006051	-1	-1
888	sp|Q9CYK2	QPCT_MOU	41 kDa	0.998803	3	100%	Q9CYK2	Qpct	Glutaminyl	22.55832	20.3088	22.12631	20.06781	21.77316	20.14065	1.98018	1.250027	2.710333	21.16251	6.454927	0.513303	0.000398	0.006063	1	1
201	sp|P12970	RL7A_MOU	30 kDa	0.999456	10	100%	P12970	Rpl7a	60S riboson	24.97129	23.25296	25.23147	23.27719	24.75899	23.24403	1.729186	1.091552	2.36682	24.12266	6.454625	0.51301	0.000398	0.006063	1	1
325	sp|P46062	SIPA1_MOU	112 kDa	0.999569	7	100%	P46062	Sipa1	Signal-indu	22.8695	19.37953	21.36005	19.55026	22.09384	19.09797	2.765208	1.743169	3.787247	20.72519	6.456705	0.473304	0.000418	0.006259	1	1
12	sp|Q8CGB6	TNS2_MOU	152 kDa	0.999844	38	97%	Q8CGB6	Tns2	Tensin-2	27.48814	25.23302	27.2899	24.28074	27.30562	25.42792	2.380662	1.497997	3.263327	26.17089	6.429779	0.463807	0.00042	0.006259	1	1
620	sp|Q9CR57	RL14_MOU	24 kDa	0.999496	4	100%	Q9CR57	Rpl14	60S riboson	25.19446	23.55456	25.51297	23.76991	25.40258	23.79248	1.664354	1.038076	2.290632	24.53783	6.325271	0.386636	0.000449	0.006612	1	1
313	sp|Q9CR62	M2OM_MC	34 kDa	0.999669	7	100%	Q9CR62	Slc25a11	Mitochond	23.14452	24.76875	23.08397	25.90765	22.56156	25.32734	-2.40457	-3.3112	-1.49793	24.1323	-6.32262	0.359554	0.000464	0.006761	-1	-1
863	sp|P60867	RS20_MOU	13 kDa	0.99904	3	100%	P60867	Rps20	40S riboson	22.76709	20.86503	23.24579	21.0859	22.65141	21.19691	1.838817	1.141489	2.536146	21.96869	6.276274	0.338213	0.00047	0.006775	1	1
442	sp|P21981	TGM2_MOU	77 kDa	0.999192	6	100%	P21981	Tgm2	Protein-glu	23.24223	24.71714	23.06424	24.9141	23.33857	24.83918	-1.60846	-2.22203	-0.9949	24.01924	-6.23955	0.301716	0.000486	0.006871	-1	-1
762	sp|Q8CJG1	AGO1_MOU	97 kDa	0.997303	4	50%	Q8CJG1	Ago1	Protein arg	25.07083	26.84661	25.39802	27.50542	25.40617	27.12042	-1.86581	-2.57759	-1.15402	26.22458	-6.23905	0.301218	0.000486	0.006871	-1	-1
121	sp|O70133	DHX9_MOU	149 kDa	0.999414	14	100%	O70133	Dhx9	ATP-depen	24.84958	22.61174	24.1873	22.58325	24.62655	22.89707	1.857126	1.147273	2.566979	23.62592	6.226926	0.289132	0.000492	0.006878	1	1
227	sp|Q9QZM0	UBQL2_MC	67 kDa	0.999751	9	78%	Q9QZM0	Ubqln2	Ubiquilin-2	25.1567	22.82399	26.28683	22.33402	24.68113	22.3235	2.88105	1.784037	3.978064	23.93436	6.267431	0.289529	0.000498	0.006888	1	1
164	sp|Q920Q6	MSI2H_MO	37 kDa	0.999498	11	91%	Q920Q6	Msi2	RNA-bindir	30.20477	26.58534	29.58572	24.87125	30.10317	27.15205	3.761676	2.325269	5.198084	28.08372	6.249632	0.272013	0.000506	0.006933	1	1
137	sp|Q3UZ01	RNPC3_MC	58 kDa	0.999248	13	100%	Q3UZ01	Rnpc3	RNA-bindir	25.61458	20.83318	24.21064	22.35387	25.69598	21.84229	3.497288	2.152342	4.842235	23.42509	6.205503	0.22841	0.000527	0.007135	1	1
934	sp|Q3U9G9	LBR_MOUS	71 kDa	0.998165	3	100%	Q3U9G9	Lbr	Delta(14)-s	19.24133	23.56489	20.08595	21.79438	19.18778	22.75413	-3.19945	-4.43145	-1.96745	21.10474	-6.19747	0.220444	0.000531	0.007135	-1	-1
1033.1	sp|O08583	THOC4_MC	27 kDa	0.999499	2	100%	O08583	Alyref	THO compl	21.36412	24.28091	20.31773	25.11617	18.88521	23.73803	-4.18935	-5.81467	-2.56403	22.2837	-6.15116	0.174372	0.000555	0.007302	-1	-1
253	sp|O54774	AP3D1_MC	135 kDa	0.997664	9	100%	O54774	Ap3d1	AP-3 comp	18.61988	20.98259	18.38482	20.68194	18.8474	20.1498	-1.98741	-2.76301	-1.21182	19.61107	-6.09892	0.160342	0.000556	0.007302	-1	-1
180	sp|Q80U40	RIMB2_MO	118 kDa	0.998865	11	100%	Q80U40	Rimbp2	RIMS-bindi	22.50782	19.61629	22.41039	18.36859	23.01815	20.31138	3.213367	1.9648	4.461934	21.03877	6.14185	0.165072	0.00056	0.007302	1	1
119	sp|Q8VEK3	HNRPU_M	88 kDa	0.999549	14	100%	Q8VEK3	Hnrnpu	Heterogene	26.3786	24.6574	26.38156	24.48236	26.37727	25.01479	1.660958	1.008343	2.313572	25.54866	6.057632	0.118341	0.000578	0.007469	1	1
825	tr|A0A075	A0A075B5	11 kDa	0.999375	3	33%	A0A075B5	Ighv1-56	Immunoglo	23.47433	20.17281	22.34031	20.90487	23.77537	20.27581	2.745506	1.665334	3.825678	21.82392	6.065691	0.088595	0.000602	0.00764	1	1
511	sp|Q3TWN	CNNM2_M	97 kDa	0.999457	5	100%	Q3TWN3	Cnnm2	Metal trans	20.05472	17.92137	19.41725	17.7696	20.64281	17.37158	2.350741	1.425285	3.276196	18.86289	6.060599	0.086212	0.000603	0.00764	1	1
1041	sp|Q8VHH5	AGAP3_MC	98 kDa	0.999401	2	100%	Q8VHH5	Agap3	Arf-GAP wi	21.07145	23.06676	21.07973	23.7278	21.0453	22.63263	-2.0769	-2.90335	-1.25046	22.10394	-5.98172	0.039839	0.000623	0.007788	-1	-1
1040	sp|P62267	RS23_MOU	16 kDa	0.99942	2	100%	P62267	Rps23	40S riboson	24.25166	22.62279	24.47838	22.71259	24.08159	22.75469	1.573851	0.946897	2.200804	23.48362	5.974877	0.033478	0.000627	0.007788	1	1
199	sp|Q7TNC4	LC7L2_MO	47 kDa	0.999471	10	100%	Q7TNC4	Luc7l2	Putative RN	23.05372	24.66801	22.94288	24.84157	23.44575	24.92716	-1.6648	-2.32891	-1.00068	23.97985	-5.9665	0.024836	0.000632	0.007788	-1	-1
203	sp|O08917	FLOT1_MO	48 kDa	0.9994	10	100%	O08917	Flot1	Flotillin-1	21.59521	23.52845	21.92339	24.57669	22.24405	24.3758	-2.23943	-3.13842	-1.34045	23.0406	-5.93855	-0.02629	0.000668	0.008158	-1	-1
331	sp|Q99KP6	PRP19_MO	55 kDa	0.999488	7	100%	Q99KP6	Prpf19	Pre-mRNA-	24.82743	22.70567	24.27015	23.05885	25.17441	22.69926	1.93607	1.146878	2.725263	23.78929	5.839006	-0.10786	0.000716	0.008616	1	1
485	sp|Q9D0L4	ADCK1_MC	60 kDa	0.999797	5	100%	Q9D0L4	Adck1	Uncharacte	22.14353	19.38703	21.67331	19.53578	22.35148	20.44388	2.267211	1.346956	3.187467	20.9225	5.877701	-0.09952	0.000718	0.008616	1	1
710	sp|P08226	APOE_MOU	36 kDa	0.998824	4	100%	P08226	Apoe	Apolipopro	21.16605	19.66038	21.29676	19.95859	21.53739	19.48436	1.632292	0.96341	2.301174	20.51726	5.808305	-0.14014	0.000738	0.008781	1	1
235	sp|Q9Z2N8	ACL6A_MO	47 kDa	0.999542	9	89%	Q9Z2N8	Actl6a	Actin-like p	24.21382	21.73671	23.18929	21.35708	24.51204	21.71926	2.367363	1.399225	3.335502	22.78803	5.8355	-0.14725	0.000752	0.008839	1	1
405	sp|P62754	RS6_MOUS	29 kDa	0.999619	6	100%	P62754	Rps6	40S riboson	25.81162	24.16112	26.02414	23.63338	25.38644	24.0423	1.795134	1.055764	2.534505	24.84316	5.778772	-0.17132	0.00076	0.008839	1	1
1086	sp|P39654	LOX15_MO	75 kDa	0.999073	2	100%	P39654	Alox15	Arachidona	24.30309	26.42581	24.06726	25.96152	24.83022	26.41315	-1.86664	-2.63589	-1.09739	25.33351	-5.77558	-0.1747	0.000763	0.008839	-1	-1
928	sp|P01831	THY1_MOU	18 kDa	0.998266	3	100%	P01831	Thy1	Thy-1 mem	18.01021	16.79774	18.45987	15.83689	18.24632	15.81	2.090589	1.221915	2.959262	17.19351	5.73728	-0.23635	0.000814	0.009355	1	1
281	sp|Q61103	REQU_MOU	44 kDa	0.999382	8	88%	Q61103	Dpf2	Zinc finger	24.10588	21.64723	23.5233	21.22568	24.4496	22.29356	2.304103	1.347004	3.261203	22.87421	5.74507	-0.24186	0.000822	0.009368	1	1
315	sp|Q8R081	HNRPL_MC	64 kDa	0.999659	7	100%	Q8R081	Hnrnpl	Heterogene	23.52547	21.8458	23.09379	21.48003	23.40808	21.87588	1.608544	0.935155	2.281933	22.53818	5.685484	-0.27059	0.000835	0.009433	1	1
535	sp|P50544	ACADV_MC	71 kDa	0.999215	5	100%	P50544	Acadvl	Very long-c	18.81377	20.64122	18.5118	20.44809	19.08922	21.41604	-2.03018	-2.88479	-1.17558	19.82002	-5.66193	-0.31351	0.000875	0.009789	-1	-1
170	sp|P48962	ADT1_MOU	33 kDa	0.999351	11	55%	P48962	Slc25a4	ADP/ATP tr	24.79143	23.2315	24.44439	22.75918	24.49906	23.01531	1.576296	0.909271	2.243321	23.79015	5.624665	-0.33597	0.000888	0.009789	1	1
437	sp|O55203	LDB2_MOU	43 kDa	0.999263	6	83%	O55203	Ldb2	LIM domair	23.60646	21.88231	23.91088	20.79993	23.29214	21.68058	2.148885	1.242273	3.055498	22.52872	5.650481	-0.32866	0.000888	0.009789	1	1
191	sp|Q3U0V1	FUBP2_MO	77 kDa	0.999648	10	90%	Q3U0V1	Khsrp	Far upstrea	24.72273	21.66067	24.14036	21.8274	23.23654	21.44241	2.389714	1.380986	3.398441	22.83835	5.653567	-0.33875	0.000901	0.009848	1	1
204	sp|Q60634	FLOT2_MO	47 kDa	0.999346	10	100%	Q60634	Flot2	Flotillin-2	21.85661	23.9754	21.5404	23.67719	22.37026	23.52969	-1.805	-2.57745	-1.03256	22.82493	-5.56175	-0.40414	0.000947	0.010239	-1	-1
1057	sp|A2AFS3	K1324_MO	111 kDa	0.999283	2	100%	A2AFS3	Kiaa1324	UPF0577 p	14.40482	16.87575	13.91451	16.51142	15.23356	16.78388	-2.20605	-3.14634	-1.26577	15.62066	-5.59896	-0.39713	0.000951	0.010239	-1	-1
489	sp|P47963	RL13_MOU	24 kDa	0.99974	5	100%	P47963	Rpl13	60S riboson	25.0958	23.73636	25.33176	23.65812	24.96917	23.67106	1.443731	0.824413	2.06305	24.41038	5.548471	-0.41861	0.00096	0.010246	1	1
562	sp|P84751	HVM63_M	25 kDa	0.998735	5	40%	P84751	NA	Ig heavy ch	17.50556	21.33888	17.75594	21.7733	16.89312	19.47553	-3.4777	-4.97845	-1.97695	19.12372	-5.53011	-0.47133	0.00102	0.01075	-1	-1

1140	sp|Q8K2T1	NMRL1_M	34 kDa	0.998537	2	100%	Q8K2T1	Nmral1	NmrA-like f	18.37238	20.24967	17.58994	19.46955	18.02624	20.04258	-1.92441	-2.75928	-1.08953	18.95839	-5.48652	-0.487	0.001024	0.01075	-1	-1
1164	sp|Q9JMD	ZN207_MO	53 kDa	0.998285	2	100%	Q9JMD0	Znf207	BUB3-inter	19.74728	21.67862	19.75813	21.20502	19.82974	21.09754	-1.54867	-2.22141	-0.87594	20.55272	-5.47921	-0.49445	0.001031	0.01075	-1	-1
553	sp|P62141	PP1B_MOU	37 kDa	0.998911	5	40%	P62141	Ppp1cb	Serine/thre	22.1122	19.15572	21.42328	20.32593	22.05817	19.44851	2.221164	1.258729	3.183599	20.75397	5.507569	-0.49578	0.001044	0.010776	1	1
491	sp|Q99LX5	MMTA2_M	29 kDa	0.999696	5	100%	Q99LX5	Mmtag2	Multiple m	22.0584	23.60252	22.02218	23.40948	21.98945	23.27442	-1.40546	-2.01824	-0.79269	22.72608	-5.4591	-0.51659	0.001052	0.010776	-1	-1
1261	sp|P51660	DHB4_MOU	79 kDa	0.996427	2	100%	P51660	Hsd17b4	Peroxisoma	20.02291	18.00302	19.22207	17.81911	20.25533	17.99475	1.894478	1.067929	2.721027	18.8862	5.455607	-0.52104	0.001057	0.010776	1	1
441	sp|P62717	RL18A_MO	21 kDa	0.999204	6	100%	P62717	Rpl18a	60S ribosor	23.75002	22.17157	23.98249	22.46415	23.73768	22.51228	1.440731	0.805961	2.0755	23.10303	5.402163	-0.57961	0.001117	0.011262	1	1
787	sp|P00920	CAH2_MOU	29 kDa	0.999666	3	100%	P00920	Ca2	Carbonic ar	22.45722	24.61984	23.44527	24.91472	22.92938	24.74197	-1.81489	-2.61509	-1.01468	23.8514	-5.39817	-0.58405	0.001121	0.011262	-1	-1
255	sp|P63038	CH60_MOU	61 kDa	0.99996	8	100%	P63038	Hspd1	60 kDa heat	24.20734	25.35854	24.24799	25.71248	23.98566	25.76242	-1.46415	-2.11229	-0.81602	24.87907	-5.37678	-0.60786	0.001147	0.011298	-1	-1
1227	sp|Q3UMR	MCU_MOU	40 kDa	0.997551	2	100%	Q3UMR5	Mcu	Calcium un	19.99982	22.60322	21.23592	22.51478	20.10491	22.5854	-2.12092	-3.05604	-1.1858	21.50734	-5.41262	-0.59952	0.001151	0.011298	-1	-1
916.8	sp|C0HKE8	H2A1O_MO	14 kDa	0.998436	3	33%	C0HKE8	Hist1h2ao	Histone H2	22.23442	23.69836	22.50782	23.97068	22.58551	24.18567	-1.50899	-2.17846	-0.83952	23.19708	-5.36487	-0.62115	0.001161	0.011298	-1	-1
1093.2	sp|Q9CQL1	MGN2_MO	17 kDa	0.999012	2	100%	Q9CQL1	Magohb	Protein ma	17.28198	20.77473	18.80031	21.09546	18.0593	20.05533	-2.59464	-3.74123	-1.44806	19.34452	-5.40035	-0.61302	0.001165	0.011298	-1	-1
57	sp|Q8CG14	CS1A_MOU	77 kDa	0.999695	19	53%	Q8CG14	C1sa	Compleme	25.23354	26.80708	25.39255	27.05831	24.99033	26.46789	-1.57228	-2.2704	-0.87417	25.99162	-5.36049	-0.62604	0.001166	0.011298	-1	-1
452.1	sp|P62270	RS18_MOU	18 kDa	0.999008	6	100%	P62270	Rps18	40S ribosor	24.85654	23.21343	24.95486	23.24909	24.33298	22.92725	1.584867	0.875634	2.294099	23.92236	5.318691	-0.67285	0.001219	0.011522	1	1
452.2	tr|A0A1Y7	A0A1Y7VKY	18 kDa	0.999008	6	100%	A0A1Y7VKY	Gm11361	MCG11667	24.85654	23.21343	24.95486	23.24909	24.33298	22.92725	1.584867	0.875634	2.294099	23.92236	5.318691	-0.67285	0.001219	0.011522	1	1
452.3	tr|F6YVP7|	F6YVP7_M	18 kDa	0.999008	6	100%	F6YVP7	Gm10260	Predicted g	24.85654	23.21343	24.95486	23.24909	24.33298	22.92725	1.584867	0.875634	2.294099	23.92236	5.318691	-0.67285	0.001219	0.011522	1	1
777	sp|Q62159	RHOC_MOU	22 kDa	0.99532	4	25%	Q62159	Rhoc	Rho-related	23.62226	22.13382	23.3445	21.15459	23.28133	21.55788	1.8006	0.994191	2.607008	22.51573	5.314763	-0.67783	0.001225	0.011522	1	1
1172	sp|Q9QXB	DRG2_MOU	41 kDa	0.998227	2	100%	Q9QXB9	Drg2	Developme	18.50962	NA	15.50291	25.30819	16.69878	NA	-8.40442	-11.5833	-5.22555	19.00488	-6.92992	-0.19366	0.001232	0.011522	-1	-1
655	sp|P14206	RSSA_MOU	33 kDa	0.999272	4	100%	P14206	Rpsa	40S ribosor	21.95529	23.99335	22.54462	23.8496	22.24228	23.57012	-1.55696	-2.25852	-0.8554	23.02587	-5.28218	-0.71396	0.001267	0.011767	-1	-1
109	sp|P62702	RS4X_MOU	30 kDa	0.999356	15	100%	P62702	Rps4x	40S ribosor	24.89577	23.38949	24.89357	23.57903	24.68421	23.46402	1.346999	0.738328	1.95567	24.15102	5.267271	-0.7308	0.001287	0.011873	1	1
546	sp|P98086	C1QA_MOU	26 kDa	0.999049	5	100%	P98086	C1qa	Compleme	25.07402	26.66377	25.41641	27.02354	24.78715	26.46682	-1.62552	-2.36253	-0.88851	25.90529	-5.24954	-0.75086	0.001312	0.012017	-1	-1
104	sp|Q6A068	CDC5L_MO	92 kDa	0.9996	15	27%	Q6A068	Cdc5l	Cell divisio	22.38783	20.79496	22.74659	20.95253	22.79606	21.42558	1.585804	0.865296	2.306312	21.85059	5.238554	-0.76332	0.001327	0.012077	1	1
75	sp|P16546	SPTN1_MO	285 kDa	0.999629	17	100%	P16546	Sptan1	Spectrin al	24.12955	25.25185	23.58235	25.19174	23.77182	25.49079	-1.48355	-2.16013	-0.80698	24.56969	-5.21903	-0.7855	0.001355	0.012249	-1	-1
176	sp|Q9Z204	HNRPC_M	34 kDa	0.999252	11	100%	Q9Z204	Hnrnpc	Heterogene	25.77075	23.35799	24.80237	23.26727	26.29288	23.58338	2.21912	1.21016	3.228079	24.51244	5.248771	-0.78161	0.001365	0.012261	1	1
87	sp|P61979	HNRPK_M	51 kDa	0.999832	16	100%	P61979	Hnrnpk	Heterogene	25.68902	24.44393	25.63567	24.37872	25.64861	24.2448	1.301951	0.705108	1.898794	25.00679	5.192008	-0.8163	0.001395	0.012336	1	1
7	sp|P70336	ROCK2_M	161 kDa	0.99975	49	100%	P70336	Rock2	Rho-associ	27.49656	24.87803	28.27345	24.56162	26.65873	25.46058	2.509501	1.362953	3.656049	26.22149	5.223309	-0.81026	0.001403	0.012336	1	1
386	sp|Q6NXH	K2C73_MO	59 kDa	0.997663	7	29%	Q6NXH9	Krt73	Keratin, typ	19.95852	21.63969	18.08747	21.57577	19.05922	22.74447	-2.95158	-4.30129	-1.60187	20.51086	-5.21873	-0.81543	0.001409	0.012336	-1	-1
574	sp|P35276	RAB3D_M	24 kDa	0.998133	5	20%	P35276	Rab3d	Ras-related	23.05199	21.57264	22.76164	21.55718	22.76453	21.38654	1.353929	0.73204	1.975819	22.18242	5.181839	-0.82792	0.00141	0.012336	1	1
482	sp|O35295	PURB_MOU	34 kDa	0.99993	5	100%	O35295	Purb	Transcripti	22.71308	20.3186	21.9463	20.82005	22.24894	20.75416	1.671833	0.901958	2.441707	21.46685	5.168606	-0.84306	0.00143	0.012407	1	1
541	sp|P18529	HVM58_M	13 kDa	0.999137	5	20%	P18529	NA	Ig heavy ch	20.90458	22.80126	21.44474	23.44875	20.59252	22.53165	-1.9466	-2.84138	-1.05183	21.95392	-5.1863	-0.84052	0.001437	0.012407	-1	-1
1133	sp|Q9ERU9	RBP2_MOU	341 kDa	0.998587	2	100%	Q9ERU9	Ranbp2	E3 SUMO-p	20.797	19.66775	21.43199	19.14767	20.93915	19.54311	1.603206	0.861802	2.34461	20.25445	5.146776	-0.8681	0.001464	0.012567	1	1
277	sp|P62281	RS11_MOU	18 kDa	0.999465	8	100%	P62281	Rps11	40S ribosor	24.70272	23.4546	24.58657	22.66491	24.48314	22.88109	1.590609	0.853852	2.327365	23.79551	5.138543	-0.87756	0.001477	0.012599	1	1
347	sp|P01837	IGKC_MOU	12 kDa	0.999358	7	100%	P01837	Igkc	Immunoglo	25.62897	27.77326	26.25782	28.12965	25.62317	27.19193	-1.86163	-2.72373	-0.99952	26.76746	-5.14784	-0.88434	0.001497	0.012686	-1	-1
685	sp|Q6ZWU	RS27_MOU	9 kDa	0.999015	4	50%	Q6ZWU9	Rps27	40S ribosor	21.56806	19.69359	21.59014	20.16681	21.07589	19.46714	1.63552	0.873956	2.397085	20.59361	5.111524	-0.90867	0.001521	0.012812	1	1
607	sp|P62751	RL23A_MO	18 kDa	0.999674	4	100%	P62751	Rpl23a	60S ribosor	24.08806	22.72058	24.21192	22.90146	24.01876	22.82157	1.291708	0.688635	1.89478	23.46039	5.097951	-0.92435	0.001544	0.012892	1	1
833	sp|P01029	CO4B_MOU	193 kDa	0.99931	3	100%	P01029	C4b	Compleme	25.37734	26.83135	24.74714	26.71139	25.56214	27.20751	-1.68787	-2.47646	-0.89929	26.07281	-5.0944	-0.92845	0.00155	0.012892	-1	-1
1142.1	sp|P47915	RL29_MOU	18 kDa	0.998518	2	100%	P47915	Rpl29	60S ribosor	18.46726	20.34786	18.94775	21.21091	17.87443	20.19413	-2.15449	-3.15835	-1.15062	19.50706	-5.12177	-0.92546	0.001563	0.012896	-1	-1
1169	sp|Q7TQA1	IGSF1_MOU	147 kDa	0.998241	2	100%	Q7TQA1	Igsf1	Immunoglo	20.38809	23.01611	21.21515	22.93423	20.39119	22.10245	-2.01945	-2.96112	-1.07778	21.67454	-5.11784	-0.92995	0.001569	0.012896	-1	-1
699	sp|P33267	CP2F2_MO	56 kDa	0.998901	4	100%	P33267	Cyp2f2	Cytochrom	19.63472	17.50575	19.24635	17.57778	19.99771	18.32354	1.823905	0.969059	2.678751	18.71431	5.085218	-0.95373	0.001596	0.013037	1	1
1060.2	tr|A2A4X6	A2A4X6_M	19 kDa	0.999248	2	100%	A2A4X6	Gm12355	MCG21910	20.09105	21.88482	20.20941	21.18869	19.83026	21.66737	-1.53672	-2.2627	-0.81074	20.81193	-5.03813	-0.99375	0.001648	0.013378	-1	-1
592	sp|Q9D1G1	RAB1B_MO	22 kDa	0.999802	4	25%	Q9D1G1	Rab1b	Ras-related	19.76854	18.12916	20.66956	18.64778	19.87042	18.24636	1.761741	0.926592	2.596891	19.22197	5.02111	-1.0141	0.00168	0.01351	1	1
916.15	tr|Q8CGP4	Q8CGP4_M	14 kDa	0.998436	3	33%	Q8CGP4	Hist1h2aa	Histone H2	22.70511	24.71866	21.19408	24.28151	22.63311	24.54156	-2.33648	-3.4401	-1.23286	23.34567	-5.05234	-1.00511	0.001684	0.01351	-1	-1
142	sp|Q8BG05	ROA3_MOU	40 kDa	0.999911	12	75%	Q8BG05	Hnrnpa3	Heterogene	25.09629	23.31561	24.26269	23.01576	24.88287	23.11078	1.599898	0.839059	2.360738	23.94733	5.004955	-1.03247	0.001709	0.013629	1	1
719	sp|Q02819	NUCB1_M	53 kDa	0.998696	4	100%	Q02819	Nucb1	Nucleobind	20.65448	21.99998	20.00173	22.41841	20.34966	21.62265	-1.67839	-2.47787	-0.87891	21.17448	-4.99675	-1.04207	0.001724	0.013654	-1	-1
1250	sp|Q9D1R9	RL34_MOU	13 kDa	0.996907	2	100%	Q9D1R9	Rpl34	60S ribosor	18.36554	20.49861	16.84509	19.59845	16.29315	20.05903	-2.88411	-4.25345	-1.51476	18.60998	-5.02631	-1.03516	0.001732	0.013654	-1	-1
1259	sp|Q9CU62	SMC1A_M	143 kDa	0.996524	2	100%	Q9CU62	Smc1a	Structural	19.69182	17.35776	20.37165	18.03578	19.11579	16.78584	2.333295	1.22361	3.44298	18.55977	5.017885	-1.0449	0.001748	0.013699	1	1
23	sp|Q61768	KINH_MOU	110 kDa	0.999746	27	81%	Q61768	Kif5b	Kinesin-1 h	24.23015	22.50157	24.08387	22.4242	24.27546	23.13422	1.509827	0.785926	2.233728	23.44158	4.964192	-1.08027	0.001787	0.013927	1	1
111	sp|Q62095	DDX3Y_M	73 kDa	0.997675	15	13%	Q62095	Ddx3y	ATP-depen	19.10992	17.52939	19.08762	16.97791	18.737	17.74815	1.559697	0.805989	2.313406	18.19833	4.925359	-1.12604	0.001865	0.014455	1	1
788	sp|P16125	LDHB_MOU	37 kDa	0.999654	3	67%	P16125	Ldhb	L-lactate de	21.37743	17.29409	19.77552	17.52084	22.76833	18.14281	3.654516	1.893459	5.415572	19.47984	4.952303	-1.12114	0.001877	0.014463	1	1
765	sp|P12382	PFKAL_MO	85 kDa	0.997215	4	50%	P12382	Pfkl	ATP-depen	20.07549	21.14724	20.12847	21.53602	19.63317	21.39185	-1.41266	-2.0977	-0.72762	20.65204	-4.90822	-1.14632	0.001901	0.014566	-1	-1
140	sp|Q6ZQ38	CAND1_M	136 kDa	0.999055	13	100%	Q6ZQ38	Cand1	Cullin-asso	23.07882	25.01108	23.75891	24.58636	23.03663	25.13218	-1.61842	-2.40482	-0.83203	24.10066	-4.89837	-1.15798	0.001922	0.014644	-1	-1
472	tr|A0A075	A0A075B5	13 kDa	0.997707	6	17%	A0A075B5	Ighv1-22	Immunoglo	15.67546	17.83639	16.93668	19.07442	16.01395	18.7235	-2.33607	-3.46928	-1.20286	17.37673	-4.91956	-1.15945	0.001946	0.014741	-1	-1
1098	sp|Q8CH18	CCAR1_MO	132 kDa	0.99899	2	100%	Q8CH18	Ccar1	Cell divisio	16.3035	21.98187	16.40305	19.46667	17.0631	19.23739	-3.63876	-5.41676	-1.86076	18.40926	-4.88395	-1.20129	0.002024	0.015179	-1	-1
430	sp|P01810	HVM40_M	13 kDa	0.99932	6	17%	P01810	NA	Ig heavy ch	23.84835	25.8581	24.30025	25.89394	23.23519	25.21181	-1.86002	-2.77044	-0.94959	24.7246	-4.87054	-1.20704	0.002026	0.015179	-1	-1
503	sp|Q9JM93	AR6P4_MO	26 kDa	0.999531	5	100%	Q9JM93	Arl6ip4	ADP-ribosy	22.14555	23.97164	22.74135	24.49789	23.04196	24.73911	-1.75993	-2.62535	-0.89451	23.52292	-4.848	-1.23353	0.002077	0.015474	-1	-1
616	sp|P62821	RAB1A_MO	23 kDa	0.999554	4	25%	P62821	Rab1A	Ras-related	19.98859	18.9982	20.65602	18.91909	20.51377	18.20002	1.680356	0.845515	2.515197	19.54595	4.790927	-1.28672	0.00217	0.016076	1	1
631	sp|Q924W	RBTN1_MO	18 kDa	0.999432	4	25%	Q924W9	Lmo1	Rhombotin	20.35864	15.1481	20.83886	17.96954	19.56281	16.95335	3.563107	1.795188	5.331027	18.47189	4.809689	-1.28917	0.002197	0.016125	1	1
524	sp|Q9CZM2	RL15_MOU	24 kDa	0.999297	5	100%	Q9CZM2	Rpl15	60S ribosor	24.96063	23.24249	24.88872	23.63036	24.9648	23.87104	1.356754	0.680046	2.033462	24.25967	4.772004	-1.30903	0.002215	0.016125	1	1
741	sp|P49615	CDK5_MOU	33 kDa	0.9981	4	100%	P49615	Cdk5	Cyclin-depe	20.73597	19.32378	21.71029	19.15244	20.60443	19.31223	1.754077	0.880937	2.627218	20.13986	4.789152	-1.30366	0.002218	0.016125	1	1
1114	sp|Q9DCX2	ATP5H_MO	19 kDa	0.998808	2	100%	Q9DCX2	Atp5pd	ATP syntha	24.55405	25.93757	23.79019	25.79254	24.63254	25.86288	-1.53873	-2.30744	-0.77003	25.09496	-4.76438	-1.31822	0.002234	0.016125	-1	-1
328	sp|Q8BRM	GORAB_M	41 kDa	0.999541	7	100%	Q8BRM2	Gorab	RAB6-intera	24.13988	21.38348	22.70421	21.12528	23.93925	21.7973	2.159091	1.084359	3.233824	22.5149	4.794256	-1.30754	0.002236	0.016125	1	1
812	sp|Q61753	SERA_MOU	57 kDa	0.999441	3	100%	Q61753	Phgdh	D-3-phosph	18.26501	15.45136	18.07564	14.6353	17.04925	16.12053	2.394237	1.1893	3.599173	16.59952	4.741913	-1.37011	0.002371	0.017008	1	1
944	sp|Q8K019	BCLF1_MO	106 kDa	0.998011	3	100%	Q8K019	Bclaf1	Bcl-2-assoc	21.51691	20.60907	21.94028	20.36035	22.17413	20.55908	1.367607	0.669924	2.065289	21.1933	4.665565	-1.43814	0.0025	0.017844	1	1
83	sp|Q8VDN	AT1A1_MO	113 kDa	0.998975	17	59%	Q8VDN2	Atp1a1	Sodium/po	23.47933	25.19346	24.01967	24.79375	23.26609	25.25398	-1.49204	-2.25466	-0.72942	24.33438	-4.65665	-1.44903	0.002526	0.017932	-1	-1
163	sp|Q8BPB5	FBLN3_MO	55 kDa	0.999504	11	100%	Q8BPB5	Efemp1	EGF-contai	25.46593	23.13289	24.44394	23.06345	25.39581	23.70309	1.802079	0.881151	2.723007	24.20085	4.668441	-1.45602	0.002566	0.017983	1	1
341	sp|P50516	VATA_MOU	68 kDa	0.999405	7	100%	P50516	Atp6v1a	V-type prot	22.05077	24.05637	22.30866	23.72622	22.79099	23.69231	-1.4415	-2.18053	-0.70246	23.10422	-4.64249	-1.46637	0.002567	0.017983	-1	-1
871	sp|P48758	CBR1_MOU	31 kDa	0.998987	3	67%	P48758	Cbr1	Carbonyl re	20.78965	22.03135	21.29824	22.7534	20.54205	22.71952	-1.62478	-2.45805	-0.7915	21.68903	-4.64116	-1.46843	0.002573	0.017983	-1	-1
372	sp|P80560	PTPR2_MO	111 kDa	0.998928	7	86%	P80560	Ptprn2	Receptor-ty	21.65221	22.84576	21.98758	23.17468	21.52102	23.67379	-1.51114	-2.287	-0.73528	22.47584	-4.63579	-1.47458	0.002587	0.017991	-1	-1
1046	sp|Q80TT2	BAIP3_MO	127 kDa	0.999382	2	100%	Q80TT2	Baiap3	BAI1-associ	18.3657	15.61973	18.83519	17.41713	19.38739	16.12937	2.474014	1.205602	3.742425	17.62575	4.654709	-1.47529	0.002616	0.018057	1	1
790	sp|P03976	KV2A5_MO	12 kDa	0.999638	3	33%	P03976	NA	Ig kappa ch	21.81688	19.43468	20.28002	18.3122	21.67842	17.72153	2.768972	1.348592	4.189351	19.87395	4.652269	-1.47825	0.002623	0.018057	1	1
1191.1	sp|P36536	SAR1A_MO	22 kDa	0.998052	2	100%	P36536	Sar1a	GTP-bindin	17.67067	20.11671	18.4915	19.82267	18.71094	19.83924	-1.63517	-2.47763	-0.7927	19.10862	-4.62303	-1.49671	0.002649	0.018142	-1	-1
1015.1	sp|P47758	SRPRB_MO	30 kDa	0.99986	2	100%	P47758	Srprb	Signal reco	21.12096	23.99881	21.22367	23.19838	22.45571	24.18518	-2.19401	-3.32386	-1.06417	22.69712	-4.63416	-1.50024	0.002678	0.018163	-1	-1
1216	sp|Q3UPF5	ZCCHV_MO	107 kDa	0.997739	2	100%	Q3UPF5	Zc3hav1	Zinc finger	20.17213	21.26897	20.4747	22.14526	20.01053	21.8335	-1.53012	-2.32098	-0.73927	20.98418	-4.60499	-1.5124	0.002681	0.018163	-1	-1
859.2	sp|Q6PDQ	CHD4_MOU	218 kDa	0.99907	3	100%	Q6PDQ2	Chd4	Chromodo	22.31997	24.12919	22.92134	24.56679	22.56858	23.73819	-1.54143	-2.33953	-0.74333	23.37401	-4.59694	-1.52232	0.002706	0.018163	-1	-1
860	sp|Q7TMF3	NDUAC_M	17 kDa	0.999069	3	100%	Q7TMF3	Ndufa12	NADH dehy	22.31997	24.12919	22.92134	24.56679	22.56858	23.73819	-1.54143	-2.33953	-0.74333	23.37401	-4.59694	-1.52232	0.002706	0.018163	-1	-1
1153	sp|Q7TNG	LDHD_MOU	52 kDa	0.998398	2	100%	Q7TNG8	Ldhd	Probable D	19.67819	21.57733	19.17163	21.6382	20.60031	22.08775	-1.95105	-2.9622	-0.9399	20.79223	-4.60474	-1.53608	0.002769	0.0185	-1	-1
310	sp|Q8VH51	RBM39_M	59 kDa	0.999697	7	100%	Q8VH51	Rbm39	RNA-bindin	23.28023	24.50378	23.38271	24.53827	23.06747	24.29109	-1.20091	-1.82752	-0.5743	23.84393	-4.56156	-1.56599	0.002819	0.018737	-1	-1
1195	sp|Q9EQ06	DHB11_M	33 kDa	0.998018	2	100%	Q9EQ06	Hsd17b11	Estradiol 1	17.55304	20.36387	17.31148	19.48534	18.65086	19.80308	-2.04564	-3.11407	-0.97721	18.86128	-4.56912	-1.57965	0.002885	0.019082	-1	-1
1056.2	tr|Q63ZW6	Q63ZW6_M	162 kDa	0.999285	2	100%	Q63ZW6	Col4a5	Col4a5 pro	19.54683	18.24412	19.82987	18.05429	19.42494	16.95742	1.848605	0.881459	2.815752	18.67624	4.561446	-1.58906	0.00291	0.019158	1	1
1022	sp|P00687	AMY1_MOU	58 kDa	0.999718	2	100%	P00687	Amy1	Alpha-amyl	22.9301	20.18507	21.64963	18.50636	23.00559	20.3898	2.834698	1.34859	4.320806	21.11109	4.55205	-1.6006	0.002942	0.019244	1	1
1156	sp|P84309	ADCY5_MO	139 kDa	0.998382	2	100%	P84309	Adcy5	Adenylate c	18.79536	19.80088	18.23325	20.04702	17.82734	19.62704	-1.53966	-2.35002	-0.72931	19.05515	-4.52242	-1.61494	0.002952	0.019244	-1	-1
983	sp|Q8BU30	SYIC_MOUS	144 kDa	0.997151	3	100%	Q8BU30	Iars	Isoleucine-	17.57924	19.69756	15.54359	19.24074	16.4751	18.52327	-2.62121	-4.00471	-1.23771	17.84325	-4.5214	-1.63832	0.003048	0.019775	-1	-1
1212	sp|P70280	VAMP7_M	25 kDa	0.997775	2	100%	P70280	Vamp7	Vesicle-ass	21.74346	23.0541	21.66027	23.37537	22.18273	23.04085	-1.29462	-1.98137	-0.60788	22.50946	-4.48695	-1.65872	0.003075	0.019857	-1	-1
901	sp|P61255	RL26_MOU	17 kDa	0.99868	3	100%	P61255	Rpl26	60S ribosor	24.22743	25.74522	24.67834	25.54254	24.42699	25.67184	-1.20895	-1.85119	-0.56671	25.04873	-4.48036	-1.66695	0.003099	0.019916	-1	-1
824	tr|A0A140	A0A140T8	13 kDa	0.999377	3	100%	A0A140T8	Igkv13-84	Immunoglo	23.25403	22.29525	23.27321	21.35865	23.14747	21.18339	1.61248	0.747207	2.477753	22.41867	4.442578	-1.72742	0.0033	0.021108	1	1
436	sp|Q8VED5	K2C79_MO	58 kDa	0.99927	6	17%	Q8VED5	Krt79	Keratin, typ	24.02412	24.99836	23.48772	25.05226	23.6187	25.98452	-1.63486	-2.5154	-0.75432	24.52761	-4.42615	-1.74796	0.003364	0.021255	-1	-1
958	sp|P99029	PRDX5_MO	22 kDa	0.997769	3	100%	P99029	Prdx5	Peroxiredo	21.3084	22.36403	21.05864	22.15546	20.97149	22.29167	-1.15754	-1.78239	-0.5327	21.69161	-4.40927	-1.75617	0.00337	0.021255	-1	-1
311	tr|A0A075	A0A075B5	13 kDa	0.999685	7	43%	A0A075B5	Ighv4-1	Immunoglo	26.70387	28.2283	26.98323	28.15251	26.16557	27.77908	-1.43574	-2.21077	-0.66071	27.33543	-4.40917	-1.75629	0.00337	0.021255	-1	-1
560	sp|P14106	C1QB_MOU	27 kDa	0.998823	5	100%	P14106	C1qb	Compleme	26.98189	28.1934	27.18665	28.42181	26.79338	28.00411	-1.21913	-1.8792	-0.55907	27.59687	-4.39606	-1.77284	0.003423	0.021488	-1	-1
894	sp|P21107	TPM3_MOU	33 kDa	0.998754	3	33%	P21107	Tpm3	Tropomyos	23.42961	22.32265	24.02872	22.19628	23.23099	22.03522	1.378388	0.630675	2.126101	22.87391	4.387702	-1.78339	0.003457	0.021602	1	1
1019	sp|P07759	SPA3K_MO	47 kDa	0.999735	2	100%	P07759	Serpina3k	Serine prot	18.43752	14.04793	19.3601	NA	18.39439	16.31902	3.547192	1.705851	5.388533	17.31179	4.775089	-1.56869	0.003536	0.021945	1	1
917	sp|Q6IME9	K2C72_MO	57 kDa	0.998428	3	33%	Q6IME9	Krt72	Keratin, typ	18.93367	20.01606	18.19652	19.8107	18.74804	20.04097	-1.32983	-2.05467	-0.60499	19.291	-4.36672	-1.80994	0.003544	0.021945	-1	-1
77	sp|Q91XU	WRIP1_M	72 kDa	0.999602	17	100%	Q91XU0	Wrnip1	ATPase WR	25.96271	22.74806	24.0297	22.65917	26.33713	22.96971	2.650867	1.199294	4.10244	24.11775	4.358126	-1.84173	0.003688	0.02264	1	1
990	sp|Q3THK7	GUAA_MOU	77 kDa	0.99685	3	100%	Q3THK7	Gmps	GMP synth	19.43538	18.31149	19.37049	17.27847	19.24929	16.69458	1.92354	0.870142	2.976938	18.38995	4.357721	-1.84224	0.00369	0.02264	1	1
456	sp|Q6ZQ58	LARP1_MO	121 kDa	0.998957	6	100%	Q6ZQ58	Larp1	La-related	20.37757	22.14973	18.76882	21.93413	20.51588	22.09752	-2.17303	-3.36601	-0.98005	20.97394	-4.34694	-1.85581	0.003737	0.022827	-1	-1
136	sp|P14148	RL7_MOUS	31 kDa	0.99931	13	100%	P14148	Rpl7	60S ribosor	25.84312	24.28867	26.35916	24.82055	25.8448	24.87377	1.354696	0.607028	2.102364	25.33834	4.312547	-1.87881	0.003781	0.022951	1	1
321	sp|O88665	BRD7_MOU	74 kDa	0.999593	7	100%	O88665	Brd7	Bromodom	22.8287	20.91494	21.83513	20.89656	23.0596	20.983	1.642973	0.737377	2.54857	21.75299	4.325039	-1.87532	0.003791	0.022951	1	1
178	sp|Q68FH0	PKP4_MOU	132 kDa	0.999035	11	100%	Q68FH0	Pkp4	Plakophilin	22.72108	20.14036	21.18809	19.96626	21.97705	20.28417	1.83181	0.814718	2.848901	21.04617	4.298044	-1.91761	0.00396	0.02377	1	1
28	sp|A2BH40	ARI1A_MOU	242 kDa	0.999848	25	92%	A2BH40	Arid1a	AT-rich inte	23.41653	22.00725	22.88232	21.53515	23.66679	22.13474	1.429503	0.633365	2.225641	22.60713	4.273634	-1.92856	0.003962	0.02377	1	1
702	sp|Q9WV5	VAPA_MOU	28 kDa	0.998865	4	75%	Q9WV55	Vapa	Vesicle-ass	21.03147	20.30019	21.34536	19.71504	21.1408	19.77819	1.241406	0.543529	1.939283	20.55184	4.233854	-1.97965	0.004157	0.024829	1	1
654	tr|E9Q0F0	E9Q0F0_M	112 kDa	0.999273	4	50%	E9Q0F0	Krt78	Keratin 78	25.82564	27.54229	26.53719	27.80519	26.68771	28.27118	-1.5227	-2.379	-0.66641	27.11154	-4.23894	-1.98449	0.004199	0.024944	-1	-1
717	sp|Q9CYH2	PXL2A_MO	24 kDa	0.998714	4	100%	Q9CYH2	Prxl2a	Peroxiredo	20.74462	19.6203	21.02182	19.35665	20.32793	19.31563	1.267262	0.552984	1.981541	20.06449	4.222793	-1.9939	0.004213	0.024944	1	1
567	sp|P58269	DPF3_MOU	43 kDa	0.99854	5	80%	P58269	Dpf3	Zinc finger	17.7865	18.80425	17.87035	19.49178	17.77296	20.21977	-1.69533	-2.64963	-0.74103	18.6576	-4.23954	-1.99203	0.004246	0.025026	-1	-1
24	sp|P28738	KIF5C_MOU	109 kDa	0.999682	27	74%	P28738	Kif5c	Kinesin hea	26.14704	27.13623	25.48745	27.13381	26.15793	27.62057	-1.36606	-2.13783	-0.59429	26.61384	-4.21291	-2.00664	0.004263	0.025026	-1	-1
1027	sp|P04247	MYG_MOUS	17 kDa	0.999598	2	100%	P04247	Mb	Myoglobin	19.8928	18.99333	20.06902	18.93971	20.65781	18.32598	1.453536	0.631476	2.275597	19.47977	4.208661	-2.01249	0.004288	0.02506	1	1
123	sp|Q6IFX2	K1C42_MO	50 kDa	0.998174	14	29%	Q6IFX2	Krt42	Keratin, typ	22.57409	24.7726	23.7388	25.0831	23.72628	25.27867	-1.6984	-2.65783	-0.73897	24.19559	-4.22453	-2.01121	0.004324	0.025066	-1	-1
370	sp|Q64331	MYO6_MOU	146 kDa	0.998994	7	100%	Q64331	Myo6	Unconvent	20.78207	22.9034	21.20147	23.22503	20.65398	21.85456	-1.78182	-2.78917	-0.77447	21.77008	-4.2212	-2.01547	0.004341	0.025066	-1	-1
174	sp|P51881	ADT2_MOU	33 kDa	0.999284	11	55%	P51881	Slc25a5	ADP/ATP tr	24.52507	23.05248	23.82315	22.66325	24.18888	22.97536	1.282003	0.555055	2.008951	23.53803	4.197458	-2.02661	0.004344	0.025066	1	1
483	sp|O08547	SC22B_MO	25 kDa	0.999894	5	100%	O08547	Sec22b	Vesicle-traf	23.79215	22.57444	24.05767	22.73716	23.46862	22.24778	1.25302	0.538889	1.967152	23.1463	4.176194	-2.05413	0.004458	0.025501	1	1
621	sp|Q9ER00	STX12_MO	31 kDa	0.999496	4	100%	Q9ER00	Stx12	Syntaxin-12	23.70315	22.0989	23.50824	20.65139	23.50365	22.18932	1.925143	0.830354	3.019932	22.60911	4.196461	-2.04716	0.004472	0.025501	1	1
720	sp|Q6ZPV2	INO80_MO	177 kDa	0.998686	4	100%	Q6ZPV2	Ino80	Chromatin-	18.01565	18.8961	17.12394	18.80979	17.46178	18.87278	-1.32577	-2.08196	-0.56958	18.19667	-4.17288	-2.05842	0.004476	0.025501	-1	-1
394	sp|Q9CZT8	RAB3B_MO	25 kDa	0.999744	6	33%	Q9CZT8	Rab3b	Ras-related	22.3992	21.13097	22.84088	20.58854	22.00084	21.22692	1.4315	0.611413	2.251587	21.69789	4.154831	-2.08221	0.004578	0.025802	1	1
330	sp|Q9Z0H3	SNF5_MOU	44 kDa	0.999526	7	100%	Q9Z0H3	Smarcb1	SWI/SNF-re	24.42627	22.33792	23.4462	21.24587	24.44884	22.66562	2.023966	0.867448	3.180484	23.09512	4.176393	-2.07294	0.004581	0.025802	1	1
543	sp|P21956	MFGM_MO	51 kDa	0.999127	5	100%	P21956	Mfge8	Lactadherin	20.43569	19.30064	21.21015	19.72779	20.78354	19.4737	1.309081	0.558832	2.059331	20.15525	4.152997	-2.08424	0.004586	0.025802	1	1
918	sp|Q69ZQ2	ISY1_MOUS	33 kDa	0.998423	3	100%	Q69ZQ2	Isy1	Pre-mRNA-	22.5809	24.67487	22.99313	24.77975	21.84843	23.63831	-1.89016	-2.97268	-0.80764	23.41923	-4.1669	-2.08516	0.004634	0.025929	-1	-1
596	sp|Q99K48	NONO_MO	55 kDa	0.999738	4	75%	Q99K48	Nono	Non-POU d	20.89474	19.68457	21.03482	19.68744	20.48027	19.06889	1.322976	0.562791	2.083162	20.14179	4.142221	-2.09826	0.004647	0.025929	1	1
675	sp|P05132	KAPCA_MO	41 kDa	0.999091	4	50%	P05132	Prkaca	cAMP-depe	19.83985	21.77463	19.78772	21.18173	20.64757	22.04998	-1.57706	-2.4851	-0.66903	20.88025	-4.14048	-2.11201	0.004735	0.026134	-1	-1
1277	sp|Q9D287	SPF27_MO	26 kDa	0.99577	2	100%	Q9D287	Bcas2	Pre-mRNA-	18.70835	13.9885	18.59317	17.27421	18.81194	15.04786	3.267629	1.385691	5.149567	17.07067	4.143602	-2.1152	0.004767	0.026134	1	1
1278.1	sp|Q7TSF0	DSG1C_M	101 kDa	0.995714	2	100%	Q7TSF0	Dsg1c	Desmoglein	18.70835	13.9885	18.59317	17.27421	18.81194	15.04786	3.267629	1.385691	5.149567	17.07067	4.143602	-2.1152	0.004767	0.026134	1	1
653	sp|P42225	STAT1_MO	87 kDa	0.999283	4	100%	P42225	Stat1	Signal trans	20.35865	19.5651	20.85073	19.40968	20.54185	19.40103	1.12514	0.475323	1.774957	20.02117	4.121132	-2.12573	0.004768	0.026134	1	1
579	sp|P63087	PP1G_MOU	37 kDa	0.997723	5	20%	P63087	Ppp1cc	Serine/thre	19.43867	21.97419	20.38385	21.23431	20.21514	22.78308	-1.98464	-3.12828	-0.84101	21.00487	-4.14139	-2.11806	0.00478	0.026134	-1	-1
774	tr|A0A075	A0A075B5	13 kDa	0.995633	4	25%	A0A075B5	Ighv7-3	Immunoglo	22.6625	21.39578	22.08317	21.19738	22.67557	21.21905	1.203013	0.50692	1.899106	21.87224	4.113428	-2.13579	0.004813	0.026213	1	1
1152	sp|P01844	LAC2_MOU	11 kDa	0.99843	2	50%	P01844	Iglc2	Ig lambda-2	20.71314	19.61536	20.98702	18.86239	20.24305	19.26072	1.401577	0.589401	2.213754	19.94695	4.107604	-2.14373	0.00485	0.026309	1	1
744	sp|P54823	DDX6_MOU	54 kDa	0.997909	4	100%	P54823	Ddx6	Probable A	22.85688	25.07803	24.25186	26.20248	24.08181	26.17252	-2.08749	-3.29646	-0.87853	24.77393	-4.12061	-2.14492	0.004902	0.026481	-1	-1
628	tr|A0A140	A0A140T8	13 kDa	0.99946	4	50%	A0A140T8	Igkv8-30	Immunoglo	21.70008	23.91505	22.5471	24.33119	21.3175	23.08488	-1.92215	-3.03787	-0.80643	22.81597	-4.11133	-2.15694	0.004957	0.026675	-1	-1
507	tr|A0A075	A0A075B5	13 kDa	0.999496	5	20%	A0A075B5	Ighv6-6	Immunoglo	23.10393	25.22683	23.57669	25.34276	22.26528	24.21038	-1.94469	-3.07589	-0.81348	23.95431	-4.1026	-2.16827	0.00501	0.026853	-1	-1
468	sp|O70228	ATP9A_MO	119 kDa	0.998178	6	67%	O70228	Atp9a	Probable p	20.46668	19.03661	20.53157	19.15316	20.25761	19.58721	1.159627	0.481043	1.838212	19.83881	4.067386	-2.19606	0.005094	0.027196	1	1
903.1	sp|Q6GSS7	H2A2A_M	14 kDa	0.998649	3	33%	Q6GSS7	Hist2h2aa2	Histone H2	20.47444	22.10716	19.57708	21.36901	20.57363	21.82685	-1.55929	-2.47429	-0.64429	20.98803	-4.06359	-2.21363	0.005214	0.027525	-1	-1
274	sp|P16014	SCG1_MOU	78 kDa	0.999505	8	100%	P16014	Chgb	Secretogra	22.73764	23.72222	22.2271	23.6863	22.81412	23.85366	-1.16111	-1.84372	-0.47849	23.17351	-4.04853	-2.22084	0.005214	0.027525	-1	-1
21	sp|O08810	U5S1_MOU	109 kDa	0.999725	28	96%	O08810	Eftud2	116 kDa U5	25.76133	24.08064	24.68752	22.86079	26.05939	23.60689	1.986638	0.821078	3.152199	24.50943	4.067567	-2.21381	0.005229	0.027525	1	1
1234	tr|Q9JHF5	Q9JHF5_M	93 kDa	0.997355	2	50%	Q9JHF5	Tcirg1	V-type prot	23.00079	21.08417	23.83329	21.73598	22.65635	21.76223	1.636017	0.675878	2.596156	22.34547	4.066346	-2.2154	0.005237	0.027525	1	1
196	sp|Q80WJ	LYRIC_MO	64 kDa	0.999538	10	100%	Q80WJ7	Mtdh	Protein LYF	24.06499	23.15208	24.28559	22.94461	24.34649	23.33512	1.088419	0.447462	1.729377	23.68815	4.041735	-2.22978	0.005258	0.02753	1	1
776	sp|Q9D0M	DYL2_MOU	10 kDa	0.995494	4	50%	Q9D0M5	Dynll2	Dynein ligh	22.52327	20.83074	22.79541	21.85832	22.67702	21.11447	1.39739	0.573796	2.220984	21.96654	4.038558	-2.23429	0.005282	0.027546	1	1

612	tr|A0A140	A0A140T8	13 kDa	0.999605	4	25%	A0A140T8	Igkv6-23	Immunogl	20.91445	23.2108	22.11242	23.42372	20.73671	22.63175	-1.83423	-2.91343	-0.75503	22.17164	-4.05604	-2.22884	0.005304	0.027554	-1	-1
963	sp|Q3UHK	TNR6A_MO	203 kDa	0.997696	3	100%	Q3UHK8	Tnrc6a	Trinucleoti	18.3557	20.51249	19.01337	20.02509	NA	20.34402	-1.60933	-2.52615	-0.69251	19.65013	-4.33477	-2.02574	0.005344	0.027658	-1	-1
969	sp|Q0846	KCMA1_M	134 kDa	0.997601	3	100%	Q08460	Kcnma1	Calcium-ac	17.678	15.88339	18.00248	16.26589	17.57318	16.89396	1.403468	0.57301	2.233927	17.04948	4.022597	-2.25532	0.005387	0.027767	1	1
167	sp|Q91VR5	DDX1_MO	82 kDa	0.999436	11	100%	Q91VR5	Ddx1	ATP-depend	23.30122	21.80544	22.70698	21.38053	22.84285	21.92862	1.245489	0.50796	1.983019	22.32761	4.019401	-2.25922	0.005406	0.027767	1	1
826	sp|Q9CQN	TRAP1_MO	80 kDa	0.999366	3	100%	Q9CQN1	Trap1	Heat shock	21.91922	18.94401	19.9797	19.23083	22.06275	18.69712	2.363236	0.965036	3.761437	20.13894	4.033558	-2.2582	0.005452	0.027897	1	1
599	tr|A0A075	A0A075B5	10 kDa	0.999712	4	75%	A0A075B5	Igkv9-124	Immunogl	20.57192	22.66423	21.69615	23.13404	20.39761	22.24333	-1.79197	-2.85393	-0.73002	21.78455	-4.02696	-2.26684	0.005496	0.028019	-1	-1
348	sp|P23249	MOV10_M	114 kDa	0.999342	7	100%	P23249	Mov10	Putative he	21.27676	19.93984	21.55647	20.3593	20.98829	19.98992	1.177482	0.475892	1.879071	20.6851	3.994585	-2.29202	0.005575	0.028171	1	1
1096	sp|O08807	PRDX4_M	31 kDa	0.999	2	50%	O08807	Prdx4	Peroxiredo	17.9931	19.94334	18.22076	20.20226	18.03585	18.89354	-1.59647	-2.54548	-0.64747	18.88147	-4.01461	-2.28301	0.00558	0.028171	-1	-1
116	sp|Q8CGC7	SYEP_MOU	170 kDa	0.999741	14	100%	Q8CGC7	Eprs	Bifunctiona	23.73363	25.04604	23.68682	25.21788	24.44025	25.48289	-1.29537	-2.06757	-0.52317	24.60125	-3.9927	-2.29452	0.005589	0.028171	-1	-1
608	sp|Q99M2	RNPS1_MO	34 kDa	0.999648	4	100%	Q99M28	Rnps1	RNA-bindin	23.17282	21.85916	22.4934	21.54962	22.90669	21.61598	1.182714	0.472782	1.892646	22.26628	3.965189	-2.33098	0.005784	0.029047	1	1
59	sp|Q3TKT4	SMCA4_M	181 kDa	0.999626	19	58%	Q3TKT4	Smarca4	Transcripti	23.64992	21.92278	22.80156	22.20405	24.01612	21.35074	1.663339	0.66639	2.660288	22.65753	3.981607	-2.32635	0.005812	0.029083	1	1
652	sp|Q9R226	KHDR3_M	39 kDa	0.99932	4	75%	Q9R226	Khdrbs3	KH domain	23.03737	20.64425	21.33682	19.77296	22.90573	20.54607	2.105547	0.835703	3.375391	21.37387	3.956992	-2.35877	0.005992	0.029873	1	1
919	sp|Q99KQ	NAMPT_M	55 kDa	0.998411	3	100%	Q99KQ4	Nampt	Nicotinami	19.43594	21.40354	20.3241	21.20609	20.1842	21.21246	-1.29262	-2.07485	-0.51038	20.62772	-3.9331	-2.37367	0.006021	0.029906	-1	-1
5	sp|Q8R0W	EPIPL_MOU	723 kDa	0.999889	53	96%	Q8R0W0	Eppk1	Epiplakin	26.42704	23.51274	24.96372	24.08294	26.96435	24.25857	2.166955	0.852678	3.481232	25.03489	3.934718	-2.3882	0.00616	0.030487	1	1
428	sp|P70333	HNRH2_M	49 kDa	0.999352	6	33%	P70333	Hnrnph2	Heterogene	22.53427	20.68878	23.45805	20.71131	23.04984	22.05715	1.861641	0.730894	2.992388	22.08323	3.928993	-2.39577	0.006204	0.030593	1	1
536	sp|Q922Q	LRC59_MO	35 kDa	0.999211	5	100%	Q922Q8	Lrrc59	Leucine-ric	22.67913	21.96097	22.66218	21.11112	22.70258	21.02209	1.316565	0.514377	2.118754	22.02301	3.90631	-2.40942	0.006227	0.030594	1	1
705	sp|P01786	HVM17_M	12 kDa	0.998848	4	25%	P01786	NA	Ig heavy ch	23.3802	25.11393	23.71698	25.62746	22.65071	24.32328	-1.77226	-2.8519	-0.69261	24.13543	-3.91739	-2.41113	0.006295	0.030671	-1	-1
559	sp|Q80YR5	SAFB2_MO	112 kDa	0.99883	5	100%	Q80YR5	Safb2	Scaffold att	23.31818	21.69918	22.26191	21.08378	23.0486	21.47517	1.456853	0.568295	2.345411	22.1478	3.908624	-2.41626	0.006302	0.030671	1	1
938	sp|Q8C0C7	SYFA_MOU	58 kDa	0.99806	3	100%	Q8C0C7	Farsa	Phenylalan	22.4362	23.05793	22.19186	23.36902	21.99061	23.60554	-1.13794	-1.83317	-0.44271	22.77519	-3.89574	-2.42355	0.006311	0.030671	-1	-1
418	sp|P70372	ELAV1_MO	36 kDa	0.999519	6	100%	P70372	Elavl1	ELAV-like p	23.48833	22.39918	23.45239	22.14754	23.07889	22.30528	1.055871	0.408458	1.703284	22.81194	3.881775	-2.44225	0.006423	0.031104	1	1
671	sp|Q922H4	GMPPA_M	46 kDa	0.999115	4	100%	Q922H4	Gmppa	Mannose-1	21.88209	20.61914	21.71688	21.04773	21.85652	20.61797	1.056882	0.407984	1.70578	21.29006	3.876601	-2.44919	0.006465	0.031134	1	1
400	sp|Q6ZWV	RL10_MOU	25 kDa	0.99967	6	100%	Q6ZWV3	Rpl10	60S riboson	23.70277	22.76863	24.73022	22.67266	23.89147	22.84036	1.347603	0.519987	2.175219	23.43435	3.875745	-2.45063	0.006475	0.031134	1	1
148	sp|Q9WU2	MTHR_MO	75 kDa	0.999556	12	100%	Q9WU20	Mthfr	Methylenet	25.13436	23.70929	24.14165	22.34666	25.26627	21.77208	2.238083	0.863195	3.61297	23.72839	3.884718	-2.45451	0.006557	0.031417	1	1
1090	sp|Q9CYR6	AGM1_MO	59 kDa	0.999025	2	100%	Q9CYR6	Pgm3	Phosphoac	21.62672	23.36316	21.61716	22.77109	21.69295	22.47944	-1.22562	-1.98167	-0.46957	22.25842	-3.85841	-2.47361	0.006615	0.031586	-1	-1
338	sp|Q9CQV	1433B_MO	28 kDa	0.999452	7	43%	Q9CQV8	Ywhab	14-3-3 prot	20.69061	22.25122	20.94938	21.77248	20.85264	21.74035	-1.09047	-1.76435	-0.4166	21.37611	-3.85157	-2.48279	0.006673	0.031749	-1	-1
632	tr|A0A075	A0A075B5	13 kDa	0.999431	4	25%	A0A075B5	Ighv14-1	Immunogl	20.65544	22.77383	22.0985	24.27004	21.14302	23.09677	-2.08123	-3.367	-0.79545	22.3396	-3.86282	-2.48366	0.006739	0.031952	-1	-1
916.12	sp|Q8CGP	H2A1H_M	14 kDa	0.998436	3	33%	Q8CGP6	Hist1h2ah	Histone H2	20.6451	22.11734	20.2852	21.92949	21.20398	21.83807	-1.25021	-2.02814	-0.47227	21.33653	-3.82507	-2.51847	0.006901	0.032606	-1	-1
186	sp|P62242	RS8_MOUS	24 kDa	0.999717	10	100%	P62242	Rps8	40S riboson	26.12299	25.33812	26.36842	24.02069	25.86389	24.09249	1.634663	0.617928	2.651397	25.3011	3.836817	-2.51838	0.006963	0.032784	1	1
1251.2	sp|Q9JKP5	MBNL1_M	37 kDa	0.996815	2	100%	Q9JKP5	Mbnl1	Muscleblin	17.19217	18.18926	15.52598	18.93352	16.80841	18.13649	-1.9109	-3.10584	-0.71596	17.46431	-3.81631	-2.54582	0.007145	0.033449	-1	-1
1017	sp|Q921T2	TOIP1_MO	67 kDa	0.999769	2	100%	Q921T2	Tor1aip1	Torsin-1A-i	19.02487	20.18072	16.56918	20.30915	18.27851	20.41865	-2.34532	-3.81243	-0.87821	19.13018	-3.81497	-2.54762	0.007157	0.033449	-1	-1
1100	sp|O88741	GDAP1_M	41 kDa	0.998964	2	100%	O88741	Gdap1	Ganglioside	16.57023	17.09956	15.5506	17.64949	15.95305	17.3945	-1.35656	-2.20697	-0.50614	16.7029	-3.8019	-2.55774	0.007195	0.033449	-1	-1
811	sp|Q62186	SSRD_MOU	19 kDa	0.999444	3	100%	Q62186	Ssr4	Translocon	23.63197	22.07744	23.67203	21.73563	23.51822	22.88704	1.374038	0.51262	2.235455	22.92039	3.802587	-2.55813	0.007203	0.033449	1	1
638	sp|P01633	KV5A1_MO	16 kDa	0.999406	4	50%	P01633	Igk-V19-17	Ig kappa ch	22.52496	24.83582	24.19079	26.06789	22.61157	24.9458	-2.17406	-3.53917	-0.80895	24.19614	-3.80061	-2.56687	0.007288	0.03373	-1	-1
637	sp|P01869	IGH1M_M	43 kDa	0.999409	4	100%	P01869	Ighg1	Ig gamma-1	25.16993	27.35553	25.73048	27.52137	24.17904	26.22533	-2.00759	-3.27374	-0.74144	26.03028	-3.7839	-2.58932	0.007444	0.034334	-1	-1
378	sp|P62259	1433E_MO	29 kDa	0.998499	7	71%	P62259	Ywhae	14-3-3 prot	21.92258	23.55076	22.10954	24.49489	21.71562	22.83284	-1.71025	-2.7925	-0.62799	22.77104	-3.77119	-2.60643	0.007565	0.034773	-1	-1
1014	sp|P12961	7B2_MOUS	24 kDa	0.999876	2	100%	P12961	Scg5	Neuroendo	18.97902	17.09026	18.34253	15.80426	19.94018	17.59953	2.255898	0.82642	3.685375	17.9593	3.766108	-2.61326	0.007614	0.034782	1	1
740	sp|Q9Z108	STAU1_MO	54 kDa	0.998308	4	100%	Q9Z108	Stau1	Double-stra	20.90502	19.94529	21.08713	19.76023	20.53657	19.60812	1.071692	0.391062	1.752321	20.30706	3.747658	-2.62327	0.007618	0.034782	1	1
88	sp|Q8CHC4	SYNJ1_MO	173 kDa	0.999829	16	100%	Q8CHC4	Synj1	Synaptojan	23.53908	21.82992	22.44211	21.43725	23.00188	21.69697	1.339644	0.488383	2.190904	22.32454	3.750784	-2.62676	0.007678	0.034939	1	1
159	sp|P62908	RS3_MOUS	27 kDa	0.999714	11	100%	P62908	Rps3	40S riboson	26.7488	25.49396	26.55744	25.63845	26.84899	25.94945	1.024457	0.369736	1.679178	26.20618	3.724245	-2.65514	0.007851	0.035516	1	1
1187	sp|Q8VHE0	SEC63_MO	88 kDa	0.998093	2	100%	Q8VHE0	Sec63	Translocati	19.45419	20.33196	19.3614	20.70331	19.32853	20.18215	-1.02443	-1.67926	-0.36961	19.89359	-3.72358	-2.65604	0.007858	0.035516	-1	-1
56	sp|Q8CG16	C1RA_MOU	80 kDa	0.999755	19	100%	Q8CG16	C1ra	Compleme	26.88471	27.83345	26.95398	28.23979	26.72597	27.65979	-1.05612	-1.73272	-0.37952	27.38295	-3.71519	-2.66749	0.007943	0.035732	-1	-1
694	tr|A0A140	A0A140T8	13 kDa	0.998985	4	50%	A0A140T8	Igkv6-15	Immunogl	21.18154	23.5582	22.15962	23.61855	20.17522	22.6342	-2.09819	-3.44013	-0.75626	22.22122	-3.73134	-2.66019	0.007958	0.035732	-1	-1
566	sp|Q4FK66	PR38A_MO	37 kDa	0.998592	5	100%	Q4FK66	Prpf38a	Pre-mRNA-	21.39541	18.91557	20.33887	19.80857	21.57198	19.67731	1.634938	0.582709	2.687168	20.28462	3.708016	-2.69176	0.008198	0.036481	1	1
406	sp|P14869	RLA0_MOU	34 kDa	0.999608	6	100%	P14869	Rplp0	60S acidic r	22.43835	21.56258	23.14436	21.87303	22.72625	21.48823	1.128374	0.39992	1.856827	22.20547	3.686818	-2.70623	0.008239	0.036481	1	1
679.1	sp|P02301	H3C_MOUS	15 kDa	0.999062	4	100%	P02301	H3f3c	Histone H3	26.84963	25.79016	26.23383	25.38899	27.05354	24.9619	1.331984	0.472108	2.191861	26.04634	3.69281	-2.70671	0.008286	0.036481	1	1
679.2	sp|P68433	H31_MOUS	15 kDa	0.999062	4	100%	P68433	Hist1h3i	Histone H3	26.84963	25.79016	26.23383	25.38899	27.05354	24.9619	1.331984	0.472108	2.191861	26.04634	3.69281	-2.70671	0.008286	0.036481	1	1
679.3	sp|P84228	H32_MOUS	15 kDa	0.999062	4	100%	P84228	Hist2h3c2	Histone H3	26.84963	25.79016	26.23383	25.38899	27.05354	24.9619	1.331984	0.472108	2.191861	26.04634	3.69281	-2.70671	0.008286	0.036481	1	1
679.4	sp|P84244	H33_MOUS	15 kDa	0.999062	4	100%	P84244	H3f3b	Histone H3	26.84963	25.79016	26.23383	25.38899	27.05354	24.9619	1.331984	0.472108	2.191861	26.04634	3.69281	-2.70671	0.008286	0.036481	1	1
208	sp|Q02257	PLAK_MOU	82 kDa	0.999314	10	100%	Q02257	Jup	Junction pl	20.71432	22.87278	21.67919	22.86479	22.06726	23.68831	-1.65504	-2.7244	-0.58568	22.31444	-3.69347	-2.71149	0.008352	0.036654	-1	-1
385	sp|P17427	AP2A2_MO	104 kDa	0.997759	7	57%	P17427	Ap2a2	AP-2 comp	21.497	19.59318	21.49084	19.78374	20.31035	19.56154	1.45324	0.512366	2.394115	20.37278	3.686009	-2.72162	0.008433	0.03672	1	1
239	sp|Q9QWK	CD5L_MOU	39 kDa	0.999386	9	100%	Q9QWK4	Cd5l	CD5 antige	22.73384	24.67661	22.84612	24.54785	21.72721	23.40758	-1.77496	-2.92517	-0.62474	23.3232	-3.68264	-2.7262	0.008469	0.03672	-1	-1
242	sp|Q9Z0H8	CLIP2_MO	116 kDa	0.999156	9	100%	Q9Z0H8	Clip2	CAP-Gly do	21.51879	22.86324	20.35985	22.89537	22.03308	23.31907	-1.72199	-2.83807	-0.60591	22.1649	-3.68201	-2.72705	0.008476	0.03672	-1	-1
304	sp|P19001	K1C19_MO	45 kDa	0.994115	8	25%	P19001	Krt19	Keratin, typ	21.29072	22.47524	22.15137	23.41253	21.4512	23.52047	-1.50498	-2.48047	-0.5295	22.38359	-3.68183	-2.7273	0.008478	0.03672	-1	-1
793	sp|Q9CWZ	RBM8A_M	20 kDa	0.999607	3	100%	Q9CWZ3	Rbm8a	RNA-bindin	24.88031	25.75978	24.53114	25.6741	24.92557	26.41514	-1.17066	-1.93145	-0.40988	25.36434	-3.66244	-2.73961	0.008503	0.03672	-1	-1
677	sp|Q80WC	UBN2_MOU	142 kDa	0.999085	4	100%	Q80WC1	Ubn2	Ubinuclein	19.17213	13.26706	17.38307	5.625675	21.08303	14.01973	8.241925	2.886949	13.5969	15.09178	3.673007	-2.73929	0.008574	0.036911	1	1
195	sp|O54941	SMCE1_M	47 kDa	0.999567	10	100%	O54941	Smarce1	SWI/SNF-re	24.90503	23.47545	23.76077	22.6846	25.01228	22.45604	1.687331	0.587594	2.787067	23.71569	3.661527	-2.75491	0.008702	0.03734	1	1
486	sp|P61358	RL27_MOU	16 kDa	0.999784	5	100%	P61358	Rpl27	60S riboson	24.21536	22.86925	24.38481	23.44313	24.006	23.12912	1.054886	0.36499	1.744781	23.67461	3.639342	-2.77132	0.008762	0.037479	1	1
523	sp|O88712	CTBP1_MO	48 kDa	0.99931	5	100%	O88712	Ctbp1	C-terminal-	21.15973	22.87995	21.98129	22.82075	21.88324	22.78585	-1.1541	-1.90951	-0.39869	22.2518	-3.6363	-2.7755	0.008796	0.037509	-1	-1
701	sp|Q60737	CSK21_MO	45 kDa	0.998885	4	100%	Q60737	Csnk2a1	Casein kina	21.30618	23.54571	20.38929	21.82659	21.0032	22.80043	-1.82469	-3.02029	-0.62908	21.8119	-3.6421	-2.7814	0.008922	0.037925	-1	-1
439	sp|Q6NZF1	ZC11A_MO	86 kDa	0.999213	6	100%	Q6NZF1	Zc3h11a	Zinc finger	24.71797	22.96025	23.5417	22.63279	25.27682	22.80947	1.711325	0.589156	2.833494	23.6565	3.639359	-2.78514	0.008953	0.03794	1	1
1011	sp|Q9CQQ	AT5F1_MO	29 kDa	0.994424	3	100%	Q9CQQ7	Atp5pb	ATP syntha	22.55471	24.70787	22.84626	24.27014	21.49252	23.37996	-1.82149	-3.01905	-0.62393	23.20858	-3.62979	-2.7982	0.009065	0.038024	-1	-1
391	sp|Q8R570	SNP47_MO	47 kDa	0.999813	6	100%	Q8R570	Snap47	Synaptoson	23.19089	24.21361	23.0016	24.28519	22.91097	23.72572	-1.04035	-1.7258	-0.35491	23.55466	-3.6125	-2.80826	0.009073	0.038024	-1	-1
1047.1	sp|P01642	KV5A9_MO	13 kDa	0.999377	2	100%	P01642	Gm10881	Ig kappa ch	17.57796	19.35847	18.21446	19.68604	NA	19.06195	-1.47261	-2.41715	-0.52807	18.77978	-3.85033	-2.59557	0.009085	0.038024	-1	-1
1047.2	tr|A0A140	A0A140T8	13 kDa	0.999377	2	100%	A0A140T8	Igkv5-48	Protein Igk	17.57796	19.35847	18.21446	19.68604	NA	19.06195	-1.47261	-2.41715	-0.52807	18.77978	-3.85033	-2.59557	0.009085	0.038024	-1	-1
508	sp|Q8K0E8	FIBB_MOU	55 kDa	0.999491	5	100%	Q8K0E8	Fgb	Fibrinogen	22.61871	20.39334	21.63383	21.20591	22.10401	20.67346	1.361281	0.463388	2.259174	21.43821	3.614243	-2.81408	0.00917	0.038262	1	1
831	sp|Q0VBL3	RBM15_M	106 kDa	0.999348	3	100%	Q0VBL3	Rbm15	RNA-bindin	22.4191	20.36229	20.53938	18.73121	21.96914	18.78609	2.34934	0.798737	3.899944	20.46787	3.615728	-2.81743	0.009231	0.038395	1	1
1010	tr|A0A075	A0A075B5	13 kDa	0.99458	3	33%	A0A075B5	Ighv1-37	Immunogl	18.8997	22.01298	19.21375	19.69044	18.23639	21.6541	-2.33589	-3.88314	-0.78865	19.95123	-3.60283	-2.83508	0.009386	0.038921	-1	-1
184	sp|Q8CFG8	CS1B_MOU	77 kDa	0.999751	10	10%	Q8CFG8	C1sb	Compleme	21.15259	22.72476	21.61556	22.43485	20.79505	22.0608	-1.21907	-2.02888	-0.40926	21.79727	-3.58315	-2.849	0.009431	0.038989	-1	-1
571	tr|A0A075	A0A075B5	13 kDa	0.998299	5	20%	A0A075B5	Ighv6-3	Immunogl	19.66521	21.26084	19.86692	21.61582	18.50265	20.43951	-1.76046	-2.92964	-0.59129	20.22516	-3.59333	-2.8481	0.009502	0.039092	-1	-1
921	sp|Q9WVG	MS3L1_M	60 kDa	0.998387	3	100%	Q9WVG9	Msl3	Male-specif	18.51269	20.48919	19.83753	22.12256	19.09067	20.74367	-1.97151	-3.28119	-0.66183	20.13272	-3.59239	-2.84938	0.009513	0.039092	-1	-1
1271	sp|P55937	GOGA3_M	167 kDa	0.996086	2	100%	P55937	Golga3	Golgin subf	19.3429	20.73359	19.63075	20.40843	19.19605	20.16801	-1.04677	-1.7454	-0.34815	19.91329	-3.56625	-2.87213	0.009638	0.039485	-1	-1
198	sp|P46096	SYT1_MOU	47 kDa	0.999481	10	80%	P46096	Syt1	Synaptotag	24.23333	23.41788	24.31347	23.2686	24.4028	23.53072	0.9108	0.301588	1.520012	23.86113	3.558406	-2.88299	0.009738	0.039772	0	0
532	sp|Q6NVF9	CPSF6_MO	59 kDa	0.999221	5	100%	Q6NVF9	Cpsf6	Cleavage an	23.60424	22.58169	23.01529	21.44957	23.17718	21.52069	1.414919	0.466095	2.363744	22.55811	3.558742	-2.8956	0.009938	0.040469	1	1
112	sp|P06880	SOMA_MO	25 kDa	0.999893	14	100%	P06880	Gh1	Somatotro	30.46798	29.19401	31.5654	30.12217	31.10104	29.50956	1.436224	0.468745	2.403703	30.32669	3.542676	-2.91772	0.010148	0.040629	1	1
780	sp|P62830	RL23_MOU	15 kDa	0.999855	3	100%	P62830	Rpl23	60S riboson	22.57372	24.72081	22.8465	24.40768	21.26138	23.37754	-1.94148	-3.25058	-0.63238	23.19794	-3.53924	-2.92246	0.010194	0.040629	-1	-1
8	sp|Q6P4T2	U520_MOU	245 kDa	0.999706	46	100%	Q6P4T2	Snrnp200	U5 small nu	26.14711	24.35526	25.19469	24.83136	26.5794	24.24679	1.495931	0.486917	2.504946	25.22577	3.538059	-2.92409	0.010209	0.040629	1	1
542.1	sp|P01787	HVM18_M	14 kDa	0.999129	5	60%	P01787	NA	Ig heavy ch	22.6187	24.57004	22.52038	24.12021	21.36085	23.18444	-1.79158	-3.00022	-0.58295	23.06244	-3.53747	-2.92489	0.010217	0.040629	-1	-1
542.2	sp|P01788	HVM19_M	14 kDa	0.999129	5	60%	P01788	NA	Ig heavy ch	22.6187	24.57004	22.52038	24.12021	21.36085	23.18444	-1.79158	-3.00022	-0.58295	23.06244	-3.53747	-2.92489	0.010217	0.040629	-1	-1
542.3	sp|P01790	HVM21_M	14 kDa	0.999129	5	60%	P01790	NA	Ig heavy ch	22.6187	24.57004	22.52038	24.12021	21.36085	23.18444	-1.79158	-3.00022	-0.58295	23.06244	-3.53747	-2.92489	0.010217	0.040629	-1	-1
542.4	sp|P01791	HVM22_M	14 kDa	0.999129	5	60%	P01791	NA	Ig heavy ch	22.6187	24.57004	22.52038	24.12021	21.36085	23.18444	-1.79158	-3.00022	-0.58295	23.06244	-3.53747	-2.92489	0.010217	0.040629	-1	-1
542.5	sp|P01794	HVM25_M	14 kDa	0.999129	5	60%	P01794	NA	Ig heavy ch	22.6187	24.57004	22.52038	24.12021	21.36085	23.18444	-1.79158	-3.00022	-0.58295	23.06244	-3.53747	-2.92489	0.010217	0.040629	-1	-1
800	sp|Q9CQR5	ZMAT5_M	20 kDa	0.999518	3	100%	Q9CQR5	Zmat5	Zinc finger	22.62015	24.90333	23.50593	25.25033	21.88105	23.76511	-1.97054	-3.30254	-0.63855	23.65432	-3.53048	-2.93453	0.010311	0.040881	-1	-1
19	sp|Q8BTI8	SRRM2_M	295 kDa	0.999729	29	100%	Q8BTI8	Srrm2	Serine/argi	25.50006	24.34192	25.24684	24.57461	26.15066	24.54117	1.146622	0.369694	1.923551	25.05921	3.512693	-2.94644	0.010341	0.040881	1	1
1178	sp|Q93092	TALDO_MO	37 kDa	0.998161	2	100%	Q93092	Taldo1	Transaldola	22.04436	21.23042	22.13229	20.93387	21.7499	20.87381	0.962817	0.30772	1.617914	21.49411	3.498153	-2.96668	0.010541	0.041552	0	0
343	sp|P68254	1433T_MO	28 kDa	0.99937	7	71%	P68254	Ywhaq	14-3-3 prot	22.78802	21.55871	22.96714	21.86973	22.28267	21.25707	1.11744	0.350156	1.884724	22.12056	3.46632	-3.01109	0.010994	0.043027	1	1
324	tr|A0A0B4	A0A0B4J1	13 kDa	0.999571	7	100%	A0A0B4J1	Igkv1-135	Immunogl	23.71496	25.78531	24.76285	26.71331	23.1627	25.08425	-1.98079	-3.33927	-0.6223	24.87056	-3.47964	-3.00484	0.01102	0.043027	-1	-1
419	sp|P01801	HVM32_M	13 kDa	0.999484	6	17%	P01801	NA	Ig heavy ch	20.15813	21.63836	20.46698	22.09655	19.21493	20.92708	-1.60732	-2.70982	-0.50482	20.75034	-3.47917	-3.00549	0.011027	0.043027	-1	-1
459	sp|Q569Z5	DDX46_M	117 kDa	0.998922	6	100%	Q569Z5	Ddx46	Probable A	20.75107	21.78378	20.94048	21.49856	20.50994	21.75951	-0.94679	-1.59763	-0.29595	21.20722	-3.46243	-3.01652	0.011051	0.043027	0	0
832.5	tr|A0A140	A0A140T8	13 kDa	0.999312	3	33%	A0A140T8	Igkv3-4	Immunogl	23.40676	22.58069	23.36312	21.70752	23.16474	22.31016	1.112086	0.347275	1.876898	22.7555	3.460868	-3.0187	0.011074	0.043027	1	1
657	sp|Q9QUI0	RHOA_MO	22 kDa	0.999264	4	25%	Q9QUI0	Rhoa	Transformi	19.77692	17.76794	20.3614	18.64351	19.62169	19.0265	1.440683	0.448794	2.432572	19.19966	3.46622	-3.02345	0.011216	0.043453	1	1
874	sp|Q91WJ	FUBP1_MO	69 kDa	0.998969	3	67%	Q91WJ8	Fubp1	Far upstrea	19.29235	21.24632	20.78671	22.4224	19.608	21.36592	-1.78252	-3.01449	-0.55055	20.78695	-3.45291	-3.04193	0.011414	0.044056	-1	-1
402	sp|G5E829	AT2B1_MO	135 kDa	0.999659	6	100%	G5E829	Atp2b1	Plasma me	21.27672	24.26736	21.99073	23.04389	22.80853	24.06834	-1.76787	-2.99024	-0.5455	22.90926	-3.45142	-3.04401	0.011436	0.044056	-1	-1
271	sp|Q9CXY6	ILF2_MOUS	43 kDa	0.999564	8	100%	Q9CXY6	Ilf2	Interleukin	22.558	21.56879	22.00128	21.26223	22.60948	20.60511	1.24421	0.382711	2.105708	21.76748	3.442967	-3.05101	0.011475	0.04408	1	1
916.11	sp|Q8CGP	H2A1F_MO	14 kDa	0.998436	3	33%	Q8CGP5	Hist1h2af	Histone H2	21.70109	19.53103	20.35261	19.26683	20.57721	19.59098	1.414022	0.431684	2.396359	20.16996	3.435153	-3.06662	0.011684	0.044755	1	1
1290	sp|Q80XN	BDH_MOUS	38 kDa	0.995005	2	100%	Q80XN0	Bdh1	D-beta-hyd	15.86316	18.82183	16.37755	17.56597	16.71688	17.37783	-1.60269	-2.71967	-0.4857	17.12054	-3.42415	-3.08195	0.011855	0.045282	-1	-1
1009	sp|Q9EPN1	NBEA_MOU	327 kDa	0.994717	3	33%	Q9EPN1	Nbea	Neurobeac	18.7965	22.44869	19.08408	20.13113	19.50881	23.80586	-2.99877	-5.09631	-0.90123	20.62918	-3.4118	-3.09916	0.01205	0.045898	-1	-1
1062	sp|Q0P5V2	SOBP_MOU	92 kDa	0.999243	2	100%	Q0P5V2	Sobp	Sine oculis-	18.26344	19.82591	18.13364	18.75869	16.92038	20.4005	-1.88921	-3.21155	-0.56687	18.7171	-3.40948	-3.10239	0.012087	0.04591	-1	-1
1210	sp|Q8R326	PSPC1_MO	59 kDa	0.99779	2	100%	Q8R326	Pspc1	Paraspeckle	19.0285	17.98653	18.45406	16.52011	18.40393	16.43615	1.647899	0.491239	2.80456	17.80488	3.399973	-3.11566	0.01224	0.046361	1	1
1286	sp|P42232	STA5B_MO	90 kDa	0.995294	2	100%	P42232	Stat5b	Signal trans	18.58149	19.83637	17.8418	19.0329	18.54645	19.94087	-1.28014	-2.18298	-0.37729	18.96331	-3.38014	-3.13885	0.012472	0.047066	-1	-1
786	tr|A0A075	A0A075B6	11 kDa	0.999674	3	33%	A0A075B6	Ighv1-78	Immunogl	21.42798	18.9608	20.20741	20.11767	21.60376	18.59672	1.854654	0.546871	3.162437	20.15239	3.384371	-3.13746	0.012495	0.047066	1	1
74	sp|Q9Z1R2	BAG6_MOU	121 kDa	0.999644	17	100%	Q9Z1R2	Bag6	Large proli	24.82119	26.31045	25.63808	25.99043	24.92573	26.42931	-1.11506	-1.90333	-0.32679	25.68587	-3.36687	-3.15063	0.012552	0.047147	-1	-1
382	sp|Q99PU8	DHX30_M	137 kDa	0.99821	7	100%	Q99PU8	Dhx30	ATP-depen	20.1292	18.1591	19.60263	17.52874	19.21206	18.85229	1.467917	0.430368	2.505465	18.914	3.376321	-3.14872	0.01263	0.047309	1	1
1058	sp|Q5SUR0	PUR4_MOU	145 kDa	0.999277	2	100%	Q5SUR0	Pfas	Phosphorib	18.74609	17.12656	18.66624	12.98109	19.0687	16.2118	3.387192	0.985407	5.788977	17.13341	3.365552	-3.16379	0.012811	0.047575	1	1
319	sp|P63011	RAB3A_MO	25 kDa	0.999637	7	71%	P63011	Rab3a	Ras-related	24.38117	23.10715	24.12305	23.33827	23.86707	23.14006	0.928602	0.269147	1.588057	23.65946	3.351545	-3.17223	0.012812	0.047575	0	0
832.1	sp|P01665	KV3AD_MO	12 kDa	0.999312	3	33%	P01665	NA	Ig kappa ch	24.16156	26.14511	25.44105	27.12187	23.31243	25.84295	-2.06496	-3.53051	-0.59942	25.3375	-3.36251	-3.16805	0.012863	0.047575	-1	-1
832.2	sp|P01666	KV3AE_MO	12 kDa	0.999312	3	33%	P01666	NA	Ig kappa ch	24.16156	26.14511	25.44105	27.12187	23.31243	25.84295	-2.06496	-3.53051	-0.59942	25.3375	-3.36251	-3.16805	0.012863	0.047575	-1	-1
206	tr|E9Q1Z0	E9Q1Z0_M	58 kDa	0.99933	10	10%	E9Q1Z0	Krt90	Keratin 90	25.17991	26.3187	25.04602	26.05911	25.64295	26.84973	-1.11955	-1.91582	-0.32329	25.8494	-3.34648	-3.17938	0.012899	0.047575	-1	-1
785	sp|Q6PDM	MSL1_MOU	67 kDa	0.999675	3	100%	Q6PDM1	Msl1	Male-specif	19.42529	20.31994	19.49852	20.25393	18.72494	21.48599	-1.47037	-2.51479	-0.42595	19.95144	-3.35972	-3.17195	0.012911	0.047575	-1	-1
1230	sp|Q9Z1T1	AP3B1_MO	123 kDa	0.997522	2	100%	Q9Z1T1	Ap3b1	AP-3 comp	18.18484	18.58027	17.71707	20.40209	17.76675	21.07671	-2.13014	-3.6484	-0.61188	18.95462	-3.34822	-3.18808	0.01311	0.047997	-1	-1
379	sp|Q9QZQ8	H2AY_MOU	40 kDa	0.998402	7	100%	Q9QZQ8	H2afy	Core histor	23.14974	22.48891	22.8055	21.74509	23.35294	21.52854	1.18188	0.337856	2.025905	22.51178	3.335293	-3.19822	0.013161	0.047997	1	1
410	sp|P14115	RL27A_MO	17 kDa	0.999587	6	100%	P14115	Rpl27a	60S riboson	25.0773	24.35798	25.51709	24.37646	25.06241	24.16433	0.91934	0.260875	1.577805	24.75926	3.323105	-3.21241	0.013311	0.047997	0	0
627.1	sp|P10853	H2B1F_MO	14 kDa	0.999468	4	100%	P10853	Hist1h2bn	Histone H2	27.60808	26.65783	26.88069	25.35859	27.35263	25.46878	1.452069	0.411749	2.492388	26.55443	3.330972	-3.21228	0.013415	0.047997	1	1
627.2	sp|P10854	H2B1M_M	14 kDa	0.999468	4	100%	P10854	Hist1h2bm	Histone H2	27.60808	26.65783	26.88069	25.35859	27.35263	25.46878	1.452069	0.411749	2.492388	26.55443	3.330972	-3.21228	0.013415	0.047997	1	1
627.3	sp|Q6ZWY	H2B1C_MO	14 kDa	0.999468	4	100%	Q6ZWY9	Hist1h2bg	Histone H2	27.60808	26.65783	26.88069	25.35859	27.35263	25.46878	1.452069	0.411749	2.492388	26.55443	3.330972	-3.21228	0.013415	0.047997	1	1
627.4	sp|Q8CGP1	H2B1K_MO	14 kDa	0.999468	4	100%	Q8CGP1	Hist1h2bk	Histone H2	27.60808	26.65783	26.88069	25.35859	27.35263	25.46878	1.452069	0.411749	2.492388	26.55443	3.330972	-3.21228	0.013415	0.047997	1	1
627.5	sp|Q8CGP2	H2B1P_MO	14 kDa	0.999468	4	100%	Q8CGP2	Hist1h2bp	Histone H2	27.60808	26.65783	26.88069	25.35859	27.35263	25.46878	1.452069	0.411749	2.492388	26.55443	3.330972	-3.21228	0.013415	0.047997	1	1
627.6	sp|Q64475	H2B1B_MO	14 kDa	0.999468	4	100%	Q64475	Hist1h2bb	Histone H2	27.60808	26.65783	26.88069	25.35859	27.35263	25.46878	1.452069	0.411749	2.492388	26.55443	3.330972	-3.21228	0.013415	0.047997	1	1
627.7	sp|Q64478	H2B1H_MO	14 kDa	0.999468	4	100%	Q64478	Hist1h2bh	Histone H2	27.60808	26.65783	26.88069	25.35859	27.35263	25.46878	1.452069	0.411749	2.492388	26.55443	3.330972	-3.21228	0.013415	0.047997	1	1
627.8	sp|Q64525	H2B2B_MO	14 kDa	0.999468	4	100%	Q64525	Hist2h2bb	Histone H2	27.60808	26.65783	26.88069	25.35859	27.35263	25.46878	1.452069	0.411749	2.492388	26.55443	3.330972	-3.21228	0.013415	0.047997	1	1
782	sp|Q9JKK1	STX6_MOU	29 kDa	0.999725	3	100%	Q9JKK1	Stx6	Syntaxin-6	21.04916	19.62749	19.84004	19.1177	21.38642	18.66022	1.623402	0.459569	2.787235	19.94684	3.328788	-3.21535	0.013454	0.04801	1	1
1063.1	tr|A0A0A6	A0A0A6YX	42 kDa	0.999242	2	100%	A0A0A6YX	Igha	Immunogl	21.34396	22.84424	22.27622	22.72817	20.9602	22.90266	-1.29823	-2.22974	-0.36672	22.17591	-3.32593	-3.21936	0.013506	0.048067	-1	-1
66	sp|Q62261	SPTB2_MO	274 kDa	0.999538	18	94%	Q62261	Sptbn1	Spectrin be	23.05836	24.39588	23.34265	24.07382	23.25027	23.91527	-0.91123	-1.57009	-0.25237	23.67271	-3.29182	-3.25671	0.013884	0.04917	0	0
1291	sp|Q3TDD	PPR21_MO	88 kDa	0.994795	2	100%	Q3TDD9	Ppp1r21	Protein pho	19.72781	21.30293	20.62817	22.03197	20.77996	21.64606	-1.28168	-2.20723	-0.35613	21.01948	-3.30404	-3.24936	0.013888	0.04917	-1	-1
220	sp|Q9R0H5	K2C71_MO	57 kDa	0.99783	10	30%	Q9R0H5	Krt71	Keratin, typ	20.71627	26.99334	22.32913	25.93382	25.47407	26.84644	-3.75138	-6.46264	-1.04012	24.71551	-3.30195	-3.25309	0.013945	0.049244	-1	-1
982	sp|Q8BJM7	TYW1_MO	82 kDa	0.99719	3	100%	Q8BJM7	Tyw1	S-adenosyl-	18.54003	20.42801	19.3526	19.63303	18.22847	19.99479	-1.31158	-2.26077	-0.36239	19.36282	-3.29757	-3.25925	0.014027	0.049405	-1	-1
771	sp|P21126	UBL4A_MO	18 kDa	0.996743	4	100%	P21126	Ubl4a	Ubiquitin-l	19.11274	20.47747	18.74828	20.26007	19.71726	20.14981	-1.10302	-1.90404	-0.30201	19.74427	-3.27752	-3.27699	0.014155	0.049718	-1	-1
1064	sp|Q31125	S39A7_MO	51 kDa	0.99924	2	100%	Q31125	Slc39a7	Zinc transp	23.65166	23.03033	23.93482	22.70736	23.55237	22.70352	0.899217	0.245815	1.552619	23.26334	3.275553	-3.27979	0.014193	0.049718	0	0
457	sp|P97762	RP9_MOUS	25 kDa	0.998954	6	100%	P97762	rp9	Retinitis pi	19.1707	20.75125	20.26302	21.88216	19.48914	20.76678	-1.49244	-2.57597	-0.40891	20.38717	-3.28706	-3.27407	0.014226	0.049718	-1	-1
962	sp|P20357	MTAP2_M	199 kDa	0.997708	3	100%	P20357	Map2	Microtubu	19.9232	19.08077	19.92721	17.82748	19.56382	18.68972	1.272088	0.347611	2.196565	19.1687	3.283124	-3.27884	0.014283	0.049787	1	1
575	sp|P23116	EIF3A_MO	162 kDa	0.998125	5	100%	P23116	Eif3a	Eukaryotic	21.03761	22.24267	21.56231	23.9039	20.93486	22.34992	-1.6539	-2.85715	-0.45065	22.00521	-3.28022	-3.28371	0.014357	0.049919	-1	-1
216	sp|Q91YR7	PRP6_MOU	107 kDa	0.998848	10	100%	Q91YR7	Prpf6	Pre-mRNA-	22.78952	23.57737	22.44745	23.3747	22.79357	23.79134	-0.90429	-1.56473	-0.24385	23.12899	-3.25891	-3.30343	0.014516	0.050316	0	0
650	sp|Q9Z1Q9	SYVC_MOU	140 kDa	0.999336	4	100%	Q9Z1Q9	Vars	Valine--tRN	21.27092	22.27429	21.92173	22.78289	21.34803	22.54732	-1.02127	-1.7675	-0.27504	22.0242	-3.2574	-3.30558	0.014546	0.050316	-1	-1

1144	sp|Q61464	ZN638_MO	218 kDa	0.998506	2	100%	Q61464	Znf638	Zinc finger	18.73794	20.15227	17.33851	19.61122	19.09184	20.96889	-1.8547	-3.21253	-0.49687	19.31678	-3.25971	-3.31265	0.014758	0.05092	-1	-1
1003	sp|Q8BX10	PGAM5_M	32 kDa	0.995432	3	100%	Q8BX10	Pgam5	Serine/thre	20.61479	19.25946	21.03657	20.16825	20.80334	19.797	1.076663	0.285382	1.867945	20.2799	3.238543	-3.3324	0.014922	0.051357	1	1
1119.2	sp|C0HK80	ARXS2_MO	20 kDa	0.998757	2	100%	C0HK80	Arxes2	Adipocyte-	22.44177	21.28251	22.2516	21.36534	21.8199	20.95819	0.969081	0.255958	1.682204	21.68655	3.234421	-3.33827	0.015006	0.051514	0	0
898	sp|P02468	LAMC1_MO	177 kDa	0.998706	3	100%	P02468	Lamc1	Laminin su	17.36052	21.12654	18.73089	18.83599	18.10737	21.03991	-2.26789	-3.93636	-0.59941	19.2002	-3.24379	-3.33516	0.015077	0.051539	-1	-1
218	sp|Q3UV17	K22O_MOU	63 kDa	0.998045	10	10%	Q3UV17	Krt76	Keratin, typ	24.17855	27.2646	26.69078	28.41119	26.21784	28.21609	-2.26823	-3.93761	-0.59886	26.82984	-3.24254	-3.33692	0.015103	0.051539	-1	-1
613	sp|P62918	RL8_MOUS	28 kDa	0.99957	4	100%	P62918	Rpl8	60S ribosor	23.91971	22.96292	25.35297	23.62357	24.659	23.16656	1.392877	0.367367	2.418387	23.94745	3.241333	-3.33863	0.015127	0.051539	1	1
730	sp|Q9CYN9	RENR_MOU	39 kDa	0.998453	4	100%	Q9CYN9	Atp6ap2	Renin recep	21.33995	20.01686	20.3396	19.81024	21.11157	19.49734	1.15556	0.302683	2.008438	20.35259	3.229248	-3.35088	0.015248	0.05179	1	1
945	sp|Q62311	TAF6_MOU	73 kDa	0.997951	3	100%	Q62311	Taf6	Transcripti	21.53924	22.48376	20.59371	21.84033	21.20001	22.75409	-1.2484	-2.16977	-0.32704	21.73519	-3.23258	-3.3499	0.015277	0.05179	-1	-1
484	tr|A0A0B4	A0A0B4J1J	11 kDa	0.999849	5	20%	A0A0B4J1J	Ighv1-82	Immunoglo	22.72671	25.18137	24.90635	26.44601	23.22512	25.42486	-2.06469	-3.59068	-0.53869	24.65174	-3.22888	-3.35625	0.015383	0.051893	-1	-1
1185	sp|O35963	RB33B_MO	26 kDa	0.998107	2	100%	O35963	Rab33b	Ras-related	18.61365	21.92673	19.41745	24.03444	19.06121	20.10867	-2.99251	-5.20601	-0.77901	20.52702	-3.22632	-3.35988	0.015436	0.051893	-1	-1
987	sp|P98195	ATP9B_MO	129 kDa	0.996941	3	33%	P98195	Atp9b	Probable p	22.32723	21.06901	22.25345	21.34965	21.88627	21.36956	0.892905	0.231489	1.55432	21.70919	3.213155	-3.36858	0.015446	0.051893	0	0
1075	sp|Q61033	LAP2A_MO	75 kDa	0.999163	2	100%	Q61033	Tmpo	Lamina-ass	17.217	16.49091	18.70633	16.7869	17.75882	16.16876	1.411857	0.366835	2.456878	17.18812	3.224155	-3.36294	0.015481	0.051893	1	1
617	tr|A0A0A6	A0A0A6YV	13 kDa	0.999549	4	50%	A0A0A6YV	Ighv1-23	Immunoglo	22.47945	24.95764	24.66166	26.19997	23.08218	25.1787	-2.03767	-3.54674	-0.5286	24.4266	-3.22237	-3.36548	0.015519	0.051893	-1	-1
408	sp|P25444	RS2_MOUS	31 kDa	0.99959	6	100%	P25444	Rps2	40S ribosor	24.90052	23.55319	25.20167	24.13509	24.62236	24.04582	0.996815	0.257387	1.736242	24.40978	3.208629	-3.37504	0.015542	0.051893	0	0
641	tr|A0A075	A0A075B5	11 kDa	0.999388	4	25%	A0A075B5	Ighv1-12	Immunoglo	22.45846	24.94402	24.64641	26.18745	23.05892	25.15689	-2.04153	-3.55472	-0.52833	24.40869	-3.21967	-3.3693	0.015575	0.051893	-1	-1
1037	sp|Q922P9	GLYR1_MO	60 kDa	0.999433	2	100%	Q922P9	Glyr1	Putative ox	23.28803	25.61854	24.96253	26.65848	22.73394	25.13884	-2.14379	-3.74066	-0.54692	24.73339	-3.20378	-3.39183	0.015913	0.052759	-1	-1
1038	sp|Q9R099	TBL2_MOU	50 kDa	0.999431	2	100%	Q9R099	Tbl2	Transducin	23.28803	25.61854	24.96253	26.65848	22.73394	25.13884	-2.14379	-3.74066	-0.54692	24.73339	-3.20378	-3.39183	0.015913	0.052759	-1	-1
602	tr|A0A075	A0A075B5	11 kDa	0.999702	4	75%	A0A075B5	Igkv8-28	Immunoglo	22.93043	24.69853	24.26432	25.97369	23.40683	24.76228	-1.61097	-2.81214	-0.4098	24.33935	-3.20061	-3.39633	0.015982	0.052842	-1	-1
916.10	sp|Q8BFU2	H2A3_MOU	14 kDa	0.998436	3	33%	Q8BFU2	Hist3h2a	Histone H2	21.75737	23.81681	22.88746	23.58622	22.97043	24.67067	-1.48615	-2.59528	-0.37702	23.28149	-3.19765	-3.40052	0.016046	0.052842	-1	-1
1109	sp|O35465	FKBP8_MO	44 kDa	0.998852	2	100%	O35465	Fkbp8	Peptidyl-pr	20.08854	22.03708	20.31717	21.40816	19.89263	20.68473	-1.27721	-2.23107	-0.32335	20.73805	-3.19542	-3.40369	0.016094	0.052842	-1	-1
1110	sp|P45591	COF2_MOU	19 kDa	0.998832	2	50%	P45591	Cfl2	Cofilin-2	20.08854	22.03708	20.31717	21.40816	19.89263	20.68473	-1.27721	-2.23107	-0.32335	20.73805	-3.19542	-3.40369	0.016094	0.052842	-1	-1
258	sp|P04104	K2C1_MOU	66 kDa	0.999759	8	75%	P04104	Krt1	Keratin, typ	26.89041	28.2927	27.75588	28.70276	27.80706	28.99446	-1.17885	-2.06193	-0.29577	28.07388	-3.1824	-3.41836	0.016272	0.053296	-1	-1
269	sp|Q3TTY5	K22E_MOU	71 kDa	0.999598	8	75%	Q3TTY5	Krt2	Keratin, typ	27.42303	27.91043	27.46561	28.48307	26.55497	28.61685	-1.18891	-2.0817	-0.29613	27.74233	-3.17466	-3.42938	0.016444	0.053627	-1	-1
182	sp|Q8BTM8	FLNA_MOU	281 kDa	0.999873	10	90%	Q8BTM8	Flna	Filamin-A	21.44473	22.20546	21.50788	22.2146	20.82194	22.18872	-0.94474	-1.65478	-0.23471	21.73056	-3.1669	-3.43466	0.016452	0.053627	0	0
490	sp|P18527	HVM56_M	11 kDa	0.999709	5	20%	P18527	NA	Ig heavy ch	22.83483	25.09691	23.69477	25.12349	22.16545	23.72507	-1.75014	-3.06584	-0.43444	23.77342	-3.17443	-3.43351	0.016558	0.053715	-1	-1
500	tr|A0A075	A0A075B5	13 kDa	0.999544	5	40%	A0A075B5	Ighv5-17	Immunoglo	22.83483	25.09691	23.69477	25.12349	22.16545	23.72507	-1.75014	-3.06584	-0.43444	23.77342	-3.17443	-3.43351	0.016558	0.053715	-1	-1
1245	sp|Q8BVI5	STX16_MO	37 kDa	0.997032	2	100%	Q8BVI5	Stx16	Syntaxin-16	20.14193	21.24357	20.01671	20.61781	19.15516	20.9595	-1.16902	-2.05031	-0.28774	20.35578	-3.1623	-3.447	0.016723	0.054119	-1	-1
466	sp|P46638	RB11B_MO	24 kDa	0.998356	6	100%	P46638	Rab11b	Ras-related	22.58047	26.01932	23.02968	23.33919	22.75785	25.93625	-2.30892	-4.04978	-0.56806	23.94379	-3.16516	-3.44669	0.016768	0.054128	-1	-1
1136	sp|P80316	TCPE_MOU	60 kDa	0.99857	2	100%	P80316	Cct5	T-complex	22.13264	23.04299	22.06049	22.687	22.01365	22.94274	-0.82198	-1.44279	-0.20116	22.47992	-3.15137	-3.45691	0.016805	0.054128	0	0
79	sp|Q8CAQ8	MIC60_MO	84 kDa	0.999498	17	100%	Q8CAQ8	Immt	MICOS com	23.8663	24.79439	24.22846	24.78502	23.67939	24.78143	-0.86223	-1.51852	-0.20593	24.35583	-3.12696	-3.4919	0.017377	0.055837	0	0
398	sp|P62264	RS14_MOU	16 kDa	0.999699	6	100%	P62264	Rps14	40S ribosor	24.61183	23.89033	25.18527	23.92356	24.67504	23.99588	0.887456	0.211267	1.563646	24.38032	3.123774	-3.49647	0.017453	0.055949	0	0
1068	sp|Q9DCR2	AP3S1_MO	22 kDa	0.999217	2	100%	Q9DCR2	Ap3s1	AP-3 comp	21.64431	17.63543	19.06564	18.13848	21.87576	19.16112	2.550227	0.605097	4.495357	19.58679	3.128822	-3.49845	0.017617	0.056254	1	0
213	sp|Q8BMK4	CKAP4_MO	64 kDa	0.999127	10	100%	Q8BMK4	Ckap4	Cytoskeleto	20.95514	22.1632	21.41182	23.17518	21.88914	22.60386	-1.22871	-2.16607	-0.29136	22.03306	-3.12822	-3.49932	0.017631	0.056254	-1	0
360	sp|B2RY56	RBM25_M	100 kDa	0.999135	7	100%	B2RY56	Rbm25	RNA-bindir	22.1694	21.06942	22.01795	21.60517	22.17716	20.71115	0.992924	0.232444	1.753404	21.62504	3.107628	-3.51966	0.017845	0.05676	0	0
916.13	sp|Q8CGP7	H2A1K_M	14 kDa	0.998436	3	33%	Q8CGP7	Hist1h2ak	Histone H2	17.49933	19.37395	18.35155	18.9447	17.68718	NA	-1.31331	-2.30287	-0.32374	18.37134	-3.28478	-3.31376	0.017878	0.05676	-1	0
844	sp|Q9JII5|	DAZP1_MO	43 kDa	0.999212	3	100%	Q9JII5	Dazap1	DAZ-associ	20.35194	21.99044	19.6933	21.21492	18.77912	20.58635	-1.65578	-2.9237	-0.38787	20.43601	-3.11648	-3.51607	0.017916	0.05676	-1	0
678	sp|Q9CZX8	RS19_MOU	16 kDa	0.999069	4	100%	Q9CZX8	Rps19	40S ribosor	21.1663	21.7784	21.68498	23.73654	20.76809	23.36253	-1.7527	-3.09593	-0.40948	22.08281	-3.11393	-3.5197	0.017978	0.056825	-1	0
161	sp|P97351	RS3A_MOU	30 kDa	0.9996	11	100%	P97351	Rps3a	40S ribosor	23.82999	23.23401	24.11161	22.54046	23.52044	22.68903	0.999514	0.231542	1.767486	23.32092	3.097734	-3.53388	0.018089	0.057033	0	0
806	sp|Q61598	GDIB_MOU	51 kDa	0.999474	3	67%	Q61598	Gdi2	Rab GDP di	22.89698	21.89981	22.61068	22.17156	22.89627	21.83406	0.832838	0.192612	1.473064	22.38489	3.096195	-3.53609	0.018128	0.057033	0	0
1081	sp|Q3TJZ6	FA98A_MO	55 kDa	0.999131	2	100%	Q3TJZ6	Fam98a	Protein FAI	26.18688	23.61623	25.0779	23.13685	26.61336	25.17665	1.982799	0.452619	3.51298	24.96798	3.092337	-3.55056	0.018516	0.058038	1	0
849	sp|Q6ZPJ3	UBE2O_MO	141 kDa	0.999172	3	100%	Q6ZPJ3	Ube2o	(E3-indeper	21.22238	20.05973	20.16797	19.47169	21.10626	18.95748	1.335902	0.304726	2.367077	20.16425	3.091666	-3.55152	0.018533	0.058038	1	0
609	sp|P01639	KV5A7_MO	14 kDa	0.999647	4	50%	P01639	Gm5571	Ig kappa ch	20.90958	22.87286	21.92188	23.28731	20.92352	21.9382	-1.4478	-2.57159	-0.324	21.97556	-3.07447	-3.57612	0.018974	0.059115	-1	0
364	sp|Q9R0N5	SYT5_MOU	43 kDa	0.999051	7	71%	Q9R0N5	Syt5	Synaptotag	21.4152	20.62117	21.62429	21.05952	21.80493	20.41685	0.915628	0.204058	1.627197	21.15699	3.062684	-3.58433	0.018985	0.059115	0	0
373	sp|P11983	TCPA_MOU	60 kDa	0.998923	7	86%	P11983	Tcp1	T-complex	22.03277	23.32591	23.27968	27.16754	22.5978	26.63184	-3.07168	-5.45696	-0.6864	24.17259	-3.07318	-3.57797	0.019007	0.059115	-1	0
1141	sp|Q8BTX9	HSDL1_MO	37 kDa	0.998519	2	100%	Q8BTX9	Hsdl1	Inactive hy	19.83488	24.78124	17.29591	24.24659	19.97972	20.23571	-4.05101	-7.20072	-0.9013	21.06234	-3.06933	-3.58348	0.019108	0.059291	-1	0
810	sp|Q9R112	SQOR_MOU	50 kDa	0.99945	3	100%	Q9R112	Sqor	Sulfide:qui	20.86886	21.47694	20.97894	21.78368	20.24894	21.66869	-0.94419	-1.68116	-0.20721	21.17101	-3.04935	-3.60355	0.019338	0.059868	0	0
530	sp|Q9WVR	FXR2_MOU	74 kDa	0.999225	5	60%	Q9WVR4	Fxr2	Fragile X m	21.13431	22.50088	20.4116	21.58402	21.16765	22.02335	-1.13157	-2.01667	-0.24646	21.4703	-3.04774	-3.61104	0.019562	0.060255	-1	0
306.1	sp|P01863	GCAA_MOU	36 kDa	0.999867	7	86%	P01863	Ighg	Ig gamma-2	27.36892	28.74938	28.56541	29.53275	27.71947	29.09629	-1.24154	-2.21267	-0.2704	28.50537	-3.05092	-3.60985	0.019596	0.060255	-1	0
306.2	sp|P01865	GCAM_MO	44 kDa	0.999867	7	86%	P01865	Igh-1a	Ig gamma-2	27.36892	28.74938	28.56541	29.53275	27.71947	29.09629	-1.24154	-2.21267	-0.2704	28.50537	-3.05092	-3.60985	0.019596	0.060255	-1	0
157	sp|Q9D0E1	HNRPM_M	78 kDa	0.999898	11	100%	Q9D0E1	Hnrnpm	Heterogene	23.81454	23.01013	23.27448	22.16757	23.58094	22.45551	1.012249	0.218618	1.80588	23.05053	3.035775	-3.62314	0.019704	0.060451	1	0
90	sp|P07901	HS90A_MO	85 kDa	0.99979	16	56%	P07901	Hsp90aa1	Heat shock	23.48696	24.244	23.47314	24.19927	22.50916	24.20911	-1.06104	-1.89443	-0.22765	23.68694	-3.03044	-3.63099	0.019856	0.060779	-1	0
1205	sp|Q921F4	HNRLL_MO	64 kDa	0.997883	2	100%	Q921F4	Hnrnpll	Heterogene	19.87285	20.43901	19.90515	20.61592	19.68001	20.79096	-0.79596	-1.42435	-0.16757	20.21732	-3.01482	-3.65339	0.020285	0.061898	0	0
1097	sp|Q91YP0	L2HDH_M	51 kDa	0.998996	2	100%	Q91YP0	L2hgdh	L-2-hydroxy	19.68227	20.11841	19.74544	21.1738	19.49732	20.75919	-1.04212	-1.86511	-0.21913	20.16274	-3.01402	-3.65471	0.020313	0.061898	-1	0
323	sp|P35980	RL18_MOU	22 kDa	0.999574	7	100%	P35980	Rpl18	60S ribosor	25.86043	25.50647	26.16436	24.52851	25.80528	24.252	1.181033	0.245946	2.11612	25.35284	3.01412	-3.66267	0.020613	0.062671	1	0
931	sp|Q3TXS7	PSMD1_M	106 kDa	0.998211	3	100%	Q3TXS7	Psmd1	26S protea	21.5493	20.55774	22.5378	20.84932	21.24385	20.13167	1.264069	0.261182	2.266957	21.14495	3.00794	-3.67155	0.020789	0.063065	1	0
905	tr|G3X8U3	G3X8U3_M	39 kDa	0.998612	3	100%	G3X8U3	2210016F1	Queuosine	22.50088	21.59274	22.86089	22.04512	22.57885	21.81241	0.830117	0.166165	1.494069	22.23182	2.975797	-3.70985	0.021415	0.064819	0	0
63	sp|Q9ESU6	BRD4_MOU	156 kDa	0.999719	18	100%	Q9ESU6	Brd4	Bromodom	25.06452	23.9049	23.7937	22.30319	25.51418	22.88037	1.761315	0.351272	3.171358	23.91014	2.980954	-3.71037	0.021578	0.065165	1	0
322	sp|Q3TDQ1	STT3B_MO	93 kDa	0.999583	7	86%	Q3TDQ1	Stt3b	Dolichyl-di	23.29999	22.48761	22.96804	22.32553	23.13935	22.27421	0.773343	0.150134	1.396552	22.74912	2.953514	-3.74213	0.02209	0.066566	0	0
656	sp|O70404	VAMP8_M	11 kDa	0.999267	4	100%	O70404	Vamp8	Vesicle-ass	25.10347	23.49243	24.69223	23.33267	24.8207	24.45309	1.112737	0.215775	2.009699	24.31577	2.957416	-3.74119	0.022164	0.066639	1	0
1137	sp|Q9CYG7	TOM34_M	34 kDa	0.998561	2	100%	Q9CYG7	Tomm34	Mitochond	19.79804	20.41211	19.44821	20.48668	19.50637	20.21103	-0.78573	-1.42265	-0.14882	19.97708	-2.93626	-3.76716	0.022629	0.067396	0	0
353	tr|A0A2R8	A0A2R8VH	58 kDa	0.999252	7	29%	A0A2R8VH	Gm5478	Predicted p	25.24538	25.88246	25.78906	26.98654	24.1742	26.94941	-1.53659	-2.78182	-0.29136	25.83784	-2.94483	-3.76243	0.022683	0.067396	-1	0
1088	sp|P27612	PLAP_MOU	87 kDa	0.999063	2	100%	P27612	Plaa	Phospholip	18.74677	21.07718	18.88185	19.2794	18.94684	21.83052	-1.87054	-3.3868	-0.35429	19.79376	-2.94406	-3.76353	0.022707	0.067396	-1	0
929	tr|A0A075	A0A075B5	13 kDa	0.998258	3	33%	A0A075B5	Ighv14-2	Immunoglo	19.51302	20.26388	19.08072	20.14489	19.59203	20.20793	-0.81031	-1.46773	-0.15289	19.80041	-2.93363	-3.77097	0.022712	0.067396	0	0
101	sp|P01872	IGHM_MOU	50 kDa	0.999648	15	100%	P01872	Ighm	Immunoglo	27.19116	28.49345	27.11103	28.4493	26.34852	27.41747	-1.2365	-2.23901	-0.234	27.50182	-2.94347	-3.76439	0.022726	0.067396	-1	0
267	sp|P62806	H4_MOUSE	11 kDa	0.999599	8	100%	P62806	Hist4h4	Histone H4	28.31263	27.3346	27.27886	25.73448	28.04698	26.0879	1.49383	0.282503	2.705158	27.13258	2.943	-3.76506	0.022741	0.067396	1	0
695	sp|Q8QZT1	THIL_MOUS	45 kDa	0.99896	4	100%	Q8QZT1	Acat1	Acetyl-CoA	20.63042	21.06243	20.81015	22.70028	20.11609	23.79761	-2.00122	-3.62438	-0.37806	21.5195	-2.94229	-3.76609	0.022763	0.067396	-1	0
1002.2	sp|Q80VQ0	AL3B1_MO	52 kDa	0.995458	3	100%	Q80VQ0	Aldh3b1	Aldehyde d	20.88722	19.67256	23.96654	17.32303	21.72996	20.42034	3.05593	0.571543	5.540317	20.66661	2.935451	-3.77595	0.02298	0.067424	1	0
481	tr|A0A075	A0A075B5	11 kDa	0.999942	5	40%	A0A075B5	Ighv1-43	Immunoglo	22.81384	25.18867	25.11803	26.68872	23.29034	25.45492	-2.0367	-3.69291	-0.38049	24.75909	-2.93469	-3.77706	0.023005	0.067424	-1	0
84	sp|Q922U2	K2C5_MOU	62 kDa	0.998902	17	47%	Q922U2	Krt5	Keratin, typ	23.34071	25.41956	25.3252	26.50245	24.71066	26.81444	-1.78662	-3.2417	-0.33154	25.35217	-2.9302	-3.78353	0.023148	0.067424	-1	0
729	sp|P62334	PRS10_MO	44 kDa	0.998479	4	100%	P62334	Psmc6	26S protea	22.05284	23.49345	21.79907	27.08325	21.94051	23.56295	-2.78241	-5.04856	-0.51626	23.32201	-2.9301	-3.78368	0.023151	0.067424	-1	0
453	sp|Q9Z110	P5CS_MOU	87 kDa	0.998979	6	100%	Q9Z110	Aldh18a1	Delta-1-pyr	20.49007	21.0054	20.21388	21.84483	20.12265	20.96502	-0.99621	-1.80828	-0.18414	20.77364	-2.91998	-3.79092	0.023155	0.067424	0	0
583	sp|P84091	AP2M1_M	50 kDa	0.997211	5	100%	P84091	Ap2m1	AP-2 comp	23.81037	22.32336	23.57002	22.19383	22.90525	22.73735	1.010369	0.186655	1.834082	22.92336	2.919616	-3.79146	0.023167	0.067424	1	0
464	sp|Q6ZPR4	KCNT1_M	138 kDa	0.998579	6	100%	Q6ZPR4	Kcnt1	Potassium	19.77841	17.82771	18.95494	16.91251	20.32247	18.98341	1.777396	0.328975	3.225818	18.79657	2.928464	-3.78604	0.023204	0.067424	1	0
1163	sp|P00405	COX2_MOU	26 kDa	0.998301	2	100%	P00405	Mtco2	Cytochrom	16.45857	18.43037	18.35068	18.63351	16.46537	19.78843	-1.85923	-3.37446	-0.344	18.02115	-2.92823	-3.78638	0.023212	0.067424	-1	0
587	sp|P47857	PFKAM_M	85 kDa	0.996794	5	60%	P47857	Pfkm	ATP-depen	20.18599	20.98899	19.55696	20.47379	19.05935	20.59214	-1.08421	-1.96856	-0.19987	20.14287	-2.92271	-3.79137	0.023259	0.067424	-1	0
751.2	sp|P01656	KV3A3_MO	12 kDa	0.997764	4	25%	P01656	NA	Ig kappa ch	22.20678	21.61101	22.09707	21.46517	22.32532	21.23552	0.772497	0.141384	1.40361	21.82348	2.913337	-3.80044	0.023366	0.067424	0	0
352	sp|P43274	H14_MOUS	22 kDa	0.999273	7	29%	P43274	Hist1h1e	Histone H1	24.95628	24.40787	25.13317	24.51737	25.59897	24.15171	0.870488	0.159246	1.58173	24.79423	2.91304	-3.80087	0.023375	0.067424	0	0
1053	sp|Q9D1D4	TMEDA_M	25 kDa	0.999297	2	100%	Q9D1D4	Tmed10	Transmemb	21.72646	22.43562	21.72431	22.37141	21.81843	22.82564	-0.78782	-1.43156	-0.14409	22.15031	-2.91287	-3.80112	0.023381	0.067424	0	0
974	sp|O88935	SYN1_MOU	74 kDa	0.997544	3	67%	O88935	Syn1	Synapsin-1	NA	18.52299	16.46347	19.55697	18.02984	19.07375	-1.80458	-3.26115	-0.34801	18.32941	-3.07097	-3.59725	0.02342	0.067424	-1	0
636	tr|J3QK03	J3QK03_M	11 kDa	0.99941	4	50%	J3QK03	Ighv6-5	Immunoglo	19.24566	20.88704	19.94511	21.38176	18.5662	19.94772	-1.48651	-2.70306	-0.26997	19.99558	-2.91602	-3.80401	0.023608	0.067824	-1	0
880	sp|Q9CW0	SMC3_MOU	142 kDa	0.998919	3	100%	Q9CW03	Smc3	Structural	21.30276	20.49528	22.70691	21.01078	22.34286	21.09511	1.25045	0.220391	2.280509	21.49228	2.897043	-3.83143	0.02424	0.06949	1	0
858	sp|Q61545	EWS_MOUS	68 kDa	0.99907	3	100%	Q61545	Ewsr1	RNA-bindir	18.34569	19.49539	18.86731	19.66019	17.73537	19.06338	-1.0902	-1.98993	-0.19046	18.86122	-2.88858	-3.84081	0.024393	0.069783	-1	0
770	sp|Q9WUA	SYFB_MOUS	66 kDa	0.996919	4	100%	Q9WUA2	Farsb	Phenylalan	21.28569	19.8359	22.92422	20.56716	21.21848	20.46881	1.518838	0.263461	2.774214	21.05004	2.887274	-3.84556	0.024571	0.070145	1	0
626	sp|Q9D1M	SEC13_MO	36 kDa	0.999481	4	100%	Q9D1M0	Sec13	Protein SEC	22.36954	20.35718	21.84705	20.1058	21.82239	21.61808	1.319306	0.227413	2.4112	21.35334	2.883474	-3.85105	0.024702	0.070369	1	0
600	tr|A0A140	A0A140T8	13 kDa	0.999708	4	25%	A0A140T8	Igkv6-25	Immunoglo	20.74847	22.99187	22.60429	23.77756	20.8654	22.56049	-1.70392	-3.11668	-0.29116	22.25801	-2.87826	-3.8586	0.024882	0.070733	-1	0
590	sp|Q6P542	ABCF1_MO	95 kDa	0.999828	4	100%	Q6P542	Abcf1	ATP-bindin	20.71838	17.61124	18.74682	17.92869	18.71617	17.72178	1.639884	0.27567	3.004098	18.57385	2.868674	-3.87247	0.025217	0.071535	1	0
127	sp|Q8BHS8	SYBU_MOU	73 kDa	0.999745	13	85%	Q8BHS8	Sybu	Syntabulin	24.71025	23.33591	23.35876	20.98313	24.62368	22.5543	1.939781	0.322297	3.557264	23.26101	2.861959	-3.88219	0.025454	0.072058	1	0
539	sp|P61620	S61A1_MO	52 kDa	0.999172	5	100%	P61620	Sec61a1	Protein tra	22.0348	21.37862	22.38587	21.40309	21.90202	21.20375	0.779075	0.125891	1.43226	21.71802	2.838864	-3.90879	0.025941	0.073283	0	0
470	sp|P84084	ARF5_MOU	21 kDa	0.998134	6	50%	P84084	Arf5	ADP-ribosy	21.94885	23.92619	20.69065	21.74001	20.42162	22.96983	-1.85831	-3.42507	-0.29154	21.94953	-2.83051	-3.92777	0.026598	0.07494	-1	0
412	sp|Q9ER58	TICN2_MO	47 kDa	0.999573	6	100%	Q9ER58	Spock2	Testican-2	21.94076	24.06247	21.67806	22.17376	21.94692	23.67778	-1.44943	-2.67238	-0.22647	22.57996	-2.82836	-3.93087	0.026678	0.07494	-1	0
862	sp|Q9CQC7	NDUB4_M	15 kDa	0.999046	3	100%	Q9CQC7	Ndufb4	NADH dehy	20.37357	21.85819	21.24276	21.69905	20.57247	21.43936	-0.93594	-1.72628	-0.14559	21.19757	-2.81858	-3.93836	0.026693	0.07494	0	0
190	sp|P07356	ANXA2_M	39 kDa	0.999661	10	100%	P07356	Anxa2	Annexin A2	23.67308	24.04217	23.4087	24.61763	23.63475	24.476	-0.80642	-1.48961	-0.12324	23.97539	-2.80947	-3.95165	0.027038	0.075752	0	0
1036	sp|Q9WUQ	PREB_MOU	45 kDa	0.999441	2	100%	Q9WUQ2	Preb	Prolactin re	15.84759	18.96055	17.54844	18.72038	16.36642	17.26072	-1.7264	-3.19625	-0.25655	17.45068	-2.80298	-3.96771	0.027644	0.077288	-1	0
1287	sp|P26638	SYSC_MOU	58 kDa	0.995287	2	100%	P26638	Sars	Serine--tRN	17.36066	16.74451	17.39737	16.11356	16.99057	15.13956	1.250322	0.183512	2.317132	16.62437	2.796955	-3.97645	0.027878	0.077783	1	0
225	sp|Q60930	VDAC2_M	32 kDa	0.999813	9	100%	Q60930	Vdac2	Voltage-dep	23.99638	23.4321	24.1305	23.07336	23.76874	23.10344	0.762237	0.110134	1.41434	23.58409	2.782112	-3.99158	0.028104	0.078142	0	0
1170	sp|P30275	KCRU_MOU	47 kDa	0.998235	2	100%	P30275	Ckmt1	Creatine kir	24.52315	23.90961	24.81755	23.8433	24.40754	23.7831	0.737407	0.106444	1.36837	24.21404	2.78166	-3.99224	0.028122	0.078142	0	0
1147	sp|P63325	RS10_MOU	19 kDa	0.998503	2	100%	P63325	Rps10	40S ribosor	22.95405	21.94229	23.53032	22.16273	22.45879	21.94641	0.96391	0.138123	1.789697	22.4991	2.778373	-3.99716	0.02826	0.078299	0	0
687	tr|A0A075	A0A075B5	11 kDa	0.999008	4	50%	A0A075B5	Igkv8-27	Immunoglo	18.70096	21.10714	19.27954	21.14212	20.64572	24.14502	-2.58935	-4.80702	-0.37168	20.83675	-2.78641	-3.99176	0.028294	0.078299	-1	0
469	sp|Q68FL6	SYMC_MOU	101 kDa	0.998165	6	100%	Q68FL6	Mars	Methionine	19.51202	21.3058	20.22308	20.2157	19.70669	21.35491	-1.14487	-2.12608	-0.16366	20.38637	-2.7845	-3.99454	0.02837	0.07835	-1	0
141	sp|Q9DBG3	AP2B1_MO	105 kDa	0.997806	13	46%	Q9DBG3	Ap2b1	AP-2 comp	23.15598	21.37204	23.25725	21.8094	21.75426	20.86725	1.372933	0.193299	2.552567	22.03603	2.77749	-4.00472	0.028651	0.078964	1	0
114	sp|O70624	MYOC_MOU	55 kDa	0.999859	14	100%	O70624	Myoc	Myocilin	24.06301	23.32106	23.72197	22.85188	23.66256	22.87739	0.799072	0.111022	1.487121	23.41631	2.764183	-4.01776	0.028826	0.079287	0	0
951	sp|Q8K1M6	DNM1L_M	83 kDa	0.997901	3	100%	Q8K1M6	Dnm1l	Dynamin-1	20.22039	18.34894	20.94661	19.36239	20.20156	19.83991	1.27244	0.176408	2.368472	19.81997	2.770543	-4.01481	0.028932	0.079416	1	0
14	sp|O88737	BSN_MOUS	419 kDa	0.9995	35	100%	O88737	Bsn	Protein bas	23.04352	25.00809	21.45565	25.0599	24.43219	24.75986	-1.96549	-3.66361	-0.26738	23.95987	-2.76221	-4.02692	0.029273	0.080049	-1	0
392	sp|Q3UHK1	MYCT_MOU	69 kDa	0.999778	6	100%	Q3UHK1	Slc2a13	Proton myo	22.47252	23.45209	22.9104	23.64619	22.39145	23.13867	-0.82086	-1.53059	-0.11112	23.00189	-2.75279	-4.03441	0.029295	0.080049	0	0
845	tr|Q80ZM5	Q80ZM5_M	20 kDa	0.999209	3	100%	Q80ZM5	H1fx	H1 histone	21.13084	22.67627	21.70975	21.97843	21.35144	22.18377	-0.88215	-1.64517	-0.11913	21.83842	-2.75173	-4.03597	0.02934	0.080049	0	0
299	sp|Q11011	PSA_MOUS	103 kDa	0.998545	8	100%	Q11011	Npepps	Puromycin	21.21294	23.02534	22.30321	23.37755	21.85324	22.3792	-1.13757	-2.12269	-0.15244	22.35858	-2.75573	-4.03633	0.029541	0.08039	-1	0
526	sp|Q9ER69	FL2D_MOU	44 kDa	0.999285	5	100%	Q9ER69	Wtap	Pre-mRNA-	18.5583	19.12888	17.4902	19.38718	18.68982	19.43559	-1.07111	-1.99903	-0.14319	18.78166	-2.75467	-4.03784	0.029583	0.08039	-1	0
1165	sp|P01027	CO3_MOUS	186 kDa	0.998285	2	100%	P01027	C3	Compleme	20.40933	19.88472	20.16579	19.19634	20.19402	18.81703	0.957015	0.126382	1.787647	19.77787	2.742406	-4.0497	0.029737	0.080406	0	0
789	sp|Q9D384	U1SBP_MO	29 kDa	0.999646	3	100%	Q9D384	Snrnp35	U11/U12 sr	18.22159	19.78875	16.79628	19.43194	19.07134	19.60016	-1.57722	-2.94613	-0.2083	18.81834	-2.74957	-4.04529	0.029798	0.080406	-1	0
399	sp|Q3B7Z2	OSBP1_MO	89 kDa	0.999671	6	100%	Q3B7Z2	Osbp	Oxysterol-b	22.60444	20.02101	21.7037	21.84946	22.58158	20.61257	1.468892	0.193964	2.743819	21.56212	2.749507	-4.04539	0.029801	0.080406	1	0
414	sp|Q9Z1Z0	USO1_MOU	107 kDa	0.999567	6	100%	Q9Z1Z0	Uso1	General ves	22.22573	22.81753	22.1343	22.71304	21.71459	22.8099	-0.75529	-1.41134	-0.09923	22.40251	-2.74013	-4.05291	0.029826	0.080406	0	0
662	sp|P63024	VAMP3_M	11 kDa	0.999219	4	50%	P63024	Vamp3	Vesicle-ass	22.11123	20.79044	20.86191	19.33916	21.63336	20.36416	1.370912	0.178921	2.562904	20.85004	2.744651	-4.05245	0.030005	0.080729	1	0
463	sp|Q9DCD2	SYF1_MOU	100 kDa	0.998711	6	100%	Q9DCD2	Xab2	Pre-mRNA-	20.7791	20.10436	20.67987	20.2501	21.74623	19.77823	1.024172	0.131058	1.917286	20.55631	2.733759	-4.06589	0.030322	0.081418	1	0
278	sp|Q8VDJ3	VIGLN_MO	142 kDa	0.99943	8	100%	Q8VDJ3	Hdlbp	Vigilin	21.88987	21.0612	21.71129	20.23761	21.73413	21.2303	0.935393	0.117732	1.753054	21.31073	2.72297	-4.07812	0.030569	0.081781	0	0
981	sp|Q6NSU3	GL8D1_MO	42 kDa	0.997254	3	100%	Q6NSU3	Glt8d1	Glycosyltra	18.38342	16.25019	15.80608	13.96295	19.09967	15.22438	2.617219	0.330419	4.904018	16.45445	2.731257	-4.07193	0.030577	0.081781	1	0
236	sp|Q00PI9	HNRL2_MO	85 kDa	0.999487	9	100%	Q00PI9	Hnrnpul2	Heterogene	23.46053	21.80072	23.27767	21.56276	22.95646	22.90901	1.140717	0.141444	2.13999	22.66119	2.724233	-4.08215	0.030882	0.082417	1	0
836.1	sp|P01638	KV5A6_MO	13 kDa	0.999277	3	100%	P01638	NA	Ig kappa ch	17.54744	14.73293	15.63094	16.03972	17.94353	13.2049	2.381449	0.29432	4.468578	15.84991	2.722969	-4.08399	0.030937	0.082417	1	0
1102	sp|Q09163	DLK1_MOU	41 kDa	0.998951	2	100%	Q09163	Dlk1	Protein del	21.01135	19.91239	20.76942	20.2948	21.63649	20.45027	0.919935	0.112343	1.727526	20.67912	2.71136	-4.0951	0.031077	0.082629	0	0
798.3	tr|A0A2I3	A0A2I3BRL	42 kDa	0.999544	3	100%	A0A2I3BRL	Gm7324	Predicted g	21.97355	22.70747	21.68837	22.54516	22.18523	22.85399	-0.75315	-1.41625	-0.09006	22.32563	-2.70338	-4.10667	0.031425	0.08339	0	0
1270	sp|Q9CQW	YKT6_MOU	22 kDa	0.996185	2	100%	Q9CQW1	Ykt6	Synaptobre	15.1606	16.13552	15.10459	16.04965	15.83482	16.82286	-0.96934	-1.82324	-0.11544	15.85134	-2.7056	-4.10645	0.031522	0.083484	0	0
294	sp|Q4G0F8	UBN1_MOU	122 kDa	0.998983	8	100%	Q4G0F8	Ubn1	Ubinuclein	22.94501	23.40816	20.99325	23.90496	22.96744	24.64749	-1.68497	-3.18058	-0.18935	23.14439	-2.68857	-4.13407	0.032478	0.085848	-1	0
462	sp|Q9Z1N5	DX39B_MO	49 kDa	0.998744	6	100%	Q9Z1N5	Ddx39b	Spliceosom	21.87734	24.82251	21.67497	22.01079	21.87744	24.41092	-1.93816	-3.66133	-0.21498	22.779	-2.68417	-4.14048	0.032681	0.086216	-1	0
207	sp|P02535	K1C10_MO	58 kDa	0.999318	10	80%	P02535	Krt10	Keratin, typ	28.48804	28.91114	28.41481	29.1065	27.67688	30.3615	-1.26647	-2.39342	-0.13953	28.82648	-2.68191	-4.14377	0.032785	0.086324	-1	0
147	sp|P70704	AT8A1_MO	131 kDa	0.999574	12	100%	P70704	Atp8a1	Phospholip	22.34894	23.84426	23.58151	23.71198	22.43669	24.00702	-1.06537	-2.01426	-0.11648	23.32173	-2.67937	-4.14746	0.032903	0.086466	-1	0
80	sp|Q3TEA8	HP1B3_MO	61 kDa	0.999402	17	100%	Q3TEA8	Hp1bp3	Heterochro	24.89401	23.54595	25.31159	24.88947	25.29301	23.61559	1.149199	0.123926	2.174472	24.5916	2.674892	-4.15399	0.033112	0.086848	1	0
813	sp|Q9CRB9	MIC19_MO	26 kDa	0.999433	3	100%	Q9CRB9	Chchd3	MICOS com	19.50863	21.93516	21.54366	21.97173	19.91339	21.48422	-1.47514	-2.79377	-0.15652	21.05947	-2.66971	-4.16154	0.033356	0.087319	-1	0
514	sp|P17751	TPIS_MOUS	32 kDa	0.999415	5	100%	P17751	Tpi1	Triosephos	21.1336	21.92856	20.82541	22.05441	21.35506	21.63712	-0.76867	-1.45694	-0.0804	21.48902	-2.65816	-4.17286	0.033516	0.087525	0	0
992	tr|A0A140	A0A140T8	13 kDa	0.996713	3	67%	A0A140T8	Igkv1-117	Immunoglo	20.8362	21.81629	21.39161	22.61264	21.5681	22.13768	-0.92357	-1.75132	-0.09583	21.72709	-2.65581	-4.17641	0.033636	0.087525	0	0
993	sp|Q8BP47	SYNC_MOU	64 kDa	0.99656	3	100%	Q8BP47	Nars	Asparagine	20.8362	21.81629	21.39161	22.61264	21.5681	22.13768	-0.92357	-1.75132	-0.09583	21.72709	-2.65581	-4.17641	0.033636	0.087525	0	0
955	sp|Q9DBP5	KCY_MOUS	22 kDa	0.99784	3	100%	Q9DBP5	Cmpk1	UMP-CMP	17.69648	16.82647	17.40337	14.87972	18.18634	11.29231	3.429234	0.354809	6.503659	16.04745	2.661851	-4.17299	0.03373	0.087525	1	0
124	sp|Q7TSC1	PRC2A_MO	229 kDa	0.999914	13	100%	Q7TSC1	Prrc2a	Protein PRI	21.8237	20.29848	21.32324	21.30351	21.97834	20.57564	0.982552	0.101322	1.863782	21.21715	2.658034	-4.17637	0.033758	0.087525	0	0
1272	sp|Q8K0C9	GMDS_MO	42 kDa	0.99604	2	100%	Q8K0C9	Gmds	GDP-mann	21.6135	20.78303	21.08911	20.67999	21.42575	19.87977	0.928523	0.094961	1.762084	20.91186	2.65141	-4.18284	0.033848	0.087591	0	0

731	sp|Q8CGZ0	CHERP_MO	106 kDa	0.998439	4	100%	Q8CGZ0	Cherp	Calcium ho	19.7326	18.91843	19.39718	18.98631	19.52252	18.5118	0.745254	0.075018	1.41549	19.17814	2.646533	-4.18989	0.034077	0.088017	0	0
854	sp|P32921	SYWC_MOU	54 kDa	0.999093	3	100%	P32921	Wars	Tryptophar	21.51485	22.31613	20.69197	22.03792	21.77243	22.54966	-0.97482	-1.8544	-0.09524	21.81383	-2.64207	-4.19968	0.034533	0.089023	0	0
957	sp|Q99P58	RB27B_MO	25 kDa	0.997785	3	33%	Q99P58	Rab27b	Ras-related	21.76208	21.26309	22.24039	20.23237	21.23287	20.50384	1.078678	0.103477	2.05388	21.20578	2.639661	-4.20533	0.034808	0.089564	1	0
781	tr|Q3U4B1	Q3U4B1_M	37 kDa	0.999788	3	67%	Q3U4B1	Ssbp4	Single strar	20.36239	19.84935	21.6409	19.64033	20.25046	19.25902	1.168347	0.111184	2.225511	20.16707	2.637428	-4.20858	0.034919	0.089678	1	0
980	sp|Q8BH04	PCKGM_M	71 kDa	0.99726	3	100%	Q8BH04	Pck2	Phosphoen	17.59617	18.48901	16.7916	19.04809	18.4791	19.05903	-1.24308	-2.36898	-0.11719	18.24383	-2.63483	-4.21238	0.035048	0.08984	-1	0
350	sp|Q61941	NNTM_MO	114 kDa	0.999294	7	100%	Q61941	Nnt	NAD(P) trar	23.46375	24.9691	24.41784	26.04697	24.91549	25.53374	-1.25091	-2.38495	-0.11687	24.89115	-2.63238	-4.21594	0.03517	0.089982	-1	0
1093.1	sp|P61327	MGN_MOU	17 kDa	0.999012	2	100%	P61327	Magoh	Protein ma	15.39886	19.18514	17.61647	19.42956	18.63677	19.17715	-2.04658	-3.90933	-0.18384	18.24066	-2.62196	-4.23113	0.035694	0.091152	-1	0
295	sp|Q9Z2K1	K1C16_MO	52 kDa	0.998923	8	38%	Q9Z2K1	Krt16	Keratin, typ	18.29305	20.6316	20.19063	20.98453	20.18094	22.0199	-1.65714	-3.16832	-0.14596	20.38344	-2.61694	-4.23845	0.03595	0.091631	-1	0
179	sp|Q04750	TOP1_MOU	91 kDa	0.998937	11	100%	Q04750	Top1	DNA topois	21.97346	23.4105	22.36539	22.74803	22.66678	23.59587	-0.91625	-1.75287	-0.07964	22.79334	-2.60727	-4.24783	0.036078	0.091631	0	0
651	sp|Q91W50	CSDE1_MO	89 kDa	0.999328	4	100%	Q91W50	Csde1	Cold shock	20.14475	18.86037	19.51494	19.47688	20.01891	18.62934	0.904005	0.078517	1.729492	19.44086	2.606645	-4.24842	0.036085	0.091631	0	0
301	tr|B2RT41	B2RT41_M	225 kDa	0.998007	8	100%	B2RT41	Zfc3h1	Zinc finger,	19.72281	20.72066	17.7719	20.22584	19.58492	20.47482	-1.44723	-2.77115	-0.12331	19.75016	-2.60872	-4.25044	0.036373	0.09219	-1	0
887	sp|Q9EPE9	AT131_MO	132 kDa	0.998839	3	100%	Q9EPE9	Atp13a1	Manganese	13.16176	18.05621	13.59603	14.18098	NA	17.3538	-3.15144	-6.02973	-0.27314	15.26976	-2.71397	-4.0743	0.036789	0.092959	-1	0
303	sp|Q9DCV7	K2C7_MOU	51 kDa	0.995589	8	38%	Q9DCV7	Krt7	Keratin, typ	20.06203	19.66068	20.01247	19.14906	20.61708	19.47183	0.803335	0.06582	1.54085	19.82886	2.592547	-4.26898	0.036813	0.092959	0	0
577	sp|Q9R0N0	GALK1_MO	42 kDa	0.99795	5	100%	Q9R0N0	Galk1	Galactokin	21.59296	22.36245	21.54921	22.47905	21.12872	21.83593	-0.80218	-1.54233	-0.06203	21.82472	-2.57959	-4.28797	0.037504	0.094439	0	0
827	sp|Q8BP67	RL24_MOU	18 kDa	0.999366	3	100%	Q8BP67	Rpl24	60S ribosor	20.71275	19.88765	20.2485	19.1527	20.53617	19.91277	0.848101	0.065378	1.630823	20.07509	2.578935	-4.28893	0.037539	0.094439	0	0
766	sp|Q8BX90	FND3A_MO	132 kDa	0.997145	4	100%	Q8BX90	Fndc3a	Fibronectir	18.10043	17.85575	15.54876	18.62786	15.42358	18.30464	-1.90516	-3.66505	-0.14527	17.31017	-2.58344	-4.28731	0.037706	0.094683	-1	0
290	sp|P42932	TCPQ_MOU	60 kDa	0.999068	8	100%	P42932	Cct8	T-complex	22.82982	24.0613	22.47278	23.20813	22.5006	23.17574	-0.88066	-1.6984	-0.06291	23.0414	-2.56336	-4.31185	0.038395	0.096212	0	0
1199	sp|Q8BML1	OACYL_MO	77 kDa	0.997952	2	100%	Q8BML2	Oacyl	O-acyltrans	14.46419	15.15861	11.69788	15.35234	14.51149	16.6196	-2.15233	-4.15124	-0.15342	14.63402	-2.5696	-4.30749	0.038456	0.096212	-1	0
984	sp|Q8CIE2	ZMIZ2_MO	97 kDa	0.997118	3	100%	Q8CIE2	Zmiz2	Zinc finger	20.73819	20.30062	19.56023	22.01002	19.40958	21.97877	-1.52714	-2.9498	-0.10447	20.66624	-2.56169	-4.31903	0.038893	0.097052	-1	0
9	sp|Q99PV0	PRP8_MOU	274 kDa	0.999619	45	100%	Q99PV0	Prpf8	Pre-mRNA-	26.51989	24.63566	25.40605	25.26912	26.96499	25.38132	1.201608	0.081871	2.321345	25.69617	2.560927	-4.32015	0.038935	0.097052	1	0
750.2	tr|A0A140	A0A140T8	13 kDa	0.997781	4	50%	A0A140T8	Igkv12-41	Protein Igk	20.09583	22.51047	21.24994	22.65159	22.41439	24.81552	-2.07247	-4.01044	-0.1345	22.28962	-2.55207	-4.33307	0.039431	0.097927	-1	0
751.1	sp|P01654	KV3A1_MO	12 kDa	0.997764	4	25%	P01654	NA	Ig kappa ch	20.09583	22.51047	21.24994	22.65159	22.41439	24.81552	-2.07247	-4.01044	-0.1345	22.28962	-2.55207	-4.33307	0.039431	0.097927	-1	0
144	sp|Q3UTJ2	SRBS2_MO	132 kDa	0.999691	12	100%	Q3UTJ2	Sorbs2	Sorbin and	23.3373	20.86913	21.87923	20.29638	23.36335	22.3805	1.677959	0.105736	3.250182	22.02098	2.546935	-4.34056	0.039721	0.098467	1	0
169	sp|Q99JR8	SMRD2_M	59 kDa	0.999372	11	91%	Q99JR8	Smarcd2	SWI/SNF-re	23.43968	20.97937	22.3396	22.52246	23.50122	21.8694	1.303092	0.0767	2.529483	22.44195	2.535692	-4.35696	0.040364	0.099878	1	0
1218	sp|O55022	PGRC1_MO	22 kDa	0.997686	2	50%	O55022	Pgrmc1	Membrane	18.78903	19.97469	15.9403	20.01927	18.39555	19.00039	-1.95649	-3.80505	-0.10792	18.68654	-2.52577	-4.37143	0.04094	0.10112	-1	0
187	sp|P10126	EF1A1_MO	50 kDa	0.999707	10	30%	P10126	Eef1a1	Elongation	24.32869	23.62159	24.18156	23.69618	24.27302	23.58216	0.627782	0.033005	1.222559	23.9472	2.512207	-4.38672	0.041317	0.101864	0	0
41	sp|Q9R0I7	YLPM1_M	155 kDa	0.999814	22	100%	Q9R0I7	Ylpm1	YLP motif-c	26.61066	24.99479	25.31427	25.07381	26.83904	25.33052	1.121616	0.053378	2.189854	25.69385	2.505688	-4.40073	0.042133	0.103689	1	0
1191.2	sp|Q9CQC9	SAR1B_MO	22 kDa	0.998052	2	100%	Q9CQC9	Sar1b	GTP-bindin	20.15253	21.06316	19.64633	20.38487	20.25306	21.82015	-1.07209	-2.09376	-0.05041	20.55335	-2.5042	-4.4029	0.042223	0.103722	-1	0
256	sp|Q99KI0	ACON_MO	85 kDa	0.99984	8	100%	Q99KI0	Aco2	Aconitate h	21.84682	23.09235	22.84626	22.97281	21.96035	23.19137	-0.8677	-1.69881	-0.03658	22.65166	-2.48502	-4.42663	0.042975	0.105378	0	0
1242	sp|P21278	GNA11_M	42 kDa	0.997167	2	100%	P21278	Gna11	Guanine nu	16.86335	14.97846	18.33561	17.22075	17.6544	13.02014	2.544668	0.102554	4.986782	16.34545	2.486656	-4.42848	0.043297	0.105893	1	0
794	sp|P01630	KV2A6_MO	12 kDa	0.999602	3	33%	P01630	NA	Ig kappa ch	21.1021	20.68125	21.18696	20.40725	21.12151	20.41317	0.636303	0.025267	1.247339	20.81871	2.478552	-4.43602	0.04337	0.105893	0	0
1280	sp|Q8K0L9	ZBT20_MO	81 kDa	0.995702	2	100%	Q8K0L9	Zbtb20	Zinc finger	20.26318	18.44949	22.24132	20.89245	21.43402	18.80873	1.929284	0.076287	3.782281	20.3482	2.48469	-4.43135	0.043419	0.105893	1	0
422	sp|O08538	ANGP1_M	58 kDa	0.999433	6	100%	O08538	Angpt1	Angiopoiet	21.66208	20.81513	22.21574	21.05985	21.15419	19.82284	1.111399	0.041083	2.181715	21.12164	2.478041	-4.44105	0.043835	0.106715	1	0
33	sp|Q99104	MYO5A_M	216 kDa	0.999369	24	100%	Q99104	Myo5a	Unconvent	23.16301	23.89178	23.11372	23.74498	23.36102	23.89655	-0.63186	-1.24114	-0.02257	23.52851	-2.46829	-4.45105	0.044016	0.106964	0	0
475	sp|Q80SY5	PR38B_MO	64 kDa	0.997437	6	100%	Q80SY5	Prpf38b	Pre-mRNA-	19.65407	21.97681	19.6132	20.7489	20.77621	20.90881	-1.19701	-2.35397	-0.04005	20.613	-2.46905	-4.45416	0.044404	0.107713	-1	0
624	sp|Q9QYH6	MAGD1_M	86 kDa	0.999491	4	100%	Q9QYH6	Maged1	Melanoma-	20.44559	18.32565	20.25479	19.83698	20.48935	19.70369	1.107803	0.035008	2.180599	19.84268	2.464316	-4.46106	0.044706	0.108018	1	0
668.1	sp|P19253	RL13A_MO	23 kDa	0.99914	4	100%	P19253	Rpl13a	60S ribosor	23.91459	23.18001	23.71477	23.09677	23.68246	23.16763	0.622468	0.019363	1.225572	23.45937	2.456548	-4.46825	0.044769	0.108018	0	0
668.2	tr|A0A1B0	A0A1B0GS	51 kDa	0.99914	4	100%	A0A1B0GS	Gm45713	Predicted g	23.91459	23.18001	23.71477	23.09677	23.68246	23.16763	0.622468	0.019363	1.225572	23.45937	2.456548	-4.46825	0.044769	0.108018	0	0
1142.2	tr|W4VSN7	W4VSN7_N	18 kDa	0.998518	2	100%	W4VSN7	Gm3550	60S ribosor	21.53802	19.7664	22.0227	20.84075	21.50734	21.23563	1.075095	0.031224	2.118966	21.15181	2.457824	-4.47053	0.045125	0.108491	1	0
1143	sp|P41105	RL28_MOU	16 kDa	0.998509	2	100%	P41105	Rpl28	60S ribosor	21.53802	19.7664	22.0227	20.84075	21.50734	21.23563	1.075095	0.031224	2.118966	21.15181	2.457824	-4.47053	0.045125	0.108491	1	0
300	sp|Q6NV83	SR140_MO	118 kDa	0.998301	8	100%	Q6NV83	U2surp	U2 snRNP-a	23.30277	23.86703	22.72884	23.66706	22.96029	23.55779	-0.69999	-1.38126	-0.01872	23.3473	-2.44553	-4.48438	0.045487	0.109035	0	0
1148	sp|Q9QX66	DPF1_MOU	44 kDa	0.998488	2	50%	Q9QX66	Dpf1	Zinc finger	19.46792	19.66093	19.06247	19.91646	19.11227	20.19687	-0.71053	-1.40218	-0.01889	19.56949	-2.44515	-4.48495	0.045512	0.109035	0	0
238	sp|O08553	DPYL2_MO	62 kDa	0.999407	9	67%	O08553	Dpysl2	Dihydropyr	22.47915	22.79795	21.95698	22.93316	22.20652	22.92139	-0.66995	-1.32271	-0.01719	22.54919	-2.4428	-4.48839	0.045667	0.109213	0	0
891	sp|Q8BG32	PSD11_MO	47 kDa	0.998773	3	100%	Q8BG32	Psmd11	26S protea	22.34552	19.62844	19.25061	18.66524	22.13427	19.29053	2.048736	0.048762	4.04871	20.2191	2.444625	-4.48977	0.045988	0.109594	1	0
892	sp|Q80WC7	AGFG2_MO	49 kDa	0.99877	3	100%	Q80WC7	Agfg2	Arf-GAP do	22.34552	19.62844	19.25061	18.66524	22.13427	19.29053	2.048736	0.048762	4.04871	20.2191	2.444625	-4.48977	0.045988	0.109594	1	0
92	sp|P14685	PSMD3_M	61 kDa	0.999619	16	100%	P14685	Psmd3	26S protea	23.61728	24.04203	23.26095	23.82918	23.25207	24.38788	-0.7096	-1.40298	-0.01621	23.73157	-2.43579	-4.49865	0.046131	0.109744	0	0
961	sp|Q64378	FKBP5_MO	51 kDa	0.997747	3	100%	Q64378	Fkbp5	Peptidyl-pr	21.81719	19.52478	21.06636	20.02997	21.8661	21.34804	1.282288	0.028515	2.536062	20.94207	2.440716	-4.49547	0.046246	0.109825	1	0
110	sp|Q7TQG1	PKHA6_M	131 kDa	0.999124	15	100%	Q7TQG1	Plekha6	Pleckstrin h	23.65664	21.53064	22.01761	22.06882	23.09271	21.79914	1.122787	0.023842	2.221731	22.36093	2.438216	-4.49911	0.046413	0.109911	1	0
881	sp|Q91V41	RAB14_MO	24 kDa	0.998909	3	67%	Q91V41	Rab14	Ras-related	20.1225	20.80785	20.42186	21.48924	20.29558	20.80112	-0.75275	-1.48972	-0.01578	20.65636	-2.43111	-4.5055	0.046445	0.109911	0	0
1128	sp|P35486	ODPA_MO	43 kDa	0.998636	2	100%	P35486	Pdha1	Pyruvate de	21.51543	20.80337	21.10743	20.53453	21.93836	20.8999	0.774473	0.015479	1.533466	21.13317	2.428672	-4.50906	0.046608	0.110037	0	0
1162	tr|A0A075	A0A075B5	13 kDa	0.99832	2	50%	A0A075B5	Igkv3-1	Immunoglo	19.78814	19.06371	20.4904	19.56496	19.70587	18.55576	0.933328	0.018131	1.848526	19.52814	2.431786	-4.50729	0.04671	0.110037	0	0
544	sp|O08576	RUN3A_M	50 kDa	0.999109	5	100%	O08576	Rundc3a	RUN domai	19.70988	23.13447	19.16494	21.78753	22.66871	23.89923	-2.4259	-4.80614	-0.04565	21.72746	-2.43221	-4.50786	0.046815	0.110037	-1	0
518	sp|P09103	PDIA1_MO	57 kDa	0.999354	5	100%	P09103	P4hb	Protein dis	20.44397	21.02852	20.36413	21.43987	20.71194	21.07258	-0.67364	-1.3352	-0.01209	20.8435	-2.42361	-4.51647	0.046951	0.110037	0	0
688	sp|Q9WU42	NCOR2_M	270 kDa	0.999006	4	100%	Q9WU42	Ncor2	Nuclear rec	18.56896	19.38305	18.10729	20.49671	19.62972	20.01871	-1.1975	-2.37403	-0.02098	19.36741	-2.42899	-4.51256	0.047032	0.110037	-1	0
900	tr|E9QAP7	E9QAP7_M	105 kDa	0.998684	3	100%	E9QAP7	Taf4	TATA-box-b	19.83317	20.60674	19.70936	21.62335	20.98159	22.32683	-1.34427	-2.66536	-0.02317	20.84684	-2.4283	-4.51357	0.047079	0.110037	-1	0
681	sp|Q9D0W5	PPIL1_MO	18 kDa	0.999051	4	100%	Q9D0W5	Ppil1	Peptidyl-pr	21.37567	20.23097	20.71761	20.84909	21.91929	19.55584	1.125558	0.019056	2.232059	20.77474	2.427541	-4.51467	0.04713	0.110037	1	0
297	sp|Q01853	TERA_MOU	89 kDa	0.998647	8	100%	Q01853	Vcp	Transitiona	22.64674	23.74505	23.27683	23.41815	22.3216	25.62771	-1.51524	-3.00499	-0.0255	23.50601	-2.42729	-4.51504	0.047147	0.110037	-1	0
275	sp|Q64012	RALY_MOU	33 kDa	0.999479	8	100%	Q64012	Raly	RNA-bindir	24.25149	23.26493	23.75608	23.47938	24.43591	23.54171	0.719155	0.008507	1.429802	23.78825	2.408623	-4.5384	0.04798	0.111635	0	0
594.1	sp|P01661	KV3A9_MO	14 kDa	0.999744	4	50%	P01661	NA	Ig kappa ch	17.65179	21.03122	20.27979	20.51485	19.21963	20.36423	-1.58636	-3.15483	-0.0179	19.84358	-2.41367	-4.53487	0.048079	0.111635	-1	0
594.2	sp|P01662	KV3AA_MO	12 kDa	0.999744	4	50%	P01662	NA	Ig kappa ch	17.65179	21.03122	20.27979	20.51485	19.21963	20.36423	-1.58636	-3.15483	-0.0179	19.84358	-2.41367	-4.53487	0.048079	0.111635	-1	0
98	sp|Q80U23	SNPH_MOU	54 kDa	0.999775	15	87%	Q80U23	Snph	Syntaphilir	24.56258	20.65571	23.42978	21.67479	24.41434	23.97825	2.032649	0.019173	4.046125	23.11924	2.409166	-4.54144	0.048392	0.112169	1	0
635	sp|P61027	RAB10_MO	23 kDa	0.99942	4	50%	P61027	Rab10	Ras-related	20.99505	20.28275	20.83494	19.72103	20.90705	20.52256	0.7369	0.002363	1.471436	20.5439	2.387788	-4.56887	0.049449	0.114084	0	0
907	sp|Q68FF6	GIT1_MOU	85 kDa	0.998593	3	100%	Q68FF6	Git1	ARF GTPase	20.14337	19.68871	20.58869	18.81858	21.31187	20.16075	1.125295	0.003098	2.247492	20.11866	2.39303	-4.56494	0.049528	0.114084	1	0
1288	sp|Q99KY4	GAK_MOUS	144 kDa	0.99518	2	100%	Q99KY4	Gak	Cyclin-G-as	19.7152	20.58733	20.4211	22.09889	20.70414	23.73377	-1.85985	-3.71486	-0.00484	21.21007	-2.39266	-4.56548	0.049554	0.114084	-1	0
1289	tr|A0A140	A0A140T8	13 kDa	0.995165	2	50%	A0A140T8	Igkv1-122	Immunoglo	19.7152	20.58733	20.4211	22.09889	20.70414	23.73377	-1.85985	-3.71486	-0.00484	21.21007	-2.39266	-4.56548	0.049554	0.114084	-1	0
1179	sp|O89079	COPE_MOU	35 kDa	0.998156	2	100%	O89079	Cope	Coatomer s	19.07455	21.38768	20.87227	21.07544	19.69889	20.73699	-1.1848	-2.36972	0.000114	20.4743	-2.38621	-4.57487	0.050016	0.114541	-1	0
1202	sp|Q8BTV2	CPSF7_MO	52 kDa	0.997928	2	100%	Q8BTV2	Cpsf7	Cleavage ar	20.75181	20.37373	21.80442	20.05874	20.7041	20.13299	0.898292	-0.00017	1.796752	20.63763	2.383485	-4.57738	0.050032	0.114541	0	0
259	sp|Q921F2	TADBP_MO	45 kDa	0.999752	8	100%	Q921F2	Tardbp	TAR DNA-bi	24.50671	23.36841	23.37408	22.91112	24.68604	23.31289	0.99147	-0.00039	1.983329	23.69321	2.385503	-4.5759	0.050068	0.114541	0	0
36	sp|E1U8D0	SOGA1_MO	159 kDa	0.99968	23	91%	E1U8D0	Soga1	Protein SO	23.38885	22.81849	22.73312	22.3781	23.91569	21.72737	1.037901	-0.00087	2.076668	22.82694	2.384452	-4.57743	0.050143	0.114541	1	0
85	sp|Q9CWF2	TBB2B_MO	50 kDa	0.998684	17	6%	Q9CWF2	Tubb2b	Tubulin bet	20.83364	19.967	21.31004	20.26951	20.2755	19.1449	1.012591	-0.00103	2.026213	20.3001	2.384014	-4.57807	0.050175	0.114541	1	0
193	sp|O55201	SPT5H_MO	121 kDa	0.999629	10	100%	O55201	Supt5h	Transcripti	24.24779	24.5721	22.28038	25.1202	24.24871	25.2634	-1.39294	-2.79075	0.004878	24.28876	-2.37811	-4.58666	0.050603	0.115324	-1	0
1066	sp|Q9DAW6	PRP4_MOU	58 kDa	0.999236	2	100%	Q9DAW6	Prpf4	U4/U6 sma	18.80277	18.89969	19.31853	22.16425	18.67556	21.70541	-1.99083	-3.99383	0.012164	19.9277	-2.37195	-4.59563	0.051054	0.116157	-1	0
997	sp|Q9CQY5	MAGT1_M	38 kDa	0.996064	3	100%	Q9CQY5	Magt1	Magnesium	19.52609	19.13462	19.692	18.33757	19.02055	18.0906	0.891949	-0.01032	1.794217	18.9669	2.356669	-4.61647	0.052008	0.11774	0	0
356.1	sp|P15864	H12_MOUS	21 kDa	0.999195	7	29%	P15864	Hist1h1c	Histone H1	23.14631	22.63602	23.42859	22.65585	23.57128	22.92237	0.643983	-0.00752	1.295483	23.06007	2.352665	-4.62019	0.05203	0.11774	0	0
356.2	sp|P43277	H13_MOUS	22 kDa	0.999195	7	29%	P43277	Hist1h1d	Histone H1	23.14631	22.63602	23.42859	22.65585	23.57128	22.92237	0.643983	-0.00752	1.295483	23.06007	2.352665	-4.62019	0.05203	0.11774	0	0
722	sp|Q99KK2	NEUA_MO	48 kDa	0.998657	4	100%	Q99KK2	Cmas	N-acylneur	21.61629	20.1071	21.84499	21.25798	21.27918	20.71487	0.886838	-0.01071	1.78439	21.13673	2.355475	-4.61821	0.052097	0.11774	0	0
971	sp|Q9D394	RUFY3_MO	53 kDa	0.997581	3	100%	Q9D394	Rufy3	Protein RU	18.15332	21.67238	16.86989	18.88303	NA	19.03884	-2.35315	-4.73929	0.032991	18.92349	-2.44447	-4.44132	0.052314	0.118033	-1	0
289	sp|Q91VI7	RINI_MOUS	50 kDa	0.999122	8	100%	Q91VI7	Rnh1	Ribonuclea	22.87007	23.23037	22.4386	23.09264	21.80077	23.20573	-0.80643	-1.62633	0.013471	22.77303	-2.34114	-4.63708	0.052916	0.119193	0	0
754	sp|Q9R233	TPSN_MOU	50 kDa	0.997633	4	100%	Q9R233	Tapbp	Tapasin	19.3476	20.46715	15.40894	19.93171	18.25698	19.42601	-2.27045	-4.58034	0.039446	18.8064	-2.34569	-4.63383	0.053024	0.119237	-1	0
309	sp|Q60932	VDAC1_M	32 kDa	0.99972	7	100%	Q60932	Vdac1	Voltage-de	23.46704	22.68136	24.19251	22.91082	23.37237	23.11738	0.774119	-0.01397	1.562205	23.29025	2.337947	-4.64168	0.053153	0.119331	0	0
433	sp|Q9D051	ODPB_MO	39 kDa	0.999287	6	100%	Q9D051	Pdhb	Pyruvate de	22.47638	23.53241	22.59785	23.1129	22.4291	22.88218	-0.67472	-1.36272	0.01329	22.83847	-2.33415	-4.64722	0.053447	0.119791	0	0
374	sp|Q9Z2I9	SUCB1_MO	50 kDa	0.998806	7	100%	Q9Z2I9	Sucla2	Succinate-	22.6028	24.62411	22.48925	23.09585	22.63647	23.18563	-1.05902	-2.14532	0.02728	23.10569	-2.32651	-4.6617	0.054511	0.121975	-1	0
387	sp|B2RQC6	PYR1_MOU	243 kDa	0.99735	7	100%	B2RQC6	Cad	CAD protei	20.18287	21.52596	19.64392	20.26748	20.4574	21.93899	-1.14941	-2.3297	0.030872	20.66944	-2.32402	-4.66532	0.054707	0.122213	-1	0
676	sp|Q9EPU4	CPSF1_MO	161 kDa	0.999086	4	100%	Q9EPU4	Cpsf1	Cleavage ar	20.60048	19.8573	19.9053	19.49584	20.3059	18.54551	0.971007	-0.02799	1.970003	19.78506	2.319579	-4.67177	0.055059	0.122724	0	0
1275	sp|Q7TNI2	TM59L_MO	38 kDa	0.995772	2	100%	Q7TNI2	Tmem59l	Transmemb	17.12223	15.45169	17.60215	16.91581	17.53475	NA	1.235958	-0.03787	2.509787	16.92533	2.405047	-4.49528	0.055117	0.122724	0	0
759	tr|A0A075	A0A075B5	13 kDa	0.997521	4	50%	A0A075B5	Ighv7-2	Immunoglo	20.51741	18.7803	19.95402	20.36438	21.16758	18.33081	1.387845	-0.04441	2.8201	19.85242	2.312445	-4.68213	0.055629	0.123515	0	0
792	sp|Q3V3Q7	PACS2_MO	95 kDa	0.999629	3	67%	Q3V3Q7	Pacs2	Phosphofu	19.66859	19.39897	19.95115	18.58753	19.50722	18.91582	0.741546	-0.02375	1.506841	19.33821	2.306268	-4.68789	0.055654	0.123515	0	0
409.1	sp|P61205	ARF3_MOU	21 kDa	0.999587	6	50%	P61205	Arf3	ADP-ribosy	22.93053	22.17259	23.22057	22.59268	22.81393	22.2623	0.645819	-0.02293	1.314569	22.66544	2.298519	-4.69918	0.056284	0.124505	0	0
409.2	sp|P84078	ARF1_MOU	21 kDa	0.999587	6	50%	P84078	Arf1	ADP-ribosy	22.93053	22.17259	23.22057	22.59268	22.81393	22.2623	0.645819	-0.02293	1.314569	22.66544	2.298519	-4.69918	0.056284	0.124505	0	0
1255	tr|G3X972	G3X972_M	119 kDa	0.996704	2	100%	G3X972	Sec24c	SEC24 relat	16.35178	18.29013	17.49884	19.74665	18.02601	18.05301	-1.40439	-2.8617	0.052929	17.9944	-2.29977	-4.70054	0.056657	0.125125	0	0
642	sp|P61372	ISL1_MOUS	39 kDa	0.999383	4	100%	P61372	Isl1	Insulin gen	21.44796	19.90153	20.70495	18.87481	20.57345	20.54201	1.136004	-0.04445	2.316459	20.34079	2.29658	-4.70516	0.056918	0.125498	0	0
947	sp|Q80VI1	TRI56_MO	80 kDa	0.997927	3	100%	Q80VI1	Trim56	E3 ubiquiti	19.90851	19.84905	19.02738	21.15116	19.64108	20.53434	-0.98586	-2.01146	0.039744	20.01859	-2.29396	-4.70896	0.057134	0.125769	0	0
248	sp|Q6ZWN5	RS9_MOUS	23 kDa	0.998829	9	100%	Q6ZWN5	Rps9	40S ribosor	25.33911	24.4305	25.31446	24.9012	25.10763	24.54685	0.627551	-0.0273	1.282399	24.93996	2.280916	-4.72482	0.057743	0.126438	0	0
643	sp|O70305	ATX2_MOU	136 kDa	0.999382	4	100%	O70305	Atxn2	Ataxin-2	23.61795	24.8761	24.78795	25.92741	23.7155	30.00502	-2.89571	-5.91873	0.127315	25.48832	-2.28594	-4.72061	0.0578	0.126438	0	0
578	sp|P35441	TSP1_MOU	130 kDa	0.997925	5	100%	P35441	Thbs1	Thrombosp	19.30392	18.31727	19.15528	18.17946	19.74027	19.17603	0.842234	-0.0373	1.721768	18.97871	2.282833	-4.72389	0.05787	0.126438	0	0
861	sp|Q8BK64	AHSA1_MO	38 kDa	0.999048	3	100%	Q8BK64	Ahsa1	Activator o	22.09504	21.6814	21.98867	21.1492	22.26285	21.58001	0.645314	-0.02855	1.319181	21.79286	2.279277	-4.7272	0.05788	0.126438	0	0
748	sp|Q6DID3	SCAF8_MO	140 kDa	0.997845	4	25%	Q6DID3	Scaf8	SR-related	20.36778	22.42936	22.17318	23.78716	19.41568	21.51597	-1.92528	-3.9363	0.085743	21.61486	-2.28469	-4.72241	0.057904	0.126438	0	0
117	sp|Q03265	ATPA_MOU	60 kDa	0.999674	14	100%	Q03265	Atp5f1a	ATP syntha	27.64126	27.00649	27.78058	26.94099	27.46944	27.16288	0.593641	-0.02717	1.214454	27.33361	2.275954	-4.73204	0.058161	0.126794	0	0
804	sp|Q4QQM4	P5I11_MO	21 kDa	0.999496	3	100%	Q4QQM4	Trp53i11	Tumor prot	13.88303	15.97338	14.62379	14.68579	NA	15.76436	-1.2211	-2.50283	0.060635	14.98607	-2.36148	-4.55494	0.058401	0.127114	0	0
783	tr|A0A075	A0A075B5	10 kDa	0.999719	3	100%	A0A075B5	Igkv4-63	Immunoglo	19.42727	21.06022	21.05137	22.2924	21.26137	22.81953	-1.47738	-3.02855	0.073783	21.31869	-2.27293	-4.73947	0.058897	0.127808	0	0
698	sp|Q91V12	BACH_MO	43 kDa	0.998903	4	100%	Q91V12	Acot7	Cytosolic a	22.18701	21.56343	21.69473	21.30658	22.27314	21.31347	0.657133	-0.03294	1.347204	21.72306	2.266522	-4.74576	0.058964	0.127808	0	0
610	sp|Q5SUF2	LC7L3_MO	51 kDa	0.999627	4	100%	Q5SUF2	Luc7l3	Luc7-like p	21.09798	21.80842	20.7883	21.59458	21.53058	22.33529	-0.77381	-1.58657	0.038954	21.52586	-2.26617	-4.74634	0.059003	0.127808	0	0
326	sp|P26039	TLN1_MOU	270 kDa	0.999568	7	100%	P26039	Tln1	Talin-1	20.72182	21.70198	21.38482	21.37281	20.78427	22.10382	-0.76257	-1.56408	0.03895	21.34492	-2.26447	-4.74875	0.05914	0.127901	0	0
48	sp|Q61656	DDX5_MOU	69 kDa	0.999587	20	60%	Q61656	Ddx5	Probable A	24.37106	23.50322	24.3068	23.23213	24.07786	23.96358	0.685599	-0.03733	1.408529	23.90911	2.257227	-4.75928	0.059766	0.129049	0	0
758	sp|P62196	PRS8_MOU	46 kDa	0.997543	4	100%	P62196	Psmc5	26S protea	19.3257	20.27089	17.8908	18.97265	16.0805	19.59572	-1.84742	-3.80081	0.105969	18.68938	-2.25698	-4.76258	0.060271	0.129801	0	0
1121	sp|P27546	MAP4_MO	117 kDa	0.998723	2	100%	P27546	Map4	Microtubu	19.63936	19.57015	19.57633	17.9499	19.49861	17.39109	1.267717	-0.07295	2.608388	18.93757	2.256583	-4.76315	0.060306	0.129801	0	0
1161	sp|Q8VEK0	CC50A_MO	41 kDa	0.998326	2	100%	Q8VEK0	Tmem30a	Cell cycle c	18.2963	17.94929	18.50579	17.21228	18.18613	17.75457	0.690692	-0.04123	1.422612	17.98406	2.246065	-4.7755	0.060744	0.130537	0	0
49	sp|P07724	ALBU_MO	69 kDa	0.999586	20	100%	P07724	Alb	Serum albu	25.14534	25.54977	24.56097	26.08565	25.69402	26.99156	-1.07555	-2.21818	0.067077	25.67122	-2.24635	-4.77797	0.061205	0.13132	0	0
105	sp|P20152	VIME_MOU	54 kDa	0.999598	15	93%	P20152	Vim	Vimentin	23.75672	23.50756	23.68261	26.28432	23.55944	26.29925	-1.69745	-3.50863	0.113727	24.51498	-2.23659	-4.79209	0.062075	0.132976	0	0
896	sp|P62245	RS15A_MO	15 kDa	0.998735	3	100%	P62245	Rps15a	40S ribosor	21.14847	20.39999	20.74654	19.2188	19.97439	18.54891	1.233898	-0.08482	2.552619	20.00618	2.232939	-4.79737	0.062404	0.133259	0	0
897	sp|Q8R1A4	DOCK7_M	241 kDa	0.998714	3	100%	Q8R1A4	Dock7	Dedicator c	21.14847	20.39999	20.74654	19.2188	19.97439	18.54891	1.233898	-0.08482	2.552619	20.00618	2.232939	-4.79737	0.062404	0.133259	0	0
125	sp|P16858	G3P_MOUS	36 kDa	0.999792	13	85%	P16858	Gapdh	Glyceraldel	28.16674	27.56843	28.26585	27.57199	28.00266	27.61463	0.560067	-0.0435	1.163636	27.86505	2.208585	-4.8299	0.064149	0.136665	0	0
424	sp|Q9ERI2	RB27A_MO	25 kDa	0.999413	6	67%	Q9ERI2	Rab27a	Ras-related	23.33434	22.84304	23.4829	22.62545	22.95118	22.32735	0.657527	-0.05125	1.366301	22.92738	2.208042	-4.83069	0.0642	0.136665	0	0
1282	sp|Q8VI75	IPO4_MOU	119 kDa	0.995585	2	100%	Q8VI75	Ipo4	Importin-4	16.87967	18.7431	15.36351	24.20768	16.46692	17.10444	-3.78171	-7.87076	0.307342	18.12755	-2.20707	-4.83475	0.064785	0.137693	0	0
1268	sp|Q9ERS2	NDUAD_M	17 kDa	0.996241	2	100%	Q9ERS2	Ndufa13	NADH dehy	16.35494	18.75895	17.40005	19.32248	18.3782	17.97596	-1.30807	-2.72885	0.112713	18.03177	-2.19712	-4.84912	0.065725	0.139458	0	0
837	sp|Q9D819	IPYR_MOUS	33 kDa	0.999266	3	100%	Q9D819	Ppa1	Inorganic p	21.30183	18.63942	20.36794	20.15095	21.9805	20.65114	1.402921	-0.12232	2.928164	20.5153	2.195051	-4.8521	0.065922	0.139458	0	0
926	sp|P01795	HVM26_M	16 kDa	0.998317	3	67%	P01795	NA	Ig heavy ch	19.84225	22.13909	20.03015	21.77417	19.08465	19.6113	-1.5225	-3.1778	0.132798	20.4136	-2.19499	-4.8522	0.065928	0.139458	0	0
659	sp|Q8K2C9	HACD3_M	43 kDa	0.999233	4	100%	Q8K2C9	Hacd3	Very-long-c	21.67203	19.79713	20.94981	21.38432	21.63156	19.32649	1.248487	-0.10953	2.606505	20.79356	2.193961	-4.85368	0.066026	0.139458	0	0
737	sp|B1AZI6	THOC2_MO	183 kDa	0.998344	4	100%	B1AZI6	Thoc2	THO compl	14.35192	16.63941	13.08975	15.25741	15.14647	14.98837	-1.43235	-2.9972	0.132508	14.91222	-2.18436	-4.86752	0.066951	0.141117	0	0
1029	sp|P61969	LMO4_MO	18 kDa	0.999551	2	100%	P61969	Lmo4	LIM domair	20.35958	19.44375	19.82501	19.74339	20.07819	18.65762	0.806004	-0.07475	1.686757	19.68459	2.181612	-4.87049	0.06702	0.141117	0	0
674	sp|Q6PHN9	RAB35_MO	23 kDa	0.999093	4	75%	Q6PHN9	Rab35	Ras-related	22.62656	21.8727	22.81318	22.32889	22.57005	22.02688	0.593767	-0.05576	1.243294	22.37304	2.175805	-4.87736	0.067286	0.141457	0	0
690	tr|Q8C6B0	Q8C6B0_M	28 kDa	0.998998	4	100%	Q8C6B0	Mettl7a1	MCG20149	22.2672	23.67224	22.61654	22.55147	22.44558	23.56109	-0.81849	-1.71562	0.078633	22.85236	-2.17498	-4.88006	0.067668	0.141622	0	0
903.2	sp|Q64523	H2A2C_M	14 kDa	0.998649	3	33%	Q64523	Hist2h2ac	Histone H2	18.29777	20.58504	16.49685	18.40752	18.35316	18.94464	-1.59647	-3.34661	0.153667	18.51416	-2.17691	-4.87827	0.067678	0.141622	0	0
904	sp|P61021	RAB5B_MO	24 kDa	0.998636	3	33%	P61021	Rab5b	Ras-related	18.29777	20.58504	16.49685	18.40752	18.35316	18.94464	-1.59647	-3.34661	0.153667	18.51416	-2.17691	-4.87827	0.067678	0.141622	0	0
2	sp|Q61879	MYH10_M	229 kDa	0.999667	65	80%	Q61879	Myh10	Myosin-10	25.34386	25.94549	25.2567	25.98808	25.65435	26.03852	-0.5724	-1.20085	0.056055	25.7045	-2.16783	-4.88889	0.068072	0.142227	0	0
413	sp|Q8C9B9	DIDO1_MO	247 kDa	0.999571	6	100%	Q8C9B9	Dido1	Death-indu	19.48619	19.17092	19.42985	28.65219	19.07651	24.02508	-4.61855	-9.70578	0.468683	21.64012	-2.16658	-4.89314	0.068698	0.142911	0	0
623.1	sp|P51410	RL9_MOUS	22 kDa	0.999492	4	100%	P51410	Rpl9	60S ribosor	22.37216	21.71602	22.55843	21.55681	22.40655	22.18749	0.625609	-0.06332	1.314537	22.13291	2.161373	-4.89822	0.068715	0.142911	0	0
623.2	tr|A0A140	A0A140T8	22 kDa	0.999492	4	100%	A0A140T8	Rpl9-ps6	Ribosomal	22.37216	21.71602	22.55843	21.55681	22.40655	22.18749	0.625609	-0.06332	1.314537	22.13291	2.161373	-4.89822	0.068715	0.142911	0	0
229	sp|Q9CSN1	SNW1_MO	61 kDa	0.999739	9	100%	Q9CSN1	Snw1	SNW doma	22.63537	23.28721	21.94737	24.45001	23.21464	28.76926	-2.90303	-6.10542	0.29935	24.05064	-2.16336	-4.89777	0.069019	0.143092	0	0
847	sp|P21550	ENOB_MO	47 kDa	0.999183	3	33%	P21550	Eno3	Beta-enola	21.59499	19.76458	21.30936	20.68132	21.82217	21.3675	0.971038	-0.10022	2.042298	21.08999	2.163179	-4.89804	0.069037	0.143092	0	0
972	tr|V9GX06	V9GX06_M	20 kDa	0.997556	3	33%	V9GX06	Gm11214	Predicted g	18.50721	19.78297	18.03757	18.59997	18.84494	19.82273	-0.93865	-1.97457	0.097268	18.93257	-2.16236	-4.89921	0.069119	0.143092	0	0
20	sp|Q99NB9	SF3B1_MO	146 kDa	0.999583	29	100%	Q99NB9	Sf3b1	Splicing fac	25.87324	24.79496	25.11067	24.78684	26.0706	25.20829	0.754806	-0.07876	1.588375	25.30743	2.15534	-4.90698	0.069331	0.143313	0	0

927	sp|Q99JI4	PSMD6_MO	46 kDa	0.998291	3	100%	Q99JI4	Psmd6	26S protea	20.53188	22.94741	20.64792	24.1763	20.45174	20.35352	-1.94856	-4.10926	0.212132	21.51813	-2.15215	-4.91392	0.07015	0.144407	0	0
1188	sp|P53026	RL10A_MO	25 kDa	0.998088	2	100%	P53026	Rpl10a	60S riboso	16.47305	15.93451	15.75128	18.88918	16.27828	19.73769	-2.01959	-4.25931	0.220138	17.17733	-2.15188	-4.91429	0.070177	0.144407	0	0
1206	sp|Q91YI0	ARLY_MOU	52 kDa	0.997863	2	100%	Q91YI0	Asl	Argininosu	19.60739	19.38953	19.69633	18.52972	19.49555	18.84432	0.678569	-0.07372	1.430856	19.26047	2.146896	-4.91912	0.07018	0.144407	0	0
749	tr|A0A140	A0A140T8	13 kDa	0.997834	4	25%	A0A140T8	Igkv6-14	Immunoglo	21.24037	23.49166	24.40437	26.1255	22.75383	24.90559	-2.0414	-4.31043	0.227639	23.82022	-2.14702	-4.92128	0.070673	0.144981	0	0
750.1	sp|P01635	KV5A3_MO	13 kDa	0.997781	4	50%	P01635	NA	Ig kappa ch	21.24037	23.49166	24.40437	26.1255	22.75383	24.90559	-2.0414	-4.31043	0.227639	23.82022	-2.14702	-4.92128	0.070673	0.144981	0	0
1273	sp|Q99L04	DHRS1_MO	34 kDa	0.996	2	100%	Q99L04	Dhrs1	Dehydroge	18.49905	16.14366	17.89996	17.81497	17.73644	17.08379	1.031013	-0.11706	2.179087	17.52964	2.143111	-4.92691	0.071075	0.145585	0	0
1139	sp|Q8VCW	ACSF2_MO	68 kDa	0.998538	2	100%	Q8VCW8	Acsf2	Acyl-CoA sy	18.50746	18.33504	19.98435	17.84882	18.7316	18.18022	0.953114	-0.10877	2.015002	18.59791	2.141986	-4.92852	0.071191	0.145602	0	0
1176	sp|Q61335	BAP31_MO	28 kDa	0.998178	2	100%	Q61335	Bcap31	B-cell recep	20.72006	21.74586	21.5107	21.60528	21.16145	23.15	-1.03631	-2.19588	0.123269	21.64889	-2.13275	-4.94179	0.07215	0.147121	0	0
1177.1	sp|P01750	HVM06_M	13 kDa	0.998168	2	50%	P01750	NA	Ig heavy ch	20.72006	21.74586	21.5107	21.60528	21.16145	23.15	-1.03631	-2.19588	0.123269	21.64889	-2.13275	-4.94179	0.07215	0.147121	0	0
316	sp|P53994	RAB2A_MO	24 kDa	0.999651	7	29%	P53994	Rab2a	Ras-related	22.90826	22.36791	22.9107	22.24305	22.60549	22.13464	0.559615	-0.06757	1.186804	22.52834	2.123693	-4.95256	0.072594	0.147803	0	0
791	tr|A0A140	A0A140T8	13 kDa	0.999633	3	67%	A0A140T8	Igkv12-46	Immunoglo	22.37028	24.61678	25.16711	26.85868	22.747	24.67052	-1.95387	-4.14615	0.238421	24.40506	-2.1269	-4.95018	0.072764	0.147928	0	0
38	sp|Q8BSQ9	PB1_MOUS	187 kDa	0.999426	23	100%	Q8BSQ9	Pbrm1	Protein pol	24.00882	24.38111	22.83492	24.53214	24.1884	24.79239	-0.89117	-1.89175	0.109415	24.12296	-2.12548	-4.95223	0.072914	0.148012	0	0
589	sp|P57776	EF1D_MOU	31 kDa	0.99988	4	100%	P57776	Eef1d	Elongation	20.68779	21.34694	21.48152	21.72065	20.77347	22.20864	-0.77782	-1.65251	0.096874	21.36983	-2.11991	-4.95935	0.073303	0.148527	0	0
177	sp|Q3TLH4	PRC2C_MO	311 kDa	0.999166	11	100%	Q3TLH4	Prrc2c	Protein PRI	22.51569	21.66818	21.91524	21.97381	23.06239	21.50143	0.783296	-0.09788	1.664476	22.10612	2.119119	-4.96048	0.073387	0.148527	0	0
995	sp|A0A0B4	FCGR4_MO	28 kDa	0.996145	3	100%	A0A0B4J1	Fcgr4	Low affinity	19.48471	19.5456	19.12275	21.14874	19.69717	20.48786	-0.95919	-2.04133	0.12295	19.91447	-2.1153	-4.96683	0.073999	0.149541	0	0
1060.1	sp|P84104	SRSF3_MO	19 kDa	0.999248	2	100%	P84104	Srsf3	Serine/argi	18.04716	17.88871	18.42212	16.55398	17.99105	17.46613	0.850503	-0.11351	1.814518	17.72819	2.105441	-4.98096	0.075064	0.151377	0	0
669	sp|Q61466	SMRD1_M	58 kDa	0.999133	4	100%	Q61466	Smarcd1	SWI/SNF-re	20.89988	19.41932	19.69688	19.84245	20.92136	19.68011	0.858749	-0.11489	1.832393	20.07667	2.10483	-4.98184	0.07513	0.151377	0	0
726	sp|P62852	RS25_MOU	14 kDa	0.998563	4	100%	P62852	Rps25	40S riboso	23.24155	23.15851	23.94058	22.4012	23.2139	22.62173	0.738199	-0.09965	1.576043	23.09625	2.097779	-4.99011	0.075454	0.151805	0	0
1080	sp|P63321	RALA_MOU	24 kDa	0.999136	2	100%	P63321	Rala	Ras-related	17.08238	18.81028	19.13867	19.00508	17.31019	19.30791	-1.19734	-2.55797	0.163281	18.44242	-2.10006	-4.98867	0.075652	0.151976	0	0
1196	sp|Q91YW	DNJC3_MO	57 kDa	0.998015	2	100%	Q91YW3	Dnajc3	DnaJ homo	19.86516	19.15825	19.3162	16.8684	19.68905	18.91287	1.3103	-0.17984	2.800441	18.96832	2.098428	-4.99101	0.075831	0.152111	0	0
1115	sp|Q9Z2Z6	MCAT_MOU	33 kDa	0.998798	2	100%	Q9Z2Z6	Slc25a20	Mitochond	18.16067	19.06355	18.15772	18.1919	17.55955	18.83327	-0.73693	-1.57661	0.102748	18.32778	-2.09008	-5.00134	0.07635	0.152926	0	0
832.3	sp|P01667	KV3AF_MO	12 kDa	0.999312	3	33%	P01667	NA	Ig kappa ch	21.28451	22.07846	21.4009	22.0484	21.74552	21.97898	-0.5583	-1.19923	0.082625	21.75613	-2.0733	-5.02497	0.078126	0.156253	0	0
832.4	sp|P01669	KV3AH_MO	12 kDa	0.999312	3	33%	P01669	NA	Ig kappa ch	21.11795	21.2825	20.81303	21.2857	20.13261	21.63708	-0.7139	-1.53462	0.106829	21.04481	-2.07042	-5.02912	0.078464	0.156697	0	0
876	sp|Q8BH43	WASF2_MO	54 kDa	0.998953	3	100%	Q8BH43	Wasf2	Wiskott-Ald	19.41742	20.28957	19.92755	20.26697	19.30488	19.94935	-0.61868	-1.33059	0.093226	19.85929	-2.06845	-5.03191	0.078681	0.156898	0	0
604	sp|P35979	RL12_MOU	18 kDa	0.99968	4	100%	P35979	Rpl12	60S riboso	21.17717	20.86809	22.20451	20.85737	21.13721	20.43754	0.785296	-0.12088	1.691476	21.11365	2.065917	-5.03671	0.079285	0.157871	0	0
747	sp|P54775	PRS6B_MO	47 kDa	0.997862	4	100%	P54775	Psmc4	26S protea	17.61825	19.01362	18.03245	18.91802	18.36808	18.24818	-0.72035	-1.55389	0.113188	18.36643	-2.05703	-5.0483	0.080011	0.159083	0	0
848	sp|Q8BI72	CARF_MOU	60 kDa	0.999175	3	100%	Q8BI72	Cdkn2aip	CDKN2A-in	18.8391	22.32455	19.24099	18.67581	19.26623	23.77319	-2.47574	-5.34395	0.392464	20.35331	-2.0599	-5.04607	0.080188	0.159202	0	0
686	sp|Q9D1H7	GET4_MOU	37 kDa	0.999009	4	100%	Q9D1H7	Get4	Golgi to ER	20.27562	21.25011	20.78394	21.18487	20.79132	21.13495	-0.57302	-1.24005	0.094015	20.90347	-2.04466	-5.06594	0.081456	0.161483	0	0
429	sp|Q91YT0	NDUV1_M	51 kDa	0.999344	6	100%	Q91YT0	Ndufv1	NADH dehy	22.94854	21.28818	21.62113	22.16856	22.89369	20.90478	1.033948	-0.17229	2.240188	21.97081	2.045575	-5.06649	0.081871	0.162069	0	0
1105	sp|Q64471	GSTT1_MO	27 kDa	0.998915	2	100%	Q64471	Gstt1	Glutathion	20.76699	20.15229	21.15158	20.10874	20.58863	20.46888	0.59243	-0.09974	1.284605	20.53952	2.037147	-5.07667	0.082353	0.162785	0	0
1007	sp|P63168	DYL1_MOU	10 kDa	0.995001	3	33%	P63168	Dynll1	Dynein ligh	20.14808	20.3688	20.73643	22.17295	20.19844	21.59913	-1.01931	-2.21235	0.173729	20.87064	-2.03893	-5.07595	0.082664	0.163162	0	0
670	sp|P19783	COX41_MO	20 kDa	0.999124	4	100%	P19783	Cox4i1	Cytochrom	22.38329	24.04041	23.76296	23.92295	22.54927	23.41614	-0.89466	-1.94702	0.157702	23.34584	-2.02882	-5.09033	0.083884	0.16533	0	0
194.1	sp|P03987	IGHG3_MO	44 kDa	0.999623	10	100%	P03987	NA	Ig gamma-3	31.06637	31.41955	31.23238	31.84414	30.74192	31.51004	-0.57769	-1.26244	0.107071	31.3024	-2.00797	-5.11826	0.08593	0.168627	0	0
194.2	tr|A0A1Y7	A0A1Y7VJ	44 kDa	0.999623	10	100%	A0A1Y7VJN	Ighg3	Immunoglo	31.06637	31.41955	31.23238	31.84414	30.74192	31.51004	-0.57769	-1.26244	0.107071	31.3024	-2.00797	-5.11826	0.08593	0.168627	0	0
194.3	tr|A0A075	A0A075B5	36 kDa	0.999623	10	100%	A0A075B5	Ighg3	Immunoglo	31.06637	31.41955	31.23238	31.84414	30.74192	31.51004	-0.57769	-1.26244	0.107071	31.3024	-2.00797	-5.11826	0.08593	0.168627	0	0
73	sp|Q9QWL	K1C17_MO	48 kDa	0.999655	17	41%	Q9QWL7	Krt17	Keratin, typ	21.78826	23.94931	24.59178	25.21657	23.7104	25.73277	-1.60274	-3.50653	0.301047	24.16485	-2.00907	-5.11837	0.086321	0.169149	0	0
1203	sp|Q91Z49	UIF_MOUS	36 kDa	0.997924	2	100%	Q91Z49	Fyttd1	UAP56-inte	17.54395	20.67548	21.10342	22.47061	19.29936	20.56734	-1.92223	-4.20837	0.363908	20.27669	-2.00657	-5.12192	0.086635	0.169519	0	0
1266	sp|Q80WM	HPLN4_MO	43 kDa	0.996345	2	100%	Q80WM4	Hapln4	Hyaluronar	17.31378	17.18517	16.70941	18.95847	16.57449	17.54914	-1.0317	-2.26042	0.197016	17.38174	-2.00379	-5.12586	0.086984	0.169755	0	0
11	sp|Q3UHU	MTCL1_MO	214 kDa	0.99993	39	92%	Q3UHU5	Mtcl1	Microtubul	25.32065	23.95463	24.22275	24.3119	25.82497	24.38785	0.904665	-0.17285	1.982179	24.67046	2.003621	-5.1261	0.087006	0.169755	0	0
989	sp|Q6NZA9	TAF9B_MO	27 kDa	0.996854	3	100%	Q6NZA9	Taf9b	Transcripti	19.22655	21.38171	18.28191	19.95401	19.96765	19.74334	-1.20098	-2.63262	0.230657	19.7592	-2.00195	-5.12847	0.087217	0.169922	0	0
263	sp|P18872	GNAO_MO	40 kDa	0.999709	8	75%	P18872	Gnao1	Guanine nu	23.37746	22.92768	23.83198	23.16522	23.26878	22.4688	0.638839	-0.12464	1.402315	23.17332	1.991576	-5.14155	0.088006	0.171215	0	0
973	sp|P61211	ARL1_MOU	20 kDa	0.997547	3	100%	P61211	Arl1	ADP-ribosy	21.06796	20.32074	21.05429	20.73157	20.71281	19.47779	0.768323	-0.15257	1.689217	20.56086	1.990481	-5.14453	0.08862	0.172162	0	0
872	sp|Q8VDM	HNRL1_MO	96 kDa	0.998975	3	100%	Q8VDM6	Hnrnpul1	Heterogene	17.87002	20.53286	17.82245	18.14826	18.4014	19.03686	-1.20803	-2.65826	0.242192	18.63531	-1.9879	-5.14837	0.089011	0.172674	0	0
809	sp|P21460	CYTC_MOU	16 kDa	0.999461	3	100%	P21460	Cst3	Cystatin-C	20.25379	20.61908	20.32381	21.16063	19.97906	20.55024	-0.5911	-1.30197	0.11978	20.4811	-1.97909	-5.15927	0.089622	0.173514	0	0
1125	sp|Q8VBT0	TMX1_MO	31 kDa	0.998676	2	100%	Q8VBT0	Tmx1	Thioredoxi	20.1296	20.37123	21.13759	18.59105	21.40044	20.22167	1.161229	-0.23704	2.559497	20.30859	1.981884	-5.15687	0.089791	0.173514	0	0
692	sp|P01671	KV3AJ_MO	12 kDa	0.998994	4	50%	P01671	NA	Ig kappa ch	21.86401	22.76124	21.36318	27.61294	22.02664	22.61566	-2.57867	-5.68444	0.527106	23.04061	-1.98142	-5.15753	0.089851	0.173514	0	0
1224	sp|Q9JIK9	RT34_MOU	26 kDa	0.997577	2	100%	Q9JIK9	Mrps34	28S riboso	17.41549	17.08561	17.37655	16.21332	17.34728	16.93214	0.636085	-0.12989	1.402057	17.06173	1.976531	-5.16289	0.089956	0.173514	0	0
344	sp|Q05920	PYC_MOUS	130 kDa	0.999363	7	100%	Q05920	Pc	Pyruvate ca	21.57737	22.62811	21.58681	22.18397	22.1268	22.26508	-0.5954	-1.31398	0.123184	22.06136	-1.97211	-5.16915	0.090537	0.174169	0	0
1016	sp|Q9CRB2	NHP2_MO	17 kDa	0.999784	2	100%	Q9CRB2	Nhp2	H/ACA ribo	20.50024	20.11229	18.97951	21.27965	19.62813	20.64152	-0.97519	-2.15292	0.202529	20.19022	-1.97605	-5.16511	0.090553	0.174169	0	0
165	sp|Q99LF4	RTCB_MOU	55 kDa	0.999492	11	100%	Q99LF4	Rtcb	tRNA-splici	23.6508	24.02761	23.06105	23.62805	23.19807	24.02312	-0.58962	-1.30277	0.12353	23.59812	-1.96785	-5.17519	0.0911	0.174975	0	0
351	sp|Q9EQP2	EHD4_MO	61 kDa	0.999293	7	100%	Q9EQP2	Ehd4	EH domain-	21.90479	23.40897	22.56605	22.56219	21.64188	22.47898	-0.77914	-1.72611	0.167832	22.42714	-1.96349	-5.18284	0.092216	0.176867	0	0
515	sp|P35550	FBRL_MOU	34 kDa	0.999391	5	100%	P35550	Fbl	rRNA 2'-O-r	21.53485	20.90654	20.80488	20.82081	21.67181	19.73344	0.850251	-0.18442	1.884925	20.91206	1.961076	-5.18625	0.092539	0.176918	0	0
26	tr|E9Q6R7	E9Q6R7_M	393 kDa	0.999404	26	100%	E9Q6R7	Utrn	Utrophin	23.74854	24.11449	22.95712	24.01407	23.29688	23.72689	-0.61764	-1.36898	0.133705	23.643	-1.95657	-5.19114	0.092608	0.176918	0	0
1254	sp|Q61838	PZP_MOUS	166 kDa	0.996716	2	100%	Q61838	Pzp	Pregnancy	18.06137	17.17247	17.91895	17.92188	18.16303	15.57041	1.159525	-0.25201	2.571064	17.46802	1.960368	-5.18725	0.092634	0.176918	0	0
814	sp|P62889	RL30_MOU	13 kDa	0.999429	3	100%	P62889	Rpl30	60S riboso	18.76563	20.81154	22.01423	23.84083	20.20538	22.31093	-1.99269	-4.43086	0.445487	21.32476	-1.95041	-5.20128	0.093981	0.179237	0	0
272	sp|Q9CY64	BIEA_MOU	34 kDa	0.999545	8	100%	Q9CY64	Blvra	Biliverdin r	22.22623	21.43275	22.26378	21.66638	21.86617	21.6715	0.52852	-0.11822	1.175259	21.85447	1.94506	-5.2074	0.094173	0.179353	0	0
582	sp|Q80XI3	IF4G3_MO	175 kDa	0.997392	5	60%	Q80XI3	Eif4g3	Eukaryotic	18.57404	17.95481	18.62386	17.98449	18.65492	18.36391	0.516538	-0.11883	1.151901	18.35934	1.934999	-5.22158	0.095562	0.181742	0	0
129	sp|Q8C2Q3	RBM14_M	69 kDa	0.999661	13	100%	Q8C2Q3	Rbm14	RNA-bindir	23.65097	23.01881	24.15315	23.52274	23.68625	23.22118	0.575876	-0.13336	1.285108	23.54218	1.932598	-5.22497	0.095897	0.182123	0	0
355	sp|Q00612	G6PD1_M	59 kDa	0.99922	7	100%	Q00612	G6pdx	Glucose-6-	21.28601	21.79778	21.25247	22.04192	21.36447	21.60195	-0.5129	-1.14668	0.120881	21.55743	-1.92617	-5.23402	0.096798	0.183578	0	0
733	sp|P80314	TCPB_MOU	57 kDa	0.998386	4	100%	P80314	Cct2	T-complex	21.83593	22.83208	19.75246	21.64363	21.73009	22.54547	-1.23423	-2.76294	0.294472	21.72328	-1.92674	-5.23454	0.097257	0.18419	0	0
368	sp|Q9D554	SF3A3_MO	59 kDa	0.999004	7	100%	Q9D554	Sf3a3	Splicing fac	23.46252	22.1916	22.37233	22.83474	23.45323	21.57347	0.896091	-0.21557	2.007748	22.64798	1.923677	-5.23884	0.09769	0.184635	0	0
712	sp|P59708	SF3B6_MO	15 kDa	0.998754	4	100%	P59708	Sf3b6	Splicing fac	23.31885	24.30827	22.60936	23.4416	23.45743	23.78346	-0.7159	-1.60402	0.172225	23.48649	-1.92164	-5.24118	0.097764	0.184635	0	0
633	sp|Q8R1Q8	DC1L1_MO	57 kDa	0.999422	4	100%	Q8R1Q8	Dync1li1	Cytoplasmi	20.62262	20.38908	21.32239	19.68148	21.79843	20.99653	0.89212	-0.21566	1.999895	20.80175	1.921863	-5.24139	0.097947	0.184723	0	0
1239	sp|Q920R6	VPP4_MOU	96 kDa	0.997235	2	50%	Q920R6	Atp6v0a4	V-type prot	19.78293	20.66062	19.81136	20.35308	20.27215	23.29947	-1.48224	-3.3259	0.361409	20.6966	-1.91863	-5.24592	0.098406	0.185331	0	0
337	sp|P04919	B3AT_MOU	103 kDa	0.999457	7	100%	P04919	Slc4a1	Band 3 anio	22.61503	21.81561	22.10359	20.26714	21.51081	21.30427	0.947472	-0.23211	2.127058	21.60274	1.916848	-5.24842	0.09866	0.185552	0	0
1048	sp|O08529	CAN2_MOU	80 kDa	0.999364	2	100%	O08529	Capn2	Calpain-2 c	19.61498	18.73694	21.28691	19.80762	20.13886	19.53021	0.988664	-0.24366	2.220993	19.85259	1.914578	-5.2516	0.098985	0.185905	0	0
192	sp|Q61584	FXR1_MOU	76 kDa	0.999635	10	90%	Q61584	Fxr1	Fragile X m	22.1959	20.3458	21.5801	20.38229	21.96489	22.04761	0.988393	-0.24478	2.221564	21.41943	1.912746	-5.25416	0.099248	0.186141	0	0
244	sp|P67778	PHB_MOUS	30 kDa	0.999094	9	100%	P67778	Phb	Prohibitin	22.88709	23.70663	23.35839	23.59607	22.54393	23.30905	-0.60745	-1.36706	0.152164	23.23353	-1.90335	-5.26608	0.100064	0.187413	0	0
920	sp|P70452	STX4_MOU	34 kDa	0.998396	3	100%	P70452	Stx4	Syntaxin-4	21.55224	21.87244	22.14579	22.66145	21.24589	22.50962	-0.69986	-1.57974	0.180017	21.9979	-1.8962	-5.27683	0.101437	0.189723	0	0
1055	sp|A2AI08	TPRN_MOU	80 kDa	0.999289	2	100%	A2AI08	Tprn	Taperin	17.34622	19.36158	15.93158	17.5636	15.94543	16.6191	-1.44035	-3.25393	0.373222	17.12792	-1.89533	-5.27853	0.101781	0.189842	0	0
1056.1	sp|P02463	CO4A1_MO	161 kDa	0.999285	2	100%	P02463	Col4a1	Collagen al	17.34622	19.36158	15.93158	17.5636	15.94543	16.6191	-1.44035	-3.25393	0.373222	17.12792	-1.89533	-5.27853	0.101781	0.189842	0	0
1175	sp|Q8VCM	NCLN_MOU	63 kDa	0.998178	2	100%	Q8VCM8	Ncln	Nicalin	22.78731	23.43039	23.23011	23.26334	22.39577	23.48805	-0.58953	-1.33325	0.154194	23.09916	-1.88667	-5.28944	0.10252	0.190797	0	0
870	sp|P97461	RS5_MOUS	23 kDa	0.998991	3	100%	P97461	Rps5	40S riboso	20.62235	21.00697	21.06139	21.68757	20.61873	21.3633	-0.58513	-1.32344	0.153187	21.06005	-1.8863	-5.28996	0.102574	0.190797	0	0
1049	sp|P62855	RS26_MOU	13 kDa	0.999332	2	100%	P62855	Rps26	40S riboso	18.8746	20.18957	19.34226	20.1773	20.08921	20.0271	-0.69597	-1.57515	0.183218	19.78334	-1.88713	-5.2895	0.102778	0.190915	0	0
798.1	sp|Q9WV0	RBMX_MO	42 kDa	0.999544	3	100%	Q9WV02	Rbmx	RNA-bindir	22.08296	20.84523	20.38172	19.78061	21.85413	20.63443	1.019515	-0.2713	2.310336	20.92984	1.884858	-5.29314	0.103334	0.191684	0	0
237	sp|Q61666	HIRA_MOU	112 kDa	0.999458	9	100%	Q61666	Hira	Protein HIR	22.95569	21.48586	21.55902	21.54089	24.35422	22.00045	1.280577	-0.34562	2.906779	22.31602	1.879239	-5.30097	0.104177	0.192984	0	0
819	sp|Q5FWK	RHG01_M	50 kDa	0.999402	3	100%	Q5FWK3	Arhgap1	Rho GTPase	22.7233	22.13758	22.16103	20.45174	21.2809	19.67692	1.299665	-0.35361	2.952937	21.40525	1.876022	-5.30545	0.104663	0.193579	0	0
1180	sp|Q8VHG	AMOT_MO	121 kDa	0.99815	2	100%	Q8VHG2	Amot	Angiomotir	19.98924	19.4095	20.58648	19.8315	19.79393	19.24965	0.626333	-0.17017	1.422835	19.81005	1.871625	-5.31045	0.104784	0.193579	0	0
1078	sp|Q9CR78	MSD3_MO	32 kDa	0.999141	2	100%	Q9CR78	Msantd3	Myb/SANT-	17.06162	19.77194	19.20468	18.48108	18.38776	20.1465	-1.24849	-2.83945	0.342482	18.84226	-1.87272	-5.31005	0.105163	0.194016	0	0
58	sp|O55143	AT2A2_MO	115 kDa	0.999641	19	100%	O55143	Atp2a2	Sarcoplasm	24.40454	25.50284	24.36559	25.76005	24.10731	24.17567	-0.85371	-1.94399	0.236566	24.71933	-1.86864	-5.31573	0.105786	0.1949	0	0
423	sp|E9PV24	FIBA_MOU	87 kDa	0.999433	6	100%	E9PV24	Fga	Fibrinogen	24.08215	25.11773	22.61217	24.17888	24.01419	24.22623	-0.93811	-2.13696	0.260739	24.03856	-1.86741	-5.31743	0.105974	0.19498	0	0
68	sp|P50446	K2C6A_MO	59 kDa	0.999228	18	22%	P50446	Krt6a	Keratin, typ	19.02408	21.00874	21.5277	22.14506	21.24919	23.36931	-1.57405	-3.58756	0.439465	21.38735	-1.86558	-5.31998	0.106254	0.195232	0	0
829	sp|Q9R0U0	SRS10_MO	31 kDa	0.999358	3	100%	Q9R0U0	Srsf10	Serine/argi	22.44571	21.7926	22.13316	22.21904	22.70403	21.46182	0.603146	-0.16847	1.374765	22.12606	1.860458	-5.32602	0.106497	0.195412	0	0
743	sp|Q61187	TS101_MO	44 kDa	0.997956	4	100%	Q61187	Tsg101	Tumor susc	20.45914	22.6445	19.68307	20.46677	20.51144	20.91239	-1.12334	-2.56695	0.320278	20.77955	-1.85699	-5.33191	0.107582	0.197136	0	0
200	sp|Q9WTI7	MYO1C_M	122 kDa	0.999458	10	100%	Q9WTI7	Myo1c	Unconvent	22.21048	21.46957	21.66281	20.82646	21.94149	21.65874	0.620004	-0.17962	1.41963	21.62826	1.845474	-5.34686	0.108838	0.199169	0	0
584	sp|P17742	PPIA_MOU	18 kDa	0.997028	5	100%	P17742	Ppia	Peptidyl-pr	23.37981	22.72229	22.4707	23.99901	22.3828	24.41963	-0.9692	-2.2225	0.284091	23.22904	-1.84549	-5.34786	0.109383	0.199897	0	0
1257	sp|Q8BH24	TM9S4_MO	75 kDa	0.996566	2	100%	Q8BH24	Tm9sf4	Transmemb	17.17198	14.69957	18.04486	16.30367	17.3162	17.56562	1.321395	-0.38945	3.032236	16.85032	1.843205	-5.35103	0.109745	0.200289	0	0
586	sp|Q9R1C7	PR40A_MO	108 kDa	0.996922	5	100%	Q9R1C7	Prpf40a	Pre-mRNA-	20.39896	20.29174	20.55306	19.57126	20.89738	20.21221	0.591395	-0.17685	1.359639	20.32077	1.832226	-5.36525	0.110949	0.202009	0	0
403.1	sp|C0HKD8	MFA1A_M	52 kDa	0.999627	6	100%	C0HKD8	Mfap1a	Microfibril	22.26411	21.18953	21.34933	21.40389	22.88534	21.54029	0.788361	-0.2372	1.813919	21.77208	1.83449	-5.36309	0.111135	0.202009	0	0
403.2	sp|C0HKD9	MFA1B_M	52 kDa	0.999627	6	100%	C0HKD9	Mfap1b	Microfibril	22.26411	21.18953	21.34933	21.40389	22.88534	21.54029	0.788361	-0.2372	1.813919	21.77208	1.83449	-5.36309	0.111135	0.202009	0	0
332	sp|Q61990	PCBP2_MO	38 kDa	0.999483	7	57%	Q61990	Pcbp2	Poly(rC)-bi	23.73992	23.27985	23.70337	23.15774	23.41051	22.93915	0.492353	-0.15059	1.135295	23.37176	1.822656	-5.3785	0.112499	0.204215	0	0
756	sp|Q9JKY5	HIP1R_MO	119 kDa	0.997556	4	100%	Q9JKY5	Hip1r	Huntingtin	22.81746	22.1869	22.6072	22.54227	22.95581	22.14697	0.501441	-0.15502	1.157903	22.54277	1.818074	-5.38483	0.113249	0.205088	0	0
565	sp|Q6ZWV	RL35_MOU	15 kDa	0.998603	5	100%	Q6ZWV7	Rpl35	60S riboso	22.7477	23.54159	23.15752	23.56501	22.9289	23.22948	-0.50066	-1.15635	0.15504	23.19503	-1.81735	-5.38584	0.113368	0.205088	0	0
135	sp|P21619	LMNB2_M	67 kDa	0.99944	13	92%	P21619	Lmnb2	Lamin-B2	23.36068	24.06857	23.58733	23.98207	23.68842	23.99327	-0.46916	-1.08375	0.145422	23.78006	-1.81695	-5.38639	0.113434	0.205088	0	0
416	sp|Q02053	UBA1_MO	118 kDa	0.99956	6	100%	Q02053	Uba1	Ubiquitin-l	21.0104	22.86488	22.57702	22.02513	21.44343	22.76394	-0.87436	-2.02318	0.274455	22.11413	-1.81631	-5.38818	0.114088	0.205997	0	0
1030.2	tr|A0A075	A0A075B5	13 kDa	0.999544	2	100%	A0A075B5	Igkv17-121	Immunoglo	21.3192	21.76438	21.22886	21.73695	21.26646	21.65953	-0.44878	-1.03994	0.142378	21.4959	-1.80689	-5.40028	0.115099	0.207546	0	0
866	sp|Q9CQ62	DECR_MOU	36 kDa	0.999025	3	100%	Q9CQ62	Decr1	2,4-dienoy	20.71805	19.86758	21.12629	20.55935	20.62873	20.34668	0.566492	-0.18323	1.316215	20.54112	1.798429	-5.41194	0.116517	0.209823	0	0
1094	sp|Q52KI8	SRRM1_M	107 kDa	0.99901	2	100%	Q52KI8	Srrm1	Serine/argi	17.7902	19.55372	19.21977	19.57931	19.4818	19.97399	-0.87175	-2.0303	0.286805	19.26646	-1.79567	-5.41659	0.117536	0.211148	0	0
358	sp|O54734	OST48_MO	49 kDa	0.999154	7	100%	O54734	Ddost	Dolichyl-di	23.30466	23.23761	23.62532	22.90064	23.32284	22.39744	0.572374	-0.18863	1.333376	23.13142	1.790175	-5.4233	0.117917	0.211148	0	0
1225	sp|O35326	SRSF5_MO	31 kDa	0.997565	2	50%	O35326	Srsf5	Serine/argi	20.6504	20.17127	19.96271	19.11824	20.30169	19.62527	0.666673	-0.22021	1.553556	19.9716	1.792008	-5.42128	0.117938	0.211148	0	0
1226	sp|Q925I1	ATAD3_MO	67 kDa	0.997556	2	100%	Q925I1	Atad3	ATPase fam	20.6504	20.17127	19.96271	19.11824	20.30169	19.62527	0.666673	-0.22021	1.553556	19.9716	1.792008	-5.42128	0.117938	0.211148	0	0
467	sp|P63154	CRNL1_MO	83 kDa	0.998287	6	100%	P63154	Crnkl1	Crooked ne	21.55484	19.96839	20.45266	20.71832	21.73213	20.69854	0.784789	-0.25989	1.829471	20.85415	1.792749	-5.42059	0.118031	0.211148	0	0
968	sp|Q99MR	ACL6B_MO	47 kDa	0.997607	3	67%	Q99MR0	Actl6b	Actin-like p	20.41374	19.47328	20.52814	19.83619	19.86589	19.85308	0.548409	-0.18283	1.279649	19.99505	1.785029	-5.43037	0.118798	0.212241	0	0
250	sp|P11276	FINC_MOU	273 kDa	0.998514	9	100%	P11276	Fn1	Fibronectin	21.63618	22.45669	21.69182	22.90146	21.75908	21.68862	-0.65323	-1.53032	0.223865	22.02231	-1.77547	-5.44396	0.120786	0.215508	0	0
1174	sp|Q9D832	DNJB4_MO	38 kDa	0.998188	2	100%	Q9D832	Dnajb4	DnaJ homo	17.64287	17.58509	19.29275	18.16608	18.72357	17.32004	0.862661	-0.29839	2.02371	18.12173	1.773129	-5.44747	0.121414	0.216343	0	0
902	sp|Q8BRF7	SCFD1_MO	72 kDa	0.998675	3	100%	Q8BRF7	Scfd1	Sec1 family	20.57609	20.29953	21.49423	20.33285	20.81199	20.5077	0.580742	-0.20093	1.362417	20.6704	1.76831	-5.4533	0.121705	0.216386	0	0
634	sp|Q6DFW	NOP58_M	60 kDa	0.999422	4	100%	Q6DFW4	Nop58	Nucleolar p	19.71475	18.06997	19.89668	18.17703	20.23419	20.29432	1.10143	-0.38262	2.585477	19.39782	1.771169	-5.45015	0.121757	0.216386	0	0
893	sp|Q8BKT7	THOC5_M	79 kDa	0.998758	3	100%	Q8BKT7	Thoc5	THO compl	18.8306	17.41741	17.07995	15.33539	19.42075	17.99951	1.526329	-0.53378	3.586435	17.6806	1.76811	-5.45433	0.122294	0.21699	0	0
937	sp|Q9QZD9	EIF3I_MOU	36 kDa	0.998119	3	100%	Q9QZD9	Eif3i	Eukaryotic	18.84203	18.23063	19.08623	18.46689	18.65219	18.44887	0.478018	-0.16686	1.122898	18.62114	1.764277	-5.45882	0.122417	0.21699	0	0
506	sp|Q60931	VDAC3_M	31 kDa	0.999502	5	100%	Q60931	Vdac3	Voltage-de	22.41538	21.37195	22.1468	21.35602	22.03295	22.13765	0.576504	-0.20395	1.356958	21.91013	1.758149	-5.4672	0.123505	0.218634	0	0
335	sp|Q9R0Q1	SYTL4_MO	76 kDa	0.999466	7	100%	Q9R0Q1	Sytl4	Synaptotag	21.71318	22.30569	21.82421	22.07671	21.96402	22.56567	-0.48222	-1.13654	0.1721	22.07491	-1.7541	-5.47272	0.124229	0.219628	0	0
930.2	sp|P63001	RAC1_MO	21 kDa	0.998224	3	100%	P63001	Rac1	Ras-related	19.77637	20.1645	19.79768	20.15404	19.59646	20.1729	-0.44031	-1.0383	0.157675	19.94366	-1.75255	-5.47485	0.124509	0.219836	0	0
1281	sp|Q8C5N3	CWC22_M	105 kDa	0.995614	2	100%	Q8C5N3	Cwc22	Pre-mRNA-	21.6343	22.10314	20.99153	21.64782	21.26518	21.73397	-0.5313	-1.25582	0.193215	21.56266	-1.7454	-5.4846	0.125801	0.221768	0	0
988	sp|Q9EQZ6	RPGF4_MO	115 kDa	0.996858	3	100%	Q9EQZ6	Rapgef4	Rap guanin	18.6788	18.85052	18.92413	21.09611	19.87571	21.20583	-1.22461	-2.89674	0.447525	19.77185	-1.74774	-5.48209	0.12593	0.221768	0	0
380	sp|Q5XJY5	COPD_MO	57 kDa	0.998264	7	100%	Q5XJY5	Arcn1	Coatomer s	21.91961	22.57273	21.47775	22.50547	21.56178	21.68752	-0.6022	-1.42422	0.219825	21.95414	-1.74372	-5.48689	0.126115	0.221806	0	0
1002.1	sp|J3QMK6	AL3B3_MO	53 kDa	0.995458	3	100%	J3QMK6	Aldh3b3	Aldehyde d	22.21939	22.81456	21.61647	22.09614	22.11875	22.81778	-0.59129	-1.39911	0.216523	22.28052	-1.74226	-5.48889	0.126382	0.221988	0	0
709	sp|P09405	NUCL_MOU	77 kDa	0.998826	4	100%	P09405	Ncl	Nucleolin	21.14682	21.73915	20.82734	21.32898	21.34954	21.79336	-0.5126	-1.21465	0.189454	21.3642	-1.73783	-5.49489	0.127181	0.223101	0	0
1077	sp|Q6NZR2	MSD2_MO	61 kDa	0.999153	2	100%	Q6NZR2	Msantd2	Myb/SANT-	20.39327	21.18038	20.69604	21.30296	20.7497	20.82728	-0.49054	-1.16355	0.182477	20.85827	-1.7348	-5.49902	0.127739	0.223685	0	0
558	sp|P11352	GPX1_MO	22 kDa	0.99885	5	100%	P11352	Gpx1	Glutathion	23.09901	21.90279	21.44827	21.09699	22.18951	21.22025	0.838921	-0.3135	1.99134	21.82614	1.737246	-5.49634	0.127843	0.223685	0	0
708	sp|P06745	G6PI_MOU	63 kDa	0.998826	4	100%	P06745	Gpi	Glucose-6-	20.75272	20.49471	21.15124	20.06202	21.24761	20.89434	0.566832	-0.21646	1.35012	20.76711	1.722398	-5.51586	0.130043	0.227241	0	0
94	sp|P99024	TBB5_MOU	50 kDa	0.999508	16	12%	P99024	Tubb5	Tubulin bet	24.32673	23.33734	23.97248	23.32587	23.15299	22.78236	0.668879	-0.26087	1.598628	23.48296	1.716852	-5.52396	0.131642	0.229738	0	0
1028	sp|Q04447	KCRB_MOU	43 kDa	0.999575	2	100%	Q04447	Ckb	Creatine ki	20.49903	20.64227	21.28593	19.82857	20.6927	20.18824	0.606192	-0.24072	1.4531	20.52279	1.705553	-5.53889	0.133474	0.232389	0	0
658	sp|Q9JJL8	SYSM_MOU	58 kDa	0.999254	4	100%	Q9JJL8	Sars2	Serine--tRN	21.71237	19.6845	19.78574	19.84282	21.36138	20.49805	0.9447	-0.37597	2.265374	20.48081	1.707062	-5.53717	0.133504	0.232389	0	0
538	sp|P62827	RAN_MOUS	24 kDa	0.99919	5	100%	P62827	Ran	GTP-bindin	24.67969	24.23354	24.92683	24.34232	24.72777	24.44456	0.437956	-0.17475	1.050659	24.55912	1.701299	-5.54439	0.134056	0.233051	0	0
581	sp|Q9Z1Q2	ABHGA_M	63 kDa	0.997559	5	100%	Q9Z1Q2	Abhd16a	Phosphatid	20.92941	19.01781	21.03051	19.78409	20.58343	21.04451	0.898982	-0.3616	2.159564	20.3983	1.701888	-5.54414	0.134498	0.233519	0	0
784	sp|Q91WS	CISD1_MO	12 kDa	0.999709	3	100%	Q91WS0	Cisd1	CDGSH iron	19.06946	20.72622	20.99448	20.75499	20.37419	22.02083	-1.0213	-2.45437	0.41177	20.65669	-1.70074	-5.54569	0.13472	0.233606	0	0
103	sp|Q9ESE1	LRBA_MOU	317 kDa	0.999616	15	87%	Q9ESE1	Lrba	Lipopolysa	22.55533	22.18078	22.66058	21.9909	22.33142	22.05555	0.440037	-0.18104	1.061111	22.29576	1.686345	-5.56454	0.13697	0.236913	0	0
939	sp|Q60854	SPB6_MOU	43 kDa	0.998046	3	100%	Q60854	Serpinb6	Serpin B6	20.36318	19.72682	20.19545	20.32601	20.44886	18.83408	0.706861	-0.2918	1.705522	19.9824	1.689142	-5.56128	0.136976	0.236913	0	0
455	sp|P27046	MA2A1_M	132 kDa	0.99896	6	100%	P27046	Man2a1	Alpha-man	21.29452	20.97972	21.1154	22.428	21.24594	22.5988	-0.78356	-1.89997	0.332854	21.61039	-1.67493	-5.58033	0.139791	0.241473	0	0
591.1	sp|P01644	KV5AB_MO	12 kDa	0.999808	4	50%	P01644	NA	Ig kappa ch	30.56848	30.99447	29.93028	30.48349	30.59641	31.45695	-0.61324	-1.48958	0.263096	30.67168	-1.66823	-5.58912	0.140917	0.24264	0	0
591.2	sp|P01645	KV5AC_MO	12 kDa	0.999808	4	50%	P01645	NA	Ig kappa ch	30.56848	30.99447	29.93028	30.48349	30.59641	31.45695	-0.61324	-1.48958	0.263096	30.67168	-1.66823	-5.58912	0.140917	0.24264	0	0
1193	sp|Q9R0P6	SC11A_MO	21 kDa	0.998027	2	100%	Q9R0P6	Sec11a	Signal pept	19.80913	19.89768	19.16466	19.66622	19.19866	20.19005	-0.52717	-1.28024	0.225906	19.6544	-1.66614	-5.59163	0.141003	0.24264	0	0

245	sp|Q9QZE5	COPG1_MO	98 kDa	0.999019	9	78%	Q9QZE5	Copg1	Coatomer s	21.82022	21.22272	22.36363	21.18759	21.36307	21.45236	0.561419	-0.24255	1.365389	21.56827	1.662133	-5.597	0.141826	0.243474	0	0
998	sp|P01636	KV5A4_MO	12 kDa	0.995948	3	33%	P01636	NA	Ig kappa ch	15.55487	16.57059	11.80174	15.69161	14.5461	14.63078	-1.66342	-4.04801	0.721159	14.79928	-1.66472	-5.59397	0.141847	0.243474	0	0
334	sp|E9Q286	ICE1_MOUS	242 kDa	0.999467	7	100%	E9Q286	Ice1	Little elong	21.68771	22.01328	20.78232	22.21347	22.00889	22.13213	-0.62666	-1.52559	0.272277	21.8063	-1.66187	-5.59761	0.142206	0.243782	0	0
648	sp|Q8BZL8	LMO3_MOU	17 kDa	0.999349	4	25%	Q8BZL8	Lmo3	LIM domain	20.00626	19.19275	20.54784	19.28175	19.32354	19.53644	0.622229	-0.27263	1.517089	19.6481	1.657637	-5.60325	0.14307	0.24488	0	0
713	sp|Q9QY76	VAPB_MOU	27 kDa	0.998753	4	75%	Q9QY76	Vapb	Vesicle-asso	22.38498	21.55169	20.56986	20.79709	22.67565	19.97025	1.103821	-0.48528	2.692922	21.32492	1.65767	-5.60338	0.143284	0.24488	0	0
320	sp|Q8CBW	ABI1_MOUS	52 kDa	0.999605	7	100%	Q8CBW3	Abi1	Abl interact	22.78899	22.29972	21.21939	19.54282	22.51208	20.89948	1.259481	-0.55427	3.073235	21.54375	1.657157	-5.60406	0.143389	0.24488	0	0
1030.1	tr|A0A075	A0A075B5J	12 kDa	0.999544	2	100%	A0A075B5J	Igkv17-127	Immunoglo	19.53636	19.24193	20.70039	19.70731	19.47109	17.9754	0.927737	-0.41117	2.266641	19.43875	1.653582	-5.60882	0.144123	0.245824	0	0
836.2	tr|A0A140	A0A140T8F	13 kDa	0.999277	3	100%	A0A140T8F	Igkv14-111	Immunoglo	24.1277	24.30344	23.1277	25.28147	23.97254	23.92815	-0.7617	-1.86681	0.343406	24.1235	-1.64487	-5.6204	0.145927	0.248589	0	0
359	sp|P19096	FAS_MOUS	272 kDa	0.999148	7	100%	P19096	Fasn	Fatty acid s	22.23164	21.60814	20.94065	22.22972	21.35763	23.94744	-1.08512	-2.66505	0.494807	22.05254	-1.63905	-5.62813	0.147144	0.250347	0	0
1069	sp|Q9JJK2	LANC2_MO	51 kDa	0.999217	2	100%	Q9JJK2	Lancl2	LanC-like p	19.56307	18.28328	20.40465	17.77966	19.43289	20.14441	1.064422	-0.48929	2.618137	19.26799	1.634906	-5.63362	0.148016	0.251515	0	0
732	sp|P97427	DPYL1_MO	62 kDa	0.9984	4	25%	P97427	Crmp1	Dihydropyr	21.39214	21.71855	22.07068	22.69001	21.04639	22.07159	-0.65698	-1.61689	0.302937	21.83156	-1.63331	-5.63573	0.148353	0.251773	0	0
954	sp|Q9Z2D6	MECP2_MO	52 kDa	0.997848	3	100%	Q9Z2D6	Mecp2	Methyl-CpG	21.28345	22.56401	21.75678	21.30964	20.97674	22.17584	-0.6775	-1.66852	0.313511	21.67774	-1.63148	-5.63816	0.14874	0.252115	0	0
682	sp|P31324	KAP3_MOU	46 kDa	0.99905	4	75%	P31324	Prkar2b	cAMP-depe	19.3267	19.26679	19.49492	18.43125	19.51251	19.09077	0.515104	-0.24099	1.2712	19.18716	1.621507	-5.65102	0.150315	0.254466	0	0
1126	sp|Q8BG51	MIRO1_MO	72 kDa	0.998666	2	100%	Q8BG51	Rhot1	Mitochond	20.20886	20.71566	20.85144	21.28954	20.24017	20.86126	-0.522	-1.28958	0.245585	20.69449	-1.61862	-5.65484	0.150938	0.255195	0	0
714	sp|P80317	TCPZ_MOU	58 kDa	0.998736	4	100%	P80317	Cct6a	T-complex	19.29978	19.64555	19.76247	20.61356	19.23408	19.79159	-0.58479	-1.4457	0.276129	19.72451	-1.61931	-5.65411	0.151122	0.255195	0	0
1221	sp|Q9D6J6	NDUV2_MO	27 kDa	0.997654	2	100%	Q9D6J6	Ndufv2	NADH dehy	22.13019	20.19584	20.13594	20.94936	22.87734	20.62026	1.126002	-0.54083	2.79283	21.15149	1.612126	-5.66371	0.152899	0.257322	0	0
280	sp|Q922B2	SYDC_MOU	57 kDa	0.999384	8	100%	Q922B2	Dars	Aspartate--	21.61051	21.51561	22.17834	21.29702	21.6656	21.24156	0.466756	-0.22352	1.157031	21.58477	1.609416	-5.66699	0.152935	0.257322	0	0
597	sp|Q8VIJ6	SFPQ_MOU	75 kDa	0.999723	4	75%	Q8VIJ6	Sfpq	Splicing fac	22.94767	22.14397	22.44565	22.7139	22.78353	21.33934	0.659882	-0.31709	1.636855	22.39568	1.611886	-5.66403	0.152951	0.257322	0	0
81	sp|Q62167	DDX3X_MO	73 kDa	0.999216	17	24%	Q62167	Ddx3x	ATP-depend	22.3851	20.92064	22.03355	21.79823	22.2409	22.12249	0.606064	-0.29485	1.506975	21.91682	1.603726	-5.67465	0.154517	0.259635	0	0
308	sp|Q3URS9	CCD51_MO	45 kDa	0.999785	7	100%	Q3URS9	Ccdc51	Coiled-coil	21.81858	19.80296	20.33623	20.88405	22.13494	20.88448	0.906089	-0.4455	2.257682	20.97688	1.599837	-5.67987	0.155595	0.2608	0	0
769	sp|P47962	RL5_MOUS	34 kDa	0.997008	4	100%	P47962	Rpl5	60S ribosor	20.29553	20.55558	20.1062	20.45196	19.40144	20.35779	-0.52072	-1.29662	0.255184	20.19475	-1.59734	-5.68288	0.155595	0.2608	0	0
1089	sp|Q9D6M	GHC1_MOU	35 kDa	0.999038	2	100%	Q9D6M3	Slc25a22	Mitochond	20.1199	19.48487	19.73123	19.403	19.61848	19.25781	0.441311	-0.21875	1.101371	19.60255	1.591337	-5.69076	0.156933	0.262717	0	0
1246	sp|P31938	MP2K1_MO	43 kDa	0.996991	2	100%	P31938	Map2k1	Dual specif	22.18774	23.36572	20.20119	22.89809	22.16394	21.60415	-1.10503	-2.76553	0.555474	22.07014	-1.58813	-5.69521	0.158206	0.264522	0	0
850	tr|A0A140	A0A140T8N	13 kDa	0.999137	3	100%	A0A140T8N	Igkv6-32	Immunoglo	21.53047	22.3488	21.3377	22.36287	22.07352	21.80139	-0.52379	-1.31132	0.263743	21.90912	-1.58303	-5.70165	0.158802	0.265191	0	0
661	sp|P10922	H10_MOUS	21 kDa	0.999226	4	100%	P10922	H1f0	Histone H1	24.15853	23.79194	23.69151	23.53611	24.62709	23.59527	0.517935	-0.26154	1.297408	23.90007	1.581521	-5.70362	0.159144	0.265436	0	0
71	sp|P05784	K1C18_MO	48 kDa	0.999757	17	82%	P05784	Krt18	Keratin, typ	24.42293	23.90281	24.39502	24.4042	25.01887	24.04625	0.494518	-0.25144	1.24048	24.36502	1.577855	-5.70841	0.159977	0.266497	0	0
1219	tr|A0A075	A0A075B5X	11 kDa	0.997683	2	50%	A0A075B5X	Ighv1-67	Immunoglo	18.28485	18.9101	19.63178	20.75041	19.16561	20.13085	-0.90304	-2.26833	0.462241	19.47893	-1.57847	-5.70783	0.16039	0.266857	0	0
1026	sp|P63242	IF5A1_MOU	17 kDa	0.999606	2	100%	P63242	Eif5a	Eukaryotic	19.69219	22.40331	21.48355	23.10251	23.93995	25.12685	-1.839	-4.62536	0.947373	22.62473	-1.57504	-5.71229	0.161171	0.267828	0	0
296	sp|P33175	KIF5A_MOU	117 kDa	0.998745	8	12%	P33175	Kif5a	Kinesin hea	20.31659	18.92504	18.20802	18.88076	20.677	18.26149	1.044776	-0.54016	2.629718	19.21148	1.573116	-5.71481	0.161612	0.268233	0	0
549	sp|Q9DBE8	ALG2_MOU	47 kDa	0.998978	5	100%	Q9DBE8	Alg2	Alpha-1,3/1	22.52353	22.30899	22.37436	21.4002	22.23581	21.89774	0.508925	-0.26412	1.281966	22.12344	1.566937	-5.72266	0.162482	0.269307	0	0
1211	sp|Q3UMB	WASC4_MO	136 kDa	0.997787	2	100%	Q3UMB9	Washc4	WASH com	14.9721	17.51921	12.97503	18.14232	17.28625	15.24019	-1.88945	-4.76407	0.985176	16.02252	-1.56857	-5.72072	0.162657	0.269307	0	0
1119.1	sp|C0HK79	ARXS1_MO	20 kDa	0.998757	2	100%	C0HK79	Arxes1	Adipocyte-	20.79483	20.16016	21.48443	20.81437	20.45198	19.88434	0.624119	-0.33068	1.578914	20.59835	1.559939	-5.73194	0.164659	0.272289	0	0
1168	sp|Q8VE97	SRSF4_MOU	56 kDa	0.998262	2	50%	Q8VE97	Srsf4	Serine/argi	19.61238	19.2232	18.76235	19.98669	19.16938	20.07829	-0.58136	-1.47177	0.309057	19.47205	-1.55649	-5.73635	0.165247	0.272413	0	0
82	sp|Q7TMM	TBB2A_MO	50 kDa	0.999055	17	6%	Q7TMM9	Tubb2a	Tubulin bet	22.94849	22.60223	23.258	22.37028	22.49043	22.2841	0.480102	-0.25483	1.215036	22.65892	1.554842	-5.73839	0.1653	0.272413	0	0
807	sp|P35922	FMR1_MOU	69 kDa	0.999463	3	33%	P35922	Fmr1	Synaptic fu	22.07779	21.50725	22.6493	21.71026	21.96177	22.00088	0.490158	-0.26038	1.240697	21.98454	1.554401	-5.73896	0.165404	0.272413	0	0
573	sp|Q6PHZ2	KCC2D_MO	56 kDa	0.998209	5	60%	Q6PHZ2	Camk2d	Calcium/ca	21.98621	23.10317	21.25374	23.33645	22.40286	21.69289	-0.8299	-2.10257	0.442771	22.29589	-1.55618	-5.73681	0.165537	0.272413	0	0
680.1	sp|P0DP26	CALM1_MO	17 kDa	0.99906	4	100%	P0DP26	Calm1	Calmodulin	21.50927	20.84945	21.26034	21.24079	21.23826	20.51891	0.466241	-0.25108	1.183559	21.10284	1.547029	-5.74852	0.167145	0.273785	0	0
680.2	sp|P0DP27	CALM2_MO	17 kDa	0.99906	4	100%	P0DP27	Calm2	Calmodulin	21.50927	20.84945	21.26034	21.24079	21.23826	20.51891	0.466241	-0.25108	1.183559	21.10284	1.547029	-5.74852	0.167145	0.273785	0	0
680.3	sp|P0DP28	CALM3_MO	17 kDa	0.99906	4	100%	P0DP28	Calm3	Calmodulin	21.50927	20.84945	21.26034	21.24079	21.23826	20.51891	0.466241	-0.25108	1.183559	21.10284	1.547029	-5.74852	0.167145	0.273785	0	0
521	sp|P50481	LHX3_MOU	44 kDa	0.999329	5	100%	P50481	Lhx3	LIM/homed	20.32543	20.08119	20.6918	17.99755	19.63035	19.75225	0.938862	-0.50738	2.385107	19.74643	1.549211	-5.74584	0.167178	0.273785	0	0
948	sp|Q922U1	PRPF3_MO	77 kDa	0.997918	3	100%	Q922U1	Prpf3	U4/U6 sma	19.899	21.03168	17.8648	20.67317	21.16724	20.87095	-1.21492	-3.0918	0.661955	20.25114	-1.54477	-5.75158	0.168232	0.275143	0	0
99	sp|Q8BMS1	ECHA_MOU	83 kDa	0.999761	15	100%	Q8BMS1	Hadha	Trifunction	23.52318	23.5922	24.0243	23.32312	23.7086	22.89657	0.481398	-0.26185	1.224646	23.51133	1.541598	-5.75554	0.168438	0.275143	0	0
55	sp|P14733	LMNB1_MO	67 kDa	0.999761	19	95%	P14733	Lmnb1	Lamin-B1	24.72842	25.12719	24.94358	25.52291	24.66242	24.99112	-0.4356	-1.10862	0.237419	24.99594	-1.5405	-5.75696	0.168701	0.275143	0	0
181	sp|E9Q557	DESP_MOU	333 kDa	0.998772	11	91%	E9Q557	Dsp	Desmoplak	26.4519	27.72025	25.8171	26.80747	27.65173	27.94761	-0.85153	-2.16928	0.466223	27.06601	-1.54211	-5.75501	0.168864	0.275143	0	0
991	sp|Q6ZWY3	RS27L_MO	9 kDa	0.996755	3	33%	Q6ZWY3	Rps27l	40S ribosor	18.43975	19.48885	19.44739	19.30052	19.01831	19.71013	-0.53135	-1.35324	0.290544	19.23416	-1.53882	-5.75914	0.169114	0.275143	0	0
339	sp|P97467	AMD_MOU	109 kDa	0.999426	7	100%	P97467	Pam	Peptidyl-gl	22.78346	22.61932	22.60715	22.17065	22.95593	22.27116	0.42847	-0.23447	1.091414	22.56794	1.538311	-5.75979	0.169225	0.275143	0	0
228	sp|P63239	NEC1_MOU	84 kDa	0.999749	9	89%	P63239	Pcsk1	Neuroendo	23.54351	23.21976	23.43057	22.859	23.50683	23.18907	0.40436	-0.22185	1.030567	23.29146	1.536919	-5.76159	0.16956	0.275357	0	0
1171	sp|Q9JLI8	SART3_MO	110 kDa	0.998231	2	100%	Q9JLI8	Sart3	Squamous	24.53754	24.33501	24.85107	24.11704	24.42377	24.11128	0.41635	-0.22905	1.061747	24.39595	1.53544	-5.7635	0.169915	0.275604	0	0
761	sp|P48453	PP2BB_MO	59 kDa	0.997338	4	100%	P48453	Ppp3cb	Serine/thre	18.54963	18.63115	18.91097	20.08064	18.65731	19.31212	-0.63533	-1.62261	0.35194	19.02364	-1.53573	-5.76324	0.170395	0.276052	0	0
1117	sp|P47757	CAPZB_MO	31 kDa	0.998764	2	100%	P47757	Capzb	F-actin-cap	20.26947	20.08153	20.29621	19.23993	20.47194	20.16228	0.517961	-0.28701	1.322931	20.08689	1.531576	-5.76848	0.170859	0.276278	0	0
835	tr|V9GX38	V9GX38_M	38 kDa	0.999287	3	33%	V9GX38	Gm17190	Predicted g	19.21437	20.68553	19.61596	19.31395	19.52128	20.22245	-0.62344	-1.59369	0.34681	19.76226	-1.53342	-5.76621	0.170951	0.276278	0	0
1095	tr|A0A075	A0A075B5I	10 kDa	0.999003	2	50%	A0A075B5I	Igkv5-39	Immunoglo	19.70284	19.95872	20.11754	19.06697	19.98818	19.23474	0.516042	-0.28655	1.318634	19.67816	1.530351	-5.77006	0.171145	0.276278	0	0
231	sp|Q9Z315	SNUT1_MO	91 kDa	0.999674	9	100%	Q9Z315	Sart1	U4/U6.U5 t	20.94027	21.37044	20.48983	21.14614	21.17071	21.45395	-0.45657	-1.16768	0.254536	21.09522	-1.52818	-5.77285	0.171672	0.276799	0	0
666	sp|P62849	RS24_MOU	15 kDa	0.999157	4	100%	P62849	Rps24	40S ribosor	22.15371	21.20951	21.4961	20.34672	21.88396	21.91178	0.688588	-0.38846	1.76564	21.5003	1.525715	-5.77612	0.172822	0.278112	0	0
1235	sp|Q02248	CTNB1_MO	85 kDa	0.997288	2	100%	Q02248	Ctnnb1	Catenin bet	18.0649	16.35402	18.39154	18.53567	17.51481	15.79664	1.09497	-0.61902	2.808956	17.44293	1.524564	-5.7776	0.173102	0.278112	0	0
1236	sp|Q7TMY8	HUWE1_M	483 kDa	0.997286	2	100%	Q7TMY8	Huwe1	E3 ubiquiti	18.0649	16.35402	18.39154	18.53567	17.51481	15.79664	1.09497	-0.61902	2.808956	17.44293	1.524564	-5.7776	0.173102	0.278112	0	0
742	sp|Q8BGC4	PTGR3_MO	41 kDa	0.997995	4	100%	Q8BGC4	Zadh2	Prostagland	20.78401	22.5345	21.10698	20.55711	20.97627	22.24272	-0.82236	-2.11124	0.466515	21.36693	-1.52266	-5.78005	0.173569	0.278266	0	0
134	tr|F8VQC1	F8VQC1_M	75 kDa	0.999512	13	100%	F8VQC1	Srp72	Signal reco	23.30278	28.82273	23.30846	22.81869	23.55645	24.85482	-2.10952	-5.41609	1.197052	24.44399	-1.5225	-5.78026	0.173608	0.278266	0	0
533	sp|Q3UEB3	PUF60_MO	60 kDa	0.99922	5	100%	Q3UEB3	Puf60	Poly(U)-bin	21.10803	22.10785	20.97972	20.94478	21.21398	21.97337	-0.57476	-1.47611	0.326596	21.38795	-1.52014	-5.78324	0.173969	0.278515	0	0
1131	sp|Q9JKX6	NUDT5_MO	24 kDa	0.99861	2	100%	Q9JKX6	Nudt5	ADP-sugar	20.77212	19.03802	21.93863	21.74438	21.59847	20.32745	1.066458	-0.61433	2.747243	20.90318	1.514197	-5.79089	0.175652	0.280546	0	0
1132	sp|P67871	CSK2B_MO	25 kDa	0.998588	2	100%	P67871	Csnk2b	Casein kina	20.77212	19.03802	21.93863	21.74438	21.59847	20.32745	1.066458	-0.61433	2.747243	20.90318	1.514197	-5.79089	0.175652	0.280546	0	0
145	sp|P52480	KPYM_MOU	58 kDa	0.99964	12	100%	P52480	Pkm	Pyruvate ki	24.438	24.67056	24.85356	25.20332	24.62281	25.48808	-0.48253	-1.24671	0.281638	24.87939	-1.50293	-5.80523	0.177916	0.283828	0	0
1189	sp|O89051	ITM2B_MO	30 kDa	0.998086	2	100%	O89051	Itm2b	Integral me	17.42239	17.98042	18.1624	12.28564	17.16919	16.38427	2.03455	-1.19426	5.263359	16.56738	1.503754	-5.80423	0.178255	0.283854	0	0
799	sp|Q9CX86	ROA0_MOU	31 kDa	0.999526	3	100%	Q9CX86	Hnrnpa0	Heterogene	20.31193	22.76135	23.80267	25.02931	19.66552	21.72003	-1.91019	-4.94357	1.123187	22.21513	-1.5028	-5.80545	0.178494	0.283854	0	0
445	sp|P57780	ACTN4_MO	105 kDa	0.999129	6	100%	P57780	Actn4	Alpha-actin	21.12255	20.96159	20.70941	21.58629	20.60384	21.27276	-0.46161	-1.19389	0.270672	21.04274	-1.50037	-5.8085	0.17856	0.283854	0	0
6	sp|Q9JHU4	DYHC1_MO	532 kDa	0.999666	50	100%	Q9JHU4	Dync1h1	Cytoplasmi	25.49044	26.32785	25.53898	25.23382	25.2618	26.5393	-0.60325	-1.56291	0.356414	25.73203	-1.50013	-5.80885	0.179166	0.283972	0	0
1051	sp|P58281	OPA1_MOU	111 kDa	0.999313	2	100%	P58281	Opa1	Dynamin-li	23.23577	22.40208	22.09365	22.37312	23.05695	21.84288	0.589432	-0.34849	1.527354	22.50074	1.499748	-5.80933	0.179262	0.283972	0	0
1052	sp|Q9R0P9	UCHL1_MO	25 kDa	0.99931	2	100%	Q9R0P9	Uchl1	Ubiquitin c	23.23577	22.40208	22.09365	22.37312	23.05695	21.84288	0.589432	-0.34849	1.527354	22.50074	1.499748	-5.80933	0.179262	0.283972	0	0
1173	sp|P55249	LX12E_MO	75 kDa	0.998211	2	100%	P55249	Alox12e	Arachidona	18.10271	17.45414	19.11794	18.08479	17.63845	17.2586	0.68719	-0.40822	1.782596	17.94277	1.497106	-5.8127	0.17993	0.284697	0	0
768	sp|P28660	NCKP1_MO	129 kDa	0.997017	4	100%	P28660	Nckap1	Nck-associa	21.67127	20.4981	21.94754	21.20502	21.66573	21.82739	0.584678	-0.34827	1.517631	21.46918	1.495574	-5.81465	0.180318	0.284954	0	0
525	sp|P35455	NEU2_MOU	18 kDa	0.999285	5	80%	P35455	Avp	Vasopressin	19.70739	25.82576	22.65475	23.37696	23.98801	22.72488	-1.85915	-4.82726	1.10896	23.04629	-1.49481	-5.81562	0.180513	0.284954	0	0
942	sp|P01668	KV3AG_MO	12 kDa	0.998019	3	33%	P01668	NA	Ig kappa ch	16.2335	16.01487	16.56614	19.27991	17.00409	18.07093	-1.18732	-3.08422	0.709571	17.19491	-1.49374	-5.81697	0.180783	0.285048	0	0
752	sp|Q8BW4	PMGT2_MO	69 kDa	0.997653	4	100%	Q8BW41	Pomgnt2	Protein O-l	19.13876	19.83357	19.25925	21.29196	18.27615	18.62164	-1.02434	-2.66515	0.616473	19.40355	-1.48983	-5.82195	0.181781	0.28629	0	0
683	sp|P35293	RAB18_MO	23 kDa	0.999044	4	75%	P35293	Rab18	Ras-related	20.96792	21.09972	20.77681	21.18074	20.53618	21.18275	-0.3941	-1.02538	0.237174	20.95735	-1.4859	-5.82691	0.182242	0.286396	0	0
188	sp|Q7TPV4	MBB1A_MO	152 kDa	0.999675	10	100%	Q7TPV4	Mybbp1a	Myb-bindin	22.38563	22.14493	22.77721	22.26044	22.49628	22.07004	0.394568	-0.2375	1.026638	22.35576	1.485788	-5.82705	0.182271	0.286396	0	0
1063.2	tr|A0A075	A0A075B6A	37 kDa	0.999242	2	100%	A0A075B6A	Igha	Immunoglo	22.16911	21.41782	22.21547	21.74372	21.77535	21.74489	0.417832	-0.25194	1.087601	21.84439	1.484834	-5.82827	0.182516	0.286449	0	0
493	tr|A0A075	A0A075B5I	10 kDa	0.999661	5	40%	A0A075B5I	Igkv6-13	Immunoglo	23.9316	26.1743	21.65218	23.36178	21.12807	22.27183	-1.69869	-4.42912	1.031741	23.08663	-1.48468	-5.82847	0.183099	0.287032	0	0
454	sp|Q9JKF1	IQGA1_MO	189 kDa	0.998976	6	100%	Q9JKF1	Iqgap1	Ras GTPase	20.51589	21.37397	20.64323	20.4434	19.80473	20.8257	-0.55974	-1.46383	0.34435	20.60115	-1.47594	-5.83954	0.185145	0.289903	0	0
868	sp|O09106	HDAC1_MO	55 kDa	0.998995	3	100%	O09106	Hdac1	Histone dea	20.94364	19.98263	19.54545	19.88414	20.646	19.32672	0.647203	-0.39984	1.69425	20.05476	1.475114	-5.84058	0.185576	0.290243	0	0
1023	sp|Q922V4	PLRG1_MO	57 kDa	0.999706	2	100%	Q922V4	Plrg1	Pleiotropic	20.29109	20.47224	19.97076	21.5589	19.66128	19.93901	-0.68234	-1.78896	0.424269	20.31554	-1.47149	-5.84515	0.18652	0.291384	0	0
665	sp|Q8R4X3	RBM12_MO	103 kDa	0.999174	4	100%	Q8R4X3	Rbm12	RNA-bindin	21.42093	22.19443	20.42327	21.16665	21.4883	21.87072	-0.6331	-1.66062	0.394418	21.42738	-1.47039	-5.84654	0.186808	0.291499	0	0
404	sp|Q9JIF7	COPB_MOU	107 kDa	0.999621	6	100%	Q9JIF7	Copb1	Coatomer s	21.62258	21.66188	22.07055	20.78293	21.48591	21.22945	0.501593	-0.31278	1.315963	21.47555	1.466061	-5.85202	0.187414	0.292018	0	0
78	sp|P29341	PABP1_MO	71 kDa	0.999579	17	88%	P29341	Pabpc1	Polyadenyl	25.00281	21.99944	23.58065	21.92393	24.69661	25.22812	1.37619	-0.86179	3.61417	23.73859	1.467483	-5.85021	0.187572	0.292018	0	0
1253	sp|Q99K01	PDXD1_MO	87 kDa	0.996729	2	100%	Q99K01	Pdxdc1	Pyridoxal-d	19.63986	19.35901	19.33071	20.2351	19.73239	23.0122	-1.30112	-3.42569	0.823451	20.21821	-1.46149	-5.85775	0.189153	0.294141	0	0
1181	sp|P15508	SPTB1_MO	245 kDa	0.998147	2	100%	P15508	Sptb	Spectrin be	20.9722	20.28426	21.14096	20.10071	20.66647	20.94354	0.483705	-0.30819	1.275604	20.68469	1.453824	-5.86742	0.190654	0.296136	0	0
531	sp|Q7TN33	CELF6_MOU	48 kDa	0.999224	5	100%	Q7TN33	Celf6	CUGBP Elav	22.46618	22.44068	21.56423	22.07976	21.39844	22.51871	-0.53677	-1.41692	0.343383	22.078	-1.45386	-5.86734	0.19097	0.296287	0	0
383	sp|Q6PH08	ERC2_MOU	111 kDa	0.998165	7	100%	Q6PH08	Erc2	ERC protein	19.38581	19.82916	18.82525	21.31985	21.08043	21.08828	-0.98193	-2.60431	0.640441	20.2548	-1.44438	-5.8792	0.193736	0.299975	0	0
427	sp|P08752	GNAI2_MO	40 kDa	0.999401	6	50%	P08752	Gnai2	Guanine nu	20.31076	20.35625	21.4175	20.17157	20.58204	20.23514	0.515778	-0.336	1.367553	20.51221	1.443223	-5.88067	0.193789	0.299975	0	0
262	sp|Q91ZR2	SNX18_MO	68 kDa	0.999726	8	100%	Q91ZR2	Snx18	Sorting nex	25.12914	22.76566	23.16367	22.64179	25.67968	24.90054	1.221498	-0.81592	3.258911	24.04675	1.430751	-5.89619	0.197457	0.305305	0	0
822	sp|Q9D358	PPAC_MOU	18 kDa	0.999395	3	100%	Q9D358	Acp1	Low molecu	20.3845	21.89027	19.23984	20.2441	17.99841	19.13001	-1.21388	-3.24866	0.820911	19.81452	-1.42366	-5.90499	0.199419	0.307987	0	0
598	sp|Q9DB77	QCR2_MOU	48 kDa	0.999719	4	100%	Q9DB77	Uqcrc2	Cytochrom	20.6501	21.16469	21.97187	22.2635	20.61804	21.90472	-0.69763	-1.86898	0.473716	21.42882	-1.42132	-5.9079	0.200071	0.308642	0	0
818	sp|Q9R1Q9	VAS1_MOU	51 kDa	0.999414	3	100%	Q9R1Q9	Atp6ap1	V-type prot	20.97313	21.84734	21.39982	22.21377	20.63937	20.82034	-0.62304	-1.66982	0.423733	21.31563	-1.42041	-5.90902	0.200323	0.30868	0	0
1103	sp|P28658	ATX10_MO	54 kDa	0.998947	2	100%	P28658	Atxn10	Ataxin-10	20.16387	21.32252	21.62698	20.57814	19.67945	22.24518	-0.89184	-2.407	0.623311	20.93602	-1.4047	-5.92843	0.204752	0.315146	0	0
943	sp|P35283	RAB12_MO	27 kDa	0.998013	3	67%	P35283	Rab12	Ras-related	19.41794	19.38581	19.9271	18.94686	19.13382	18.7218	0.474797	-0.33195	1.281539	19.25555	1.40086	-5.93327	0.205324	0.315578	0	0
1070	sp|Q921K9	BCL7B_MO	22 kDa	0.999205	2	50%	Q921K9	Bcl7b	B-cell CLL/l	17.47632	18.15401	18.98205	18.70716	17.57501	19.38307	-0.73696	-1.99131	0.517395	18.3796	-1.40208	-5.93165	0.205498	0.315578	0	0
516	sp|O88569	ROA2_MOU	37 kDa	0.999369	5	100%	O88569	Hnrnpa2b1	Heterogene	22.61404	22.24579	22.23639	22.64133	23.21183	21.26329	0.637284	-0.45107	1.725638	22.36878	1.397378	-5.93743	0.206844	0.317286	0	0
568	sp|P62317	SMD2_MOU	14 kDa	0.998349	5	100%	P62317	Snrpd2	Small nucle	24.09942	24.53585	23.38422	24.60029	24.5672	24.43173	-0.50568	-1.37111	0.359758	24.26979	-1.39294	-5.94293	0.207911	0.318563	0	0
362	sp|P13020	GELS_MOU	86 kDa	0.999091	7	100%	P13020	Gsn	Gelsolin	21.74806	22.1444	22.07291	22.40759	21.85665	22.22665	-0.36701	-0.99512	0.261108	22.07605	-1.39071	-5.94575	0.208237	0.318701	0	0
1209	sp|Q7TT50	MRCKB_MO	195 kDa	0.997802	2	100%	Q7TT50	Cdc42bpb	Serine/thre	15.95387	16.43879	17.26305	16.34319	16.7925	15.13228	0.698387	-0.50136	1.898139	16.32062	1.389171	-5.94749	0.209213	0.319834	0	0
684	sp|O70152	DPM1_MO	29 kDa	0.999033	4	100%	O70152	Dpm1	Dolichol-ph	21.71079	21.51958	21.43645	22.1934	21.49215	22.20897	-0.42752	-1.16226	0.307224	21.76022	-1.38491	-5.95285	0.209924	0.32056	0	0
704	sp|Q9JIS5	SV2A_MOU	83 kDa	0.998858	4	100%	Q9JIS5	Sv2a	Synaptic ve	22.04964	21.73632	22.53426	22.16459	22.27226	21.75317	0.400694	-0.28846	1.089852	22.08504	1.383869	-5.95412	0.210227	0.320662	0	0
1274	sp|E9Q3L2	PI4KA_MOU	237 kDa	0.995953	2	100%	E9Q3L2	Pi4ka	Phosphatid	19.80407	19.23859	18.72606	17.14825	19.66021	19.16098	0.880835	-0.64237	2.404038	18.95636	1.380027	-5.95866	0.211881	0.322467	0	0
1054	sp|Q80UU9	PGRC2_MO	23 kDa	0.99929	2	50%	Q80UU9	Pgrmc2	Membrane	18.21978	17.55232	19.34481	14.31366	17.49705	18.60673	1.529647	-1.11556	4.174858	17.58906	1.380008	-5.95868	0.211887	0.322467	0	0
933	sp|P01592	IGJ_MOUSE	18 kDa	0.998193	3	100%	P01592	Jchain	Immunoglo	22.27519	22.79066	22.75113	22.5666	21.48053	22.70681	-0.51908	-1.42437	0.386217	22.42849	-1.3669	-5.9747	0.215556	0.327613	0	0
173	sp|Q8BKI2	TNR6B_MO	192 kDa	0.999298	11	100%	Q8BKI2	Tnrc6b	Trinucleoti	22.3601	21.52462	21.34639	20.98332	22.02357	21.68924	0.51096	-0.3806	1.402523	21.65454	1.366245	-5.97549	0.215751	0.327613	0	0
707	sp|Q6NZL0	SOGA3_MO	103 kDa	0.998834	4	50%	Q6NZL0	Soga3	Protein SOG	18.26011	14.90411	17.94117	17.57236	18.01283	18.30415	1.144496	-0.85604	3.145028	17.49912	1.365272	-5.9766	0.21625	0.328003	0	0
444	sp|Q60605	MYL6_MOU	17 kDa	0.999136	6	83%	Q60605	Myl6	Myosin ligh	23.07272	23.44481	22.62557	22.88893	22.75131	23.32471	-0.40295	-1.10892	0.303017	23.01801	-1.35853	-5.98495	0.217749	0.329908	0	0
570	sp|O35474	EDIL3_MOU	54 kDa	0.998321	5	100%	O35474	Edil3	EGF-like rep	21.17285	22.25191	22.40025	23.03696	22.31698	22.36921	-0.58933	-1.62668	0.448015	22.25803	-1.35577	-5.98809	0.219105	0.331591	0	0
952	sp|Q9CY66	GAR1_MOU	23 kDa	0.997891	3	100%	Q9CY66	Gar1	H/ACA ribo	19.33262	20.28371	19.13859	19.31163	19.57088	19.8274	-0.46021	-1.27234	0.351917	19.57747	-1.34882	-5.99667	0.220702	0.333555	0	0
106	sp|Q9D6F9	TBB4A_MO	50 kDa	0.999582	15	13%	Q9D6F9	Tubb4a	Tubulin bet	21.58144	21.19593	21.18172	20.21757	20.72466	20.52791	0.51547	-0.39534	1.42628	20.90487	1.349211	-5.99608	0.220894	0.333555	0	0
775	tr|E9PUQ3	E9PUQ3_M	34 kDa	0.995543	4	100%	E9PUQ3	Nkapd1	NKAP doma	15.11682	17.07619	15.6913	NA	14.82018	15.12651	-0.89191	-2.50394	0.72012	15.5662	-1.37145	-5.85894	0.22179	0.334374	0	0
890	sp|Q9DCC4	P5CR3_MO	29 kDa	0.998784	3	100%	Q9DCC4	Pycr3	Pyrroline-5	17.19474	15.69695	15.76588	18.78786	16.91062	19.25783	-1.29047	-3.57763	0.996696	17.26898	-1.34648	-5.99929	0.22193	0.334374	0	0
1050	tr|A0A075	A0A075B6I	13 kDa	0.999313	2	100%	A0A075B6I	Igkv19-93	Immunoglo	14.4726	14.52237	15.42281	16.51854	12.29857	NA	-1.45579	-4.08993	1.178343	14.64698	-1.36991	-5.86077	0.222241	0.334472	0	0
1146	sp|Q62425	NDUA4_MO	9 kDa	0.998505	2	100%	Q62425	Ndufa4	Cytochrom	19.29007	20.39892	20.14955	20.29324	19.23964	19.56314	-0.52535	-1.45851	0.407815	19.82243	-1.34351	-6.00287	0.222841	0.335003	0	0
447	sp|P14131	RS16_MOU	16 kDa	0.999093	6	100%	P14131	Rps16	40S ribosor	24.59531	25.3173	24.80137	22.79347	24.28328	22.67738	0.963933	-0.75061	2.678475	24.07802	1.341681	-6.00506	0.223402	0.335474	0	0
664	sp|Q9D6Z1	NOP56_MO	64 kDa	0.999188	4	100%	Q9D6Z1	Nop56	Nucleolar p	20.08395	19.86843	20.11259	19.96428	20.09317	19.26276	0.398075	-0.31275	1.108898	19.89753	1.332922	-6.01577	0.225591	0.338386	0	0
249	tr|O35452	O35452_M	435 kDa	0.998622	9	100%	O35452	Tnxb	Tenascin X	20.15781	19.76681	19.0807	19.38652	20.4381	18.59189	0.643793	-0.50911	1.796694	19.57031	1.332617	-6.01592	0.226204	0.338932	0	0
763	sp|Q3UL36	ARGL1_MO	33 kDa	0.99725	4	100%	Q3UL36	Arglu1	Arginine an	20.71566	20.34174	20.5412	19.3493	20.2237	20.31957	0.489988	-0.3891	1.369076	20.24853	1.328763	-6.02061	0.2272	0.340048	0	0
54	sp|Q6NZJ6	IF4G1_MOU	176 kDa	0.999849	19	89%	Q6NZJ6	Eif4g1	Eukaryotic	24.22671	25.37446	23.71419	23.7381	24.68741	25.90715	-0.79714	-2.23128	0.637009	24.608	-1.32645	-6.02329	0.22813	0.340699	0	0
1283	sp|Q61011	GBB3_MOU	37 kDa	0.995547	2	50%	Q61011	Gnb3	Guanine nu	NA	15.81033	16.70831	17.15926	18.45037	16.99706	0.923794	-0.77224	2.619823	17.02507	1.350121	-5.88427	0.228137	0.340699	0	0
458	sp|P21661	NEC2_MOU	71 kDa	0.998953	6	83%	P21661	Pcsk2	Neuroendo	20.94724	20.62929	20.81091	20.11026	21.40032	21.03659	0.460778	-0.36991	1.291465	20.82244	1.320311	-6.03082	0.229553	0.342435	0	0
1237	tr|Q9Z1A1	Q9Z1A1_M	43 kDa	0.997261	2	100%	Q9Z1A1	Tfg	TFG protein	18.08519	17.72852	17.96482	17.15726	18.02598	17.97065	0.40652	-0.32981	1.142852	17.82207	1.31404	-6.03828	0.231531	0.345007	0	0
1099	sp|P19157	GSTP1_MO	24 kDa	0.998964	2	100%	P19157	Gstp1	Glutathion	20.60479	20.60859	20.79604	20.62818	20.1613	22.90224	-0.85896	-2.41813	0.700207	20.95019	-1.31471	-6.03724	0.231832	0.345076	0	0
922	sp|Q99MJ9	DDX50_MO	82 kDa	0.998358	3	100%	Q99MJ9	Ddx50	ATP-depend	22.13603	22.16664	23.14478	21.84751	22.00701	21.77412	0.499853	-0.40967	1.409377	22.17935	1.310186	-6.04271	0.233075	0.346032	0	0
923	sp|P63276	RS17_MOU	16 kDa	0.998349	3	100%	P63276	Rps17	40S ribosor	22.13603	22.16664	23.14478	21.84751	22.00701	21.77412	0.499853	-0.40967	1.409377	22.17935	1.310186	-6.04271	0.233075	0.346032	0	0
839	sp|Q60668	HNRPD_MO	38 kDa	0.999248	3	67%	Q60668	Hnrnpd	Heterogene	19.12274	19.24007	18.46981	19.39219	19.46803	19.84987	-0.47385	-1.33638	0.388686	19.25712	-1.30966	-6.04333	0.23324	0.346032	0	0
1204	sp|Q99KL7	RAB28_MO	25 kDa	0.997906	2	100%	Q99KL7	Rab28	Ras-related	16.11008	18.09839	16.67935	16.22784	15.26952	16.19803	-0.82177	-2.32294	0.679402	16.43053	-1.30638	-6.04711	0.234492	0.34751	0	0
728	sp|Q8BJ71	NUP93_MO	93 kDa	0.998481	4	100%	Q8BJ71	Nup93	Nuclear po	19.45614	20.75315	19.3343	19.10635	19.85234	20.75413	-0.65695	-1.86034	0.546433	19.87607	-1.30281	-6.05133	0.235642	0.348833	0	0
1085	sp|Q8K021	SCAM1_MO	38 kDa	0.999085	2	100%	Q8K021	Scamp1	Secretory c	19.68145	19.55658	18.13059	19.58311	19.35914	19.75749	-0.57533	-1.62998	0.479309	19.34473	-1.30186	-6.05245	0.235946	0.348902	0	0
736	sp|P54869	HMCS2_MO	57 kDa	0.998356	4	100%	P54869	Hmgcs2	Hydroxyme	16.87567	15.86102	16.68381	13.96195	17.54384	17.75657	1.174594	-0.98221	3.331401	16.44714	1.299652	-6.05505	0.236661	0.349577	0	0
703	sp|Q9CPR4	RL17_MOU	21 kDa	0.998861	4	100%	Q9CPR4	Rpl17	60S ribosor	23.9601	23.76732	23.98207	22.82783	23.66429	23.66947	0.447283	-0.37497	1.269532	23.64518	1.294795	-6.06103	0.237736	0.350783	0	0
1217	sp|Q64511	TOP2B_MO	182 kDa	0.997738	2	100%	Q64511	Top2b	DNA topois	17.75285	17.04339	18.51347	17.761	18.60066	18.37083	0.563923	-0.47847	1.606314	18.00703	1.291041	-6.06518	0.23946	0.352944	0	0

851	sp|P01193	COLI_MOU	27 kDa	0.999134	3	100%	P01193	Pomc	Pro-opiom	20.26525	20.81112	20.26936	20.07104	19.80083	20.70913	-0.41861	-1.19569	0.358466	20.32112	-1.28218	-6.07583	0.241865	0.355715	0	0
852	sp|Q8R3Q0	SARAF_MO	36 kDa	0.99911	3	100%	Q8R3Q0	Saraf	Store-opera	20.26525	20.81112	20.26936	20.07104	19.80083	20.70913	-0.41861	-1.19569	0.358466	20.32112	-1.28218	-6.07583	0.241865	0.355715	0	0
60	sp|Q3UPL0	SC31A_MO	134 kDa	0.999301	19	100%	Q3UPL0	Sec31a	Protein tra	24.52068	24.10077	23.12058	24.35929	24.19547	25.25077	-0.6247	-1.79127	0.541869	24.25793	-1.27794	-6.0805	0.243775	0.358135	0	0
772	sp|Q61554	FBN1_MOU	312 kDa	0.996719	4	100%	Q61554	Fbn1	Fibrillin-1	15.93234	15.79776	15.85361	18.33627	NA	16.53124	-0.99545	-2.89501	0.904119	16.49024	-1.29896	-5.94421	0.244045	0.358143	0	0
53.1	sp|P60710	ACTB_MOU	42 kDa	0.999884	19	32%	P60710	Actb	Actin, cyto	27.26109	26.84488	27.26374	27.07091	27.37189	27.01326	0.322558	-0.28051	0.925628	27.13763	1.273038	-6.08651	0.244903	0.358627	0	0
53.2	sp|P63260	ACTG_MOU	42 kDa	0.999884	19	32%	P63260	Actg1	Actin, cyto	27.26109	26.84488	27.26374	27.07091	27.37189	27.01326	0.322558	-0.28051	0.925628	27.13763	1.273038	-6.08651	0.244903	0.358627	0	0
1035	sp|Q4VA53	PDS5B_MO	164 kDa	0.999478	2	100%	Q4VA53	Pds5b	Sister chro	21.34795	22.48577	21.53638	21.21233	21.59444	22.27492	-0.49808	-1.4396	0.443435	21.74197	-1.26248	-6.09848	0.248955	0.364167	0	0
1138	sp|P42125	ECI1_MOUS	32 kDa	0.998555	2	100%	P42125	Eci1	Enoyl-CoA	14.26245	15.81581	15.69376	14.09489	14.49537	19.20698	-1.55536	-4.50138	1.390656	15.59488	-1.25993	-6.10143	0.249817	0.364725	0	0
501	sp|Q9CZ13	QCR1_MOU	53 kDa	0.999537	5	100%	Q9CZ13	Uqcrc1	Cytochrom	22.12501	23.54609	22.59612	22.46173	22.52523	22.64883	-0.4701	-1.36011	0.419912	22.6505	-1.25918	-6.10242	0.249874	0.364725	0	0
716	sp|P62900	RL31_MOU	14 kDa	0.998717	4	100%	P62900	Rpl31	60S riboso	23.02013	22.98067	23.86283	23.02079	23.06881	22.69608	0.418077	-0.37444	1.210591	23.10822	1.255595	-6.10674	0.250791	0.36567	0	0
512	sp|A2AR02	PPIG_MOU	88 kDa	0.999448	5	100%	A2AR02	Ppig	Peptidyl-pr	23.55085	24.8093	21.79787	22.3406	23.17949	24.19385	-0.93851	-2.7215	0.844478	23.31199	-1.25615	-6.1058	0.251102	0.36573	0	0
108	sp|Q9JIX8	ACINU_MO	151 kDa	0.999387	15	100%	Q9JIX8	Acin1	Apoptotic	23.83579	23.0013	22.59459	22.90041	23.74044	22.78002	0.496363	-0.44931	1.442033	23.14209	1.252595	-6.1099	0.252315	0.366731	0	0
183	sp|P60843	IF4A1_MOU	46 kDa	0.999862	10	40%	P60843	Eif4a1	Eukaryotic	21.07199	19.89601	19.96077	20.14829	20.41824	20.07783	0.442961	-0.40009	1.28601	20.26219	1.251492	-6.11139	0.252331	0.366731	0	0
629	sp|P17182	ENOA_MOU	47 kDa	0.999435	4	50%	P17182	Eno1	Alpha-enol	20.38999	20.77463	19.48551	20.29795	20.581	20.80718	-0.47442	-1.37898	0.430142	20.38938	-1.25031	-6.11265	0.252899	0.367164	0	0
361	sp|Q6IFZ6	K2C1B_MO	61 kDa	0.999128	7	43%	Q6IFZ6	Krt77	Keratin, typ	20.03582	19.79831	20.56672	21.47475	19.98644	21.14134	-0.60848	-1.77024	0.553288	20.50056	-1.2499	-6.113	0.253238	0.367262	0	0
994	sp|Q99L88	SNTB1_MO	58 kDa	0.996484	3	67%	Q99L88	Sntb1	Beta-1-synt	19.19151	17.90519	18.17066	18.85729	19.5099	18.42548	0.561371	-0.512	1.634739	18.67667	1.248109	-6.11506	0.253854	0.367762	0	0
548	sp|Q922D8	C1TC_MOU	101 kDa	0.999011	5	100%	Q922D8	Mthfd1	C-1-tetrahy	20.59735	21.76774	21.13135	21.43041	21.11702	20.91412	-0.42218	-1.23018	0.385817	21.15966	-1.24368	-6.12045	0.254891	0.368871	0	0
802	tr|A0A075	A0A075B5Y	13 kDa	0.999506	3	33%	A0A075B5Y	Ighv1-80	Immunoglo	19.71265	19.61132	18.62336	20.98819	19.85256	19.55977	-0.65691	-1.92068	0.606873	19.72464	-1.24046	-6.12384	0.256495	0.370797	0	0
906	sp|Q69ZU6	THS7A_MO	184 kDa	0.998597	3	100%	Q69ZU6	Thsd7a	Thrombosp	20.64849	20.98868	20.97098	24.70158	20.98408	20.72049	-1.26907	-3.71729	1.179148	21.50238	-1.23705	-6.12774	0.257681	0.372114	0	0
1265	sp|Q8C092	TAF5_MOU	87 kDa	0.996358	2	100%	Q8C092	Taf5	Transcripti	22.4533	22.08904	22.9978	22.66527	22.49715	22.00323	0.396904	-0.37131	1.16512	22.45097	1.229712	-6.13644	0.259753	0.374666	0	0
118	sp|P20029	BIP_MOUSI	72 kDa	0.999596	14	86%	P20029	Hspa5	Endoplasm	24.07169	23.69779	24.68037	24.43839	24.2952	23.64415	0.422311	-0.39557	1.240194	24.13793	1.229032	-6.13721	0.260001	0.374666	0	0
449	tr|D3Z2H9	D3Z2H9_M	29 kDa	0.999056	6	67%	D3Z2H9	Tpm3-rs7	Tropomyos	21.91828	21.58947	21.8854	22.19357	23.30543	21.6336	0.564156	-0.53714	1.665453	22.08763	1.222489	-6.14433	0.262792	0.377613	0	0
767	sp|Q61292	LAMB2_MO	197 kDa	0.997106	4	100%	Q61292	Lamb2	Laminin su	19.84692	20.85124	18.91676	19.40409	20.62722	21.61181	-0.82542	-2.43689	0.78606	20.20967	-1.22236	-6.14448	0.262838	0.377613	0	0
1214	sp|Q8CG76	ARK72_MO	41 kDa	0.997748	2	100%	Q8CG76	Akr7a2	Aflatoxin B	18.67074	14.65707	19.2279	18.10806	18.62914	19.53054	1.410706	-1.34372	4.16513	18.13724	1.222239	-6.14461	0.262881	0.377613	0	0
715	sp|Q8CJ67	STAU2_MO	63 kDa	0.998727	4	100%	Q8CJ67	Stau2	Double-stra	18.31204	17.39822	18.52715	17.42763	18.25092	18.72317	0.513697	-0.49246	1.519854	18.10652	1.218406	-6.14896	0.264242	0.379166	0	0
367	sp|P61264	STX1B_MO	33 kDa	0.999014	7	100%	P61264	Stx1b	Syntaxin-1	23.69454	23.33416	22.91814	22.26714	23.53783	23.1099	0.47977	-0.46332	1.422856	23.14362	1.214038	-6.1539	0.2658	0.380308	0	0
95	sp|E9Q7E2	ARID2_MO	196 kDa	0.999408	16	100%	E9Q7E2	Arid2	AT-rich inte	22.45908	22.99555	21.34756	20.54714	22.72861	20.01082	0.993916	-0.95993	2.947763	21.68146	1.213974	-6.15397	0.265822	0.380308	0	0
212	sp|Q9WU7	PDC6I_MO	96 kDa	0.999134	10	100%	Q9WU78	Pdcd6ip	Programme	21.80837	22.37338	22.09878	22.73573	21.50208	21.65206	-0.45065	-1.33611	0.434819	22.0284	-1.21327	-6.1549	0.265879	0.380308	0	0
1220	sp|P61022	CHP1_MOU	22 kDa	0.997671	2	100%	P61022	Chp1	Calcineurin	16.26696	17.42891	16.9435	16.1818	16.31512	17.54921	-0.54478	-1.61759	0.528036	16.78092	-1.21184	-6.15638	0.266586	0.380917	0	0
691	sp|P51150	RAB7A_MO	23 kDa	0.998994	4	100%	P51150	Rab7a	Ras-related	21.92977	22.84273	23.07417	21.85513	23.66143	22.09604	0.623826	-0.60879	1.856442	22.57654	1.207775	-6.16097	0.268047	0.382601	0	0
801	tr|E9Q6E5	E9Q6E5_M	57 kDa	0.99951	3	100%	E9Q6E5	Srsf11	Serine/argi	20.82789	20.43009	21.63236	20.80723	20.65197	20.61885	0.418684	-0.41066	1.248028	20.82807	1.201639	-6.16821	0.269788	0.384682	0	0
396	sp|P05064	ALDOA_MO	39 kDa	0.999727	6	100%	P05064	Aldoa	Fructose-bi	22.58627	23.47733	23.39034	23.49036	22.6982	22.93938	-0.41075	-1.22639	0.40489	23.09698	-1.19867	-6.17153	0.270866	0.385813	0	0
999	sp|Q3U0S6	RAIN_MOU	104 kDa	0.99588	3	100%	Q3U0S6	Rasip1	Ras-interac	19.04704	19.79216	18.94951	19.88899	19.72588	19.26755	-0.40876	-1.22583	0.408315	19.44519	-1.19077	-6.18038	0.273757	0.389522	0	0
1118	sp|Q9CZX7	PP4P2_MO	28 kDa	0.998761	2	100%	Q9CZX7	Pip4p2	Type 2 pho	18.33188	19.50028	18.0082	18.10931	18.87474	19.21301	-0.53593	-1.61676	0.544901	18.6729	-1.18332	-6.18834	0.276978	0.393691	0	0
30	sp|Q6DIC0	SMCA2_MO	180 kDa	0.999766	25	68%	Q6DIC0	Smarca2	Probable gl	24.44188	24.3204	23.4863	23.00031	24.71935	23.62814	0.566228	-0.57753	1.709982	23.93273	1.181435	-6.19043	0.277675	0.39427	0	0
232	sp|O08599	STXB1_MO	68 kDa	0.999644	9	100%	O08599	Stxbp1	Syntaxin-bi	23.33014	24.06757	23.7112	23.70792	23.53412	23.76879	-0.32294	-0.97536	0.329477	23.68662	-1.17814	-6.19444	0.278422	0.394916	0	0
1124	sp|Q8BRT1	CLAP2_MO	141 kDa	0.998677	2	100%	Q8BRT1	Clasp2	CLIP-associ	20.42563	20.1827	20.86269	20.08948	20.35398	20.37979	0.330107	-0.3428	1.003011	20.38238	1.167624	-6.20607	0.282364	0.40009	0	0
753	sp|P97789	XRN1_MOU	194 kDa	0.997634	4	100%	P97789	Xrn1	5'-3' exorib	20.61666	19.6441	20.93442	20.87949	20.85339	20.54193	0.446318	-0.46527	1.357907	20.57833	1.167248	-6.20626	0.282802	0.400292	0	0
211.1	sp|Q91VC3	IF4A3_MOU	47 kDa	0.99918	10	80%	Q91VC3	Eif4a3	Eukaryotic	23.05602	22.71626	22.55292	24.13776	23.62744	24.12589	-0.58118	-1.7747	0.612336	23.36938	-1.16207	-6.21181	0.284937	0.402054	0	0
211.2	tr|A0A0N4	A0A0N4SVI	47 kDa	0.99918	10	80%	A0A0N4SVI	Gm5580	Predicted p	23.05602	22.71626	22.55292	24.13776	23.62744	24.12589	-0.58118	-1.7747	0.612336	23.36938	-1.16207	-6.21181	0.284937	0.402054	0	0
211.3	tr|E9PV04	E9PV04_M	47 kDa	0.99918	10	80%	E9PV04	Gm8994	Predicted g	23.05602	22.71626	22.55292	24.13776	23.62744	24.12589	-0.58118	-1.7747	0.612336	23.36938	-1.16207	-6.21181	0.284937	0.402054	0	0
1159	sp|O88685	PRS6A_MO	50 kDa	0.998334	2	100%	O88685	Psmc3	26S protea	18.05916	18.96047	18.32641	18.41132	17.6215	17.92422	-0.42965	-1.31643	0.457131	18.21718	-1.15502	-6.21969	0.287433	0.405155	0	0
576	sp|Q3UIL6	PKHA7_MO	127 kDa	0.99801	5	100%	Q3UIL6	Plekha7	Pleckstrin h	20.29183	19.15625	19.08372	20.08616	19.99944	18.16854	0.654681	-0.699	2.008361	19.46432	1.154157	-6.22049	0.287951	0.405464	0	0
221	sp|Q9ESX5	DKC1_MOU	57 kDa	0.9999	9	100%	Q9ESX5	Dkc1	H/ACA ribo	22.24927	22.80967	22.01281	21.40163	22.69208	20.97133	0.59051	-0.64337	1.824388	22.0228	1.142106	-6.23362	0.292591	0.41157	0	0
996	sp|Q61699	HS105_MO	96 kDa	0.996089	3	67%	Q61699	Hsph1	Heat shock	22.72279	22.25554	23.556	22.73277	22.00289	21.60967	0.561236	-0.61544	1.737916	22.47994	1.138249	-6.23781	0.294089	0.413248	0	0
93	sp|Q8BH59	CMC1_MOU	75 kDa	0.999516	16	88%	Q8BH59	Slc25a12	Calcium-bi	24.71949	24.52611	24.85689	23.9849	24.46802	24.52528	0.336037	-0.36807	1.040146	24.51345	1.135921	-6.24071	0.294532	0.413442	0	0
696	sp|P18760	COF1_MOU	19 kDa	0.998935	4	75%	P18760	Cfl1	Cofilin-1	22.48757	21.98246	21.6139	22.46261	21.58576	22.44953	-0.40246	-1.25169	0.446778	22.09697	-1.1295	-6.24744	0.297287	0.416878	0	0
1067	sp|Q9QYJ0	DNJA2_MO	46 kDa	0.999232	2	100%	Q9QYJ0	Dnaja2	DnaJ homo	19.01908	18.39065	17.81519	20.52241	19.60757	19.81672	-0.76264	-2.38218	0.856895	19.19527	-1.12378	-6.25342	0.299765	0.419307	0	0
1262	sp|Q9QZ06	TOLIP_MO	30 kDa	0.996417	2	100%	Q9QZ06	Tollip	Toll-interac	18.56015	17.36388	16.65478	17.4022	15.91283	18.96257	-0.86697	-2.71008	0.976144	17.47607	-1.12254	-6.25475	0.300256	0.419307	0	0
1263	sp|Q8C050	KS6A5_MO	97 kDa	0.996414	2	100%	Q8C050	Rps6ka5	Ribosomal	18.56015	17.36388	16.65478	17.4022	15.91283	18.96257	-0.86697	-2.71008	0.976144	17.47607	-1.12254	-6.25475	0.300256	0.419307	0	0
1264	sp|Q9JME5	AP3B2_MO	119 kDa	0.99637	2	100%	Q9JME5	Ap3b2	AP-3 comp	18.56015	17.36388	16.65478	17.4022	15.91283	18.96257	-0.86697	-2.71008	0.976144	17.47607	-1.12254	-6.25475	0.300256	0.419307	0	0
425	sp|Q60673	PTPRN_MO	106 kDa	0.999409	6	83%	Q60673	Ptprn	Receptor-ty	21.69706	22.63807	22.76399	22.60891	21.73337	22.21977	-0.42411	-1.3273	0.479078	22.27686	-1.11943	-6.25824	0.301308	0.420344	0	0
40	sp|P17156	HSP72_MO	70 kDa	0.999892	22	55%	P17156	Hspa2	Heat shock	25.21283	24.77557	24.97384	24.75298	25.17259	24.9733	0.285805	-0.3294	0.90101	24.97685	1.105734	-6.27309	0.306517	0.427171	0	0
975	sp|O08664	BCL7C_MO	23 kDa	0.997539	3	67%	O08664	Bcl7c	B-cell CLL/l	18.52368	19.65756	18.48339	20.09007	18.47839	17.74715	-0.66977	-2.11973	0.780188	18.83004	-1.10236	-6.27628	0.308336	0.429264	0	0
411	sp|Q9Z2X1	HNRPF_MO	46 kDa	0.999584	6	83%	Q9Z2X1	Hnrnpf	Heterogene	21.42801	21.04381	20.36141	20.96755	21.14544	22.90569	-0.66073	-2.09308	0.771612	21.30865	-1.10085	-6.27787	0.308945	0.42953	0	0
91	sp|P56480	ATPB_MOU	56 kDa	0.999697	16	100%	P56480	Atp5f1b	ATP syntha	25.16068	25.45215	25.34254	25.33352	25.15319	25.75801	-0.29575	-0.93615	0.344647	25.36668	-1.09921	-6.28001	0.30916	0.42953	0	0
856	sp|P12367	KAP2_MOU	45 kDa	0.999076	3	67%	P12367	Prkar2a	cAMP-depe	20.75131	21.2365	21.2305	21.37016	21.18599	21.46858	-0.30248	-0.96358	0.358626	21.20717	-1.08899	-6.29079	0.313336	0.434564	0	0
625	sp|P01897	HA1L_MOU	41 kDa	0.999491	4	100%	P01897	H2-L	H-2 class I h	21.84149	21.48162	22.86674	22.06264	21.88092	21.80673	0.41272	-0.49091	1.316352	21.99002	1.088827	-6.29072	0.313672	0.434564	0	0
606	sp|Q99MR	MCCA_MOU	79 kDa	0.999677	4	100%	Q99MR8	Mccc1	Methylcrot	20.12466	20.95707	20.72556	20.63492	20.0551	20.36848	-0.35171	-1.12112	0.417703	20.47763	-1.08799	-6.29183	0.313745	0.434564	0	0
595	sp|P84099	RL19_MOU	23 kDa	0.99974	4	100%	P84099	Rpl19	60S riboso	24.42087	24.4852	26.29186	24.81166	24.85375	24.6224	0.549073	-0.65824	1.756381	24.91429	1.085331	-6.29423	0.315288	0.436255	0	0
302	sp|P70398	USP9X_MO	291 kDa	0.997909	8	100%	P70398	Usp9x	Probable u	21.59574	21.49756	21.545	23.19385	21.72931	21.73635	-0.51924	-1.66188	0.623403	21.88297	-1.08445	-6.29516	0.315653	0.436316	0	0
935	sp|Q8K212	PACS1_MO	105 kDa	0.998128	3	67%	Q8K212	Pacs1	Phosphofu	17.17446	16.60528	17.72118	18.03294	17.3244	20.77297	-1.06371	-3.41231	1.284883	17.93854	-1.08085	-6.29892	0.317137	0.436854	0	0
1079	sp|Q8BVE3	VATH_MOU	56 kDa	0.999136	2	100%	Q8BVE3	Atp6v1h	V-type prot	19.61019	20.00108	20.18677	20.03633	20.07403	21.13036	-0.43226	-1.38761	0.523094	20.17313	-1.07977	-6.30005	0.317587	0.436854	0	0
795	sp|Q9JLM8	DCLK1_MO	84 kDa	0.999593	3	100%	Q9JLM8	Dclk1	Serine/thre	21.98741	21.39565	21.15474	21.41427	22.03333	21.29436	0.357063	-0.43091	1.145036	21.54663	1.078536	-6.30174	0.317654	0.436854	0	0
796	sp|P70296	PEBP1_MO	21 kDa	0.99957	3	100%	P70296	Pebp1	Phosphatid	21.98741	21.39565	21.15474	21.41427	22.03333	21.29436	0.357063	-0.43091	1.145036	21.54663	1.078536	-6.30174	0.317654	0.436854	0	0
797	sp|P35282	RAB21_MO	24 kDa	0.999555	3	67%	P35282	Rab21	Ras-related	21.98741	21.39565	21.15474	21.41427	22.03333	21.29436	0.357063	-0.43091	1.145036	21.54663	1.078536	-6.30174	0.317654	0.436854	0	0
22	sp|Q99PL5	RRBP1_MO	173 kDa	0.999797	27	100%	Q99PL5	Rrbp1	Ribosome-b	25.41022	25.37928	25.6512	24.91899	25.72088	25.53594	0.316031	-0.38255	1.014609	25.43609	1.076752	-6.3036	0.318396	0.437431	0	0
29	sp|Q6A0A9	F120A_MO	122 kDa	0.999791	25	92%	Q6A0A9	FAM120A	Constitutiv	24.79018	24.02983	24.82944	23.89092	24.68235	25.12409	0.419041	-0.51009	1.348176	24.5578	1.076289	-6.30368	0.319032	0.437862	0	0
706	sp|Q76MZ3	2AAA_MOU	65 kDa	0.998838	4	100%	Q76MZ3	Ppp2r1a	Serine/thre	21.04498	20.67588	21.5613	20.78137	21.19417	21.33114	0.337356	-0.41124	1.085948	21.09814	1.072616	-6.30791	0.320121	0.438912	0	0
1108	sp|P54116	STOM_MOU	31 kDa	0.998855	2	100%	P54116	Stom	Erythrocyte	17.72699	17.93445	18.19323	NA	16.65377	15.12948	0.992699	-1.2862	3.271602	17.12758	1.079749	-6.18572	0.323827	0.443545	0	0
1155	sp|Q9EPK6	SIL1_MOUS	52 kDa	0.998388	2	100%	Q9EPK6	Sil1	Nucleotide	18.82831	20.58365	19.21105	19.52668	20.09733	19.51783	-0.49715	-1.61323	0.618926	19.62748	-1.06303	-6.31744	0.324586	0.444136	0	0
878	sp|P05202	AATM_MOU	47 kDa	0.998944	3	100%	P05202	Got2	Aspartate a	19.09702	19.5882	19.82708	20.85778	19.37733	19.33916	-0.49457	-1.6105	0.621365	19.68109	-1.05764	-6.323	0.326866	0.446316	0	0
1083	sp|Q9D666	SUN1_MOU	102 kDa	0.999095	2	100%	Q9D666	Sun1	SUN domai	18.52893	17.95948	17.8518	18.07803	18.55362	17.93081	0.322011	-0.40416	1.048177	18.15044	1.055443	-6.32567	0.327364	0.446316	0	0
1084	sp|Q8BH58	TIPRL_MOU	31 kDa	0.999091	2	100%	Q8BH58	Tiprl	TIP41-like p	18.52893	17.95948	17.8518	18.07803	18.55362	17.93081	0.322011	-0.40416	1.048177	18.15044	1.055443	-6.32567	0.327364	0.446316	0	0
97	sp|P68368	TBA4A_MO	50 kDa	0.999782	15	33%	P68368	Tuba4a	Tubulin alp	24.50154	24.53722	24.01647	24.29535	23.90417	24.50605	-0.30548	-0.99498	0.384026	24.29347	-1.0545	-6.32665	0.327768	0.446316	0	0
1031	sp|P62315	SMD1_MOU	13 kDa	0.999534	2	100%	P62315	Snrpd1	Small nucle	22.91578	20.40756	23.37379	23.58675	22.82967	22.65532	0.823206	-1.03823	2.684642	22.62814	1.055385	-6.32533	0.327825	0.446316	0	0
46	sp|P39054	DYN2_MOU	98 kDa	0.999773	20	45%	P39054	Dnm2	Dynamin-2	24.10148	23.12313	22.72644	25.41301	24.45549	25.04125	-0.76466	-2.49598	0.96666	24.14347	-1.054	-6.32675	0.328413	0.446578	0	0
76	sp|P68369	TBA1A_MO	50 kDa	0.999608	17	41%	P68369	Tuba1a	Tubulin alp	26.08717	25.4928	25.21534	25.23483	25.58089	25.13188	0.341299	-0.43062	1.113215	25.45715	1.052365	-6.32884	0.328676	0.446578	0	0
823	sp|P62996	TRA2B_MO	34 kDa	0.999382	3	100%	P62996	Tra2b	Transforme	22.90713	22.94679	24.26385	23.21417	22.91069	22.47936	0.480447	-0.61046	1.571358	23.12033	1.051009	-6.32982	0.32969	0.447507	0	0
498	sp|P28652	KCC2B_MO	60 kDa	0.999561	5	60%	P28652	Camk2b	Calcium/ca	21.79719	21.57455	21.06306	20.87704	21.43291	20.71653	0.375013	-0.4776	1.227627	21.24355	1.04831	-6.33278	0.330635	0.448342	0	0
314	sp|P49312	ROA1_MOU	34 kDa	0.99966	7	71%	P49312	Hnrnpa1	Heterogene	22.99258	22.15958	21.18278	22.92846	21.91006	22.57914	-0.52725	-1.73049	0.675984	22.2921	-1.04573	-6.33522	0.331952	0.449479	0	0
838	sp|Q9D7B6	ACAD8_MO	45 kDa	0.999265	3	100%	Q9D7B6	Acad8	Isobutyryl-	21.34062	19.95169	19.84672	21.7007	20.51211	22.10983	-0.6876	-2.2574	0.882206	20.91028	-1.0453	-6.33566	0.332137	0.449479	0	0
1222	sp|Q9ES74	NEK7_MOU	35 kDa	0.997627	2	100%	Q9ES74	Nek7	Serine/thre	17.30336	17.51005	16.46635	17.802	17.17102	16.83538	-0.40223	-1.32263	0.518171	17.18136	-1.04261	-6.33845	0.333246	0.450371	0	0
978	sp|Q8BJY1	PSMD5_MO	56 kDa	0.997369	3	100%	Q8BJY1	Psmd5	26S protea	17.99708	18.99322	17.13591	16.03473	17.32847	13.41864	1.338288	-1.72607	4.402648	16.81801	1.042223	-6.3388	0.33346	0.450371	0	0
697	sp|Q5U4D9	THOC6_MO	37 kDa	0.998918	4	100%	Q5U4D9	Thoc6	THO compl	17.57242	18.87889	17.98383	17.10629	18.95096	22.66261	-1.38019	-4.54878	1.788396	18.85917	-1.0395	-6.34157	0.334636	0.451508	0	0
371	sp|Q8BMA	SRP68_MO	71 kDa	0.998976	7	100%	Q8BMA6	Srp68	Signal reco	22.36527	22.85536	22.53385	22.82614	22.44061	22.48292	-0.2749	-0.90703	0.35724	22.58403	-1.03505	-6.34651	0.336137	0.453084	0	0
307	sp|P06151	LDHA_MOU	36 kDa	0.999832	7	86%	P06151	Ldha	L-lactate de	21.97781	22.52739	23.03656	23.13185	21.88315	22.49528	-0.419	-1.39376	0.555759	22.50867	-1.02581	-6.35543	0.340596	0.458492	0	0
649	sp|Q9CQD1	RAB5A_MO	24 kDa	0.999339	4	50%	Q9CQD1	Rab5a	Ras-related	20.33762	20.37961	20.13024	20.02577	20.43603	19.51389	0.328206	-0.4347	1.091108	20.13719	1.02395	-6.35772	0.340988	0.458492	0	0
640	sp|Q9DCD0	6PGD_MOU	53 kDa	0.9994	4	100%	Q9DCD0	Pgd	6-phospho	23.19676	23.46002	21.70685	21.87411	22.37085	23.85979	-0.63982	-2.13033	0.850696	22.74473	-1.0244	-6.35685	0.341213	0.458492	0	0
727	sp|B2RSH2	GNAI1_MO	40 kDa	0.998489	4	25%	B2RSH2	Gnai1	Guanine nu	19.58567	20.01871	20.06553	20.19325	19.85572	20.12743	-0.27749	-0.92323	0.368256	19.97438	-1.02278	-6.35889	0.341502	0.458492	0	0
495	sp|Q922R8	PDIA6_MO	48 kDa	0.999609	5	100%	Q922R8	Pdia6	Protein dis	23.45302	24.10854	23.73881	23.15559	23.21565	24.3297	-0.39545	-1.3219	0.531007	23.66688	-1.01848	-6.36282	0.3438	0.46112	0	0
644	sp|P50396	GDIA_MOU	51 kDa	0.999371	4	75%	P50396	Gdi1	Rab GDP di	21.27701	22.72952	22.70496	21.70335	21.18493	22.2775	-0.51449	-1.72397	0.694981	21.97954	-1.01516	-6.36612	0.345291	0.462663	0	0
630	sp|Q9QXV0	PCSK1_MO	27 kDa	0.999433	4	100%	Q9QXV0	Pcsk1n	ProSAAS	20.71248	20.08099	20.83897	21.9361	22.41646	19.77664	0.724726	-0.98124	2.430693	20.96027	1.013804	-6.36747	0.345891	0.463009	0	0
936	sp|Q9D5V5	CUL5_MOU	91 kDa	0.998121	3	100%	Q9D5V5	Cul5	Cullin-5	19.62982	20.14108	20.61344	20.37752	19.29841	20.21986	-0.39893	-1.34032	0.542464	20.04669	-1.01129	-6.36998	0.347008	0.464046	0	0
619	sp|P62880	GBB2_MOU	37 kDa	0.99954	4	50%	P62880	Gnb2	Guanine nu	18.66145	19.46895	20.7353	18.50855	19.12446	18.76646	0.592415	-0.80868	1.993514	19.21086	1.009039	-6.37223	0.348011	0.464929	0	0
1241.1	sp|P0C0S6	H2AZ_MOU	14 kDa	0.997171	2	50%	P0C0S6	H2afz	Histone H2	20.56766	16.89908	20.61792	21.18222	20.36502	20.32369	1.048535	-1.43777	3.534836	19.9926	1.006422	-6.37483	0.34918	0.466031	0	0
432	sp|Q8BJ05	ZC3HE_MO	82 kDa	0.999309	6	100%	Q8BJ05	Zc3h14	Zinc finger	20.97267	22.06546	22.57092	25.0766	22.87565	22.0813	-0.93471	-3.15566	1.286244	22.6071	-1.00435	-6.37688	0.350105	0.466806	0	0
1065	sp|P51174	ACADL_MO	48 kDa	0.99924	2	100%	P51174	Acadl	Long-chain	21.87831	21.67857	22.95876	22.51112	21.83541	20.93909	0.51457	-0.71022	1.739363	21.96688	1.002612	-6.37861	0.350886	0.466933	0	0
233	sp|P48036	ANXA5_MO	36 kDa	0.999564	9	100%	P48036	Anxa5	Annexin A5	22.32253	22.97193	22.33126	24.03029	23.66282	22.85298	-0.51286	-1.73359	0.70787	23.02863	-1.00261	-6.37861	0.350889	0.466933	0	0
520	sp|Q9DB20	ATPO_MOU	23 kDa	0.99933	5	100%	Q9DB20	Atp5po	ATP syntha	22.19405	22.36927	22.79422	22.20827	22.96171	22.44518	0.309088	-0.42688	1.045053	22.49545	0.999601	-6.382	0.351825	0.46772	0	0
916.14	sp|Q8R1M	H2AJ_MOU	14 kDa	0.998436	3	33%	Q8R1M2	H2afj	Histone H2	20.55017	20.91647	20.73682	20.46705	19.89855	20.78899	-0.32899	-1.11598	0.457994	20.55968	-0.995	-6.38655	0.353905	0.469451	0	0
166	sp|Q7TMQ	WDR91_M	83 kDa	0.999462	11	100%	Q7TMQ7	Wdr91	WD repeat-	22.68742	21.59898	20.86125	21.73298	23.6267	21.82641	0.672337	-0.93895	2.283628	22.05562	0.995781	-6.38536	0.353962	0.469451	0	0
1018	sp|P35454	NEU1_MOU	13 kDa	0.999755	2	50%	P35454	Oxt	Oxytocin-n	20.47943	20.51376	20.70255	20.34504	20.75425	20.30329	0.258046	-0.35958	0.875675	20.51639	0.99442	-6.38711	0.354165	0.469451	0	0
440	sp|P62192	PRS4_MOU	49 kDa	0.999207	6	100%	P62192	Psmc1	26S protea	21.47137	21.79434	21.7285	21.83467	21.39242	21.7357	-0.25748	-0.87476	0.359804	21.6595	-0.99279	-6.38872	0.354906	0.469589	0	0
43	sp|Q501J6	DDX17_MO	72 kDa	0.999633	22	64%	Q501J6	Ddx17	Probable A	23.96127	23.33629	23.52084	23.37517	23.65664	23.61145	0.271949	-0.38015	0.924048	23.57694	0.9926	-6.3889	0.354991	0.469589	0	0
151	sp|Q8K400	STXB5_MO	128 kDa	0.99924	12	92%	Q8K400	Stxbp5	Syntaxin-bi	24.20699	23.99182	23.40208	22.24267	24.0704	23.85512	0.529957	-0.74392	1.803832	23.62818	0.992807	-6.38829	0.355308	0.469589	0	0
376	sp|P46978	STT3A_MO	81 kDa	0.998661	7	86%	P46978	Stt3a	Dolichyl-di	23.98039	22.85556	23.86563	23.63066	23.53746	23.84965	0.349206	-0.49171	1.190125	23.61989	0.989083	-6.39225	0.356697	0.470965	0	0
738	sp|P01803	HVM34_M	13 kDa	0.998328	4	25%	P01803	NA	Ig heavy ch	21.00019	20.56115	21.76202	20.72628	20.83225	21.22736	0.359887	-0.50794	1.22771	21.01821	0.988618	-6.39256	0.357048	0.47097	0	0
1112	sp|Q6PDM	SRSF1_MO	28 kDa	0.998826	2	100%	Q6PDM2	Srsf1	Serine/argi	18.06532	17.94049	18.01049	17.1134	18.4661	18.34514	0.380959	-0.54432	1.306237	17.99016	0.982364	-6.39855	0.360036	0.474449	0	0
899.2	sp|P63163	RSMN_MOU	25 kDa	0.998705	3	100%	P63163	Snrpn	Small nucle	19.21848	16.87568	19.38276	19.58512	19.55483	19.54426	0.717003	-1.045	2.479002	19.02686	0.971104	-6.40946	0.36525	0.480854	0	0
284	sp|Q8BFZ9	ERLN2_MO	38 kDa	0.999317	8	100%	Q8BFZ9	Erlin2	Erlin-2	23.85399	23.55766	23.98913	23.68708	23.47934	23.22943	0.282766	-0.41261	0.978147	23.63277	0.967844	-6.41302	0.366361	0.481848	0	0
1032	sp|Q3UNZ8	QORL2_MO	38 kDa	0.999523	2	100%	Q3UNZ8	Cryzl2	Quinone ox	17.66629	16.0421	16.95147	17.82363	17.80533	17.07797	0.49313	-0.72547	1.711726	17.2278	0.965723	-6.41465	0.367749	0.483205	0	0
172	sp|O35286	DHX15_MO	91 kDa	0.999312	11	100%	O35286	Dhx15	Pre-mRNA-	23.27429	22.46308	22.84967	22.13879	23.18034	23.51594	0.395499	-0.59624	1.387244	22.90368	0.951693	-6.4281	0.374324	0.490904	0	0
1145	tr|A0A075	A0A075B5	11 kDa	0.998505	2	100%	A0A075B5	Ighv9-4	Immunoglo	20.1762	18.50193	18.55013	19.61981	19.24259	18.30321	0.514654	-0.7759	1.805211	19.06564	0.951675	-6.42811	0.374332	0.490904	0	0
143	sp|O88532	ZFR_MOUS	117 kDa	0.999727	12	100%	O88532	Zfr	Zinc finger	22.00516	21.81905	21.97103	22.60968	22.07328	22.50433	-0.29454	-1.03761	0.44854	22.16375	-0.94342	-6.43636	0.377851	0.495039	0	0
605	sp|Q8BWT	THIM_MOU	42 kDa	0.999678	4	100%	Q8BWT1	Acaa2	3-ketoacyl-	22.02005	22.32851	22.10197	21.93375	21.3777	22.1269	-0.29648	-1.04647	0.453512	21.98148	-0.94089	-6.43875	0.379055	0.495846	0	0
1233	sp|Q9CZJ2	HS12B_MO	76 kDa	0.997383	2	100%	Q9CZJ2	Hspa12b	Heat shock	19.82611	19.5062	19.87865	19.79408	19.70304	19.34939	0.252714	-0.38727	0.892701	19.67625	0.93985	-6.43974	0.379553	0.495846	0	0
718	sp|P61089	UBE2N_MO	17 kDa	0.998699	4	100%	P61089	Ube2n	Ubiquitin-c	21.65186	22.14184	22.4304	22.11026	21.35633	22.16883	-0.32745	-1.15675	0.501849	21.97659	-0.93984	-6.43974	0.379564	0.495846	0	0
504	sp|Q9D8N0	EF1G_MOU	50 kDa	0.99953	5	100%	Q9D8N0	Eef1g	Elongation	22.32356	22.52574	23.27353	22.48682	22.06277	24.34953	-0.56741	-2.01111	0.876291	22.83699	-0.93793	-6.44114	0.38086	0.49706	0	0
979	tr|Q80ZX0	Q80ZX0_M	136 kDa	0.997282	3	100%	Q80ZX0	Sec24b	Sec24-relat	13.61362	17.84742	18.20925	17.65841	18.26751	18.00711	-1.14085	-4.04913	1.767419	17.26722	-0.93615	-6.44282	0.381712	0.497694	0	0
287	sp|Q03517	SCG2_MOU	71 kDa	0.999259	8	100%	Q03517	Scg2	Secretogra	23.56611	23.04486	23.21411	24.68829	23.72483	24.05759	-0.42856	-1.52229	0.665162	23.71597	-0.9351	-6.44381	0.382216	0.497872	0	0
588	sp|Q99JP0	M4K3_MOU	101 kDa	0.996695	5	100%	Q99JP0	Map4k3	Mitogen-ac	20.31399	21.87734	19.24963	19.43373	20.36377	20.46836	-0.61734	-2.19667	0.961978	20.28447	-0.93284	-6.44593	0.383299	0.498804	0	0
1166	sp|Q99J99	THTM_MOU	33 kDa	0.998285	2	100%	Q99J99	Mpst	3-mercapto	20.49583	19.26034	20.56021	19.61356	20.28542	21.08983	0.459249	-0.73687	1.655373	20.21753	0.916269	-6.46137	0.391323	0.508757	0	0
569	sp|Q8JZX4	SPF45_MO	45 kDa	0.998333	5	100%	Q8JZX4	Rbm17	Splicing fac	20.76884	21.00199	19.31749	20.67929	20.19204	19.90931	-0.43741	-1.57854	0.703726	20.31149	-0.91475	-6.46278	0.392066	0.509235	0	0
397	sp|P62983	RS27A_MO	18 kDa	0.999709	6	100%	P62983	Rps27a	Ubiquitin-4	25.49872	25.82533	25.87552	25.85119	25.4093	25.84478	-0.24592	-0.888	0.396163	25.71747	-0.9116	-6.46609	0.393229	0.510257	0	0
985	sp|Q99JI6	RAP1B_MO	21 kDa	0.997076	3	33%	Q99JI6	Rap1b	Ras-related	18.51436	22.86944	17.55084	18.56424	18.54881	17.00714	-1.27561	-4.61591	2.064704	18.84247	-0.91134	-6.46592	0.393734	0.510424	0	0
357	sp|Q99ME2	WDR6_MO	122 kDa	0.999167	7	100%	Q99ME2	Wdr6	WD repeat-	23.62871	22.82514	23.9283	25.85653	23.42609	24.2862	-0.66159	-2.39871	1.075531	23.99183	-0.90889	-6.46818	0.394938	0.511495	0	0
494	sp|Q64332	SYN2_MOU	63 kDa	0.99965	5	80%	Q64332	Syn2	Synapsin-2	21.17829	21.07763	21.27296	20.62693	21.7488	21.5495	0.315333	-0.51235	1.143012	21.24235	0.906837	-6.47046	0.395577	0.511834	0	0
667	sp|P01867	IGG2B_MO	44 kDa	0.999146	4	100%	P01867	Igh-3	Ig gamma-2	27.36625	27.68074	27.58628	28.37094	28.37658	28.31208	-0.34488	-1.25256	0.562794	27.94881	-0.90583	-6.47115	0.396297	0.512277	0	0
1043	sp|Q791V5	MTCH2_MO	33 kDa	0.999396	2	100%	Q791V5	Mtch2	Mitochond	19.64914	21.03393	21.41856	21.57474	20.86456	20.70817	-0.46153	-1.6803	0.757246	20.87485	-0.9037	-6.47294	0.397491	0.512476	0	0
551	tr|Q8C5R8	Q8C5R8_M	35 kDa	0.998927	5	20%	Q8C5R8	Prps1l1	Phosphorib	15.3737	15.25413	16.09713	16.77661	14.93804	NA	-0.54575	-2.02753	0.936037	15.68792	-0.91293	-6.3496	0.398263	0.512476	0	0
552.1	sp|Q9D7G0	PRPS1_MO	35 kDa	0.998927	5	20%	Q9D7G0	Prps1	Ribose-pho	15.3737	15.25413	16.09713	16.77661	14.93804	NA	-0.54575	-2.02753	0.936037	15.68792	-0.91293	-6.3496	0.398263	0.512476	0	0

552.2	tr|G3UXL2	G3UXL2_M	35 kDa	0.998927	5	20%	G3UXL2	Prps1l3	Phosphorib	15.3737	15.25413	16.09713	16.77661	14.93804	NA	-0.54575	-2.02753	0.936037	15.68792	-0.91293	-6.3496	0.398263	0.512476	0	0
234	sp|P68040	RACK1_MC	35 kDa	0.999549	9	100%	P68040	Rack1	Receptor o	23.12812	23.29674	23.12434	22.45088	23.56158	23.15558	0.303612	-0.49822	1.105448	23.11954	0.901229	-6.4756	0.39834	0.512476	0	0
210	sp|Q922F4	TBB6_MOU	50 kDa	0.999274	10	10%	Q922F4	Tubb6	Tubulin be	18.24358	17.75739	18.99348	18.88422	19.11044	18.58991	0.371989	-0.61673	1.360706	18.5965	0.897861	-6.47827	0.400383	0.514615	0	0
156	sp|P46735	MYO1B_MC	129 kDa	0.997764	12	100%	P46735	Myo1b	Unconvent	22.29839	22.89147	22.598	22.30505	22.39406	22.88695	-0.26434	-0.96908	0.440398	22.56232	-0.89276	-6.48331	0.40255	0.516911	0	0
327	sp|P38647	GRP75_MC	73 kDa	0.999554	7	100%	P38647	Hspa9	Stress-70 p	22.60589	23.28218	23.21156	22.99888	25.86725	23.22692	0.72577	-1.218	2.669537	23.53221	0.891056	-6.48445	0.403771	0.517989	0	0
1004	sp|Q9EP69	SAC1_MOU	67 kDa	0.995413	3	100%	Q9EP69	Sacm1l	Phosphatid	19.02959	18.83593	20.23165	18.8804	18.97748	19.39891	0.3745	-0.63609	1.385088	19.22566	0.884357	-6.4905	0.407128	0.52085	0	0
1005	sp|Q2YDW	MSTO1_MC	61 kDa	0.995317	3	100%	Q2YDW2	Msto1	Protein mi	19.02959	18.83593	20.23165	18.8804	18.97748	19.39891	0.3745	-0.63609	1.385088	19.22566	0.884357	-6.4905	0.407128	0.52085	0	0
724	sp|Q8R001	MARE2_MC	37 kDa	0.998599	4	100%	Q8R001	Mapre2	Microtubu	20.94129	20.07386	20.39233	22.55165	20.97621	18.72213	0.987397	-1.67726	3.65205	20.27624	0.884305	-6.49055	0.407154	0.52085	0	0
1229	sp|Q91WQ	SYYC_MOU	59 kDa	0.997532	2	100%	Q91WQ3	Yars	Tyrosine--t	18.69858	18.45269	19.22397	17.54956	18.75455	19.37554	0.433104	-0.74118	1.607384	18.67582	0.880179	-6.49426	0.409232	0.523014	0	0
563	sp|Q3UGY8	BIG3_MOU	240 kDa	0.998646	5	100%	Q3UGY8	Arfgef3	Brefeldin A	20.78891	22.74401	21.24238	20.29615	21.76054	22.57253	-0.60696	-2.25753	1.043624	21.56742	-0.87755	-6.49661	0.41056	0.524218	0	0
450	sp|Q99LC3	NDUAA_M	41 kDa	0.99905	6	100%	Q99LC3	Ndufa10	NADH dehy	22.84203	23.20579	22.62237	23.09226	23.16437	23.05759	-0.24229	-0.90058	0.416001	22.9974	-0.87603	-6.49837	0.410967	0.524244	0	0
138	sp|Q91W3	NCOA5_MC	65 kDa	0.999193	13	100%	Q91W39	Ncoa5	Nuclear rec	23.69294	22.71697	23.73591	24.60557	23.25543	24.93077	-0.52301	-1.95117	0.905146	23.82293	-0.87395	-6.49983	0.412385	0.525558	0	0
977	sp|Q9QXX4	CMC2_MOI	74 kDa	0.997372	3	33%	Q9QXX4	Slc25a13	Calcium-bi	14.71319	16.20681	17.39441	17.07012	16.14617	16.74452	-0.58923	-2.20038	1.021922	16.3792	-0.87277	-6.50088	0.412985	0.525825	0	0
421	sp|Q8VEM8	MPCP_MOI	40 kDa	0.99946	6	100%	Q8VEM8	Slc25a3	Phosphate	23.74263	23.55037	25.03682	24.06591	23.70909	23.74246	0.376601	-0.65598	1.409185	23.97455	0.870376	-6.50301	0.414199	0.526879	0	0
537	sp|Q9ERK4	XPO2_MOL	110 kDa	0.999209	5	100%	Q9ERK4	Cse1l	Exportin-2	20.79164	20.64448	21.54968	20.55356	20.57623	20.83262	0.29563	-0.51532	1.106583	20.8247	0.86771	-6.50576	0.415205	0.527159	0	0
580	sp|Q9QUP5	HPLN1_MC	40 kDa	0.997599	5	100%	Q9QUP5	Hapln1	Hyaluronar	22.02736	19.64185	18.98758	19.97603	19.70585	19.08239	0.673508	-1.17919	2.52621	19.90351	0.867536	-6.50553	0.415646	0.527159	0	0
846	sp|Q9QXL2	KI21A_MOI	187 kDa	0.999184	3	100%	Q9QXL2	Kif21a	Kinesin-like	21.47721	21.9467	21.17533	21.08011	21.61225	22.20781	-0.32328	-1.21203	0.565467	21.58323	-0.86715	-6.50603	0.415699	0.527159	0	0
615	sp|P14152	MDHC_MO	37 kDa	0.999556	4	100%	P14152	Mdh1	Malate deh	22.40132	23.1796	23.65911	24.09262	23.45055	23.38866	-0.3833	-1.43879	0.672189	23.36198	-0.86663	-6.50633	0.416107	0.527159	0	0
377	sp|Q9WTM	RUVB2_MC	51 kDa	0.998589	7	100%	Q9WTM5	Ruvbl2	RuvB-like 2	24.87667	25.25968	24.24096	24.34858	25.02871	25.64906	-0.37033	-1.39137	0.650717	24.90061	-0.86555	-6.50729	0.416661	0.527159	0	0
312	sp|Q06890	CLUS_MOU	52 kDa	0.999684	7	100%	Q06890	Clu	Clusterin	23.54192	23.0837	23.17588	22.67431	23.29486	23.44701	0.269214	-0.47183	1.010263	23.20295	0.864673	-6.50846	0.416751	0.527159	0	0
283	sp|Q9QYX7	PCLO_MOL	551 kDa	0.999324	8	100%	Q9QYX7	Pclo	Protein pic	20.14311	20.43764	18.60328	20.35687	21.07699	20.60297	-0.5247	-1.98292	0.933525	20.20348	-0.85869	-6.51334	0.420177	0.530997	0	0
279	sp|O55029	COPB2_MC	102 kDa	0.999399	8	100%	O55029	Copb2	Coatomer s	23.47247	23.11115	23.40375	25.75206	23.46798	23.28906	-0.60259	-2.27938	1.074	23.74941	-0.85781	-6.51412	0.42063	0.531074	0	0
561	sp|Q99JR1	SFXN1_MO	36 kDa	0.99881	5	100%	Q99JR1	Sfxn1	Sideroflexi	27.07349	24.86069	23.05498	27.04255	22.95534	24.37574	-1.06506	-4.04586	1.915749	24.8938	-0.85269	-6.51861	0.423274	0.533915	0	0
448	sp|Q6P9Z1	SMRD3_MC	55 kDa	0.999065	6	83%	Q6P9Z1	Smarcd3	SWI/SNF-re	20.44882	18.04715	19.53106	21.11497	20.76712	19.59368	0.663739	-1.20016	2.527636	19.91713	0.849817	-6.52111	0.424759	0.535291	0	0
384	sp|O08788	DCTN1_MC	142 kDa	0.997846	7	100%	O08788	Dctn1	Dynactin su	20.77595	21.78121	21.37536	21.65293	22.37911	22.26562	-0.38678	-1.48603	0.706473	21.70503	-0.84851	-6.52225	0.425436	0.535646	0	0
859.1	sp|A2A8L1	CHD5_MOL	223 kDa	0.99907	3	100%	A2A8L1	Chd5	Chromodor	20.5247	20.64026	20.70125	21.02256	20.74777	20.98767	-0.22559	-0.86517	0.413994	20.7707	-0.8395	-6.53045	0.429786	0.540621	0	0
282	sp|O35129	PHB2_MOL	33 kDa	0.999352	8	100%	O35129	Phb2	Prohibitin-	23.17873	23.53199	23.42839	23.62756	23.45151	23.54396	-0.21496	-0.82519	0.39527	23.46036	-0.83843	-6.53138	0.430347	0.540825	0	0
115	sp|Q8K4Z5	SF3A1_MO	89 kDa	0.99977	14	100%	Q8K4Z5	Sf3a1	Splicing fac	24.38031	23.60007	23.05652	23.54372	24.49486	23.7132	0.358233	-0.67735	1.393819	23.79811	0.825525	-6.54206	0.437486	0.549287	0	0
509	sp|Q8CH25	SLTM_MOL	117 kDa	0.999485	5	100%	Q8CH25	Sltm	SAFB-like tr	20.54435	20.50325	20.07133	20.34102	21.04583	22.2248	-0.46918	-1.82722	0.888849	20.78843	-0.82449	-6.54294	0.438036	0.549469	0	0
1008	sp|O54781	SRPK2_MO	77 kDa	0.994925	3	100%	O54781	Srpk2	SRSF protei	17.84747	17.02365	19.49351	20.82396	22.13089	18.39678	1.075826	-2.05966	4.211316	19.28604	0.818817	-6.54775	0.441047	0.552735	0	0
42	sp|Q9QXS1	PLEC_MOU	534 kDa	0.999798	22	91%	Q9QXS1	Plec	Plectin	21.27534	21.50789	19.03068	20.93863	21.87201	21.50565	-0.59138	-2.32525	1.142484	21.0217	-0.81396	-6.55185	0.443638	0.55547	0	0
1104	sp|P32067	LA_MOUSE	48 kDa	0.998931	2	100%	P32067	Ssb	Lupus La pr	20.01918	20.60156	21.11963	20.78247	19.76958	20.51648	-0.3307	-1.30301	0.641602	20.46815	-0.81168	-6.55377	0.444857	0.556482	0	0
64	sp|P27773	PDIA3_MO	57 kDa	0.999569	18	100%	P27773	Pdia3	Protein dis	24.38726	25.09534	25.66653	25.41644	24.77461	25.25201	-0.3118	-1.23713	0.613539	25.0987	-0.80397	-6.56025	0.448979	0.561121	0	0
197	sp|Q8K3I4	MYRIP_MO	95 kDa	0.999504	10	100%	Q8K3I4	Myrip	Rab effecto	22.9535	23.70629	22.69515	21.50695	23.04048	22.11664	0.45308	-0.90257	1.808728	22.66984	0.797588	-6.56553	0.452454	0.564878	0	0
395	sp|Q9DBG6	RPN2_MOL	69 kDa	0.999742	6	100%	Q9DBG6	Rpn2	Dolichyl-di	23.176	23.30928	23.77754	22.80779	23.16465	23.24731	0.251272	-0.49976	1.002308	23.24709	0.796313	-6.56695	0.452819	0.564878	0	0
152	sp|P02088	HBB1_MOU	16 kDa	0.999235	12	17%	P02088	Hbb-b1	Hemoglobi	27.20821	27.32291	27.35415	26.57818	26.98872	26.91582	0.244722	-0.4877	0.977145	27.06133	0.795263	-6.56782	0.453389	0.56507	0	0
1042	sp|P61514	RL37A_MC	10 kDa	0.9994	2	100%	P61514	Rpl37a	60S ribosor	18.52674	18.99861	18.9645	18.35634	18.12218	19.10495	-0.28216	-1.13391	0.569586	18.67889	-0.78956	-6.57232	0.456664	0.568147	0	0
1000	sp|Q9Z130	HNRDL_MC	34 kDa	0.995659	3	33%	Q9Z130	Hnrnpdl	Heterogene	15.64052	16.48313	17.55449	18.10705	15.99414	16.22825	-0.54309	-2.18411	1.097925	16.66793	-0.78979	-6.57196	0.456696	0.568147	0	0
241	sp|Q99JY0	ECHB_MOL	51 kDa	0.999181	9	100%	Q99JY0	Hadhb	Trifunction	24.82575	24.57784	24.83541	24.6437	24.59079	24.41817	0.204082	-0.41255	0.820711	24.64861	0.78774	-6.574	0.457492	0.568615	0	0
721	sp|Q07076	ANXA7_MC	50 kDa	0.998675	4	100%	Q07076	Anxa7	Annexin A7	19.86612	20.26402	20.35462	19.49889	20.14255	19.84985	0.250173	-0.50751	1.007856	19.99601	0.785874	-6.57553	0.458513	0.569363	0	0
451	sp|Q99MR	SRRT_MOU	100 kDa	0.999027	6	100%	Q99MR6	Srrt	Serrate RNA	22.35835	20.59488	21.76943	23.15277	21.79816	20.44457	0.577903	-1.1802	2.336009	21.68636	0.784441	-6.57634	0.459621	0.570216	0	0
723	sp|P62874	GBB1_MOU	37 kDa	0.998625	4	50%	P62874	Gnb1	Guanine nu	20.48363	20.24224	22.07345	20.94028	20.21039	20.33389	0.41702	-0.86757	1.701614	20.71398	0.774714	-6.58425	0.464974	0.576329	0	0
473	sp|O88738	BIRC6_MOI	532 kDa	0.997622	6	100%	O88738	Birc6	Baculoviral	19.78433	19.10187	19.46946	20.917	20.65113	21.40261	-0.50552	-2.06481	1.053767	20.22107	-0.77368	-6.58508	0.465544	0.57651	0	0
663	sp|P63044	VAMP2_MC	13 kDa	0.999194	4	50%	P63044	Vamp2	Vesicle-ass	22.39879	20.19269	20.82648	22.3521	22.06751	21.2371	0.503633	-1.05858	2.065851	21.51245	0.769349	-6.58857	0.467944	0.578953	0	0
67	sp|Q6URW	MYH14_MC	229 kDa	0.999296	18	61%	Q6URW6	Myh14	Myosin-14	20.49185	21.29343	20.83259	20.83399	20.99145	20.86522	-0.22558	-0.92547	0.474314	20.88476	-0.76713	-6.59071	0.468863	0.579129	0	0
1285	sp|Q8BK72	RT27_MOU	48 kDa	0.995366	2	100%	Q8BK72	Mrps27	28S ribosor	18.15662	18.08956	18.97539	17.58982	19.35127	19.48795	0.438648	-0.92517	1.802468	18.60844	0.767555	-6.59001	0.46894	0.579129	0	0
17	sp|E9QAT4	SC16A_MO	254 kDa	0.999775	33	100%	E9QAT4	Sec16a	Protein tra	25.65975	25.0515	25.36399	25.07134	25.17908	25.42681	0.217724	-0.45979	0.895238	25.29208	0.764874	-6.59251	0.470119	0.580056	0	0
886	sp|Q61595	KTN1_MOU	153 kDa	0.998844	3	100%	Q61595	Ktn1	Kinectin	20.08275	19.8958	19.93524	20.56563	19.58447	19.87592	-0.24496	-1.0121	0.522181	19.98997	-0.76001	-6.59639	0.472834	0.582876	0	0
647	sp|P14094	AT1B1_MO	35 kDa	0.99935	4	100%	P14094	Atp1b1	Sodium/po	22.14003	22.47536	23.12338	22.05102	22.28595	22.21878	0.268067	-0.57393	1.110062	22.38402	0.758314	-6.59764	0.47387	0.583622	0	0
1076	sp|Q5I012	S38AA_MO	117 kDa	0.999159	2	100%	Q5I012	Slc38a10	Putative so	19.62342	20.02419	20.14219	18.95157	19.26409	19.13336	0.306861	-0.65958	1.273298	19.52314	0.757738	-6.59785	0.474418	0.583766	0	0
556	sp|Q9CQE8	RTRAF_MC	28 kDa	0.998864	5	100%	Q9CQE8	RTRAF	RNA transc	22.03878	21.55377	21.01344	21.64723	21.6659	22.40987	-0.29759	-1.24356	0.648388	21.7215	-0.75073	-6.60339	0.478355	0.588078	0	0
1106	sp|A2RTL5	RSRC2_MC	44 kDa	0.99891	2	100%	A2RTL5	Rsrc2	Arginine/se	17.73595	19.10717	19.39336	18.56739	19.2903	20.06157	-0.43884	-1.84101	0.963343	19.02596	-0.74688	-6.60641	0.480528	0.589968	0	0
293	sp|O35643	AP1B1_MO	104 kDa	0.998995	8	12%	O35643	Ap1b1	AP-1 comp	17.59438	19.14216	18.71509	18.10126	17.95741	18.02964	-0.3354	-1.40766	0.736867	18.25666	-0.74646	-6.60674	0.480763	0.589968	0	0
417	sp|Q91V61	SFXN3_MO	35 kDa	0.999544	6	100%	Q91V61	Sfxn3	Sideroflexi	23.16663	23.23275	23.11899	22.09398	22.64576	22.77427	0.276794	-0.61996	1.173546	22.83873	0.735832	-6.61515	0.486677	0.596368	0	0
154	tr|A0A1W2	A0A1W2P7	92 kDa	0.998967	12	8%	A0A1W2P7	Gm32717	Predicted g	17.95369	18.27901	15.44668	18.25918	18.91957	17.6597	-0.62598	-2.65647	1.404498	17.75297	-0.73572	-6.6151	0.486858	0.596368	0	0
840	sp|P62911	RL32_MOU	16 kDa	0.999242	3	100%	P62911	Rpl32	60S ribosor	18.28103	20.69263	18.42216	14.75673	17.89942	16.03677	1.038826	-2.35028	4.427932	17.68146	0.731489	-6.61837	0.489275	0.598788	0	0
37	sp|Q921M	SF3B3_MO	136 kDa	0.999613	23	100%	Q921M3	Sf3b3	Splicing fac	25.64794	25.23526	24.34345	24.97936	25.86222	24.62941	0.336525	-0.76367	1.436715	25.11627	0.729962	-6.61955	0.49015	0.599317	0	0
1087	sp|Q64737	PUR2_MOI	108 kDa	0.999065	2	100%	Q64737	Gart	Trifunction	22.93929	22.68533	22.87242	22.97413	23.01155	22.59121	0.190864	-0.43652	0.818253	22.84565	0.724084	-6.62438	0.493229	0.602539	0	0
1244	sp|Q9CQT1	MTNA_MOI	39 kDa	0.997045	2	100%	Q9CQT1	Mri1	Methylthio	18.89307	18.24818	17.94123	17.54076	18.2714	18.46826	0.275548	-0.6502	1.215861	18.22715	0.723411	-6.62457	0.49391	0.602828	0	0
345	sp|P62823	RAB3C_MC	26 kDa	0.99936	7	43%	P62823	Rab3c	Ras-related	21.05358	21.422	21.33234	21.5557	20.83664	20.91379	-0.22298	-0.9581	0.512142	21.18568	-0.72194	-6.62602	0.494461	0.602958	0	0
1044	sp|P62307	RUXF_MOL	10 kDa	0.999395	2	100%	P62307	Snrpf	Small nucle	19.49933	18.93213	20.63188	19.08913	18.99318	19.95007	0.364319	-0.8579	1.586534	19.50597	0.711352	-6.6337	0.500882	0.610159	0	0
1252	sp|Q6NSW	SPKAP_MC	185 kDa	0.996787	2	100%	Q6NSW3	Sphkap	A-kinase an	16.86386	16.99018	17.16571	15.96874	16.41088	16.67591	0.268568	-0.63249	1.169567	16.67921	0.710494	-6.63448	0.501266	0.610159	0	0
1284	sp|Q80T69	RSBN1_MC	89 kDa	0.995403	2	100%	Q80T69	Rsbn1	Lysine-spec	20.7875	21.33942	20.92294	21.14441	21.03247	20.8425	-0.19447	-0.85057	0.461629	21.01154	-0.70548	-6.63842	0.504013	0.612953	0	0
393	sp|P01942	HBA_MOUS	15 kDa	0.999751	6	100%	P01942	Hba	Hemoglobi	26.46401	26.63959	26.74094	26.81191	26.31126	26.62693	-0.18741	-0.82663	0.451822	26.59911	-0.6978	-6.64413	0.508514	0.617872	0	0
349	sp|Q8K3H0	DP13A_MC	79 kDa	0.999321	7	100%	Q8K3H0	Appl1	DCC-intera	22.19632	22.04202	22.30079	21.03712	21.46042	22.02368	0.284905	-0.69085	1.260656	21.84339	0.696805	-6.64455	0.509378	0.618368	0	0
547	sp|Q62158	TRI27_MOI	59 kDa	0.99902	5	100%	Q62158	Trim27	Zinc finger	19.25237	18.23601	17.57046	19.03589	19.73267	18.01041	0.424394	-1.03513	1.883919	18.63963	0.693918	-6.64668	0.511075	0.619873	0	0
808	sp|Q8K2B3	SDHA_MOL	73 kDa	0.999462	3	100%	Q8K2B3	Sdha	Succinate d	21.86345	22.38283	21.19742	21.06094	21.97075	22.58293	-0.3317	-1.47796	0.814565	21.84305	-0.69057	-6.64914	0.513046	0.621529	0	0
517	sp|Q9R1A8	COP1_MOL	80 kDa	0.999365	5	100%	Q9R1A8	Cop1	E3 ubiquiti	22.08696	21.79764	20.48077	20.93977	22.61125	21.22947	0.404036	-0.99327	1.801346	21.52431	0.690046	-6.64952	0.513357	0.621529	0	0
426	sp|O35526	STX1A_MO	33 kDa	0.999408	6	100%	O35526	Stx1a	Syntaxin-1/	22.50487	21.94678	20.98354	21.50129	21.57472	22.62293	-0.33596	-1.50554	0.833629	21.85569	-0.68549	-6.65285	0.516049	0.623949	0	0
1278.2	sp|Q7TSF1	DSG1B_MC	114 kDa	0.995714	2	100%	Q7TSF1	Dsg1b	Desmoglei	15.88352	20.07099	16.13897	15.54141	17.73233	16.55523	-0.80427	-3.61215	2.003609	16.98708	-0.68356	-6.65426	0.517197	0.623949	0	0
1278.3	sp|Q61495	DSG1A_MC	115 kDa	0.995714	2	100%	Q61495	Dsg1a	Desmoglei	15.88352	20.07099	16.13897	15.54141	17.73233	16.55523	-0.80427	-3.61215	2.003609	16.98708	-0.68356	-6.65426	0.517197	0.623949	0	0
1279	sp|O89112	LANC1_MC	45 kDa	0.99571	2	100%	O89112	Lancl1	Glutathion	15.88352	20.07099	16.13897	15.54141	17.73233	16.55523	-0.80427	-3.61215	2.003609	16.98708	-0.68356	-6.65426	0.517197	0.623949	0	0
189	sp|Q91VD9	NDUS1_MC	80 kDa	0.999673	10	100%	Q91VD9	Ndufs1	NADH-ubiq	22.85896	22.89416	23.25365	22.70019	22.58183	23.94331	-0.28107	-1.2689	0.706753	23.03868	-0.67903	-6.65754	0.519884	0.626586	0	0
62	sp|P11499	HS90B_MC	83 kDa	0.999751	18	50%	P11499	Hsp90ab1	Heat shock	24.64623	25.0828	25.59178	25.30348	24.47295	25.07175	-0.24902	-1.12579	0.627746	25.02817	-0.67709	-6.65906	0.520933	0.626586	0	0
438	sp|Q9EQK5	MVP_MOU	96 kDa	0.99922	6	100%	Q9EQK5	Mvp	Major vault	22.52034	21.75879	20.57396	21.97124	21.34734	21.73586	-0.34142	-1.54451	0.861672	21.65125	-0.67723	-6.65884	0.520955	0.626586	0	0
285	sp|P10630	IF4A2_MO	46 kDa	0.999295	8	25%	P10630	Eif4a2	Eukaryotic	18.98459	18.80082	19.41474	19.33311	19.11248	18.76908	0.202934	-0.51125	0.917117	19.06914	0.676313	-6.6598	0.521231	0.626586	0	0
1072	sp|Q9WUC	LY6H_MOL	15 kDa	0.999194	2	100%	Q9WUC3	Ly6h	Lymphocyt	23.806	24.17645	24.31532	24.8389	23.56914	23.51469	-0.27986	-1.28051	0.720787	24.03675	-0.66744	-6.66585	0.526808	0.632729	0	0
264	sp|Q5DQR4	STB5L_MOI	132 kDa	0.999657	8	88%	Q5DQR4	Stxbp5l	Syntaxin-bi	24.28436	25.6371	24.3766	24.87379	24.93977	20.66351	0.80878	-2.12654	3.744094	24.12919	0.657546	-6.67285	0.532767	0.639321	0	0
1197	sp|Q8C522	ENDD1_MC	55 kDa	0.997966	2	100%	Q8C522	Endod1	Endonucle	17.41515	17.38588	16.37274	14.26591	16.91452	17.34835	0.567421	-1.50203	2.636873	16.61709	0.654336	-6.6751	0.534709	0.641084	0	0
286	sp|O08709	PRDX6_MC	25 kDa	0.999289	8	100%	O08709	Prdx6	Peroxiredo	23.58778	23.54127	23.24734	23.24801	23.38377	22.84712	0.194162	-0.52125	0.909577	23.30922	0.645962	-6.68121	0.539537	0.6463	0	0
354	sp|Q61136	PRP4B_MC	117 kDa	0.999238	7	100%	Q61136	Prpf4b	Serine/thr	21.36521	20.67229	19.84939	20.2666	21.38417	20.73978	0.306694	-0.83044	1.443825	20.71399	0.643642	-6.68252	0.54121	0.647732	0	0
477	sp|Q9Z2T6	KRT85_MC	56 kDa	0.996805	6	83%	Q9Z2T6	Krt85	Keratin, typ	17.63642	21.38056	17.75515	16.35591	17.36254	17.35905	-0.78047	-3.67913	2.118186	17.97494	-0.64255	-6.68327	0.541874	0.647955	0	0
711	sp|P68181	KAPCB_MC	41 kDa	0.998773	4	50%	P68181	Prkacb	cAMP-depe	16.99514	17.78472	15.17535	14.10718	17.28583	15.64124	0.641061	-1.74457	3.026696	16.16491	0.641278	-6.68415	0.542654	0.648316	0	0
407	sp|Q00623	APOA1_MC	31 kDa	0.9996	6	100%	Q00623	Apoa1	Apolipopro	23.00534	22.96098	23.44589	23.83148	22.93709	23.24269	-0.21561	-1.02096	0.589744	23.23724	-0.63723	-6.6872	0.54488	0.650403	0	0
1134	sp|O35405	PLD3_MOL	54 kDa	0.998581	2	100%	O35405	Pld3	Phospholip	19.63274	18.3577	18.81846	19.07016	18.62872	18.92696	0.2417	-0.66462	1.148021	18.90579	0.635753	-6.68805	0.545937	0.651091	0	0
510	sp|P47738	ALDH2_MC	57 kDa	0.99947	5	100%	P47738	Aldh2	Aldehyde d	22.22193	22.29382	21.78754	21.87387	20.9932	21.59611	-0.25371	-1.21262	0.705197	21.79441	-0.63142	-6.69088	0.548701	0.653011	0	0
1238	sp|Q8VE80	THOC3_MC	39 kDa	0.997247	2	100%	Q8VE80	Thoc3	THO compl	20.55642	25.87945	20.91534	19.5815	20.01091	19.23768	-1.07199	-5.12507	2.981099	21.03022	-0.63118	-6.69105	0.548847	0.653011	0	0
252	sp|Q61414	K1C15_MC	49 kDa	0.998201	9	22%	Q61414	Krt15	Keratin, typ	18.2861	19.88792	22.21774	20.68979	19.85278	21.6699	-0.63033	-3.01446	1.753792	20.43404	-0.63095	-6.6912	0.548992	0.653011	0	0
843	sp|Q8BMC	NOP9_MOI	70 kDa	0.999222	3	100%	Q8BMC4	Nop9	Nucleolar p	23.19132	23.37565	22.84085	23.57728	23.68111	23.34961	-0.19642	-0.94447	0.55162	23.33597	-0.62498	-6.6955	0.552422	0.656516	0	0
478	sp|Q8BML9	SYQ_MOUS	88 kDa	0.996522	6	100%	Q8BML9	Qars	Glutamine-	20.39094	20.29395	20.79928	20.58554	20.48987	20.29174	0.169619	-0.48324	0.822479	20.47522	0.61838	-6.69991	0.556514	0.660799	0	0
755	sp|P15920	VPP2_MOL	98 kDa	0.999606	4	50%	P15920	Atp6v0a2	V-type prot	19.06511	19.35388	18.35353	19.56282	19.05765	18.33881	-0.25974	-1.26715	0.747666	18.9553	-0.6153	-6.70168	0.558673	0.662783	0	0
217	sp|Q5ND34	WDR81_M	212 kDa	0.998748	10	100%	Q5ND34	Wdr81	WD repeat-	20.87734	21.34136	19.88104	20.54273	21.7146	21.56276	-0.32462	-1.59786	0.948614	20.98664	-0.60845	-6.7062	0.562948	0.666829	0	0
879	sp|P35564	CALX_MOU	67 kDa	0.998936	3	100%	P35564	Canx	Calnexin	17.62096	17.24647	17.71825	18.14069	18.07511	18.83872	-0.27052	-1.33189	0.790848	17.94003	-0.60826	-6.70633	0.563067	0.666829	0	0
254	sp|P97496	SMRC1_MC	123 kDa	0.996795	9	11%	P97496	Smarcc1	SWI/SNF cc	17.99677	17.91573	17.32152	18.20306	18.29381	18.10156	-0.20275	-1.00526	0.59976	17.97207	-0.60133	-6.7111	0.567167	0.671098	0	0
460	sp|Q9DBH5	LMAN2_M	40 kDa	0.998899	6	100%	Q9DBH5	Lman2	Vesicular ir	20.93232	21.46101	22.26293	20.61199	20.83561	21.11186	0.281999	-0.8437	1.407697	21.20262	0.597827	-6.71311	0.569608	0.673398	0	0
247	sp|P62631	EF1A2_MO	50 kDa	0.998875	9	22%	P62631	Eef1a2	Elongation	19.64305	19.51025	19.98301	20.70349	20.27363	18.75752	0.309476	-0.93312	1.552074	19.81183	0.594357	-6.71535	0.571794	0.675044	0	0
734	sp|P56959	FUS_MOUS	53 kDa	0.998377	4	100%	P56959	Fus	RNA-bindir	21.80268	21.18087	21.18505	21.89109	20.36085	20.49338	0.59441	-1.79353	2.98235	20.15232	0.594037	-6.71555	0.571996	0.675044	0	0
1177.2	tr|A0A075	A0A075B5	13 kDa	0.998168	2	50%	A0A075B5	Ighv1-74	Immunogl	19.81061	20.27385	21.1667	20.37539	19.63427	18.94972	0.337541	-1.02403	1.699114	20.03509	0.591611	-6.71711	0.573528	0.676264	0	0
1127	sp|Q920I9	WDR7_MO	163 kDa	0.998654	2	100%	Q920I9	Wdr7	WD repeat-	20.11111	17.67665	18.66057	21.04512	18.16841	19.84115	-0.54095	-2.73165	1.649764	19.2505	-0.58928	-6.7186	0.575004	0.677415	0	0
318	sp|Q00493	CBPE_MOU	53 kDa	0.999646	7	100%	Q00493	Cpe	Carboxype	22.89779	24.25512	23.72315	22.69235	23.24539	21.80169	0.372389	-1.13855	1.883326	23.10258	0.588167	-6.7193	0.575706	0.677654	0	0
513	sp|Q9DCN2	NB5R3_MC	34 kDa	0.999426	5	100%	Q9DCN2	Cyb5r3	NADH-cyto	22.0716	21.98739	23.01042	22.38095	21.97085	22.04763	0.212301	-0.66055	1.085149	22.24481	0.579838	-6.72466	0.580904	0.68318	0	0
471	sp|P62137	PP1A_MOL	38 kDa	0.997925	6	33%	P62137	Ppp1ca	Serine/thre	19.60489	18.91981	18.94128	17.75669	19.18296	20.03897	0.337886	-1.05636	1.73218	19.0741	0.578339	-6.72551	0.58195	0.683816	0	0
689	sp|P46471	PRS7_MOL	49 kDa	0.999	4	100%	P46471	Psmc2	26S protea	20.829	21.20927	21.50021	20.87381	20.58285	21.41555	-0.19553	-1.01909	0.628041	21.06845	-0.5651	-6.73395	0.590203	0.69237	0	0
883	sp|Q9CU65	ZMYM2_M	155 kDa	0.998886	3	100%	Q9CU65	Zmym2	Zinc finger	17.23808	18.42317	18.76093	18.4447	17.7189	17.64749	-0.26582	-1.3885	0.856856	18.03888	-0.56505	-6.73375	0.590455	0.69237	0	0
1154.2	tr|E9PW43	E9PW43_N	12 kDa	0.998394	2	100%	E9PW43	Gm10320	Protein tra	19.65571	20.12876	20.76477	20.23821	19.90625	20.58927	-0.20984	-1.0971	0.677429	20.21383	-0.5638	-6.73461	0.591171	0.69237	0	0
1071	sp|P11531	DMD_MOU	426 kDa	0.999197	2	100%	P11531	Dmd	Dystrophin	20.4741	19.62792	19.83168	19.8011	20.29718	20.56238	0.203854	-0.65835	1.06606	20.09906	0.563641	-6.7347	0.591272	0.69237	0	0
126	sp|Q91YQ5	RPN1_MOI	69 kDa	0.999763	13	100%	Q91YQ5	Rpn1	Dolichyl-di	24.3914	24.68795	24.83312	23.73024	24.10211	24.29589	0.204166	-0.66459	1.072964	24.34012	0.560289	-6.73675	0.593431	0.694299	0	0
924	tr|A0A140	A0A140T8I	13 kDa	0.998347	3	67%	A0A140T8I	Igkv8-24	Immunogl	19.90754	20.00146	19.42872	19.12867	19.88892	20.88543	-0.26346	-1.40259	0.875674	19.87326	-0.55193	-6.74171	0.598916	0.700112	0	0
102	tr|G3X8Q1	G3X8Q1_M	243 kDa	0.999642	15	100%	G3X8Q1	Cabin1	Calcineurir	22.72226	24.03761	22.60114	22.75351	23.67907	22.9811	-0.25658	-1.38222	0.86906	23.12911	-0.54397	-6.74646	0.604091	0.70525	0	0
13	sp|E9Q7G0	NUMA1_M	236 kDa	0.999659	35	100%	E9Q7G0	Numa1	Nuclear m	24.72768	24.60314	25.0132	25.50108	24.84293	25.00648	-0.17563	-0.94514	0.593875	24.94909	-0.54324	-6.74712	0.604351	0.70525	0	0
930.1	sp|P60764	RAC3_MOL	21 kDa	0.999224	3	100%	P60764	Rac3	Ras-related	19.18671	19.40853	19.42759	19.44826	19.70052	18.98329	0.158244	-0.54281	0.859299	19.35915	0.537251	-6.75064	0.608261	0.709202	0	0
908	sp|Q9Z0P4	PALM_MOI	42 kDa	0.998551	3	100%	Q9Z0P4	Palm	Paralemmi	21.56623	21.34025	21.07598	21.3167	21.40037	21.87418	-0.16284	-0.88662	0.560928	21.42895	-0.53551	-6.75165	0.609398	0.709917	0	0
96	sp|Q8VDP4	CCAR2_MC	103 kDa	0.999847	15	100%	Q8VDP4	Ccar2	Cell cycle a	24.48677	24.21227	23.42988	23.23853	24.5259	24.26878	0.240995	-0.83517	1.317162	24.02702	0.534416	-6.75208	0.610325	0.710387	0	0
1267	sp|Q80VD1	FA98B_MC	45 kDa	0.996327	2	100%	Q80VD1	Fam98b	Protein FA	17.64117	15.69858	19.23325	18.91385	18.33087	19.07164	0.507073	-1.76674	2.780888	18.14823	0.532189	-6.75337	0.611784	0.711455	0	0
1158	sp|P47964	RL36_MOL	12 kDa	0.998336	2	100%	P47964	Rpl36	60S ribosor	19.08974	20.41369	19.74373	18.58569	19.86258	19.63001	-0.27112	-1.48992	0.947684	19.40757	-0.53086	-6.75415	0.612656	0.711455	0	0
1149	sp|Q5EG47	AAPK1_MC	64 kDa	0.998487	2	100%	Q5EG47	Prkaa1	5'-AMP-act	18.92212	19.95578	19.12417	17.8412	18.57772	19.76345	-0.31214	-1.71789	1.093611	19.03074	-0.5299	-6.7547	0.613286	0.711455	0	0
1208	tr|A0A0J9	A0A0J9YUD	233 kDa	0.997821	2	100%	A0A0J9YUD	Nup205	Nucleopori	18.11076	16.23971	17.24764	17.96186	17.88223	18.14149	0.29919	-1.04845	1.64683	17.59728	0.529814	-6.75475	0.613342	0.711455	0	0
381	sp|P16460	ASSY_MOU	47 kDa	0.998214	7	100%	P16460	Ass1	Argininosu	22.79178	22.7732	23.17322	23.0631	22.71076	22.34609	0.16446	-0.58166	0.910582	22.80969	0.524627	-6.75795	0.616547	0.714563	0	0
1120	sp|Q8K354	CBR3_MOL	31 kDa	0.998731	2	50%	Q8K354	Cbr3	Carbonyl re	20.20173	21.27588	20.73007	19.9929	19.79225	20.15657	-0.23376	-1.30283	0.835306	20.35823	-0.52182	-6.75934	0.618599	0.716328	0	0
834	sp|Q3THE2	ML12B_MC	20 kDa	0.99929	3	100%	Q3THE2	Myl12b	Myosin reg	21.38875	21.27953	21.52952	22.23487	21.48497	20.02844	0.286799	-1.02981	1.603411	21.32435	0.519841	-6.76047	0.619007	0.716857	0	0
434	sp|P10649	GSTM1_MC	26 kDa	0.999283	6	83%	P10649	Gstm1	Glutathion	20.92802	21.39024	22.893	21.88715	21.39811	21.08552	0.285405	-1.02559	1.596403	21.59701	0.519528	-6.76065	0.620113	0.716857	0	0
572	sp|Q78JE5	FBX22_MO	44 kDa	0.998299	5	100%	Q78JE5	Fbxo22	F-box only	21.78488	20.53452	20.9012	22.62494	21.95091	22.44711	-0.32319	-1.81428	1.167897	21.70726	-0.51726	-6.76194	0.621614	0.717935	0	0
363	sp|Q9ERA6	TFP11_MO	96 kDa	0.99909	7	100%	Q9ERA6	Tfip11	Tuftelin-int	20.45983	18.80913	20.29682	21.20046	19.84115	19.64128	0.315644	-1.14272	1.774008	20.04145	0.516515	-6.76236	0.622104	0.717935	0	0
1247	sp|P63085	MK01_MOI	41 kDa	0.99695	2	100%	P63085	Mapk1	Mitogen-ac	18.798	18.84499	17.98721	18.19567	18.00167	16.90873	0.279164	-1.01875	1.577081	18.12271	0.51329	-6.76417	0.62424	0.719537	0	0
86	sp|P68372	TBB4B_MC	50 kDa	0.998318	17	6%	P68372	Tubb4b	Tubulin be	24.69166	24.36238	24.89946	24.5802	24.17673	24.36194	0.154445	-0.5637	0.872587	24.51206	0.511874	-6.76517	0.62498	0.719537	0	0
1091	tr|A0A140	A0A140T8I	10 kDa	0.999019	2	100%	A0A140T8I	Igkv9-123	Immunogl	18.58958	19.4859	19.83884	19.92601	18.52368	19.0933	-0.21771	-1.23355	0.798142	19.09288	-0.51144	-6.76521	0.625468	0.719537	0	0
443	sp|O35691	PININ_MOI	82 kDa	0.999181	6	100%	O35691	Pnn	Pinin	21.90102	20.93412	20.57801	21.31886	21.20792	20.81976	0.204738	-0.75101	1.160491	21.12662	0.511216	-6.76534	0.625615	0.719537	0	0
1113	sp|P99027	RLA2_MOL	12 kDa	0.998821	2	100%	P99027	Rplp2	60S acidic r	19.81857	19.18302	18.89206	19.17815	19.04936	18.98392	-0.12836	-0.73089	0.474157	19.05085	-0.50708	-6.76784	0.628168	0.721583	0	0
964	sp|Q9CQ26	STABP_MO	49 kDa	0.997689	3	100%	Q9CQ26	Stambp	STAM-bind	23.84574	24.21123	23.48265	23.34506	23.48379	23.75128	-0.16513	-0.94091	0.610646	23.68662	-0.50664	-6.76808	0.628458	0.721583	0	0
1276	sp|Q62465	VAT1_MOU	43 kDa	0.995771	2	100%	Q62465	Vat1	Synaptic ve	19.39812	19.02562	18.71781	18.04646	19.79436	21.041	-0.40093	-2.29546	1.493605	19.17056	-0.50503	-6.76878	0.629727	0.722328	0	0
226	sp|Q8BGC0	HTSF1_MO	86 kDa	0.999777	9	100%	Q8BGC0	Htatsf1	HIV Tat-spe	22.57438	22.12624	23.23762	22.74238	22.81128	23.19069	0.187993	-0.70079	1.076773	22.78043	0.504243	-6.76929	0.630172	0.722328	0	0
529	sp|P10605	CATB_MOU	37 kDa	0.999267	5	100%	P10605	Ctsb	Cathepsin E	22.65574	22.34416	23.29181	22.49224	21.89326	22.40452	0.199963	-0.74959	1.149513	22.51362	0.502553	-6.77015	0.631376	0.723096	0	0

855	sp|Q9D0T1	NH2L1_MO	14 kDa	0.999084	3	100%	Q9D0T1	Snu13	NHP2-like p	20.79625	21.62489	20.97691	20.37932	21.00473	21.33955	-0.18863	-1.10265	0.725402	21.02028	-0.49209	-6.77591	0.638314	0.730181	0	0
260	sp|P60335	PCBP1_MO	37 kDa	0.99974	8	62%	P60335	Pcbp1	Poly(rC)-bi	23.66607	23.44202	23.89618	24.30418	23.86316	23.13573	0.181157	-0.69741	1.059726	23.71789	0.491555	-6.77622	0.638656	0.730181	0	0
865	sp|Q8BGH2	SAM50_MO	52 kDa	0.999029	3	100%	Q8BGH2	Samm50	Sorting and	21.00546	21.3021	20.98856	20.29583	21.17737	22.33767	-0.25474	-1.49312	0.983652	21.1845	-0.49089	-6.77651	0.639178	0.730181	0	0
828	sp|P62320	SMD3_MOU	14 kDa	0.999359	3	100%	P62320	Snrpd3	Small nucle	18.96889	18.69494	18.70098	18.22903	19.91639	19.88172	0.260191	-1.01459	1.53497	19.06532	0.487089	-6.77855	0.64173	0.731956	0	0
132	sp|Q9Z1X4	ILF3_MOUS	96 kDa	0.999596	13	100%	Q9Z1X4	Ilf3	Interleukin	23.28296	22.73677	22.81955	23.62603	23.39284	23.66521	-0.17755	-1.04695	0.691842	23.25389	-0.48686	-6.77874	0.641811	0.731956	0	0
342	sp|Q6R0H7	GNAS1_MO	122 kDa	0.999386	7	86%	Q6R0H7	Gnas	Guanine nu	23.17557	23.6719	23.61081	23.30896	23.02393	23.26202	-0.14419	-0.85363	0.565236	23.3422	-0.48377	-6.7805	0.643776	0.73358	0	0
15	sp|P39053	DYN1_MOU	98 kDa	0.999724	34	74%	P39053	Dnm1	Dynamin-1	26.61206	26.34164	25.67204	26.39923	26.76094	26.85276	-0.18286	-1.08944	0.723725	26.43978	-0.48084	-6.78194	0.645865	0.735343	0	0
1150	sp|O35218	CPSF2_MO	88 kDa	0.998475	2	100%	O35218	Cpsf2	Cleavage ar	19.18184	19.45775	19.45249	19.04598	19.69368	19.4092	0.138357	-0.54962	0.826328	19.37349	0.478663	-6.7832	0.647224	0.736272	0	0
1024	tr|A0A0B4	A0A0B4J1I	13 kDa	0.999697	2	100%	A0A0B4J1I	Igkv16-104	Immunoglo	21.38847	21.58627	20.24428	21.89224	21.79185	20.68011	-0.24467	-1.47781	0.988456	21.26387	-0.47351	-6.78572	0.650892	0.738682	0	0
1025	sp|Q9D032	SSBP3_MO	40 kDa	0.999644	2	50%	Q9D032	Ssbp3	Single-stran	21.38847	21.58627	20.24428	21.89224	21.79185	20.68011	-0.24467	-1.47781	0.988456	21.26387	-0.47351	-6.78572	0.650892	0.738682	0	0
739	sp|Q569Z6	TR150_MO	108 kDa	0.998309	4	100%	Q569Z6	Thrap3	Thyroid ho	20.76209	19.6964	20.58644	22.44865	20.76132	18.67997	0.428276	-1.73076	2.587308	20.48915	0.473386	-6.78578	0.650977	0.738682	0	0
816	sp|P01648	KV5AF_MO	12 kDa	0.999423	3	33%	P01648	NA	Ig kappa ch	21.41844	22.40012	21.30904	20.62245	20.90759	21.29694	-0.22815	-1.38337	0.927073	21.32577	-0.47131	-6.78687	0.652387	0.739662	0	0
966	sp|Q8JZQ2	AFG32_MO	90 kDa	0.99763	3	100%	Q8JZQ2	Afg3l2	AFG3-like p	19.27651	19.40219	20.08141	20.42771	19.80819	19.86487	-0.17622	-1.0855	0.733066	19.81015	-0.46202	-6.79171	0.658632	0.746089	0	0
333	sp|Q70FJ1	AKAP9_MO	436 kDa	0.99948	7	100%	Q70FJ1	Akap9	A-kinase an	17.06258	19.63767	18.42371	19.14454	20.46672	18.3516	-0.3936	-2.42958	1.642382	18.8478	-0.46135	-6.79198	0.659155	0.746089	0	0
1241.2	sp|Q3THW	H2AV_MOU	14 kDa	0.997171	2	50%	Q3THW5	H2afv	Histone H2	19.58837	20.26592	21.09828	20.96858	19.73209	19.88424	-0.23333	-1.4457	0.979036	20.25625	-0.45929	-6.79303	0.660557	0.747052	0	0
292	sp|Q6PE01	SNR40_MO	39 kDa	0.999016	8	100%	Q6PE01	Snrnp40	U5 small nu	22.10031	21.63682	21.56255	22.63607	22.17995	20.90991	0.220006	-0.92842	1.368431	21.8376	0.457176	-6.7941	0.662002	0.748063	0	0
270	sp|P63101	1433Z_MO	28 kDa	0.999587	8	62%	P63101	Ywhaz	14-3-3 prot	21.68044	21.91043	22.10508	22.03288	22.11662	21.57364	0.128397	-0.5613	0.818094	21.90318	0.443098	-6.80125	0.671484	0.758038	0	0
499	sp|P61982	1433G_MO	28 kDa	0.999552	5	40%	P61982	Ywhag	14-3-3 prot	20.95297	21.07012	21.48384	22.38251	21.19212	20.79487	-0.20619	-1.3178	0.905408	21.31274	-0.44267	-6.80131	0.671948	0.758038	0	0
940	sp|P46660	AINX_MOU	55 kDa	0.998031	3	100%	P46660	Ina	Alpha-inter	17.1509	16.13965	16.3956	18.83853	19.42203	19.41233	-0.47399	-3.04597	2.097989	17.89317	-0.4398	-6.80271	0.673923	0.759634	0	0
215	sp|Q6PIE5	AT1A2_MO	112 kDa	0.999015	10	10%	Q6PIE5	Atp1a2	Sodium/po	17.61301	18.61843	18.79411	17.05493	16.99542	18.52112	-0.26398	-1.73244	1.204482	17.93284	-0.429	-6.8079	0.681384	0.767406	0	0
882	sp|P01629	KV2A4_MO	12 kDa	0.998891	3	33%	P01629	NA	Ig kappa ch	20.59663	20.97024	19.3243	20.08337	21.16943	20.70445	-0.22256	-1.48174	1.036609	20.47474	-0.42181	-6.81128	0.686368	0.772378	0	0
160	sp|O35219	KCNH2_MO	127 kDa	0.999658	11	100%	O35219	Kcnh2	Potassium	23.00372	24.68984	23.60599	23.95935	23.08892	21.91815	-0.28957	-1.96279	1.383647	23.37766	-0.413	-6.81536	0.692504	0.778078	0	0
1190	sp|Q99NB8	UBQL4_MO	64 kDa	0.998054	2	50%	Q99NB8	Ubqln4	Ubiquilin-4	17.14193	18.76924	20.51699	20.42141	20.19039	19.82646	-0.38927	-2.63915	1.860615	19.47774	-0.41289	-6.81541	0.692582	0.778078	0	0
528	sp|P51655	GPC4_MOU	63 kDa	0.999272	5	100%	P51655	Gpc4	Glypican-4	20.94028	21.04677	21.73373	20.69238	20.94758	22.54985	-0.22247	-1.51777	1.072824	21.31843	-0.40988	-6.81679	0.694682	0.779792	0	0
168	tr|A8DUK4	A8DUK4_M	16 kDa	0.999378	11	27%	A8DUK4	Hbb-bs	Beta-globin	24.13773	24.42798	27.41344	27.18335	22.00268	23.74592	-0.60114	-4.13889	2.936618	24.81852	-0.40551	-6.81877	0.697746	0.782112	0	0
773	sp|P03977	KV3A4_MO	12 kDa	0.995804	4	25%	P03977	NA	Ig kappa ch	19.13728	19.53145	20.08325	19.0562	18.47123	19.6619	-0.18593	-1.28075	0.90889	19.32355	-0.40528	-6.81887	0.697902	0.782112	0	0
779	sp|Q9CY27	TECR_MOU	36 kDa	0.994712	4	100%	Q9CY27	Tecr	Very-long-c	20.79924	22.84498	21.00096	17.94505	20.66127	20.31157	0.453287	-2.22633	3.132907	20.59385	0.403692	-6.81958	0.699016	0.782693	0	0
873	sp|P35279	RAB6A_MO	24 kDa	0.998972	3	67%	P35279	Rab6a	Ras-related	19.85947	18.92636	18.51753	18.52958	19.59504	19.90064	0.205154	-1.01002	1.420331	19.22144	0.402894	-6.81994	0.699575	0.782693	0	0
953	sp|Q9R190	MTA2_MOU	75 kDa	0.997887	3	100%	Q9R190	Mta2	Metastasis-	19.05368	20.55486	21.25122	20.84917	20.75787	20.35336	-0.23154	-1.61619	1.153108	20.47003	-0.39906	-6.82165	0.702266	0.784947	0	0
243	sp|Q9D361	SNR48_MO	39 kDa	0.999104	9	100%	Q9D361	Snrnp48	U11/U12 sr	23.8802	23.84941	22.73898	23.57623	24.06127	23.74127	-0.16215	-1.13354	0.809227	23.64123	-0.39837	-6.82195	0.702748	0.784947	0	0
1059	sp|P01845	LAC3_MOU	11 kDa	0.999277	2	50%	P01845	Iglc3	Ig lambda-3	18.96789	18.93317	21.05773	21.01257	20.48814	19.71716	0.28362	-1.42314	1.990376	20.02944	0.396566	-6.82275	0.704018	0.785719	0	0
1213	sp|Q9Z2X8	KEAP1_MO	70 kDa	0.997758	2	100%	Q9Z2X8	Keap1	Kelch-like E	20.26591	19.91346	21.23667	21.64515	20.0402	20.6109	-0.20891	-1.49667	1.07885	20.61872	-0.38715	-6.82686	0.710655	0.792474	0	0
497	sp|P14211	CALR_MOU	48 kDa	0.999571	5	100%	P14211	Calr	Calreticulin	23.05235	23.38093	23.94199	23.44173	23.01304	23.57657	-0.13062	-0.94454	0.683296	23.4011	-0.38199	-6.82919	0.71416	0.795728	0	0
214	sp|Q80X90	FLNB_MOU	278 kDa	0.999039	10	90%	Q80X90	Flnb	Filamin-B	20.94778	21.06141	20.71561	20.61266	20.79619	20.45672	0.109596	-0.57533	0.794524	20.76506	0.380846	-6.82967	0.714966	0.795971	0	0
420	sp|P97868	RBBP6_MO	200 kDa	0.99947	6	100%	P97868	Rbbp6	E3 ubiquiti	17.20791	16.80985	17.42868	18.31085	18.05094	18.10283	-0.17866	-1.32144	0.96411	17.65184	-0.3731	-6.8328	0.7206	0.801587	0	0
205	sp|Q78PY7	SND1_MOU	102 kDa	0.99934	10	100%	Q78PY7	Snd1	Staphyloco	22.07838	22.82223	23.10942	22.07828	22.07989	22.82135	-0.15139	-1.12583	0.823043	22.49826	-0.37077	-6.83377	0.722256	0.80277	0	0
986	sp|Q9D2N4	DTNA_MOU	84 kDa	0.997055	3	100%	Q9D2N4	Dtna	Dystrobrev	18.22444	18.24875	19.0052	18.84882	18.24069	17.96129	0.137156	-0.75513	1.029442	18.42153	0.366442	-6.83559	0.725282	0.805473	0	0
557	sp|P70699	LYAG_MOU	106 kDa	0.998851	5	100%	P70699	Gaa	Lysosomal a	21.47067	21.69806	21.40604	20.95197	21.27295	21.85822	-0.11953	-0.90422	0.665165	21.44299	-0.36256	-6.83724	0.727971	0.807797	0	0
1194	sp|P50518	VATE1_MO	26 kDa	0.998024	2	100%	P50518	Atp6v1e1	V-type prot	20.84811	19.15862	20.85129	23.03542	20.80973	21.2867	-0.32387	-2.4718	1.824067	20.99831	-0.35983	-6.83823	0.730051	0.809307	0	0
431	sp|P59279	RAB2B_MO	24 kDa	0.999314	6	17%	P59279	Rab2b	Ras-related	18.13569	21.64292	18.60653	16.37499	18.13099	18.1757	-0.44014	-3.36456	2.484285	18.51114	-0.35917	-6.8385	0.730524	0.809307	0	0
1045	sp|O35609	SCAM3_MO	38 kDa	0.999387	2	100%	O35609	Scamp3	Secretory c	15.58442	16.91987	16.98412	13.91799	15.90329	16.71963	0.304782	-1.73069	2.34025	16.00489	0.357336	-6.83923	0.731834	0.809885	0	0
875	sp|Q9EQH2	ERAP1_MO	107 kDa	0.998959	3	100%	Q9EQH2	Erap1	Endoplasm	19.74412	18.17481	18.71048	20.06963	19.7232	19.33663	0.198907	-1.1316	1.529412	19.29315	0.356766	-6.83946	0.732241	0.809885	0	0
1243	sp|Q5RJ54	ZSC26_MO	54 kDa	0.997096	2	100%	Q5RJ54	Zscan26	Zinc finger a	18.91613	19.3333	19.48355	19.29854	18.76011	18.86824	-0.11343	-0.87504	0.648183	19.10998	-0.35448	-6.84047	0.733745	0.810887	0	0
956	sp|P62835	RAP1A_MO	21 kDa	0.997817	3	33%	P62835	Rap1a	Ras-related	19.19618	19.98074	18.56772	17.75969	18.35197	18.99809	-0.20755	-1.60825	1.193145	18.80906	-0.35362	-6.8407	0.734493	0.811052	0	0
1092	sp|P47754	CAZA2_MO	33 kDa	0.999018	2	100%	P47754	Capza2	F-actin-cap	20.14143	19.07365	19.61129	20.4151	19.4512	19.25248	0.154229	-0.88928	1.197737	19.65753	0.352712	-6.84106	0.735142	0.811109	0	0
946	sp|O88291	ZN326_MO	65 kDa	0.997936	3	100%	O88291	Znf326	DBIRD com	17.16737	16.93323	17.25425	17.47573	18.24135	17.82308	0.143644	-0.84174	1.129031	17.4825	0.347883	-6.84295	0.738604	0.814266	0	0
639	sp|P50247	SAHH_MOU	48 kDa	0.999404	4	100%	P50247	Ahcy	Adenosylho	22.11378	21.10536	22.00446	21.51085	21.09017	23.20427	-0.20402	-1.65421	1.246164	21.83815	-0.33574	-6.84759	0.747335	0.823222	0	0
857	sp|P83870	PHF5A_MO	12 kDa	0.999075	3	100%	P83870	Phf5a	PHD finger-	22.11358	22.3509	22.38025	21.79036	22.19039	22.25601	0.095651	-0.58634	0.777642	22.18025	0.33382	-6.84841	0.748599	0.823945	0	0
1160	sp|Q91WD	NDUS2_MO	53 kDa	0.998328	2	100%	Q91WD5	Ndufs2	NADH dehy	17.99742	17.66516	18.6592	19.17022	17.58113	17.89728	-0.16497	-1.35618	1.026234	18.16173	-0.3305	-6.84955	0.751119	0.826048	0	0
1231	sp|Q9Z321	TOP3B_MO	97 kDa	0.997513	2	100%	Q9Z321	Top3b	DNA topois	20.15416	20.45004	20.52617	20.19876	19.85661	20.17994	-0.09726	-0.8005	0.60598	20.22761	-0.32918	-6.85013	0.751948	0.826289	0	0
1129	sp|Q9R0P5	DEST_MOU	19 kDa	0.998633	2	100%	Q9R0P5	Dstn	Destrin	21.1694	20.56723	20.72556	20.27905	21.07219	21.70453	0.13878	-0.88359	1.161147	20.91966	0.323946	-6.85195	0.75586	0.829803	0	0
959	sp|Q6S5C2	GNPTG_MO	34 kDa	0.99775	3	100%	Q6S5C2	Gnptg	N-acetylglu	21.66111	20.92573	21.93925	22.57333	21.90516	22.47586	-0.15646	-1.3116	0.998675	21.91341	-0.32324	-6.8522	0.756369	0.829803	0	0
889	sp|Q921X9	PDIA5_MO	59 kDa	0.998796	3	100%	Q921X9	Pdia5	Protein dis	17.52168	17.37297	14.89525	17.32488	18.33935	16.87286	-0.27148	-2.29558	1.752624	17.0545	-0.32008	-6.85335	0.758664	0.831648	0	0
369	sp|P35700	PRDX1_MO	22 kDa	0.999	7	86%	P35700	Prdx1	Peroxiredo	24.56835	24.25957	24.40672	24.52826	24.31127	24.24868	0.083278	-0.54172	0.708274	24.38714	0.31714	-6.85448	0.760676	0.83318	0	0
122	sp|Q9ERD7	TBB3_MOU	50 kDa	0.99869	14	14%	Q9ERD7	Tubb3	Tubulin bet	20.68687	20.29832	19.98722	20.4519	20.1946	20.39613	-0.09255	-0.79572	0.610618	20.33584	-0.31327	-6.85585	0.763487	0.835584	0	0
130	sp|P08113	ENPL_MOU	92 kDa	0.999636	13	85%	P08113	Hsp90b1	Endoplasm	23.36985	23.98179	24.60512	24.07457	23.4087	23.70587	-0.12618	-1.09585	0.843482	23.85765	-0.31055	-6.85671	0.765584	0.836705	0	0
1240	sp|Q9DC28	KC1D_MOU	47 kDa	0.997228	2	100%	Q9DC28	Csnk1d	Casein kina	19.15325	19.769	19.64491	17.68718	19.50703	20.23264	0.205455	-1.37451	1.785418	19.33234	0.310327	-6.85679	0.765745	0.836705	0	0
1012	sp|Q8R317	UBQL1_MO	62 kDa	0.994329	3	33%	Q8R317	Ubqln1	Ubiquilin-1	25.34156	20.75504	16.76076	21.16008	18.56516	20.62432	-0.62399	-5.48539	4.237417	20.53449	-0.30631	-6.85818	0.768668	0.839222	0	0
803	tr|A0A075	A0A075B5I	11 kDa	0.9995	3	33%	A0A075B5I	Igkv3-7	Immunoglo	18.92192	18.35212	20.93875	19.58157	19.46144	20.71307	0.225121	-1.55147	2.001712	19.66148	0.302398	-6.85952	0.771523	0.841661	0	0
830	sp|P58021	TM9S2_MO	75 kDa	0.99935	3	100%	P58021	Tm9sf2	Transmemb	22.90288	23.10182	22.57837	22.57899	22.64582	22.7035	-0.08575	-0.76369	0.592195	22.7519	-0.30105	-6.86005	0.772397	0.841938	0	0
268	sp|Q8BFR5	EFTU_MOU	50 kDa	0.999599	8	100%	Q8BFR5	Tufm	Elongation	22.86184	22.51441	22.94794	22.51967	22.49548	23.62095	-0.11659	-1.04688	0.813707	22.82671	-0.29908	-6.86064	0.773948	0.84295	0	0
1215	sp|P97434	MPRIP_MO	116 kDa	0.997743	2	100%	P97434	Mprip	Myosin pho	16.82105	15.70851	14.69956	16.22087	15.9545	16.0343	-0.16285	-1.46885	1.143143	15.90646	-0.29758	-6.86114	0.77504	0.843462	0	0
1107	sp|Q9ESX4	NO40_MOU	27 kDa	0.998887	2	100%	Q9ESX4	Zcchc17	Nucleolar p	21.05791	20.94184	20.89246	21.95751	21.29829	20.69501	-0.11524	-1.06091	0.830434	21.1405	-0.2908	-6.86338	0.779999	0.847658	0	0
555	sp|Q9QXK3	COPG2_MO	98 kDa	0.998882	5	60%	Q9QXK3	Copg2	Coatomer s	19.67779	19.60972	19.09376	18.19907	19.55987	21.14586	-0.20774	-1.91371	1.498231	19.54768	-0.2906	-6.86345	0.780145	0.847658	0	0
522	sp|Q9D662	SC23B_MO	86 kDa	0.999327	5	100%	Q9D662	Sec23b	Protein tra	20.96271	21.19347	20.92166	20.48468	20.53657	21.01095	-0.08939	-0.83523	0.656451	20.85167	-0.28526	-6.86525	0.783958	0.850835	0	0
527	sp|P60122	RUVB1_MO	50 kDa	0.999281	5	100%	P60122	Ruvbl1	RuvB-like 1	21.3495	21.68897	21.58598	21.03236	21.17249	21.6511	-0.08815	-0.82516	0.648856	21.4134	-0.28468	-6.86543	0.784385	0.850835	0	0
1082	sp|P27601	GNA13_MO	44 kDa	0.999114	2	100%	P27601	Gna13	Guanine nu	21.78672	21.72878	21.39984	21.17224	21.90926	21.91978	0.091671	-0.67685	0.860191	21.65277	0.283909	-6.86568	0.784952	0.850835	0	0
365	sp|P80318	TCPG_MOU	61 kDa	0.999039	7	100%	P80318	Cct3	T-complex	22.33462	22.96928	22.87415	23.08905	24.36377	23.99901	-0.1616	-1.55849	1.235299	23.27165	-0.27607	-6.86808	0.790817	0.856508	0	0
246	sp|P17710	HXK1_MOU	108 kDa	0.99889	9	100%	P17710	Hk1	Hexokinase	21.72752	22.48938	22.72211	22.1523	22.16765	22.25295	-0.09245	-0.90109	0.716193	22.25198	-0.27212	-6.86936	0.793631	0.858785	0	0
496	sp|Q8BX02	KANK2_MO	90 kDa	0.999593	5	100%	Q8BX02	Kank2	KN motif ar	20.18583	20.02936	19.595	20.42098	20.04671	19.64596	-0.08958	-0.87533	0.696164	19.98731	-0.27136	-6.86959	0.794186	0.858785	0	0
61	sp|Q8CIE6	COPA_MOU	138 kDa	0.999781	18	100%	Q8CIE6	Copa	Coatomer s	24.14364	23.87813	24.11844	24.39352	24.21512	23.9872	0.07278	-0.58208	0.727642	24.12267	0.264523	-6.87165	0.799235	0.863556	0	0
31	sp|P48678	LMNA_MOU	74 kDa	0.999722	25	96%	P48678	Lmna	Prelamin-A	25.54546	25.69273	25.57772	25.66907	25.52795	25.4869	-0.06586	-0.66479	0.533071	25.5833	-0.26172	-6.87248	0.801305	0.864492	0	0
120	sp|P58252	EF2_MOUS	95 kDa	0.999506	14	93%	P58252	Eef2	Elongation	23.5185	23.43513	23.48735	23.49313	23.23377	23.51272	-0.06712	-0.67774	0.543501	23.44677	-0.26163	-6.87251	0.801376	0.864492	0	0
1232	sp|Q9JKN1	ZNT7_MOU	42 kDa	0.997459	2	100%	Q9JKN1	Slc30a7	Zinc transp	19.52019	20.74202	20.70684	20.88422	20.84311	18.95848	0.16181	-1.33666	1.66028	20.27581	0.257697	-6.8736	0.804379	0.867041	0	0
618	sp|Q7TQI3	OTUB1_MO	31 kDa	0.999546	4	100%	Q7TQI3	Otub1	Ubiquitin t	19.81449	20.59816	21.02587	20.40909	20.44303	20.55783	-0.0939	-0.96887	0.781084	20.47475	-0.25582	-6.87416	0.805728	0.867524	0	0
1154.1	sp|Q9CQS8	SC61B_MO	10 kDa	0.998394	2	100%	Q9CQS8	Sec61b	Protein tra	18.18731	20.39654	17.13762	18.7577	22.24006	19.39209	-0.32711	-3.38408	2.72985	19.35189	-0.25536	-6.87427	0.806107	0.867524	0	0
764	sp|P33173	KIF1A_MOU	192 kDa	0.997228	4	100%	P33173	Kif1a	Kinesin-like	20.97796	21.15958	21.30214	20.87773	21.2574	21.29183	0.069455	-0.58482	0.72373	21.14444	0.252664	-6.8751	0.808014	0.868887	0	0
899.1	sp|P27048	RSMB_MOU	24 kDa	0.998705	3	100%	P27048	Snrpb	Small nucle	21.20571	21.45506	21.00193	21.21995	21.09475	20.8366	-0.06974	-0.75092	0.611446	21.13567	-0.24368	-6.87761	0.814688	0.874768	0	0
805	sp|P08249	MDHM_MO	36 kDa	0.999491	3	100%	P08249	Mdh2	Malate deh	19.93401	19.91285	20.47652	20.18746	19.62211	19.69039	0.080649	-0.70747	0.868768	19.97055	0.243562	-6.87764	0.814773	0.874768	0	0
366	sp|Q9DC48	PRP17_MO	65 kDa	0.999017	7	100%	Q9DC48	Cdc40	Pre-mRNA-	20.73486	19.61313	19.34994	20.16581	21.23264	21.10303	0.145156	-1.29068	1.580995	20.36657	0.241257	-6.87822	0.816574	0.876009	0	0
435	sp|Q8CFQ3	AQR_MOUS	170 kDa	0.999277	6	100%	Q8CFQ3	Aqr	RNA helicas	20.99036	20.89076	20.84026	21.96435	21.60283	20.87386	-0.0985	-1.08188	0.884874	21.19373	-0.23904	-6.87882	0.818221	0.876744	0	0
52	sp|A2AQ25	SKT_MOUS	213 kDa	0.999096	20	100%	A2AQ25	Skt	Sickle tail p	23.56177	23.07745	21.95647	22.7819	24.79154	25.0798	-0.20979	-2.30809	1.888513	23.54149	-0.2386	-6.87894	0.818553	0.876744	0	0
175	sp|P02089	HBB2_MOU	16 kDa	0.999276	11	45%	P02089	Hbb-b2	Hemoglobi	26.2066	26.5462	26.29839	25.89583	26.11172	26.38165	-0.06899	-0.76249	0.624503	26.24007	-0.23679	-6.87948	0.819813	0.8774	0	0
757	sp|Q8BL97	SRSF7_MO	31 kDa	0.997544	4	100%	Q8BL97	Srsf7	Serine/argi	20.2282	20.0828	20.98585	20.85514	20.07208	20.08307	0.088374	-0.82505	1.001801	20.38452	0.230704	-6.88105	0.824408	0.880985	0	0
1183	tr|A0A0B4	A0A0B4J1J	13 kDa	0.998116	2	50%	A0A0B4J1J	Igkv5-43	Immunoglo	19.94503	19.28247	20.14324	20.41841	20.11446	20.24619	0.085223	-0.79573	0.966176	20.02497	0.230621	-6.88107	0.824461	0.880985	0	0
298	sp|Q9JKR6	HYOU1_MO	111 kDa	0.998595	8	100%	Q9JKR6	Hyou1	Hypoxia up	22.10976	22.8192	22.38509	21.87861	22.07534	22.10396	-0.07719	-0.88006	0.725676	22.22866	-0.22884	-6.88156	0.825741	0.881657	0	0
949	sp|Q6ZPZ3	ZC3H4_MO	141 kDa	0.997914	3	100%	Q6ZPZ3	Zc3h4	Zinc finger	18.39262	19.21084	19.6217	18.60171	18.30569	18.80158	-0.09804	-1.12901	0.932924	18.82236	-0.22694	-6.882	0.827239	0.882561	0	0
476	sp|Q91ZW3	SMCA5_MO	122 kDa	0.997308	6	100%	Q91ZW3	Smarca5	SWI/SNF-re	19.72855	19.11031	19.66381	20.07715	19.55415	19.99079	-0.07725	-0.89662	0.742122	19.68746	-0.22441	-6.88269	0.82905	0.883798	0	0
261	sp|Q8BFZ3	ACTBL_MO	42 kDa	0.999727	8	12%	Q8BFZ3	Actbl2	Beta-actin-	25.80617	25.79862	26.06189	25.90643	25.84889	25.8415	0.0568	-0.54988	0.663479	25.87725	0.222838	-6.88309	0.830224	0.884355	0	0
820	sp|Q8BG81	PDIP3_MO	46 kDa	0.9994	3	100%	Q8BG81	Poldip3	Polymerase	22.7588	22.21366	21.68589	22.03713	23.06807	22.95655	0.101808	-0.99634	1.199958	22.45335	0.221243	-6.88345	0.831496	0.885015	0	0
645	sp|O70480	VAMP4_MO	16 kDa	0.999362	4	100%	O70480	Vamp4	Vesicle-ass	21.3392	21.90636	20.96409	21.4667	23.09459	22.42019	-0.13179	-1.56853	1.304944	21.86519	-0.21891	-6.88403	0.833243	0.885492	0	0
540	sp|Q9DC69	NDUA9_MO	43 kDa	0.999153	5	100%	Q9DC69	Ndufa9	NADH dehy	20.95439	20.74596	21.06156	20.8071	20.58324	21.24013	-0.06467	-0.76815	0.638813	20.89873	-0.21879	-6.8841	0.833251	0.885492	0	0
1251.1	sp|Q8C181	MBNL2_MO	40 kDa	0.996815	2	100%	Q8C181	Mbnl2	Muscleblin	19.75808	18.58845	16.69542	17.3453	17.72092	17.73037	0.170101	-1.71509	2.055293	17.97309	0.215329	-6.88491	0.83592	0.887634	0	0
265	sp|Q9D0I9	SYRC_MOU	76 kDa	0.999616	8	100%	Q9D0I9	Rars	Arginine--t	22.86625	22.93493	22.67837	23.16642	23.12159	22.35769	0.069057	-0.70049	0.838606	22.85421	0.213586	-6.88537	0.837149	0.888242	0	0
646	sp|Q61171	PRDX2_MO	22 kDa	0.999353	4	100%	Q61171	Prdx2	Peroxiredo	22.13007	21.76013	22.65564	22.11627	22.02764	22.70884	0.076037	-0.77682	0.928892	22.2331	0.212494	-6.88561	0.838006	0.888457	0	0
50	sp|E9Q4N7	ARI1B_MO	238 kDa	0.999548	20	90%	E9Q4N7	Arid1b	AT-rich inte	22.91238	23.11168	22.11225	23.12616	23.51563	22.5675	-0.08836	-1.09648	0.919763	22.89093	-0.20916	-6.88639	0.840539	0.889935	0	0
693	tr|A0A140	A0A140T8	11 kDa	0.998986	4	50%	A0A140T8	Igkv8-19	Immunoglo	20.85234	22.0368	22.94648	22.69191	20.61774	20.21752	-0.17655	-2.19316	1.840056	21.56047	-0.20893	-6.88644	0.840713	0.889935	0	0
941	sp|Q9D0F3	LMAN1_MO	58 kDa	0.998022	3	100%	Q9D0F3	Lman1	Protein ERC	17.6207	13.26178	18.26963	21.05394	17.06045	19.81639	-0.39378	-4.9364	4.148852	17.84715	-0.20687	-6.88693	0.842261	0.89083	0	0
288	tr|Q8VHM5	Q8VHM5_M	71 kDa	0.999188	8	100%	Q8VHM5	Hnrnpr	Heterogene	23.30095	21.90537	22.48716	23.44388	23.13989	23.92765	-0.1163	-1.46325	1.230652	23.03415	-0.20605	-6.88712	0.842872	0.89083	0	0
1256	sp|Q9D1M	FKB11_MO	22 kDa	0.996662	2	100%	Q9D1M7	Fkbp11	Peptidyl-pr	18.64991	20.05106	19.02298	19.2587	21.36887	20.16775	-0.14525	-1.90022	1.609723	19.75321	-0.19751	-6.88907	0.849288	0.896912	0	0
1074	sp|Q9CXW	RL11_MOU	20 kDa	0.999168	2	100%	Q9CXW4	Rpl11	60S riboso	19.20832	18.50612	20.30768	20.06658	19.34228	20.64254	-0.11899	-1.57933	1.341359	19.67892	-0.19444	-6.88975	0.851595	0.898648	0	0
735	sp|P12815	PDCD6_MO	22 kDa	0.998357	4	100%	P12815	Pdcd6	Programme	22.06108	22.01147	22.57604	22.06772	21.65422	22.02628	0.061958	-0.70127	0.825185	22.06614	0.193216	-6.89005	0.852448	0.898849	0	0
884	sp|Q8BPQ7	SGSM1_MO	123 kDa	0.99885	3	100%	Q8BPQ7	Sgsm1	Small G pro	16.85289	16.09935	14.58429	16.0955	16.90943	16.52604	-0.12476	-1.6751	1.425584	16.17792	-0.19204	-6.89027	0.853405	0.899159	0	0
1039	sp|Q9CZU6	CISY_MOUS	52 kDa	0.999426	2	100%	Q9CZU6	Cs	Citrate synt	18.71935	19.08206	19.36727	18.73393	18.53251	18.61436	0.062927	-0.72662	0.852477	18.84158	0.189696	-6.89081	0.8551	0.900245	0	0
219	sp|E9Q784	ZC3HD_MO	204 kDa	0.997856	10	100%	E9Q784	Zc3h13	Zinc finger	21.42312	21.17777	20.74086	21.73705	22.0924	21.10612	0.078479	-0.91777	1.074729	21.37955	0.18799	-6.89114	0.856453	0.90097	0	0
1157	tr|D3YZZ5	D3YZZ5_M	25 kDa	0.99837	2	100%	D3YZZ5	Tmed7	Transmemb	19.16088	19.91201	20.42441	19.96342	19.02837	19.00143	-0.08773	-1.24737	1.071907	19.58175	-0.18054	-6.8927	0.862066	0.905386	0	0
276	sp|Q5SQX6	CYFP2_MO	146 kDa	0.999473	8	38%	Q5SQX6	Cyfip2	Cytoplasmi	20.79859	21.2019	19.66486	20.00923	20.57171	20.07719	-0.08439	-1.20517	1.036393	20.38725	-0.17968	-6.89287	0.862717	0.905386	0	0
291	sp|P48722	HS74L_MO	94 kDa	0.99902	8	88%	P48722	Hspa4l	Heat shock	22.65517	22.68177	22.58948	22.39452	22.79719	23.12671	-0.05372	-0.76647	0.659036	22.70747	-0.17938	-6.89296	0.862884	0.905386	0	0
746	sp|Q01405	SC23A_MO	86 kDa	0.997866	4	100%	Q01405	Sec23a	Protein tra	20.46179	20.9745	21.20348	21.70314	22.79839	22.13014	-0.1147	-1.64534	1.415938	21.54524	-0.17883	-6.89304	0.863357	0.905386	0	0
554	sp|A2AIV2	VIR_MOUS	201 kDa	0.998885	5	100%	A2AIV2	Virma	Protein viri	18.65207	20.73825	18.01073	16.58382	19.20584	19.08094	-0.17813	-2.56635	2.210094	18.71194	-0.178	-6.89321	0.863989	0.905386	0	0
622	sp|P09411	PGK1_MOU	45 kDa	0.999493	4	100%	P09411	Pgk1	Phosphogly	21.27753	21.32949	21.82823	21.64172	20.9402	20.88428	0.063492	-0.79112	0.918099	21.31691	0.17707	-6.89341	0.864658	0.905387	0	0
1101	sp|Q8VHR5	P66B_MOU	65 kDa	0.998958	2	100%	Q8VHR5	Gatad2b	Transcripti	19.44319	20.15276	20.86102	20.26719	20.01486	20.1095	-0.07013	-1.03132	0.891062	20.14142	-0.17411	-6.89399	0.866922	0.907057	0	0
230	sp|Q3UJB9	EDC4_MOU	152 kDa	0.999697	9	100%	Q3UJB9	Edc4	Enhancer o	23.14227	22.16338	22.08852	22.31154	21.60148	22.14372	0.071208	-0.95458	1.096991	22.24182	0.165662	-6.89561	0.873312	0.913039	0	0
519	sp|F8VPU2	FARP1_MO	119 kDa	0.999348	5	100%	F8VPU2	Farp1	FERM, ARH	21.29879	21.31905	21.6422	20.97503	21.28754	22.11016	-0.05857	-0.93514	0.817999	21.4388	-0.15929	-6.8968	0.878112	0.917338	0	0
545	sp|Q61316	HSP74_MO	94 kDa	0.999066	5	80%	Q61316	Hspa4	Heat shock	21.483	22.08773	22.02101	21.20066	21.08727	21.48338	-0.06016	-0.96553	0.845201	21.56051	-0.15842	-6.89696	0.878777	0.917338	0	0
1123	sp|Q69ZB0	LRCC1_MO	119 kDa	0.998684	2	100%	Q69ZB0	Lrrcc1	Leucine-ric	16.72955	15.2281	17.21865	17.44409	15.52095	16.48037	0.105532	-1.50847	1.719536	16.43695	0.156038	-6.89737	0.880602	0.918535	0	0
107	sp|P68033	ACTC_MOU	42 kDa	0.999399	15	13%	P68033	Actc1	Actin, alph	24.82825	24.95047	24.98502	25.03139	24.82405	24.7731	-0.03921	-0.6497	0.571273	24.89871	-0.15288	-6.89794	0.882938	0.920265	0	0
970	sp|Q07235	GDN_MOU	44 kDa	0.997591	3	100%	Q07235	Serpine2	Glia-derive	19.53715	19.08583	20.09918	19.9864	19.2582	19.65896	0.054449	-0.8297	0.938595	19.60429	0.146812	-6.89896	0.887581	0.924246	0	0
65	tr|E9Q0S6	E9Q0S6_M	201 kDa	0.999567	18	94%	E9Q0S6	Tns1	Tensin 1	21.95895	21.65872	20.96663	21.55416	22.5955	22.10686	0.067109	-1.02887	1.163089	21.8068	0.146127	-6.89907	0.888121	0.924246	0	0
864	sp|Q9DCT2	NDUS3_MO	30 kDa	0.999038	3	100%	Q9DCT2	Ndufs3	NADH dehy	20.36371	20.89951	20.82638	20.18019	20.21143	20.45857	-0.04558	-0.82317	0.732004	20.48996	-0.13952	-6.90016	0.893089	0.926951	0	0
1111	sp|Q9CWJ9	PUR9_MOU	64 kDa	0.998832	2	100%	Q9CWJ9	Atic	Bifunctiona	20.32476	20.59322	20.67541	20.10776	20.16423	20.34214	0.040429	-0.65645	0.737307	20.36792	0.138081	-6.90038	0.894187	0.926951	0	0
798.2	sp|Q91VM5	RMXL1_MO	42 kDa	0.999544	3	100%	Q91VM5	Rbmxl1	RNA bindin	23.32623	22.79697	22.02473	23.00488	23.438	22.81121	0.058631	-0.96831	1.085569	22.90034	0.136249	-6.90065	0.895629	0.926951	0	0
853	sp|D3YVE8	S35G2_MO	46 kDa	0.999109	3	100%	D3YVE8	Slc35g2	Solute carr	21.33574	22.58111	21.10183	20.10264	21.23871	20.73697	0.085185	-1.40964	1.580013	21.18283	0.135995	-6.90069	0.895822	0.926951	0	0
1033.2	sp|Q9JJW6	ALRF2_MO	24 kDa	0.999499	2	100%	Q9JJW6	Alyref2	Aly/REF exp	20.45353	20.23386	20.84213	19.7014	20.15506	21.32573	0.063242	-1.05543	1.181916	20.45195	0.134913	-6.90086	0.896645	0.926951	0	0
550	sp|P47856	GFPT1_MO	79 kDa	0.99894	5	100%	P47856	Gfpt1	Glutamine-	19.69151	20.05103	20.15577	19.7354	19.58693	19.76446	-0.03889	-0.73163	0.653838	19.83085	-0.13363	-6.90107	0.89757	0.926951	0	0
916.9	sp|C0HKE9	H2A1P_MO	14 kDa	0.998436	3	33%	C0HKE9	Hist1h2ap	Histone H2	20.40214	18.79705	18.81104	21.67383	19.56569	18.65782	-0.11661	-2.20082	1.967598	19.65126	-0.13352	-6.90107	0.897704	0.926951	0	0
492	sp|P26369	U2AF2_MO	54 kDa	0.999676	5	100%	P26369	U2af2	Splicing fac	21.56485	22.2022	21.52638	21.4041	22.07582	21.68901	-0.04275	-0.83702	0.751513	21.74373	-0.12811	-6.90189	0.901778	0.926951	0	0
375	sp|Q91VR2	ATPG_MOU	33 kDa	0.998696	7	100%	Q91VR2	Atp5f1c	ATP syntha	24.74527	24.59051	24.32571	25.39775	24.57984	23.8282	-0.05521	-1.09854	0.988111	24.57788	-0.12629	-6.90213	0.903206	0.926951	0	0
841	sp|Q99020	ROAA_MOU	31 kDa	0.999237	3	67%	Q99020	Hnrnpab	Heterogene	21.44213	22.40325	19.3087	19.55064	22.88734	22.09729	-0.13767	-2.74438	2.46904	21.28156	-0.12604	-6.90217	0.903401	0.926951	0	0
867.2	tr|A0A140	A0A140T8	13 kDa	0.999017	3	100%	A0A140T8	Igkv6-20	Immunoglo	20.59284	20.42443	20.78981	20.59627	20.6225	20.88238	0.034023	-0.61099	0.67904	20.65137	0.125546	-6.90226	0.903732	0.926951	0	0
950	sp|Q8C052	MAP1S_MO	103 kDa	0.997908	3	100%	Q8C052	Map1s	Microtubul	20.56109	20.87354	20.92946	20.85954	21.03078	20.6912	0.032355	-0.61833	0.683041	20.82427	0.118349	-6.90325	0.90922	0.926951	0	0
601	sp|P17225	PTBP1_MO	56 kDa	0.999706	4	100%	P17225	Ptbp1	Polypyrimi	22.61313	21.57616	21.20093	21.83852	22.70467	22.90967	0.064793	-1.24326	1.372841	22.14051	0.118209	-6.90326	0.909368	0.926951	0	0

1198	sp|P67984	RL22_MOU	15 kDa	0.997965	2	100%	P67984	Rpl22	60S ribosor	19.9585	20.06135	20.39028	20.94424	21.58985	20.75909	0.057984	-1.12321	1.239183	20.61722	0.117149	-6.9034	0.910177	0.926951	0	0
340	sp|Q8K310	MATR3_MC	95 kDa	0.999425	7	100%	Q8K310	Matr3	Matrin-3	22.1656	21.47324	21.79343	22.14264	22.12795	22.59859	-0.0425	-0.90805	0.82305	22.05024	-0.11705	-6.90342	0.910234	0.926951	0	0
153	sp|Q8CI94	PYGB_MOU	97 kDa	0.999191	12	100%	Q8CI94	Pygb	Glycogen p	23.31514	23.29045	23.23935	23.33649	23.28189	23.29564	-0.02873	-0.61712	0.55965	23.29316	-0.11623	-6.90353	0.910833	0.926951	0	0
1248	sp|O35593	PSDE_MOU	35 kDa	0.99692	2	100%	O35593	Psmd14	26S protea	21.12769	21.27214	21.34948	21.16852	21.04555	21.16986	-0.02927	-0.64192	0.583385	21.18887	-0.11371	-6.90386	0.912763	0.926951	0	0
909	sp|Q9EQH3	VPS35_MO	92 kDa	0.998549	3	100%	Q9EQH3	Vps35	Vacuolar pr	20.51883	20.37394	20.09085	20.03742	19.4143	19.47568	0.045646	-0.9159	1.007193	19.98517	0.113288	-6.9039	0.913123	0.926951	0	0
910	sp|Q64704	STX3_MOU	33 kDa	0.998547	3	100%	Q64704	Stx3	Syntaxin-3	20.51883	20.37394	20.09085	20.03742	19.4143	19.47568	0.045646	-0.9159	1.007193	19.98517	0.113288	-6.9039	0.913123	0.926951	0	0
911.1	sp|P01680	KV4A1_MO	14 kDa	0.998536	3	67%	P01680	NA	Ig kappa ch	20.51883	20.37394	20.09085	20.03742	19.4143	19.47568	0.045646	-0.9159	1.007193	19.98517	0.113288	-6.9039	0.913123	0.926951	0	0
911.2	tr|A0A075I	A0A075B5I	10 kDa	0.998536	3	67%	A0A075B5I	Igkv4-58	Immunoglo	20.51883	20.37394	20.09085	20.03742	19.4143	19.47568	0.045646	-0.9159	1.007193	19.98517	0.113288	-6.9039	0.913123	0.926951	0	0
912	sp|P62082	RS7_MOUS	22 kDa	0.998535	3	100%	P62082	Rps7	40S ribosor	20.51883	20.37394	20.09085	20.03742	19.4143	19.47568	0.045646	-0.9159	1.007193	19.98517	0.113288	-6.9039	0.913123	0.926951	0	0
913	sp|O70194	EIF3D_MOU	64 kDa	0.998488	3	100%	O70194	Eif3d	Eukaryotic	20.51883	20.37394	20.09085	20.03742	19.4143	19.47568	0.045646	-0.9159	1.007193	19.98517	0.113288	-6.9039	0.913123	0.926951	0	0
914	sp|O70401	TSN6_MOU	27 kDa	0.998475	3	100%	O70401	Tspan6	Tetraspanir	20.51883	20.37394	20.09085	20.03742	19.4143	19.47568	0.045646	-0.9159	1.007193	19.98517	0.113288	-6.9039	0.913123	0.926951	0	0
915	sp|Q8C854	MYEF2_MO	63 kDa	0.998453	3	100%	Q8C854	Myef2	Myelin expr	20.51883	20.37394	20.09085	20.03742	19.4143	19.47568	0.045646	-0.9159	1.007193	19.98517	0.113288	-6.9039	0.913123	0.926951	0	0
916.1	sp|C0HKE1	H2A1B_MC	14 kDa	0.998436	3	33%	C0HKE1	Hist1h2ab	Histone H2	20.51883	20.37394	20.09085	20.03742	19.4143	19.47568	0.045646	-0.9159	1.007193	19.98517	0.113288	-6.9039	0.913123	0.926951	0	0
916.2	sp|C0HKE2	H2A1C_MC	14 kDa	0.998436	3	33%	C0HKE2	Hist1h2ac	Histone H2	20.51883	20.37394	20.09085	20.03742	19.4143	19.47568	0.045646	-0.9159	1.007193	19.98517	0.113288	-6.9039	0.913123	0.926951	0	0
916.3	sp|C0HKE3	H2A1D_MC	14 kDa	0.998436	3	33%	C0HKE3	Hist1h2ad	Histone H2	20.51883	20.37394	20.09085	20.03742	19.4143	19.47568	0.045646	-0.9159	1.007193	19.98517	0.113288	-6.9039	0.913123	0.926951	0	0
916.4	sp|C0HKE4	H2A1E_MO	14 kDa	0.998436	3	33%	C0HKE4	Hist1h2ae	Histone H2	20.51883	20.37394	20.09085	20.03742	19.4143	19.47568	0.045646	-0.9159	1.007193	19.98517	0.113288	-6.9039	0.913123	0.926951	0	0
916.5	sp|C0HKE5	H2A1G_MC	14 kDa	0.998436	3	33%	C0HKE5	Hist1h2ag	Histone H2	20.51883	20.37394	20.09085	20.03742	19.4143	19.47568	0.045646	-0.9159	1.007193	19.98517	0.113288	-6.9039	0.913123	0.926951	0	0
916.6	sp|C0HKE6	H2A1I_MO	14 kDa	0.998436	3	33%	C0HKE6	Hist1h2ai	Histone H2	20.51883	20.37394	20.09085	20.03742	19.4143	19.47568	0.045646	-0.9159	1.007193	19.98517	0.113288	-6.9039	0.913123	0.926951	0	0
916.7	sp|C0HKE7	H2A1N_MC	14 kDa	0.998436	3	33%	C0HKE7	Hist1h2an	Histone H2	20.51883	20.37394	20.09085	20.03742	19.4143	19.47568	0.045646	-0.9159	1.007193	19.98517	0.113288	-6.9039	0.913123	0.926951	0	0
155	sp|P17426	AP2A1_MO	108 kDa	0.998576	12	75%	P17426	Ap2a1	AP-2 compl	22.93295	22.47298	22.6362	22.01365	21.94942	22.90079	0.043716	-0.87866	0.966088	22.48433	0.113079	-6.90393	0.913279	0.926951	0	0
1151	sp|Q3ULD5	MCCB_MOU	61 kDa	0.998431	2	100%	Q3ULD5	Mccc2	Methylcrot	21.55498	22.09253	22.86161	22.41022	21.89145	21.93979	-0.04483	-1.00743	0.917765	22.1251	-0.11115	-6.90418	0.914755	0.927755	0	0
1073	sp|Q8BYR5	CAPS2_MO	148 kDa	0.999194	2	100%	Q8BYR5	Cadps2	Calcium-de	17.76841	18.70472	17.65086	17.46606	17.77377	16.86584	0.052138	-1.10626	1.210536	17.70494	0.10741	-6.90464	0.91761	0.929955	0	0
885	sp|Q9D0F9	PGM1_MO	61 kDa	0.998848	3	100%	Q9D0F9	Pgm1	Phosphoglu	18.09085	15.92527	18.3743	19.68268	18.78224	19.33598	0.101155	-2.2151	2.417412	18.36522	0.10422	-6.90502	0.920047	0.931728	0	0
1116	sp|Q7M6Y3	PICAL_MOU	72 kDa	0.998771	2	100%	Q7M6Y3	Picalm	Phosphatic	20.13643	19.17647	21.37808	20.471	19.70109	21.37022	0.065972	-1.53927	1.671217	20.37222	0.098077	-6.90573	0.924741	0.935783	0	0
745	tr|A0A140T	A0A140T8M	13 kDa	0.997869	4	25%	A0A140T8M	Igkv12-44	Immunoglo	19.83415	19.31605	19.20688	18.51155	19.08573	20.16884	0.043438	-1.09724	1.184112	19.35387	0.090877	-6.9065	0.930248	0.940654	0	0
976	sp|P63323	RS12_MOU	15 kDa	0.997401	3	100%	P63323	Rps12	40S ribosor	19.49148	19.14987	16.10126	18.24643	19.09579	17.5199	-0.07589	-2.31001	2.158234	18.26745	-0.08106	-6.90746	0.937762	0.947544	0	0
251	sp|Q7TMB8	CYFP1_MO	145 kDa	0.998228	9	44%	Q7TMB8	Cyfip1	Cytoplasmi	21.39762	20.75995	19.23383	20.25249	20.90249	20.3841	0.045797	-1.3236	1.415198	20.48841	0.079811	-6.90758	0.93872	0.947729	0	0
223	sp|Q8R1B4	EIF3C_MOU	106 kDa	0.99983	9	100%	Q8R1B4	Eif3c	Eukaryotic	21.64697	21.43479	22.32186	21.69111	21.79317	22.73647	-0.03346	-1.04412	0.97721	21.9374	-0.079	-6.90765	0.939342	0.947729	0	0
1186	tr|A0A075I	A0A075B5V	11 kDa	0.998102	2	50%	A0A075B5V	Ighv8-6	Immunoglo	19.61395	20.03651	18.98044	18.48211	20.13247	20.3272	-0.03966	-1.39342	1.314111	19.59545	-0.06991	-6.90842	0.946309	0.952636	0	0
39	sp|P11679	K2C8_MOU	55 kDa	0.999223	23	78%	P11679	Krt8	Keratin, typ	24.76468	24.61796	24.25378	24.79579	25.13428	24.80522	-0.02208	-0.7797	0.735551	24.72862	-0.06935	-6.90847	0.946711	0.952636	0	0
446	sp|P62301	RS13_MOU	17 kDa	0.999096	6	100%	P62301	Rps13	40S ribosor	23.85364	23.37366	24.21973	23.1666	23.2309	24.87424	-0.03674	-1.30458	1.231096	23.78646	-0.06916	-6.90848	0.94688	0.952636	0	0
139	sp|Q6PIC6	AT1A3_MO	112 kDa	0.999076	13	31%	Q6PIC6	Atp1a3	Sodium/po	20.23796	20.39139	21.50874	21.785	20.56224	20.24407	-0.03717	-1.32307	1.248727	20.78823	-0.06898	-6.90849	0.947016	0.952636	0	0
502	sp|O08784	TCOF_MOU	135 kDa	0.999531	5	100%	O08784	Tcof1	Treacle pro	20.91253	20.42715	21.2026	22.96105	23.16259	21.7215	0.056006	-1.90819	2.020203	21.73123	0.068046	-6.90857	0.947736	0.952654	0	0
461	sp|Q9DC16	ERGI1_MOU	33 kDa	0.99886	6	100%	Q9DC16	Ergic1	Endoplasm	21.9127	21.74401	22.29723	21.89614	21.43066	22.0419	-0.01382	-0.77446	0.746813	21.88711	-0.04325	-6.91014	0.966747	0.970325	0	0
564	sp|P30415	NKTR_MOU	163 kDa	0.998614	5	100%	P30415	Nktr	NK-tumor r	18.89173	18.71571	18.58952	19.10442	19.91397	19.63225	-0.01905	-1.07998	1.041882	19.14127	-0.04285	-6.91016	0.967073	0.970325	0	0
4	sp|Q8VDD5	MYH9_MOU	226 kDa	0.999708	54	78%	Q8VDD5	Myh9	Myosin-9	25.63779	25.28698	25.19296	25.61598	25.52883	25.49176	-0.01171	-0.67088	0.647447	25.45905	-0.0423	-6.91018	0.967477	0.970325	0	0
1192	sp|Q921G7	ETFD_MOU	68 kDa	0.998029	2	100%	Q921G7	Etfdh	Electron tra	16.08342	16.00759	16.94603	16.22071	16.29947	17.15236	-0.01725	-1.01128	0.976783	16.4516	-0.04141	-6.91022	0.968178	0.970325	0	0
1182	sp|P12656	TSHB_MOU	15 kDa	0.998122	2	100%	P12656	Tshb	Thyrotropi	18.9127	18.78192	19.13725	18.57023	18.65812	19.32253	0.01113	-0.74749	0.769754	18.89713	0.034919	-6.91051	0.97315	0.974588	0	0
925	sp|P40142	TKT_MOUS	68 kDa	0.998322	3	100%	P40142	Tkt	Transketola	20.65241	20.75414	20.7934	20.09826	19.95131	20.52276	0.00732	-0.82558	0.840215	20.46204	0.020918	-6.91095	0.983913	0.98464	0	0
1260	sp|Q9D023	MPC2_MOU	14 kDa	0.99645	2	100%	Q9D023	Mpc2	Mitochond	20.59037	20.82323	21.61562	22.10251	21.26301	20.56059	-0.00578	-1.19682	1.185272	21.15922	-0.01157	-6.91112	0.991103	0.991103	0	0
1130	sp|P60766	CDC42_MC	21 kDa	0.998611	2	100%	P60766	Cdc42	Cell divisio	NA	NA	NA	NA	19.23984	NA	NA	NA	NA	19.23984	NA	NA	NA	NA	NA	NA
1228	sp|P11031	TCP4_MOU	14 kDa	0.99755	2	100%	P11031	Sub1	Activated R	14.74753	NA	NA	NA	13.88403	NA	NA	NA	NA	14.31578	NA	NA	NA	NA	NA	NA
1258	sp|P48774	GSTM5_MC	27 kDa	0.996561	2	50%	P48774	Gstm5	Glutathion	17.06121	NA	17.08049	NA	16.39204	NA	NA	NA	NA	16.84458	NA	NA	NA	NA	NA	NA
1292	sp|Q9WUA	PFKAP_MO	85 kDa	0.994644	2	100%	Q9WUA3	Pfkp	ATP-dependent 6-phosphofructokinase, platelet type																
1293	sp|Q99JX7	NXF1_MOU	70 kDa	0.994523	2	100%	Q99JX7	Nxf1	Nuclear RNA export factor 1																
1294.1	sp|P83741	WNK1_MO	251 kDa	0.994185	2	50%	P83741	Wnk1	Serine/threonine-protein kinase WNK1																
1294.2	sp|Q80UE6	WNK4_MO	132 kDa	0.994185	2	50%	Q80UE6	Wnk4	Serine/threonine-protein kinase WNK4																
1295.1	sp|Q497I4	KRT35_MO	51 kDa	0.99394	2	50%	Q497I4	Krt35	Keratin, type I cuticular Ha5																
1295.2	sp|Q61765	K1H1_MOU	47 kDa	0.99394	2	50%	Q61765	Krt31	Keratin, type I cuticular Ha1																
1295.3	sp|Q61897	KT33B_MO	46 kDa	0.99394	2	50%	Q61897	Krt33b	Keratin, type I cuticular Ha3-II																
1296	tr|Q68FG2	Q68FG2_M	271 kDa	0.993774	2	50%	Q68FG2	Sptbn2	Spectrin beta chain																
1297	sp|Q8CI43	MYL6B_MC	23 kDa	0.993631	2	50%	Q8CI43	Myl6b	Myosin light chain 6B																
1298	sp|Q05793	PGBM_MO	398 kDa	0.993589	2	100%	Q05793	Hspg2	Basement membrane-specific heparan sulfate proteoglycan core protein																
1299	sp|Q8C006	TRI35_MOU	57 kDa	0.993579	2	100%	Q8C006	Trim35	Tripartite motif-containing protein 35																
1300	sp|Q3TWW	SRSF6_MOU	39 kDa	0.993577	2	50%	Q3TWW8	Srsf6	Serine/arginine-rich splicing factor 6																
1301	sp|A1L317	K1C24_MO	54 kDa	0.993541	2	50%	A1L317	Krt24	Keratin, type I cytoskeletal 24																
1302	sp|Q9JMH6	TRXR1_MO	67 kDa	0.993513	2	100%	Q9JMH6	Txnrd1	Thioredoxin reductase 1, cytoplasmic																
1303	sp|Q91W4	KLC3_MOU	56 kDa	0.993453	2	100%	Q91W40	Klc3	Kinesin light chain 3																
1304.1	sp|P63271	SPT4A_MO	13 kDa	0.993287	2	100%	P63271	Supt4h1a	Transcription elongation factor SPT4-A																
1304.2	sp|Q9Z199	SPT4B_MO	13 kDa	0.993287	2	100%	Q9Z199	Supt4h1b	Transcription elongation factor SPT4-B																
1305	sp|Q5HZG4	TAF3_MOU	105 kDa	0.993207	2	100%	Q5HZG4	Taf3	Transcription initiation factor TFIID subunit 3																
1306	sp|Q9QUM	PSA6_MOU	27 kDa	0.99316	2	100%	Q9QUM9	Psma6	Proteasome subunit alpha type-6																

Supplementary Table S2

Protein Annotation										Log2 Cyclic Loess Normalized Intensities						Musashi_vs_IgG									
id	rotein Nam	ession Num	lecular Wei	ein Group S	ied Peptide	Exclusivity	UniProt ID	Gene name	Description	E1_MSI12	E1_IgG	E2_MSI12	E2_IgG	E3_MSI12	E3_IgG	logFC	CI.L	CI.R	AveExpr	t	B	P.Value	adj.P.Val	sig.PVal	sig.FDR
677	sp|Q80WC	UBN2_MOU	142 kDa	0.999085	4	1	Q80WC1	Ubn2	Ubinuclein-	19.17213	13.26706	17.38307	5.625675	21.08303	14.01973	8.241925	2.886949	13.5969	15.09178	3.673007	-2.73929	0.008574	0.036911	1	1
778	sp|P01811	HVM41_MC	13 kDa	0.995278	4	0.25	P01811	NA	Ig heavy ch	21.20257	14.84614	21.92793	15.22002	20.71727	15.79102	5.996865	4.988373	7.005358	18.28416	14.19065	5.325797	3.03E-06	0.000427	1	1
305	sp|Q60749	KHDR1_MC	48 kDa	0.999885	7	0.86	Q60749	Khdrbs1	KH domain	26.58346	21.36758	25.47485	20.85161	27.02601	20.11857	5.582188	4.353376	6.811	23.57035	10.84101	3.721701	1.72E-05	0.001058	1	1
1061	sp|O09108	LSHB_MOU	15 kDa	0.999248	2	1	O09108	Lhb	Lutropin su	19.02823	NA	18.45906	13.25611	19.67979	13.71208	5.571598	4.430488	6.712707	16.82705	12.10273	3.290413	2.8E-05	0.001518	1	1
1015.2	tr|E9Q035	E9Q035_M	108 kDa	0.99986	2	1	E9Q035	Gm20425	Predicted g	24.19942	18.47307	24.71601	18.60751	23.98793	19.52478	5.432666	4.499976	6.365356	21.58479	13.90037	5.207377	3.46E-06	0.000427	1	1
611	sp|Q9CYZ8	SSBP2_MO	38 kDa	0.999605	4	0.75	Q9CYZ8	Ssbp2	Single-stran	23.22381	17.48517	23.58941	19.2692	22.92989	18.70953	4.759738	3.573528	5.945949	20.86784	9.575731	2.949857	3.77E-05	0.001704	1	1
1	sp|Q68FD5	CLH1_MOU	192 kDa	0.999927	86	1	Q68FD5	Cltc	Clathrin hea	33.45827	28.44516	32.44402	27.97304	33.37165	28.92852	4.642404	3.66433	5.620478	30.77011	11.32718	3.990771	1.3E-05	0.000976	1	1
89	sp|P60879	SNP25_MO	23 kDa	0.999799	16	0.94	P60879	Snap25	Synaptosor	28.13807	22.63727	27.60588	23.42141	27.93553	23.73367	4.629044	3.744928	5.513159	25.57864	12.48178	4.611365	6.68E-06	0.000697	1	1
346	sp|P70662	LDB1_MOU	47 kDa	0.999359	7	0.86	P70662	Ldb1	LIM domain	26.67237	21.65568	26.89319	21.90304	26.29582	22.43223	4.62348	3.83559	5.41137	24.30872	13.96704	5.331501	3.01E-06	0.000427	1	1
150	sp|Q6IRU5	CLCB_MOU	25 kDa	0.999464	12	0.92	Q6IRU5	Cltb	Clathrin ligh	28.38085	23.80329	28.405	24.14557	28.20823	23.45666	4.529515	3.830334	5.228696	26.0666	15.41923	5.895842	1.57E-06	0.000425	1	1
465	sp|O08585	CLCA_MOU	26 kDa	0.998487	6	0.83	O08585	Clta	Clathrin ligh	26.99857	21.91196	26.85232	23.2787	26.83168	22.39684	4.365024	3.419942	5.310106	24.71168	11.02218	3.823632	1.54E-05	0.001047	1	1
1006	sp|Q8R1F6	HID1_MOU	89 kDa	0.995228	3	1	Q8R1F6	Hid1	Protein HID	21.51472	18.28828	21.00575	17.14254	22.13313	16.37584	4.282311	2.959029	5.605593	19.41004	7.722831	1.594312	0.000143	0.004042	1	1
51	sp|Q8BZ98	DYN3_MOU	97 kDa	0.999347	20	0.65	Q8BZ98	Dnm3	Dynamin-3	25.00528	21.075	23.35635	19.66307	25.03548	20.22352	4.145173	2.745449	5.544897	22.39312	7.067259	1.03641	0.000245	0.004931	1	1
131	sp|Q61474	MSI1H_MO	39 kDa	0.999636	13	0.92	Q61474	Msi1	RNA-bindin	28.00456	24.28889	27.83918	23.26068	27.77549	23.72374	4.115307	3.306103	4.924512	25.81542	12.10503	4.477346	7.7E-06	0.000746	1	1
25	sp|O35245	PKD2_MOU	109 kDa	0.999878	26	1	O35245	Pkd2	Polycystin-	26.92774	23.18959	26.6901	22.84655	26.98119	22.60046	3.98748	3.303276	4.671684	24.8726	13.87119	5.291385	3.15E-06	0.000427	1	1
45	sp|P28740	KIF2A_MOU	80 kDa	0.999855	21	1	P28740	Kif2a	Kinesin-like	27.45602	23.07958	26.23326	23.07752	27.59822	23.29447	3.94531	2.947576	4.943043	25.12318	9.436634	2.85807	4.13E-05	0.001752	1	1
35	sp|Q6NZC7	S23IP_MOU	111 kDa	0.999696	23	1	Q6NZC7	Sec23ip	SEC23-inte	28.70469	24.29238	27.76828	24.26505	28.49033	24.75437	3.883829	3.049447	4.718211	26.37918	11.07943	3.933021	1.37E-05	0.000976	1	1
164	sp|Q920Q6	MSI2H_MO	37 kDa	0.999498	11	0.91	Q920Q6	Msi2	RNA-bindin	30.20478	26.58534	29.58572	24.87125	30.10317	27.15205	3.761676	2.325269	5.198084	28.08372	6.249632	0.272013	0.000506	0.006933	1	1
202	sp|Q61235	SNTB2_MO	56 kDa	0.99945	10	0.9	Q61235	Sntb2	Beta-2-synt	23.90047	20.00991	23.21943	18.6166	23.04242	20.27871	3.752367	2.538014	4.966719	21.51126	7.374136	1.303293	0.000189	0.004586	1	1
415	sp|P42669	PURA_MOU	35 kDa	0.999562	6	1	P42669	Pura	Transcriptio	27.2554	23.15779	26.17552	23.10798	27.42142	23.38845	3.732709	2.792645	4.672772	25.08443	9.475831	2.884084	4.03E-05	0.001752	1	1
329	sp|P06879	PRL_MOUS	25 kDa	0.999529	7	1	P06879	Prl	Prolactin	30.24519	26.1948	30.41172	26.66332	29.35326	26.185	3.655683	2.766844	4.544523	28.17555	9.804813	3.125505	3.15E-05	0.001631	1	1
788	sp|P16125	LDHB_MOU	37 kDa	0.999654	3	0.67	P16125	Ldhb	L-lactate de	21.37743	17.29409	19.77552	17.52084	22.76833	18.14281	3.654516	1.893459	5.415572	19.47984	4.952303	-1.12114	0.001877	0.014463	1	1
185	sp|Q7TSH6	SCAF4_MO	129 kDa	0.999718	10	0.7	Q7TSH6	Scaf4	SR-related a	26.36227	22.25709	25.69055	23.08979	26.06716	21.89685	3.625415	2.680733	4.570096	24.22728	9.158468	2.670024	4.99E-05	0.001989	1	1
317	sp|Q8BP99	CA216_MC	25 kDa	0.999646	7	1	Q8BP99	NA	UPF0500 p	22.22913	17.70089	22.15634	19.02669	22.08825	19.02444	3.573903	2.569788	4.578018	20.37096	8.493954	2.195138	7.97E-05	0.002702	1	1
631	sp|Q924W	RBTN1_MO	18 kDa	0.999432	4	0.25	Q924W9	Lmo1	Rhombotin	20.35864	15.1481	20.83886	17.96954	19.56281	16.95335	3.563107	1.795188	5.331027	18.47189	4.809689	-1.28917	0.002197	0.016125	1	1
1019	sp|P07759	SPA3K_MO	47 kDa	0.999735	2	1	P07759	Serpina3k	Serine prote	18.43752	14.04793	19.3601	NA	18.39439	16.31902	3.547192	1.705851	5.388533	17.31179	4.775089	-1.56869	0.003536	0.021945	1	1
222	sp|O09044	SNP23_MO	23 kDa	0.999882	9	0.89	O09044	Snap23	Synaptosor	24.79802	21.10309	24.14536	20.64175	24.43624	21.11742	3.505791	2.767889	4.243692	22.70698	11.30807	4.061053	1.2E-05	0.000976	1	1
137	sp|Q3UZ01	RNPC3_MC	58 kDa	0.999248	13	1	Q3UZ01	Rnpc3	RNA-bindin	25.61458	20.83318	24.21064	22.35387	25.69598	21.84229	3.497288	2.152342	4.842235	23.42509	6.205503	0.22841	0.000527	0.007135	1	1
955	sp|Q9DBP5	KCY_MOUS	22 kDa	0.99784	3	1	Q9DBP5	Cmpk1	UMP-CMP k	17.69648	16.82647	17.40337	14.87972	18.18634	11.29231	3.429234	0.354809	6.503659	16.04745	2.661851	-4.17299	0.03373	0.087525	1	0
1058	sp|Q5SUR0	PUR4_MOU	145 kDa	0.999277	2	1	Q5SUR0	Pfas	Phosphorib	18.74609	17.12656	18.66624	12.98109	19.0687	16.2118	3.387192	0.985407	5.788977	17.13341	3.365552	-3.16379	0.012811	0.047575	1	1
240	sp|Q9WTY1	PDCD7_MC	54 kDa	0.999289	9	1	Q9WTY1	Pdcd7	Programme	24.95165	20.8272	23.6166	21.2312	25.04715	21.50362	3.351132	2.257519	4.444745	22.8629	7.312712	1.250699	0.000199	0.004622	1	1
1277	sp|Q9D287	SPF27_MO	26 kDa	0.99577	2	1	Q9D287	Bcas2	Pre-mRNA-	18.70835	13.9885	18.59317	17.27421	18.81194	15.04786	3.267629	1.385691	5.149567	17.07067	4.143602	-2.1152	0.004767	0.026134	1	1
1278.1	sp|Q7TSF0	DSG1C_MC	101 kDa	0.995714	2	1	Q7TSF0	Dsg1c	Desmoglein	18.70835	13.9885	18.59317	17.27421	18.81194	15.04786	3.267629	1.385691	5.149567	17.07067	4.143602	-2.1152	0.004767	0.026134	1	1
180	sp|Q80U40	RIMB2_MO	118 kDa	0.998865	11	1	Q80U40	Rimbp2	RIMS-bindi	22.50782	19.61629	22.41039	18.36859	23.01815	20.31138	3.213367	1.9648	4.461934	21.03877	6.14185	0.165072	0.00056	0.007302	1	1
18	sp|P63017	HSP7C_MC	71 kDa	0.999881	31	0.65	P63017	Hspa8	Heat shock	27.85382	24.30508	27.7775	24.49842	27.83724	25.23323	3.143945	2.361162	3.926729	26.25088	9.559479	3.007402	3.52E-05	0.001704	1	1
1184	sp|Q8K386	RAB15_MO	24 kDa	0.998107	2	0.5	Q8K386	Rab15	Ras-related	18.95745	15.91514	19.35923	16.2916	19.33877	16.0425	3.135401	2.470756	3.800046	17.65078	11.22805	4.017113	1.25E-05	0.000976	1	1
1002.2	sp|Q80VQ	AL3B1_MO	52 kDa	0.995458	3	1	Q80VQ0	Aldh3b1	Aldehyde d	20.88722	19.67256	23.96654	17.32303	21.72996	20.42034	3.05593	0.571543	5.540317	20.66661	2.935451	-3.77595	0.02298	0.067424	1	0
817	tr|A0A0B4J	A0A0B4J1N	11 kDa	0.999419	3	0.33	A0A0B4J1N	Ighv1-77	Immunoglo	19.53749	16.75791	19.94923	16.80292	19.96815	16.76371	3.043447	2.402736	3.684159	18.29657	11.30587	4.059855	1.2E-05	0.000976	1	1
1034	tr|A0A075E	A0A075B67	10 kDa	0.999481	2	1	A0A075B67	Igkv4-53	Immunoglo	22.30582	18.99434	21.35155	19.49426	22.41964	18.67668	2.970574	2.010291	3.930856	20.54038	7.382304	1.310256	0.000188	0.004586	1	1
32	sp|Q6PDG	SMRC2_MC	133 kDa	0.999852	24	0.67	Q6PDG5	Smarcc2	SWI/SNF co	25.9951	22.71468	25.24442	22.74158	26.21342	23.12373	2.957648	2.127008	3.788287	24.33882	8.47531	2.239788	7.54E-05	0.002622	1	1
224	sp|Q3UH6	WNK2_MOU	228 kDa	0.999818	9	0.89	Q3UH66	Wnk2	Serine/thre	21.59838	18.0727	21.81789	19.49968	21.87457	18.92093	2.932511	1.959063	3.90596	20.29736	7.189146	1.143654	0.000221	0.004622	1	1
401	sp|Q9CX30	YIF1B_MOU	34 kDa	0.999669	6	1	Q9CX30	Yif1b	Protein YIF1	25.40531	22.23832	25.71674	23.02212	25.38173	22.52984	2.904495	2.157756	3.651234	24.04901	9.257689	2.803678	4.32E-05	0.001774	1	1
227	sp|Q9QZM	UBQL2_MC	67 kDa	0.999751	9	0.78	Q9QZM0	Ubqln2	Ubiquilin-2	25.1567	22.82399	26.28683	22.33402	24.68113	22.3235	2.881051	1.784037	3.978064	23.93436	6.267431	0.289529	0.000498	0.006888	1	1
336	sp|Q8VCM	FIBG_MOUS	49 kDa	0.999459	7	1	Q8VCM7	Fgg	Fibrinogen g	24.21079	21.07079	23.39936	21.09125	23.81612	20.64447	2.873253	2.09987	3.646637	22.37213	8.842596	2.511556	5.77E-05	0.002206	1	1
1022	sp|P00687	AMY1_MOU	58 kDa	0.999718	2	1	P00687	Amy1	Alpha-amyl	22.9301	20.18507	21.64963	18.50636	23.00559	20.3898	2.834698	1.34859	4.320806	21.11109	4.55205	-1.6006	0.002942	0.019244	1	1
790	sp|P03976	KV2A5_MO	12 kDa	0.999638	3	0.33	P03976	NA	Ig kappa ch	21.81688	19.43468	20.28002	18.3122	21.67842	17.72153	2.768972	1.348592	4.189351	19.87395	4.652269	-1.47825	0.002623	0.018057	1	1
325	sp|P46062	SIPA1_MOU	112 kDa	0.999569	7	1	P46062	Sipa1	Signal-indu	22.8695	19.37953	21.36005	19.55026	22.09384	19.09797	2.765208	1.743169	3.787247	20.72519	6.456705	0.473304	0.000418	0.006259	1	1
825	tr|A0A075E	A0A075B5	11 kDa	0.999375	3	0.33	A0A075B5	Ighv1-56	Immunoglo	23.47433	20.17281	22.34031	20.90487	23.77537	20.27581	2.745506	1.665334	3.825678	21.82392	6.065691	0.088595	0.000602	0.00764	1	1
77	sp|Q91XU0	WRIP1_MO	72 kDa	0.999602	17	1	Q91XU0	Wrnip1	ATPase WR	25.96271	22.74806	24.0297	22.65917	26.33713	22.96971	2.650867	1.199294	4.10244	24.11775	4.358126	-1.84173	0.003688	0.022641	1	1
981	sp|Q6NSU	GL8D1_MC	42 kDa	0.997254	3	1	Q6NSU3	Glt8d1	Glycosyltra	18.38342	16.25019	15.80608	13.96295	19.09967	15.22438	2.617219	0.330419	4.904018	16.45445	2.731257	-4.07193	0.030577	0.081781	1	0
869	sp|Q61543	GSLG1_MC	134 kDa	0.998993	3	1	Q61543	Glg1	Golgi appar	22.33651	19.98973	22.35442	19.45564	22.00334	19.49077	2.58604	1.888317	3.283763	20.9384	8.821714	2.496481	5.86E-05	0.002206	1	1
1068	sp|Q9DCR	AP3S1_MC	22 kDa	0.999217	2	1	Q9DCR2	Ap3s1	AP-3 comp	21.64431	17.63543	19.06564	18.13848	21.87576	19.16112	2.550227	0.605097	4.495357	19.58679	3.128822	-3.49846	0.017617	0.056254	1	0
1242	sp|P21278	GNA11_MC	42 kDa	0.997167	2	1	P21278	Gna11	Guanine nu	16.86335	14.97846	18.33561	17.22075	17.6544	13.02014	2.544668	0.102554	4.986782	16.34545	2.486656	-4.42848	0.043297	0.105893	1	0
113	tr|Q3UJB0	Q3UJB0_M	98 kDa	0.999862	14	1	Q3UJB0	Sf3b2	Splicing fac	24.56331	21.89792	23.82033	22.09233	24.76927	21.53842	2.541412	1.698843	3.383981	23.1136	7.184315	1.174536	0.000212	0.004622	1	1
487	sp|P29699	FETUA_MO	37 kDa	0.99975	5	1	P29699	Ahsg	Alpha-2-HS	25.11261	22.42983	25.37017	22.68068	24.46618	22.30354	2.511637	1.720119	3.303154	23.72717	7.552614	1.505597	0.000154	0.004094	1	1
7	sp|P70336	ROCK2_MC	161 kDa	0.99975	49	1	P70336	Rock2	Rho-associ	27.49656	24.87803	28.27345	24.56162	26.65873	25.46058	2.509501	1.362953	3.656049	26.22149	5.223309	-0.81026	0.001403	0.012336	1	1
614	sp|O70439	STX7_MOU	30 kDa	0.999563	4	1	O70439	Stx7	Syntaxin-7	24.47173	21.79934	24.09237	22.1601	24.28612	21.40442	2.495454	1.75852	3.232387	23.03568	8.059757	1.919958	0.000103	0.003176	1	1
171	sp|Q99KN	EPN4_MOU	69 kDa	0.999326	11	1	Q99KN9	Clint1	Clathrin inte	24.55638	21.62096	24.17584	21.75137	23.7416	21.67437	2.47571	1.745107	3.206314	22.92009	8.065269	1.924321	0.000103	0.003176	1	1
1046	sp|Q80TT2	BAIP3_MOU	127 kDa	0.999382	2	1	Q80TT2	Baiap3	BAI1-assoc	18.3657	15.61973	18.83519	17.41713	19.38739	16.12937	2.474014	1.205602	3.742425	17.62575	4.654709	-1.47529	0.002616	0.018057	1	1
760	sp|Q8R3N	THOC1_MC	75 kDa	0.997356	4	1	Q8R3N6	Thoc1	THO compl	21.25154	18.81515	20.96518	18.36662	21.37676	19.01436	2.465782	1.747415	3.184149	19.96494	8.169757	2.006487	9.47E-05	0.003058	1	1
47	sp|Q9QX47	SON_MOUS	266 kDa	0.999742	20	1	Q9QX47	Son	Protein SON	23.98495	21.62267	23.9458	21.34901	24.5157	22.22077	2.417995	1.5945	3.24149	22.93982	6.989019	1.012516	0.000247	0.004931	1	1
812	sp|Q61753	SERA_MOU	57 kDa	0.999441	3	1	Q61753	Phgdh	D-3-phospl	18.26501	15.45136	18.07564	14.6353	17.04925	16.12053	2.394237	1.1893	3.599173	16.59952	4.741913	-1.37011	0.002371	0.017008	1	1
191	sp|Q3U0V1	FUBP2_MO	77 kDa	0.999648	10	0.9	Q3U0V1	Khsrp	Far upstrea	24.72273	21.66067	24.14036	21.8274	23.23654	21.44241	2.389714	1.380986	3.398441	22.83835	5.653567	-0.33875	0.000901	0.009848	1	1
836.1	sp|P01638	KV5A6_MO	13 kDa	0.999277	3	1	P01638	NA	Ig kappa ch	17.54744	14.73293	15.63094	16.03972	17.94353	13.2049	2.381449	0.29432	4.468578	15.84991	2.722969	-4.08399	0.030937	0.082417	1	0
12	sp|Q8CGB	TNS2_MOU	152 kDa	0.999844	38	0.97	Q8CGB6	Tns2	Tensin-2	27.48814	25.23302	27.2899	24.28074	27.30562	25.42792	2.380662	1.497997	3.263327	26.17089	6.429779	0.463807	0.00042	0.006259	1	1
235	sp|Q9Z2N8	ACL6A_MO	47 kDa	0.999542	9	0.89	Q9Z2N8	Actl6a	Actin-like pr	24.21382	21.73671	23.18929	21.35708	24.51204	21.71926	2.367363	1.399225	3.335502	22.78803	5.8355	-0.14725	0.000752	0.008839	1	1
826	sp|Q9CQN	TRAP1_MO	80 kDa	0.999366	3	1	Q9CQN1	Trap1	Heat shock	21.91922	18.94401	19.9797	19.23083	22.06275	18.69712	2.363236	0.965036	3.761437	20.13894	4.033558	-2.2582	0.005452	0.027897	1	1
511	sp|Q3TWN	CNNM2_M	97 kDa	0.999457	5	1	Q3TWN3	Cnnm2	Metal transp	20.05472	17.92137	19.41725	17.7696	20.64281	17.37158	2.350741	1.425285	3.276196	18.86289	6.060599	0.086212	0.000603	0.00764	1	1
831	sp|Q0VBL3	RBM15_MC	106 kDa	0.999348	3	1	Q0VBL3	Rbm15	RNA-bindin	22.4191	20.36229	20.53938	18.73121	21.96914	18.78609	2.34934	0.798737	3.899944	20.46787	3.615728	-2.81743	0.009231	0.038395	1	1
1259	sp|Q9CU6	SMC1A_MC	143 kDa	0.996524	2	1	Q9CU62	Smc1a	Structural m	19.69182	17.35776	20.37165	18.03578	19.11579	16.78584	2.333295	1.22361	3.44298	18.55977	5.017885	-1.0449	0.001748	0.013699	1	1
209	tr|Q6PHQ9	Q6PHQ9_M	72 kDa	0.999289	10	0.8	Q6PHQ9	Pabpc4	Polyadenyl	22.35413	19.69856	21.93965	19.30669	21.99438	20.32371	2.319733	1.48701	3.152457	20.93619	6.630695	0.680866	0.000339	0.00576	1	1
281	sp|Q61103	REQU_MOU	44 kDa	0.999382	8	0.88	Q61103	Dpf2	Zinc finger p	24.10588	21.64723	23.5233	21.22568	24.4496	22.29356	2.304103	1.347004	3.261203	22.87421	5.74507	-0.24186	0.000822	0.009368	1	1
485	sp|Q9D0L4	ADCK1_MC	60 kDa	0.999797	5	1	Q9D0L4	Adck1	Uncharacte	22.14353	19.38703	21.67331	19.53578	22.35148	20.44388	2.267211	1.346956	3.187467	20.9225	5.877701	-0.09952	0.000718	0.008616	1	1
1014	sp|P12961	7B2_MOUS	24 kDa	0.999876	2	1	P12961	Scg5	Neuroendo	18.97902	17.09026	18.34253	15.80426	19.94018	17.59953	2.255898	0.82642	3.685375	17.9593	3.766108	-2.61326	0.007614	0.034782	1	1
148	sp|Q9WU2	MTHR_MOU	75 kDa	0.999556	12	1	Q9WU20	Mthfr	Methylenet	25.13436	23.70929	24.14165	22.34666	25.26627	21.77208	2.238083	0.863195	3.61297	23.72839	3.884718	-2.45451	0.006557	0.031417	1	1
100.1	sp|P17879	HS71B_MC	70 kDa	0.999699	15	0.73	P17879	Hspa1b	Heat shock	24.99751	22.79349	24.59787	22.00965	24.82205	22.90713	2.23572	1.426672	3.044768	23.68795	6.577565	0.630384	0.000356	0.00576	1	1
100.2	sp|Q61696	HS71A_MC	70 kDa	0.999699	15	0.73	Q61696	Hspa1a	Heat shock	24.99751	22.79349	24.59787	22.00965	24.82205	22.90713	2.23572	1.426672	3.044768	23.68795	6.577565	0.630384	0.000356	0.00576	1	1
10	sp|Q9EPU	RENT1_MO	124 kDa	0.999794	40	1	Q9EPU0	Upf1	Regulator o	26.88991	24.32191	26.69588	24.45162	26.57692	24.69033	2.232949	1.592188	2.873711	25.60443	8.294372	2.10313	8.62E-05	0.002851	1	1
553	sp|P62141	PP1B_MOU	37 kDa	0.998911	5	0.4	P62141	Ppp1cb	Serine/thre	22.1122	19.15572	21.42328	20.32593	22.05817	19.44851	2.221164	1.258729	3.183599	20.75397	5.507569	-0.49578	0.001044	0.010776	1	1
176	sp|Q9Z204	HNRPC_MC	34 kDa	0.999252	11	1	Q9Z204	Hnrnpc	Heterogene	25.77075	23.35799	24.80237	23.26727	26.29288	23.58338	2.21912	1.21016	3.228079	24.51244	5.248771	-0.78161	0.001365	0.012261	1	1
967	sp|Q99LX0	PARK7_MO	20 kDa	0.997612	3	1	Q99LX0	Park7	Protein/nuc	19.73811	17.72553	20.29779	17.60711	19.48512	17.55125	2.212375	1.473917	2.950833	18.73415	7.130727	1.140447	0.000219	0.004622	1	1
133	sp|Q8C3F2	F120C_MO	120 kDa	0.999553	13	0.85	Q8C3F2	Fam120c	Constitutive	24.45541	21.95124	24.25257	21.71945	23.83938	22.35075	2.175304	1.422157	2.928451	23.0948	6.874494	0.909007	0.000273	0.005213	1	1
5	sp|Q8R0W	EPIPL_MOU	723 kDa	0.999889	53	0.96	Q8R0W0	Eppk1	Epiplakin	26.42704	23.51274	24.96372	24.08294	26.96435	24.25857	2.166955	0.852678	3.481232	25.03489	3.934718	-2.3882	0.00616	0.030487	1	1
328	sp|Q8BRM	GORAB_MC	41 kDa	0.999541	7	1	Q8BRM2	Gorab	RAB6-intera	24.13988	21.38348	22.70421	21.12528	23.93925	21.7973	2.159091	1.084359	3.233824	22.5149	4.794256	-1.30754	0.002236	0.016125	1	1
437	sp|O55203	LDB2_MOU	43 kDa	0.999263	6	0.83	O55203	Ldb2	LIM domain	23.60646	21.88231	23.91088	20.79993	23.29214	21.68058	2.148885	1.242273	3.055498	22.52872	5.650481	-0.32866	0.000888	0.009789	1	1
652	sp|Q9R226	KHDR3_MC	39 kDa	0.99932	4	0.75	Q9R226	Khdrbs3	KH domain-	23.03737	20.64425	21.33682	19.77296	22.90573	20.54607	2.105547	0.835703	3.375391	21.37387	3.956992	-2.35877	0.005992	0.029873	1	1
928	sp|P01831	THY1_MOU	18 kDa	0.998266	3	1	P01831	Thy1	Neuroendo	18.01021	16.79774	18.45987	15.83689	18.24632	15.81	2.090589	1.221915	2.959262	17.19351	5.73728	-0.23635	0.000814	0.009355	1	1
891	sp|Q8BG32	PSD11_MC	47 kDa	0.998773	3	1	Q8BG32	Psmd11	26S proteas	22.34552	19.62844	19.25061	18.66524	22.13427	19.29053	2.048736	0.048762	4.04871	20.2191	2.444625	-4.48977	0.045988	0.109594	1	0
892	sp|Q80WC	AGFG2_MC	49 kDa	0.99877	3	1	Q80WC7	Agfg2	Arf-GAP dor	22.34552	19.62844	19.25061	18.66524	22.13427	19.29053	2.048736	0.048762	4.04871	20.2191	2.444625	-4.48977	0.045988	0.109594	1	0
98	sp|Q80U23	SNPH_MOU	54 kDa	0.999775	15	0.87	Q80U23	Snph	Syntaphilin	24.56258	20.65571	23.42978	21.67479	24.41434	23.97825	2.032649	0.019173	4.046125	23.11924	2.409166	-4.54144	0.048392	0.112169	1	0
330	sp|Q9Z0H3	SNF5_MOU	44 kDa	0.999526	7	1	Q9Z0H3	Smarcb1	SWI/SNF-re	24.42627	22.33792	23.4462	21.24587	24.44884	22.66562	2.023966	0.867448	3.180484	23.09512	4.176393	-2.07294	0.004581	0.025802	1	1
128	sp|Q8CJG0	AGO2_MOU	97 kDa	0.999715	13	0.85	Q8CJG0	Ago2	Protein argo	23.84731	21.8418	23.43332	21.78657	23.93438	21.52956	2.019025	1.344763	2.693288	22.72882	7.127113	1.137234	0.000219	0.004622	1	1
534	sp|Q91YN9	BAG2_MOU	23 kDa	0.999217	5	1	Q91YN9	Bag2	BAG family	22.41719	20.56972	22.06817	20.48345	22.55459	19.98094	2.001945	1.276328	2.727562	21.34568	6.566671	0.620783	0.000359	0.00576	1	1
21	sp|O08810	U5S1_MOU	109 kDa	0.999725	28	0.96	O08810	Eftud2	116 kDa U5	25.76133	24.08064	24.68752	22.86079	26.05939	23.60689	1.986638	0.821078	3.152199	24.50943	4.067567	-2.21381	0.005229	0.027525	1	1
1081	sp|Q3TJZ6	FA98A_MO	55 kDa	0.999131	2	1	Q3TJZ6	Fam98a	Protein FAM	26.18688	23.61623	25.0779	23.13685	26.61336	25.17665	1.982799	0.452619	3.51298	24.96798	3.092337	-3.55056	0.018516	0.058038	1	0
888	sp|Q9CYK2	QPCT_MOU	41 kDa	0.998803	3	1	Q9CYK2	Qpct	Glutaminyl	22.55832	20.3088	22.12631	20.06781	21.77316	20.14065	1.98018	1.250027	2.710333	21.16251	6.454927	0.513303	0.000398	0.006063	1	1
505	sp|Q9CQF3	CPSF5_MO	26 kDa	0.999523	5	1	Q9CQF3	Nudt21	Cleavage ar	23.55598	21.18985	22.98271	21.12811	23.0107	21.40734	1.94136	1.245526	2.637194	22.21245	6.640507	0.690958	0.000336	0.00576	1	1
127	sp|Q8BHS8	SYBU_MOU	73 kDa	0.999745	13	0.85	Q8BHS8	Sybu	Syntabulin	24.71025	23.33591	23.35876	20.98313	24.62368	22.5543	1.939781	0.322297	3.557264	23.26101	2.861959	-3.88219	0.025454	0.072058	1	0
331	sp|Q99KP6	PRP19_MO	55 kDa	0.999488	7	1	Q99KP6	Prpf19	Pre-mRNA-	24.82743	22.70567	24.27015	23.05885	25.17441	22.69926	1.93607	1.146878	2.725263	23.78929	5.839006	-0.10786	0.000716	0.008616	1	1
1280	sp|Q8K0L9	ZBT20_MO	81 kDa	0.995702	2	1	Q8K0L9	Zbtb20	Zinc finger a	20.26318	18.44949	22.24132	20.89245	21.43402	18.80873	1.929284	0.076287	3.782281	20.3482	2.48469	-4.43135	0.043419	0.105893	1	0
621	sp|Q9ER00	STX12_MO	31 kDa	0.999496	4	1	Q9ER00	Stx12	Syntaxin-12	23.70315	22.0989	23.50824	20.65139	23.50365	22.18932	1.925143	0.830354	3.019932	22.60911	4.196461	-2.04716	0.004472	0.025501	1	1
990	sp|Q3THK7	GUAA_MOU	77 kDa	0.99685	3	1	Q3THK7	Gmps	GMP syntha	19.43538	18.31149	19.37049	17.27847	19.24929	16.69458	1.92354	0.870142	2.976938	18.38995	4.357721	-1.84224	0.00369	0.022641	1	1
1122	tr|B2RXQ2	B2RXQ2_M	143 kDa	0.998694	2	1	B2RXQ2	Ppfia1	Ppfia1 prot	20.92404	19.22578	20.93676	18.99896	20.78955	18.725	1.900201	1.251107	2.549294	19.93335	6.967754	0.994115	0.000252	0.004945	1	1
1261	sp|P51660	DHB4_MOU	79 kDa	0.996427	2	1	P51660	Hsd17b4	Peroxisoma	20.02291	18.00302	19.22207	17.81911	20.25533	17.99475	1.894478	1.067929	2.721027	18.8862	5.455607	-0.52104	0.001057	0.010776	1	1
428	sp|P70333	HNRH2_MC	49 kDa	0.999352	6	0.33	P70333	Hnrnph2	Heterogene	22.53427	20.68878	23.45805	20.71131	23.04984	22.05715	1.861641	0.730894	2.992388	22.08323	3.928993	-2.39577	0.006204	0.030593	1	1
121	sp|O70133	DHX9_MOU	149 kDa	0.999414	14	1	O70133	Dhx9	ATP-depend	24.84958	22.61174	24.1873	22.58325	24.62655	22.89707	1.857126	1.147273	2.566979	23.62592	6.226926	0.289132	0.000492	0.006878	1	1
786	tr|A0A075E	A0A075B67	11 kDa	0.999674	3	0.33	A0A075B67	Ighv1-78	Immunoglo	21.42798	18.9608	20.20741	20.11767	21.60376	18.59672	1.854654	0.546871	3.162437	20.15239	3.384371	-3.13746	0.012495	0.047066	1	1
1056.2	tr|Q63ZW6	Q63ZW6_M	162 kDa	0.999285	2	1	Q63ZW6	Col4a5	Col4a5 pro	19.54683	18.24412	19.82987	18.05429	19.42494	16.95742	1.848606	0.881459	2.815752	18.67624	4.561446	-1.58906	0.00291	0.019158	1	1
863	sp|P60867	RS20_MOU	13 kDa	0.99904	3	1	P60867	Rps20	40S riboson	22.76709	20.86503	23.24579	21.0859	22.65141	21.19691	1.838817	1.141489	2.536146	21.96869	6.276274	0.338213	0.00047	0.006775	1	1
178	sp|Q68FH0	PKP4_MOU	132 kDa	0.999035	11	1	Q68FH0	Pkp4	Plakophilin	22.72108	20.14036	21.18809	19.96626	21.97705	20.28417	1.83181	0.814718	2.848901	21.04617	4.298044	-1.91761	0.00396	0.02377	1	1
699	sp|P33267	CP2F2_MO	56 kDa	0.998901	4	1	P33267	Cyp2f2	Cytochrom	19.63472	17.50575	19.24635	17.57778	19.99771	18.32354	1.823905	0.969059	2.678751	18.71431	5.085218	-0.95373	0.001596	0.013037	1	1
163	sp|Q8BPB5	FBLN3_MO	55 kDa	0.999504	11	1	Q8BPB5	Efemp1	EGF-contai	25.46593	23.13289	24.44394	23.06345	25.39581	23.70309	1.802079	0.881151	2.723007	24.20085	4.668441	-1.45602	0.002566	0.017983	1	1
777	sp|Q62159	RHOC_MOU	22 kDa	0.99532	4	0.25	Q62159	Rhoc	Rho-related	23.62226	22.13382	23.3445	21.15459	23.28133	21.55788	1.8006	0.994191	2.607008	22.51573	5.314763	-0.67783	0.001225	0.011522	1	1
405	sp|P62754	RS6_MOUS	29 kDa	0.999619	6	1	P62754	Rps6	40S riboson	25.81162	24.16112	26.02414	23.63338	25.38644	24.0423	1.795134	1.055764	2.534505	24.84316	5.778772	-0.17132	0.00076	0.008839	1	1
464	sp|Q6ZPR4	KCNT1_MO	138 kDa	0.998579	6	1	Q6ZPR4	Kcnt1	Potassium	19.77841	17.82771	18.95494	16.91251	20.32247	18.98341	1.777396	0.328975	3.225818	18.79657	2.928464	-3.78604	0.023204	0.067424	1	0
592	sp|Q9D1G	RAB1B_MO	22 kDa	0.999802	4	0.25	Q9D1G1	Rab1b	Ras-related	19.76854	18.12916	20.66956	18.64778	19.87042	18.24636	1.761741	0.926592	2.596891	19.22197	5.02111	-1.0141	0.00168	0.01351	1	1
63	sp|Q9ESU	BRD4_MOU	156 kDa	0.999719	18	1	Q9ESU6	Brd4	Bromodom	25.06452	23.9049	23.7937	22.30319	25.51418	22.88037	1.761315	0.351272	3.171358	23.91014	2.980954	-3.71037	0.021578	0.065165	1	0
1200	sp|O35226	PSMD4_MC	41 kDa	0.997946	2	1	O35226	Psmd4	26S proteas	19.63834	18.04042	19.6948	17.72898	19.696	17.97699	1.760916	1.148333	2.373499	18.79592	6.841865	0.87899	0.000281	0.00529	1	1
741	sp|P49615	CDK5_MOU	33 kDa	0.9981	4	1	P49615	Cdk5	Cyclin-depe	20.73597	19.32378	21.71029	19.15244	20.60443	19.31223	1.754077	0.880937	2.627218	20.13986	4.789152	-1.30366	0.002218	0.016125	1	1
162	sp|P47911	RL6_MOUS	34 kDa	0.999527	11	1	P47911	Rpl6	60S riboson	25.64704	24.0894	25.82642	24.06906	25.67027	23.73458	1.750233	1.118812	2.381655	24.83946	6.597471	0.650136	0.000349	0.00576	1	1
158	sp|P27659	RL3_MOUS	46 kDa	0.999783	11	1	P27659	Rpl3	60S riboson	25.38364	23.54433	25.15199	23.32146	25.12776	23.56829	1.7431	1.121216	2.364985	24.34958	6.671347	0.720072	0.000327	0.00576	1	1
201	sp|P12970	RL7A_MOU	30 kDa	0.999456	10	1	P12970	Rpl7a	60S riboson	24.97129	23.25296	25.23147	23.27719	24.75899	23.24403	1.729186	1.091552	2.36682	24.12266	6.454625	0.51301	0.000398	0.006063	1	1
72	sp|Q9D8E6	RL4_MOUS	47 kDa	0.999669	17	1	Q9D8E6	Rpl4	60S riboson	25.47186	23.96585	25.47296	23.57612	25.53497	23.76307	1.724916	1.102302	2.347531	24.63081	6.594014	0.646847	0.00035	0.00576	1	1
439	sp|Q6NZF1	ZC11A_MO	86 kDa	0.999213	6	1	Q6NZF1	Zc3h11a	Zinc finger C	24.71797	22.96025	23.5417	22.63279	25.27682	22.80947	1.711325	0.589156	2.833494	23.6565	3.639359	-2.78514	0.008953	0.03794	1	1
195	sp|O54941	SMCE1_MC	47 kDa	0.999567	10	1	O54941	Smarce1	SWI/SNF-re	24.90503	23.47545	23.76077	22.6846	25.01228	22.45604	1.687331	0.587594	2.787067	23.71569	3.661527	-2.75491	0.008702	0.03734	1	1
616	sp|P62821	RAB1A_MO	23 kDa	0.999554	4	0.25	P62821	Rab1A	Ras-related	19.98859	18.9982	20.65602	18.91909	20.51377	18.20002	1.680356	0.845515	2.515197	19.54595	4.790927	-1.28672	0.00217	0.016076	1	1
144	sp|Q3UTJ2	SRBS2_MO	132 kDa	0.999691	12	1	Q3UTJ2	Sorbs2	Sorbin and	23.3373	20.86913	21.87923	20.29638	23.36335	22.3805	1.677959	0.105736	3.250182	22.02098	2.546935	-4.34056	0.039721	0.098467	1	0
482	sp|O35295	PURB_MOU	34 kDa	0.99993	5	1	O35295	Purb	Transcriptio	22.71308	20.3186	21.9463	20.82005	22.24894	20.75416	1.671833	0.901958	2.441707	21.46685	5.168606	-0.84306	0.00143	0.012407	1	1
620	sp|Q9CR57	RL14_MOU	24 kDa	0.999496	4	1	Q9CR57	Rpl14	60S riboson	25.19446	23.55456	25.51297	23.76991	25.40258	23.79248	1.664354	1.038076	2.290632	24.53783	6.325271	0.386636	0.000449	0.006612	1	1

59	sp|Q3TKT4	SMCA4_MO	181 kDa	0.999626	19	0.58	Q3TKT4	Smarca4	Transcriptio	23.64992	21.92278	22.80156	22.20405	24.01612	21.35074	1.663339	0.66639	2.660288	22.65753	3.981607	-2.32635	0.005812	0.029083	1	1
119	sp|Q8VEK3	HNRPU_MO	88 kDa	0.999549	14	1	Q8VEK3	Hnrnpu	Heterogene	26.3786	24.6574	26.38156	24.48236	26.37727	25.01479	1.660958	1.008343	2.313572	25.54866	6.057632	0.118341	0.000578	0.007469	1	1
1210	sp|Q8R326	PSPC1_MO	59 kDa	0.99779	2	1	Q8R326	Pspc1	Paraspeckle	19.0285	17.98653	18.45406	16.52011	18.40393	16.43615	1.647899	0.491239	2.80456	17.80488	3.399973	-3.11566	0.01224	0.046361	1	1
321	sp|O88665	BRD7_MOU	74 kDa	0.999593	7	1	O88665	Brd7	Bromodom	22.8287	20.91494	21.83513	20.89656	23.0596	20.983	1.642973	0.737377	2.54857	21.75299	4.325039	-1.87532	0.003791	0.022951	1	1
590	sp|Q6P542	ABCF1_MO	95 kDa	0.999828	4	1	Q6P542	Abcf1	ATP-binding	20.71838	17.61124	18.74682	17.92869	18.71617	17.72178	1.639884	0.27567	3.004098	18.57385	2.868674	-3.87247	0.025217	0.071535	1	0
1234	tr|Q9JHF5|	Q9JHF5_MO	93 kDa	0.997355	2	0.5	Q9JHF5	Tcirg1	V-type proto	23.00079	21.08417	23.83329	21.73598	22.65635	21.76223	1.636017	0.675878	2.596156	22.34547	4.066346	-2.2154	0.005237	0.027525	1	1
685	sp|Q6ZWU	RS27_MOU	9 kDa	0.999015	4	0.5	Q6ZWU9	Rps27	40S ribosor	21.56806	19.69359	21.59014	20.16681	21.07589	19.46714	1.635521	0.873956	2.397085	20.59361	5.111524	-0.90868	0.001521	0.012812	1	1
566	sp|Q4FK66	PR38A_MO	37 kDa	0.998592	5	1	Q4FK66	Prpf38a	Pre-mRNA-s	21.39541	18.91557	20.33887	19.80857	21.57198	19.67731	1.634938	0.582709	2.687168	20.28462	3.708016	-2.69176	0.008198	0.036481	1	1
186	sp|P62242	RS8_MOUS	24 kDa	0.999717	10	1	P62242	Rps8	40S ribosor	26.12299	25.33812	26.36842	24.02069	25.86389	24.09249	1.634663	0.617928	2.651397	25.3011	3.836817	-2.51838	0.006963	0.032784	1	1
710	sp|P08226	APOE_MOU	36 kDa	0.998824	4	1	P08226	Apoe	Apolipopro	21.16605	19.66038	21.29676	19.95859	21.53739	19.48436	1.632292	0.96341	2.301174	20.51726	5.808305	-0.14014	0.000738	0.008781	1	1
782	sp|Q9JKK1	STX6_MOU	29 kDa	0.999725	3	1	Q9JKK1	Stx6	Syntaxin-6	21.04916	19.62749	19.84004	19.1177	21.38642	18.66022	1.623402	0.459569	2.787235	19.94684	3.328788	-3.21535	0.013454	0.04801	1	1
824	tr|A0A140T	A0A140T8N	13 kDa	0.999377	3	1	A0A140T8N	Igkv13-84	Immunoglo	23.25403	22.29525	23.27321	21.35865	23.14747	21.18339	1.61248	0.747207	2.477753	22.41867	4.442578	-1.72742	0.0033	0.021108	1	1
315	sp|Q8R081	HNRPL_MO	64 kDa	0.999659	7	1	Q8R081	Hnrnpl	Heterogene	23.52547	21.8458	23.09379	21.48003	23.40808	21.87588	1.608544	0.935155	2.281933	22.53818	5.685484	-0.27059	0.000835	0.009433	1	1
1133	sp|Q9ERU9	RBP2_MOU	341 kDa	0.998587	2	1	Q9ERU9	Ranbp2	E3 SUMO-p	20.797	19.66775	21.43199	19.14767	20.93915	19.54311	1.603206	0.861802	2.34461	20.25445	5.146776	-0.8681	0.001464	0.012567	1	1
142	sp|Q8BG05	ROA3_MOU	40 kDa	0.999911	12	0.75	Q8BG05	Hnrnpa3	Heterogene	25.09629	23.31561	24.26269	23.01576	24.88287	23.11078	1.599899	0.839059	2.360738	23.94733	5.004955	-1.03247	0.001709	0.013629	1	1
277	sp|P62281	RS11_MOU	18 kDa	0.999465	8	1	P62281	Rps11	40S ribosor	24.70272	23.4546	24.58657	22.66491	24.48314	22.88109	1.590609	0.853852	2.327365	23.79551	5.138543	-0.87756	0.001477	0.012599	1	1
104	sp|Q6A068	CDC5L_MO	92 kDa	0.9996	15	0.27	Q6A068	Cdc5l	Cell divisior	22.38783	20.79496	22.74659	20.95253	22.79606	21.42558	1.585804	0.865296	2.306312	21.85059	5.238554	-0.76332	0.001327	0.012077	1	1
452.1	sp|P62270	RS18_MOU	18 kDa	0.999008	6	1	P62270	Rps18	40S ribosor	24.85654	23.21343	24.95486	23.24909	24.33298	22.92725	1.584867	0.875634	2.294099	23.92236	5.318691	-0.67285	0.001219	0.011522	1	1
452.2	tr|A0A1Y7V	A0A1Y7VKY	18 kDa	0.999008	6	1	A0A1Y7VKY	Gm11361	MCG11667	24.85654	23.21343	24.95486	23.24909	24.33298	22.92725	1.584867	0.875634	2.294099	23.92236	5.318691	-0.67285	0.001219	0.011522	1	1
452.3	tr|F6YVP7|	F6YVP7_MO	18 kDa	0.999008	6	1	F6YVP7	Gm10260	Predicted ge	24.85654	23.21343	24.95486	23.24909	24.33298	22.92725	1.584867	0.875634	2.294099	23.92236	5.318691	-0.67285	0.001219	0.011522	1	1
170	sp|P48962	ADT1_MOU	33 kDa	0.999351	11	0.55	P48962	Slc25a4	ADP/ATP tra	24.79143	23.2315	24.44439	22.75918	24.49906	23.01531	1.576296	0.909271	2.243321	23.79015	5.624665	-0.33597	0.000888	0.009789	1	1
1040	sp|P62267	RS23_MOU	16 kDa	0.99942	2	1	P62267	Rps23	40S ribosor	24.25166	22.62279	24.47838	22.71259	24.08159	22.75469	1.573851	0.946897	2.200804	23.48362	5.974877	0.033478	0.000627	0.007788	1	1
111	sp|Q62095	DDX3Y_MO	73 kDa	0.997675	15	0.13	Q62095	Ddx3y	ATP-depend	19.10992	17.52939	19.08762	16.97791	18.737	17.74815	1.559697	0.805989	2.313406	18.19833	4.925359	-1.12604	0.001865	0.014455	1	1
770	sp|Q9WUA	SYFB_MOUS	66 kDa	0.996919	4	1	Q9WUA2	Farsb	Phenylalan	21.28569	19.8359	22.92422	20.56716	21.21848	20.46881	1.518838	0.263461	2.774214	21.05004	2.887274	-3.84556	0.024571	0.070145	1	0
23	sp|Q61768	KINH_MOU	110 kDa	0.999746	27	0.81	Q61768	Kif5b	Kinesin-1 he	24.23015	22.50157	24.08387	22.4242	24.27546	23.13422	1.509827	0.785926	2.233728	23.44158	4.964192	-1.08027	0.001787	0.013927	1	1
8	sp|Q6P4T2	U520_MOU	245 kDa	0.999706	46	1	Q6P4T2	Snrnp200	U5 small nu	26.14711	24.35526	25.19469	24.83136	26.5794	24.24679	1.495931	0.486917	2.504946	25.22577	3.538059	-2.92409	0.010209	0.040629	1	1
267	sp|P62806	H4_MOUSE	11 kDa	0.999599	8	1	P62806	Hist4h4	Histone H4	28.31263	27.3346	27.27886	25.73448	28.04698	26.0879	1.49383	0.282503	2.705158	27.13258	2.943	-3.76506	0.022741	0.067396	1	0
399	sp|Q3B7Z2	OSBP1_MO	89 kDa	0.999671	6	1	Q3B7Z2	Osbp	Oxysterol-b	22.60444	20.02101	21.7037	21.84946	22.58158	20.61257	1.468892	0.193964	2.743819	21.56212	2.749507	-4.04539	0.029801	0.080406	1	0
382	sp|Q99PU8	DHX30_MO	137 kDa	0.99821	7	1	Q99PU8	Dhx30	ATP-depend	20.1292	18.1591	19.60263	17.52875	19.21206	18.85229	1.467917	0.430368	2.505465	18.914	3.376321	-3.14872	0.01263	0.047309	1	1
559	sp|Q80YR5	SAFB2_MO	112 kDa	0.99883	5	1	Q80YR5	Safb2	Scaffold att	23.31818	21.69918	22.26191	21.08378	23.0486	21.47517	1.456853	0.568295	2.345411	22.1478	3.908624	-2.41626	0.006302	0.030671	1	1
1027	sp|P04247	MYG_MOUS	17 kDa	0.999598	2	1	P04247	Mb	Myoglobin	19.8928	18.99333	20.06902	18.93971	20.65781	18.32598	1.453536	0.631476	2.275597	19.47977	4.208661	-2.01249	0.004288	0.02506	1	1
385	sp|P17427	AP2A2_MO	104 kDa	0.997759	7	0.57	P17427	Ap2a2	AP-2 compl	21.497	19.59318	21.49084	19.78374	20.31035	19.56154	1.45324	0.512366	2.394115	20.37278	3.686009	-2.72162	0.008433	0.03672	1	1
627.1	sp|P10853	H2B1F_MO	14 kDa	0.999468	4	1	P10853	Hist1h2bn	Histone H2	27.60808	26.65783	26.88069	25.35859	27.35263	25.46878	1.452069	0.411749	2.492388	26.55443	3.330972	-3.21228	0.013415	0.047997	1	1
627.2	sp|P10854	H2B1M_MO	14 kDa	0.999468	4	1	P10854	Hist1h2bm	Histone H2	27.60808	26.65783	26.88069	25.35859	27.35263	25.46878	1.452069	0.411749	2.492388	26.55443	3.330972	-3.21228	0.013415	0.047997	1	1
627.3	sp|Q6ZWY9	H2B1C_MO	14 kDa	0.999468	4	1	Q6ZWY9	Hist1h2bg	Histone H2	27.60808	26.65783	26.88069	25.35859	27.35263	25.46878	1.452069	0.411749	2.492388	26.55443	3.330972	-3.21228	0.013415	0.047997	1	1
627.4	sp|Q8CGP1	H2B1K_MO	14 kDa	0.999468	4	1	Q8CGP1	Hist1h2bk	Histone H2	27.60808	26.65783	26.88069	25.35859	27.35263	25.46878	1.452069	0.411749	2.492388	26.55443	3.330972	-3.21228	0.013415	0.047997	1	1
627.5	sp|Q8CGP2	H2B1P_MO	14 kDa	0.999468	4	1	Q8CGP2	Hist1h2bp	Histone H2	27.60808	26.65783	26.88069	25.35859	27.35263	25.46878	1.452069	0.411749	2.492388	26.55443	3.330972	-3.21228	0.013415	0.047997	1	1
627.6	sp|Q64475	H2B1B_MO	14 kDa	0.999468	4	1	Q64475	Hist1h2bb	Histone H2	27.60808	26.65783	26.88069	25.35859	27.35263	25.46878	1.452069	0.411749	2.492388	26.55443	3.330972	-3.21228	0.013415	0.047997	1	1
627.7	sp|Q64478	H2B1H_MO	14 kDa	0.999468	4	1	Q64478	Hist1h2bh	Histone H2	27.60808	26.65783	26.88069	25.35859	27.35263	25.46878	1.452069	0.411749	2.492388	26.55443	3.330972	-3.21228	0.013415	0.047997	1	1
627.8	sp|Q64525	H2B2B_MO	14 kDa	0.999468	4	1	Q64525	Hist2h2bb	Histone H2	27.60808	26.65783	26.88069	25.35859	27.35263	25.46878	1.452069	0.411749	2.492388	26.55443	3.330972	-3.21228	0.013415	0.047997	1	1
489	sp|P47963	RL13_MOU	24 kDa	0.99974	5	1	P47963	Rpl13	60S ribosor	25.0958	23.73636	25.33176	23.65812	24.96917	23.67106	1.443731	0.824413	2.06305	24.41038	5.548471	-0.41861	0.00096	0.010246	1	1
441	sp|P62717	RL18A_MO	21 kDa	0.999204	6	1	P62717	Rpl18a	60S ribosor	23.75002	22.17157	23.98249	22.46415	23.73768	22.51228	1.440731	0.805961	2.0755	23.10303	5.402163	-0.57961	0.001117	0.011262	1	1
657	sp|Q9QUI0	RHOA_MOU	22 kDa	0.999264	4	0.25	Q9QUI0	Rhoa	Transformin	19.77692	17.76794	20.3614	18.64351	19.62169	19.0265	1.440683	0.448794	2.432572	19.19966	3.46622	-3.02345	0.011216	0.043453	1	1
112	sp|P06880	SOMA_MOU	25 kDa	0.999893	14	1	P06880	Gh1	Somatotrop	30.46798	29.19401	31.5654	30.12217	31.10104	29.50956	1.436224	0.468745	2.403703	30.32669	3.542676	-2.91772	0.010148	0.040629	1	1
394	sp|Q9CZT8	RAB3B_MO	25 kDa	0.999744	6	0.33	Q9CZT8	Rab3b	Ras-related	22.3992	21.13097	22.84088	20.58854	22.00084	21.22692	1.4315	0.611413	2.251587	21.69789	4.154831	-2.08221	0.004578	0.025802	1	1
28	sp|A2BH40	ARI1A_MOU	242 kDa	0.999848	25	0.92	A2BH40	Arid1a	AT-rich inter	23.41653	22.00725	22.88232	21.53515	23.66679	22.13474	1.429503	0.633365	2.225641	22.60713	4.273634	-1.92856	0.003962	0.02377	1	1
532	sp|Q6NVF9	CPSF6_MO	59 kDa	0.999221	5	1	Q6NVF9	Cpsf6	Cleavage ar	23.60424	22.58169	23.01529	21.44957	23.17718	21.52069	1.414919	0.466095	2.363744	22.55811	3.558742	-2.8956	0.009938	0.040469	1	1
916.11	sp|Q8CGP5	H2A1F_MO	14 kDa	0.998436	3	0.33	Q8CGP5	Hist1h2af	Histone H2A	21.70109	19.53103	20.35261	19.26683	20.57721	19.59098	1.414022	0.431684	2.396359	20.16996	3.435153	-3.06662	0.011684	0.044756	1	1
1075	sp|Q61033	LAP2A_MO	75 kDa	0.999163	2	1	Q61033	Tmpo	Lamina-ass	17.217	16.49091	18.70633	16.7869	17.75882	16.16876	1.411857	0.366835	2.456878	17.18812	3.224155	-3.36294	0.015481	0.051893	1	1
969	sp|Q08460	KCMA1_MO	134 kDa	0.997601	3	1	Q08460	Kcnma1	Calcium-ac	17.678	15.88339	18.00248	16.26589	17.57318	16.89396	1.403468	0.57301	2.233927	17.04948	4.022597	-2.25532	0.005387	0.027767	1	1
1152	sp|P01844	LAC2_MOU	11 kDa	0.99843	2	0.5	P01844	Iglc2	Ig lambda-2	20.71314	19.61536	20.98702	18.86239	20.24305	19.26072	1.401577	0.589401	2.213754	19.94695	4.107604	-2.14373	0.00485	0.026309	1	1
776	sp|Q9D0M	DYL2_MOU	10 kDa	0.995494	4	0.5	Q9D0M5	Dynll2	Dynein ligh	22.52327	20.83074	22.79541	21.85832	22.67702	21.11447	1.39739	0.573796	2.220984	21.96654	4.038558	-2.23429	0.005282	0.027546	1	1
613	sp|P62918	RL8_MOUS	28 kDa	0.99957	4	1	P62918	Rpl8	60S ribosor	23.91971	22.96292	25.35297	23.62357	24.659	23.16656	1.392877	0.367367	2.418387	23.94745	3.241333	-3.33863	0.015127	0.051539	1	1
894	sp|P21107	TPM3_MOU	33 kDa	0.998754	3	0.33	P21107	Tpm3	Tropomyos	23.42961	22.32265	24.02872	22.19628	23.23099	22.03522	1.378388	0.630675	2.126101	22.87391	4.387702	-1.78339	0.003457	0.021602	1	1
811	sp|Q62186	SSRD_MOU	19 kDa	0.999444	3	1	Q62186	Ssr4	Translocon	23.63197	22.07744	23.67203	21.73563	23.51822	22.88704	1.374038	0.512621	2.235455	22.92039	3.802587	-2.55813	0.007203	0.033449	1	1
141	sp|Q9DBG3	AP2B1_MO	105 kDa	0.997806	13	0.46	Q9DBG3	Ap2b1	AP-2 compl	23.15598	21.37204	23.25725	21.8094	21.75426	20.86725	1.372933	0.193299	2.552567	22.03603	2.77749	-4.00472	0.028651	0.078964	1	0
662	sp|P63024	VAMP3_MO	11 kDa	0.999219	4	0.5	P63024	Vamp3	Vesicle-ass	22.11123	20.79044	20.86191	19.33916	21.63336	20.36416	1.370912	0.178921	2.562904	20.85004	2.744651	-4.05245	0.030005	0.080729	1	0
944	sp|Q8K019	BCLF1_MO	106 kDa	0.998011	3	1	Q8K019	Bclaf1	Bcl-2-assoc	21.51691	20.60907	21.94028	20.36035	22.17413	20.55908	1.367607	0.669924	2.065289	21.1933	4.665565	-1.43814	0.0025	0.017844	1	1
508	sp|Q8K0E8	FIBB_MOUS	55 kDa	0.999491	5	1	Q8K0E8	Fgb	Fibrinogen b	22.61871	20.39334	21.63383	21.20591	22.10401	20.67346	1.361281	0.463388	2.259174	21.43821	3.614243	-2.81408	0.00917	0.038262	1	1
524	sp|Q9CZM2	RL15_MOU	24 kDa	0.999297	5	1	Q9CZM2	Rpl15	60S ribosor	24.96063	23.24249	24.88872	23.63036	24.9648	23.87104	1.356754	0.680046	2.033462	24.25967	4.772004	-1.30903	0.002215	0.016125	1	1
136	sp|P14148	RL7_MOUS	31 kDa	0.99931	13	1	P14148	Rpl7	60S ribosor	25.84312	24.28867	26.35916	24.82055	25.8448	24.87377	1.354696	0.607028	2.102364	25.33834	4.312547	-1.87881	0.003781	0.022951	1	1
574	sp|P35276	RAB3D_MO	24 kDa	0.998133	5	0.2	P35276	Rab3d	Ras-related	23.05199	21.57264	22.76164	21.55718	22.76453	21.38654	1.353929	0.73204	1.975819	22.18242	5.181839	-0.82792	0.00141	0.012336	1	1
400	sp|Q6ZWV3	RL10_MOU	25 kDa	0.99967	6	1	Q6ZWV3	Rpl10	60S ribosor	23.70277	22.76863	24.73022	22.67266	23.89147	22.84036	1.347603	0.519987	2.175219	23.43435	3.875745	-2.45063	0.006475	0.031134	1	1
109	sp|P62702	RS4X_MOU	30 kDa	0.999356	15	1	P62702	Rps4x	40S ribosor	24.89577	23.38949	24.89357	23.57903	24.68421	23.46402	1.346999	0.738328	1.95567	24.15102	5.267271	-0.7308	0.001287	0.011873	1	1
88	sp|Q8CHC	SYNJ1_MOU	173 kDa	0.999829	16	1	Q8CHC4	Synj1	Synaptojan	23.53908	21.82992	22.44211	21.43725	23.00188	21.69697	1.339644	0.488383	2.190904	22.32454	3.750784	-2.62676	0.007678	0.034939	1	1
849	sp|Q6ZPJ3	UBE2O_MO	141 kDa	0.999172	3	1	Q6ZPJ3	Ube2o	(E3-indeper	21.22238	20.05973	20.16797	19.47169	21.10626	18.95748	1.335902	0.304726	2.367077	20.16425	3.091666	-3.55152	0.018533	0.058038	1	0
679.1	sp|P02301	H3C_MOUS	15 kDa	0.999062	4	1	P02301	H3f3c	Histone H3	26.84963	25.79016	26.23383	25.38899	27.05354	24.9619	1.331984	0.472108	2.191861	26.04634	3.69281	-2.70671	0.008286	0.036481	1	1
679.2	sp|P68433	H31_MOUS	15 kDa	0.999062	4	1	P68433	Hist1h3i	Histone H3	26.84963	25.79016	26.23383	25.38899	27.05354	24.9619	1.331984	0.472108	2.191861	26.04634	3.69281	-2.70671	0.008286	0.036481	1	1
679.3	sp|P84228	H32_MOUS	15 kDa	0.999062	4	1	P84228	Hist2h3c2	Histone H3	26.84963	25.79016	26.23383	25.38899	27.05354	24.9619	1.331984	0.472108	2.191861	26.04634	3.69281	-2.70671	0.008286	0.036481	1	1
679.4	sp|P84244	H33_MOUS	15 kDa	0.999062	4	1	P84244	H3f3b	Histone H3	26.84963	25.79016	26.23383	25.38899	27.05354	24.9619	1.331984	0.472108	2.191861	26.04634	3.69281	-2.70671	0.008286	0.036481	1	1
596	sp|Q99K48	NONO_MOU	55 kDa	0.999738	4	0.75	Q99K48	Nono	Non-POU d	20.89474	19.68457	21.03482	19.68744	20.48027	19.06889	1.322976	0.562791	2.083162	20.14179	4.142221	-2.09826	0.004647	0.025929	1	1
626	sp|Q9D1M	SEC13_MO	36 kDa	0.999481	4	1	Q9D1M0	Sec13	Protein SEC	22.36954	20.35718	21.84705	20.1058	21.82239	21.61808	1.319306	0.227413	2.4112	21.35334	2.883474	-3.85105	0.024702	0.070369	1	0
536	sp|Q922Q8	LRC59_MO	35 kDa	0.999211	5	1	Q922Q8	Lrrc59	Leucine-rich	22.67913	21.96097	22.66218	21.11112	22.70258	21.02209	1.316565	0.514377	2.118754	22.02301	3.90631	-2.40942	0.006227	0.030594	1	1
543	sp|P21956	MFGM_MOU	51 kDa	0.999127	5	1	P21956	Mfge8	Lactadherin	20.43569	19.30064	21.21015	19.72779	20.78354	19.4737	1.309081	0.558832	2.059331	20.15525	4.152997	-2.08424	0.004586	0.025802	1	1
169	sp|Q99JR8	SMRD2_MO	59 kDa	0.999372	11	0.91	Q99JR8	Smarcd2	SWI/SNF-re	23.43968	20.97937	22.3396	22.52246	23.50122	21.8694	1.303092	0.0767	2.529483	22.44195	2.535692	-4.35696	0.040364	0.099878	1	0
87	sp|P61979	HNRPK_MO	51 kDa	0.999832	16	1	P61979	Hnrnpk	Heterogene	25.68902	24.44393	25.63567	24.37872	25.64861	24.2448	1.301951	0.705108	1.898794	25.00679	5.192008	-0.8163	0.001395	0.012336	1	1
607	sp|P62751	RL23A_MO	18 kDa	0.999674	4	1	P62751	Rpl23a	60S ribosor	24.08806	22.72058	24.21192	22.90146	24.01876	22.82157	1.291708	0.688635	1.89478	23.46039	5.097951	-0.92435	0.001544	0.012892	1	1
961	sp|Q64378	FKBP5_MO	51 kDa	0.997747	3	1	Q64378	Fkbp5	Peptidyl-pr	21.81719	19.52478	21.06636	20.02997	21.8661	21.34804	1.282288	0.028515	2.536062	20.94207	2.440716	-4.49547	0.046246	0.109825	1	0
174	sp|P51881	ADT2_MOU	33 kDa	0.999284	11	0.55	P51881	Slc25a5	ADP/ATP tra	24.52507	23.05248	23.82315	22.66325	24.18888	22.97536	1.282003	0.555055	2.008951	23.53803	4.197458	-2.02661	0.004344	0.025066	1	1
951	sp|Q8K1M6	DNM1L_MO	83 kDa	0.997901	3	1	Q8K1M6	Dnm1l	Dynamin-1-	20.22039	18.34894	20.94661	19.36239	20.20156	19.83991	1.27244	0.176408	2.368472	19.81997	2.770543	-4.01481	0.028932	0.079416	1	0
962	sp|P20357	MTAP2_MO	199 kDa	0.997708	3	1	P20357	Map2	Microtubule	19.9232	19.08077	19.92721	17.82748	19.56382	18.68972	1.272088	0.347611	2.196565	19.1687	3.283124	-3.27884	0.014283	0.049787	1	1
717	sp|Q9CYH2	PXL2A_MO	24 kDa	0.998714	4	1	Q9CYH2	Prxl2a	Peroxiredox	20.74462	19.6203	21.02182	19.35665	20.32793	19.31563	1.267262	0.552984	1.981541	20.06449	4.222793	-1.9939	0.004213	0.024944	1	1
931	sp|Q3TXS7	PSMD1_MO	106 kDa	0.998211	3	1	Q3TXS7	Psmd1	26S proteas	21.5493	20.55774	22.5378	20.84932	21.24385	20.13167	1.264069	0.261182	2.266957	21.14495	3.00794	-3.67155	0.020789	0.063065	1	0
483	sp|O08547	SC22B_MO	25 kDa	0.999894	5	1	O08547	Sec22b	Vesicle-traff	23.79215	22.57444	24.05767	22.73716	23.46862	22.24778	1.25302	0.538889	1.967152	23.1463	4.176194	-2.05413	0.004458	0.025501	1	1
880	sp|Q9CW0	SMC3_MOU	142 kDa	0.998919	3	1	Q9CW03	Smc3	Structural m	21.30276	20.49528	22.70691	21.01078	22.34286	21.09511	1.25045	0.220391	2.280509	21.49228	2.897043	-3.83143	0.02424	0.06949	1	0
1287	sp|P26638	SYSC_MOU	58 kDa	0.995287	2	1	P26638	Sars	Serine--tRN	17.36066	16.74451	17.39737	16.11356	16.99057	15.13956	1.250322	0.183512	2.317132	16.62437	2.796955	-3.97645	0.027878	0.077783	1	0
167	sp|Q91VR5	DDX1_MOU	82 kDa	0.999436	11	1	Q91VR5	Ddx1	ATP-depend	23.30122	21.80544	22.70698	21.38053	22.84285	21.92862	1.245489	0.50796	1.983019	22.32761	4.019401	-2.25922	0.005406	0.027767	1	1
271	sp|Q9CXY6	ILF2_MOUS	43 kDa	0.999564	8	1	Q9CXY6	Ilf2	Interleukin	22.558	21.56879	22.00128	21.26223	22.60948	20.60511	1.24421	0.382711	2.105708	21.76748	3.442967	-3.05101	0.011475	0.04408	1	1
702	sp|Q9WV5	VAPA_MOU	28 kDa	0.998865	4	0.75	Q9WV55	Vapa	Vesicle-ass	21.03147	20.30019	21.34536	19.71504	21.1408	19.77819	1.241406	0.543529	1.939283	20.55184	4.233854	-1.97965	0.004157	0.024829	1	1
774	tr|A0A075B	A0A075B5I	13 kDa	0.995633	4	0.25	A0A075B5I	Ighv7-3	Immunoglo	22.6625	21.39578	22.08317	21.19738	22.67557	21.21905	1.203013	0.50692	1.899106	21.87224	4.113428	-2.13579	0.004813	0.026213	1	1
9	sp|Q99PV0	PRP8_MOU	274 kDa	0.999619	45	1	Q99PV0	Prpf8	Pre-mRNA-p	26.51989	24.63566	25.40605	25.26912	26.96499	25.38132	1.201608	0.081871	2.321345	25.69617	2.560927	-4.32015	0.038935	0.097052	1	0
608	sp|Q99M28	RNPS1_MO	34 kDa	0.999648	4	1	Q99M28	Rnps1	RNA-bindin	23.17282	21.85916	22.4934	21.54962	22.90669	21.61598	1.182714	0.472782	1.892646	22.26628	3.965189	-2.33098	0.005784	0.029047	1	1
379	sp|Q9QZQ8	H2AY_MOU	40 kDa	0.998402	7	1	Q9QZQ8	H2afy	Core histon	23.14974	22.48891	22.8055	21.74509	23.35294	21.52854	1.18188	0.337856	2.025905	22.51178	3.335293	-3.19822	0.013161	0.047997	1	1
323	sp|P35980	RL18_MOU	22 kDa	0.999574	7	1	P35980	Rpl18	60S ribosor	25.86043	25.50647	26.16436	24.52851	25.80528	24.252	1.181033	0.245946	2.11612	25.35284	3.01412	-3.66267	0.020613	0.062671	1	0
348	sp|P23249	MOV10_MO	114 kDa	0.999342	7	1	P23249	Mov10	Putative hel	21.27676	19.93984	21.55647	20.3593	20.98829	19.98992	1.177482	0.475892	1.879071	20.6851	3.994585	-2.29202	0.005575	0.028171	1	1
781	tr|Q3U4B1	Q3U4B1_M	37 kDa	0.999788	3	0.67	Q3U4B1	Ssbp4	Single stran	20.36239	19.84935	21.6409	19.64033	20.25046	19.25902	1.168347	0.111184	2.225511	20.16707	2.637428	-4.20858	0.034919	0.089678	1	0
468	sp|O70228	ATP9A_MO	119 kDa	0.998178	6	0.67	O70228	Atp9a	Probable ph	20.46668	19.03661	20.53157	19.15316	20.25761	19.58721	1.159627	0.481043	1.838212	19.83881	4.067386	-2.19606	0.005094	0.027196	1	1
730	sp|Q9CYN9	RENR_MOU	39 kDa	0.998453	4	1	Q9CYN9	Atp6ap2	Renin recep	21.33995	20.01686	20.3396	19.81024	21.11157	19.49734	1.15556	0.302683	2.008438	20.35259	3.229248	-3.35088	0.015248	0.05179	1	1
80	sp|Q3TEA8	HP1B3_MO	61 kDa	0.999402	17	1	Q3TEA8	Hp1bp3	Heterochro	24.89401	23.54595	25.31159	24.88947	25.29301	23.61559	1.149199	0.123926	2.174472	24.5916	2.674892	-4.15399	0.033112	0.086848	1	0
19	sp|Q8BTI8	SRRM2_MO	295 kDa	0.999729	29	1	Q8BTI8	Srrm2	Serine/argin	25.50006	24.34192	25.24684	24.57461	26.15066	24.54117	1.146622	0.369694	1.923551	25.05921	3.512693	-2.94644	0.010341	0.040881	1	1
236	sp|Q00PI9	HNRL2_MO	85 kDa	0.999487	9	1	Q00PI9	Hnrnpul2	Heterogene	23.46053	21.80072	23.27767	21.56276	22.95646	22.90901	1.140717	0.141444	2.13999	22.66119	2.724233	-4.08215	0.030882	0.082417	1	0
406	sp|P14869	RLA0_MOU	34 kDa	0.999608	6	1	P14869	Rplp0	60S acidic r	22.43835	21.56258	23.14436	21.87303	22.72625	21.48823	1.128374	0.39992	1.856827	22.20547	3.686818	-2.70623	0.008239	0.036481	1	1
681	sp|Q9D0W	PPIL1_MOU	18 kDa	0.999051	4	1	Q9D0W5	Ppil1	Peptidyl-pr	21.37567	20.23097	20.71761	20.84909	21.91929	19.55584	1.125558	0.019056	2.232059	20.77474	2.427541	-4.51467	0.04713	0.110037	1	0
907	sp|Q68FF6	GIT1_MOUS	85 kDa	0.998593	3	1	Q68FF6	Git1	ARF GTPase	20.14337	19.68871	20.58869	18.81858	21.31187	20.16075	1.125295	0.003098	2.247492	20.11866	2.39303	-4.56494	0.049528	0.114084	1	0
653	sp|P42225	STAT1_MOU	87 kDa	0.999283	4	1	P42225	Stat1	Signal trans	20.35865	19.5651	20.85073	19.40968	20.54185	19.40103	1.12514	0.475323	1.774957	20.02117	4.121132	-2.12573	0.004768	0.026134	1	1
110	sp|Q7TQG1	PKHA6_MO	131 kDa	0.999124	15	1	Q7TQG1	Plekha6	Pleckstrin h	23.65664	21.53064	22.01761	22.06882	23.09271	21.79914	1.122787	0.023842	2.221731	22.36093	2.438216	-4.49911	0.046413	0.109911	1	0
41	sp|Q9R0I7	YLPM1_MO	155 kDa	0.999814	22	1	Q9R0I7	Ylpm1	YLP motif-c	26.61066	24.99479	25.31427	25.07381	26.83904	25.33052	1.121616	0.053378	2.189854	25.69385	2.505688	-4.40073	0.042133	0.103689	1	0
343	sp|P68254	1433T_MO	28 kDa	0.99937	7	0.71	P68254	Ywhaq	14-3-3 prot	22.78802	21.55871	22.96714	21.86973	22.28267	21.25707	1.11744	0.350156	1.884724	22.12056	3.46632	-3.01109	0.010994	0.043027	1	1
656	sp|O70404	VAMP8_MO	11 kDa	0.999267	4	1	O70404	Vamp8	Vesicle-ass	25.10347	23.49243	24.69223	23.33267	24.8207	24.45309	1.112737	0.215775	2.009699	24.31577	2.957416	-3.74119	0.022164	0.066639	1	0
832.5	tr|A0A140T	A0A140T80	13 kDa	0.999312	3	0.33	A0A140T80	Igkv3-4	Immunoglo	23.40676	22.58069	23.36312	21.70752	23.16474	22.31016	1.112086	0.347275	1.876898	22.7555	3.460868	-3.0187	0.011074	0.043027	1	1
422	sp|O08538	ANGP1_MO	58 kDa	0.999433	6	1	O08538	Angpt1	Angiopoieti	21.66208	20.81513	22.21574	21.05985	21.15419	19.82284	1.111399	0.041083	2.181715	21.12164	2.478041	-4.44105	0.043835	0.106715	1	0
624	sp|Q9QYH6	MAGD1_MO	86 kDa	0.999491	4	1	Q9QYH6	Maged1	Melanoma-	20.44559	18.32565	20.25479	19.83698	20.48935	19.70369	1.107803	0.035008	2.180599	19.84268	2.464316	-4.46106	0.044706	0.108018	1	0
196	sp|Q80WJ7	LYRIC_MOU	64 kDa	0.999538	10	1	Q80WJ7	Mtdh	Protein LYR	24.06499	23.15208	24.28559	22.94461	24.34649	23.33512	1.088419	0.447462	1.729377	23.68815	4.041735	-2.22978	0.005258	0.02753	1	1
957	sp|Q99P58	RB27B_MO	25 kDa	0.997785	3	0.33	Q99P58	Rab27b	Ras-related	21.76208	21.26309	22.24039	20.23237	21.23287	20.50384	1.078678	0.103477	2.05388	21.20578	2.639661	-4.20533	0.034808	0.089564	1	0
1003	sp|Q8BX10	PGAM5_MO	32 kDa	0.995432	3	1	Q8BX10	Pgam5	Serine/threo	20.61479	19.25946	21.03657	20.16825	20.80334	19.797	1.076663	0.285382	1.867945	20.2799	3.238543	-3.3324	0.014922	0.051357	1	1
1142.2	tr|W4VSN7	W4VSN7_M	18 kDa	0.998518	2	1	W4VSN7	Gm3550	60S ribosor	21.53802	19.7664	22.0227	20.84075	21.50734	21.23563	1.075095	0.031224	2.118966	21.15181	2.457824	-4.47053	0.045125	0.108491	1	0
1143	sp|P41105	RL28_MOU	16 kDa	0.998509	2	1	P41105	Rpl28	60S ribosor	21.53802	19.7664	22.0227	20.84075	21.50734	21.23563	1.075095	0.031224	2.118966	21.15181	2.457824	-4.47053	0.045125	0.108491	1	0
740	sp|Q9Z108	STAU1_MO	54 kDa	0.998308	4	1	Q9Z108	Stau1	Double-stra	20.90502	19.94529	21.08713	19.76023	20.53657	19.60812	1.071692	0.391062	1.752321	20.30706	3.747658	-2.62327	0.007618	0.034782	1	1
671	sp|Q922H4	GMPPA_MO	46 kDa	0.999115	4	1	Q922H4	Gmppa	Mannose-1	21.88209	20.61914	21.71688	21.04773	21.85652	20.61797	1.056882	0.407984	1.70578	21.29006	3.876601	-2.44919	0.006465	0.031134	1	1
418	sp|P70372	ELAV1_MO	36 kDa	0.999519	6	1	P70372	Elavl1	ELAV-like pr	23.48833	22.39918	23.45239	22.14754	23.07889	22.30528	1.055871	0.408458	1.703284	22.81194	3.881775	-2.44225	0.006423	0.031104	1	1
486	sp|P61358	RL27_MOU	16 kDa	0.999784	5	1	P61358	Rpl27	60S ribosor	24.21536	22.86925	24.38481	23.44313	24.006	23.12912	1.054886	0.36499	1.744781	23.67461	3.639342	-2.77132	0.008762	0.037479	1	1
36	sp|E1U8D0	SOGA1_MO	159 kDa	0.99968	23	0.91	E1U8D0	Soga1	Protein SOG	23.38885	22.81849	22.73312	22.3781	23.91569	21.72737	1.037901	-0.00087	2.076668	22.82694	2.384452	-4.57743	0.050143	0.114541	1	0
159	sp|P62908	RS3_MOUS	27 kDa	0.999714	11	1	P62908	Rps3	40S ribosor	26.7488	25.49396	26.55744	25.63845	26.84899	25.94945	1.024457	0.369736	1.679178	26.20618	3.724245	-2.65514	0.007851	0.035516	1	1
463	sp|Q9DCD	SYF1_MOUS	100 kDa	0.998711	6	1	Q9DCD2	Xab2	Pre-mRNA-s	20.7791	20.10436	20.67987	20.2501	21.74623	19.77823	1.024172	0.131058	1.917286	20.55631	2.733759	-4.06589	0.030322	0.081418	1	0
85	sp|Q9CWF	TBB2B_MO	50 kDa	0.998684	17	0.06	Q9CWF2	Tubb2b	Tubulin bet	20.83364	19.967	21.31004	20.26951	20.2755	19.1449	1.012591	-0.00103	2.026213	20.3001	2.384014	-4.57807	0.050175	0.114541	1	0
157	sp|Q9D0E1	HNRPM_MO	78 kDa	0.999898	11	1	Q9D0E1	Hnrnpm	Heterogene	23.81454	23.01013	23.27448	22.16757	23.58094	22.45551	1.012249	0.218618	1.80588	23.05053	3.035775	-3.62314	0.019704	0.060451	1	0
583	sp|P84091	AP2M1_MO	50 kDa	0.997211	5	1	P84091	Ap2m1	AP-2 compl	23.81037	22.32336	23.57002	22.19383	22.90525	22.73735	1.010369	0.186655	1.834082	22.92336	2.919616	-3.79146	0.023167	0.067424	1	0
